# Comparative morphology and systematics of the cookiecutter sharks, genus *Isistius* Gill (1864) (Chondrichthyes: Squaliformes: Dalatiidae)

**DOI:** 10.1371/journal.pone.0201913

**Published:** 2018-08-20

**Authors:** Flávia de Figueiredo Petean, Marcelo R. de Carvalho

**Affiliations:** 1 Departamento de Botânica e Zoologia, Centro de Biociências, Universidade Federal do Rio Grande do Norte, Natal, RN, Brazil; 2 Departamento de Zoologia, Instituto de Biociências, Universidade de São Paulo, São Paulo, SP, Brazil; 3 American Museum of Natural History, New York, NY, United States of America; University of Michigan, UNITED STATES

## Abstract

The dalatiid genus *Isistius* Gill (1864) has three valid species currently recognized in the literature: *Isistius brasiliensis* Quoy & Gaimard (1824), *I*. *plutodus* Garrick & Springer (1964), and *I*. *labialis* Meng, Zhu & Li (1985). The most common species, *I*. *brasiliensis*, has a wide geographic distribution and is found in subtemperate and tropical seas circumglobally. A comparative analysis of specimens from different localities throughout its range, however, had never been undertaken. In the present paper, the morphological variation of this species along its entire distribution has been thoroughly analyzed, corroborating that it represents a single widespread species and that *I*. *labialis* is its junior synonym. The other congeneric species, *I*. *plutodus*, is known from only a few specimens and is also distributed worldwide. A detailed comparative analysis of available material of *I*. *plutodus* was conducted verifying its validity as a single widespread species. The present study analyzed in detail the external morphology (coloration, dentition, dermal denticles), internal morphology (skeleton, musculature), lateral-line canals, and morphometric and meristic characters of species of *Isistius* in order to better define the genus and its included valid species.

## Introduction

Sharks of the squalomorph shark family Dalatiidae are distributed worldwide and usually occur in open water ranging from the surface to more than 3,500 meters in depth. Dalatiid sharks have a highly conspicuous morphology, being characterized by a short snout without barbels, strong jaws [1], and lower teeth with strong, greatly developed cusps, as well as a striking dignathic heterodonty [2]. These sharks also have two small dorsal fins, with the first smaller or equal to the second, and both dorsal fins lack finspines (the only exception is *Squaliolus*, which has a very small spine in front of the first dorsal fin [3]). The anal fin is absent and the caudal fin is strongly asymmetrical, with its lower lobe varying from small to strongly conspicuous. Luminescent organs forming complex patterns might also be present [4].

Currently, there are seven genera in the family: *Dalatias* Rafinesque (1810) [5], *Euprotomicrus* Gill (1864) [6], *Isistius* Gill (1864) [6], *Squaliolus* Smith & Radcliffe (1912) [7], *Heteroscymnoides* Fowler (1934) [8], *Euprotomicroides* Hulley & Penrith (1966) [9], and *Mollisquama* Dolganov (1984) [10]. All genera are monospecific except *Squaliolus*, with two species, and *Isistius*, currently with three [1,11,12]

Cookiecutter sharks, genus *Isistius*, are recognized by having slender, cylindrical, cigar-shaped bodies and by their unique ectoparasitic feeding behavior that involves highly modified mandibular and hyoid arches and proportionally huge lower teeth [13,14]. Coloration in the genus *Isistius* is grey or brown, but with lighter fin tips [13,15]; however, the caudal fin in *Isistius brasiliensis* (Quoy & Gaimard, 1824) [16] has a darker region posteriorly. The dorsal side of the body is dark brown and the ventral side is slightly lighter. There is a darker brown collar around the branchial region that is clearly demarcated from the rest of the body, but which is currently described only for *I*. *brasiliensis* and *I*. *labialis* Meng, Zhu & Li (1985) [17]. The whole ventral surface, with the exception of the dark collar, has a net of small photophores that emit a green glow that can last up to three hours after death [13]. Bennett [18] described that after the death of a specimen the luminous glow completely disappears from the abdomen and only gradually from the rest of the body, remaining longer around the jaws and fins. In relation to sexual maturity of cookiecutter sharks, only data for *I*. *brasiliensis* have been reported. Parin [19] affirms that males are immature until 31.4 cm in total length (TL), while Jahn & Haedrich [20] state that maturity is only reached at 36 cm TL when the testes are fully developed. In females, Bigelow & Schroeder [21] and Jahn & Haedrich [20] mentioned distinct adults of 39 cm TL, Gadig & Gomes [22] examined a pregnant specimen of 43.1 cm TL, and Parin [19] reported an adult of 44 cm TL with seven eggs.

Specimens of *I*. *brasiliensis* are known to undertake dial vertical migrations [23], and even two human swimmers have been bitten by these sharks [24,25]. Strasburg [26] reviewed specimens collected from 63 to 200 m in depth and suggested they were collected in tropical regions and may utilize shallow regions as nursery areas. Widder [27] affirmed that the vertical migration of *I*. *brasiliensis* might be a behavioral adaptation that attracts visual preys, because the counterillumination allows the ventral collar to be more evident, being the only region that is photophore-free. Vertical migrations have also been suggested for the fossil species *Isistius triangulus* (Probst, 1879) [28] because fossil teeth were found in both shallow and deep-water paleoenvironments [29], meaning that this might be a plesiomorphic feature for cookiecutter sharks. *Isistius brasiliensis* was described by Quoy & Gaimard (1824) [16] based on a female specimen collected from the coast of Brazil. Nominal species currently considered junior synonyms of *I*. *brasiliensis* are [13]: *Scymnus (Scymnus) brasiliensis torquatus* Valenciennes [A.] in Müller & Henle (1839) [30]; *Scymnus (Scymnus) brasiliensis unicolor* Valenciennes [A.] in Müller & Henle (1839) [30]; *Squalus fulgens* Bennett (1840) [18], *Leius ferox* Kner (1864) [31], and *Isistius marmoratus* Rochebrune (1885) [32].

The poorly documented species *I*. *labialis* is reportedly very similar to *I*. *brasiliensis* [12,17,33]. The upper tooth rows are described as being more numerous (43 *vs*. 31–37); however, we counted 35 upper teeth (17+1+17) in the holotype in the South China Sea Fisheries Research Institute (Guangzhou, China). This species, whose holotype was collected just south of China in the South China Sea, is poorly known and probably oceanic, if valid. There are only two reported specimens: the holotype and a specimen collected close to Papua New Guinea (Pacific Ocean), deposited in the Florida Museum of Natural History (Gainesville, USA). If this species is valid, it might have a wider distribution than currently known and may be mistaken with *I*. *brasiliensis*, which has a worldwide distribution in tropical and subtropical waters.

*Isistius plutodus* Garrick & Springer (1964) [15], another cookiecutter shark species presently considered to be valid, is known to occur in the northern, southern, eastern, and western Atlantic Ocean, and in the northwestern and southwestern Pacific Ocean. Even though specimens are known from distinct oceans, *I*. *plutodus* is much more rare than *I*. *brasiliensis* as only a few specimens have been collected from each locality [34].

*Isistius plutodus* is also very similar to *I*. *brasiliensis*; however, it is reported as lacking the dark collar around the posterior region of the head, as well as the dark caudal fin tip [13,15]. Garrick & Springer [15] described *I*. *plutodus* from the Gulf of Mexico and were the first to revise the genus after detailed comparisons with specimens of *I*. *brasiliensis*. Recently, Stehmann & Kukuev [34] reported new records for this species from the southeastern Atlantic, and provided an historical summary of specimens known to date.

The aim of the present study is to taxonomically revise the species of the genus *Isistius* by means of a thorough comparative morphological study, promoting a more detailed definition of the genus and its valid species, and to precisely characterize the morphological variation present in the type-species *I*. *brasiliensis* (thereby elucidating the validity of *Isistius labialis* and other nominal species available in *Isistius*).

## Material and methods

The number of examined specimens of *I*. *brasiliensis* is 239 and eight of *I*. *plutodus*. Besides, 103 additional dalatiid specimens were also analyzed: 32 specimens of *Dalatias licha*, 15 of *Squaliolus aliae*, 23 of *Squaliolus laticaudus*, 31 of *Euprotomicrus bispinatus*, and one each of *Heteroscymnoides marleyi* and *Mollisquama parini* (S1 File). Other specimens, including type material deposited in collections in which access was not possible, were studied through data and photographs provided by collections staff (e.g. *Euprotomicroides zantedeshia* deposited in Hamburg). Among the examined specimens are included all holotypes of nominal species available in *Isistius*, with the exception of *I*. *marmoratus* Rochebrune (1885) [32] and *Squalus fulgens* Bennett (1840) [18] (these authors did not mention the whereabouts of their specimens, and they were not found in any collection or database).

Examined material is deposited in the following institutions: American Museum of Natural History, New York, USA (AMNH), Australian Museum, Sydney, Australia (AMS), Academy of Natural Sciences of Drexel University, Philadelphia, USA (ANSP), Academia Sinica, Taipei, Taiwan (ASIZP), Bishop Museum, Honolulu, USA (BPBM), California Academy of Sciences, San Francisco, USA (CAS), Natural History Museum and Institute, Chiba, Japan (CBM), Coleção Ictiológica do Departamento de Zoologia da Universidade do Estado do Rio de Janeiro, Rio de Janeiro, Brazil (UERJ), Commonwealth Scientific and Industrial Research Organisation, Hobart, Australia (CSIRO), Florida Museum of Natural History, Gainesville, USA (FLMNH), Field Museum of Natural History, Chicago, USA (FMNH), Fisheries Research Institute, Taipei, Taiwan (FRI), The Hokkaido University Museum, Hakodate, Japan (HUMZ), Iziko Museums of South Africa, Cape Town, South Africa (IZIKO), Museum of Comparative Zoology, Cambridge, USA (MCZ), Muséum national d'Histoire naturelle, Paris, France (MNHN), Museu Nacional da Universidade Federal do Rio de Janeiro, Rio de Janeiro, Brazil (MNRJ), Museu de Zoologia da Universidade de São Paulo, São Paulo, Brazil (MZUSP), Natural History Museum, Los Angeles, USA (LACM), Natural History Museum, London, England (NHM), Natural History Museum, Vienna, Austria (NMW), National Museum of Marine Biology and Aquarium, Checheng, Taiwan (NMMBA), Swedish Museum of Natural History, Stockholm, Sweden (NRM), National Museum of Nature and Science, Tsukuba, Japan (NSMT), The South African Institute for Aquatic Biodiversity, Grahamstown, South Africa (SAIAB), Scripps Institute of Oceanography, La Jolla, USA (SCRIPPS), South China Sea Fisheries Research Institute, Guangzhou, China (SCSFRI), Biodiversity Research and Teaching Collection, College Station, USA (TCWC), Tulane University, New Orleans, USA (TU), Universidade Federal da Paraíba, João Pessoa, Brazil (UFPB), National Museum of Natural History, Washington, D.C., USA (USNM), Burke Museum of Natural History and Culture, Seattle, USA (UW), Yale Peabody Museum of Natural History, New Haven, USA (YPM), Zoological Museum of the Zoological Institute of the Russian Academy of Sciences, Saint Petersburg, Russia (ZIN), Zoological Museum Hamburg Fish Collection, Hamburg, Germany (ZMH), Coleção de Peixes do Museu de Zoologia da Unicamp, Campinas, Brazil (ZUEC-PIS).

Analyzed specimens were preserved in alcohol 70%, isopropanol or formaldehyde 10%; for the comparative anatomical study, 18 partially or completely dissected specimens of *I*. *brasiliensis* and one of *I*. *plutodus* were analyzed, 70 *I*. *brasiliensis* and six *I*. *plutodus* were radiographed, and three *I*. *brasiliensis* and one *I*. *plutodus* were partially or totally cleared and stained [35]. Dissections were performed by direct incisions on the specimens using scalpels and forceps to examine the shape, size, origin and insertion of muscles, and to study the skeletal morphology and conduct counts, when necessary. Scanning Electron Microscopy (SEM) of dermal denticles of 12 specimens of *I*. *brasiliensis* and two of *I*. *plutodus* were made at the Instituto de Biociências of Universidade de São Paulo. To improve the observation of lateral line canals, these were injected with a saturated solution of methylene blue in 70% ethanol after skin removal.

Measurements are point-to-point and followed Compagno [13,36], and Last *et al*. [37] (Table 1). Vertebral counts are based on Compagno [38]. Terminology for skeleton and muscles followed Balfour [39], Compagno [40,41], Shirai [3], Shirai & Nakaya [14], de Carvalho *et al*. [42], and da Silva & de Carvalho [43]. Lateral-line canal terminology is based on Chu & Wen [44]. Dermal denticles followed Bigelow & Schroeder [21] and Kemp [45]; tooth morphology Adnet & Cappetta [2]; claspers and their muscles Jüngersen [46] and Gilbert & Heath [47]. Anatomical abbreviations are listed in the text and under each figure. Total length is abbreviated TL throughout, and head length as HDL. Values in percent given in diagnoses and descriptions refer to mean values, which were taken from all specimens.

**Table 1:** Morphometric measurements used in this study, with the corresponding abbreviations.

Morphometric character		Methodology
Total length	**TL**	Greatest direct distance between snout tip and caudal-fin apex
Precaudal length	**PCL**	Direct distance from snout tip to origin of upper caudal lobe
Pre-second dorsal length	**PD2**	Direct distance from snout tip to second dorsal-fin origin
Pre-first dorsal length	**PD1**	Direct distance from snout tip to first dorsal-fin origin
Pre-vent length	**SVL**	Direct distance from snout tip to anteror end of cloaca
Prepelvic length	**PP2**	Direct distance from snout tip to pelvic-fin origin
Prepectoral length	**PP1**	Direct distance from snout tip to exposed base of pectoral fin
Head length	**HDL**	Direct distance from snout tip to upper edge of the fifth gill slit
Prebranchial length	**PG1**	Direct distance from snout tip to upper edge of the first gill slit
Prespiracular length	**PSP**	Direct distance from snout tip to anteror margin of spiracle
Preorbital length	**POB**	Direct distance from snout tip to fleshy, anterior margin of orbit
Prenarial length	**PRN**	Direct distance from snout tip to anteror edge of outer nostril
Preoral length	**POR**	Direct distance from snout tip to upper jaw
Pre-inner nostril length	**PINL**	Direct distance from snout tip to inner edge of nostril
Inner nostril-labial furrow space	**INFL**	Shortest distance between nostrils and upper labial furrow
Mouth width	**MOW**	Distance between apices of labial pleats (junction of labbial furrows and postoral grooves)
Labial furrow length	**ULA**	Distance from apex of labial pleat to anteror edge of furrow
Internarial space	**INW**	Shortest distance between the two nostrils
Interorbital space	**INO**	Distance between softinterorbit in natural state (taken at midlength of eye)
Eye length	**EYL**	Length of eye, not including eye socket
Eye height	**EYH**	Height of eye
Spiracle length	**SPL**	Maximum width of opening
First gill-slit height	**GS1**	Vertical height of first gill slit (not following profile of gill)
Fifth gill-slit height	**GS5**	Vertical height of fifth gill slit (not following profile of gill)
Interdorsal space	**IDS**	Shortest distance between first dorsal-fin insertion and second dorsal-fin origin
Dorsal-caudal space	**DCS**	Shortest distance between second dorsal-fin insertion and origin of upper caudal lobe
Pectoral-pelvic space	**PPS**	Direct distance from pectoral-fin insertion to pelvic-fin origin (taken on ventral side)
Pelvic-caudal space	**PCA**	Direct distance from pelvic-fin insertion to origin of lower caudal lobe (taken on ventral side)
First dorsal length	**D1L**	Distance from first dorsal-fin origin to apex of free rear tip
First dorsal anterior margin	**D1A**	Distance from first dorsal-fin origin to point of greatest curvature of apex of fin
First dorsal base length	**D1B**	Distance from first dorsal-fin origin to first dorsal-fin inserton
First dorsal height	**D1H**	Greatest vertical height from fin base to apex of fin
First dorsal inner margin	**D1I**	Distance from first dorsal-fin insertion to apex of free rear tip
First dorsal posterior margin	**D1P**	Distance from points of greates curvature of the first dorsal-fin apex and apex of free rear tip
Second dorsal length	**D2L**	Distance from second dorsal-fin origin to apex of free rear tip
Second dorsal anterior margin	**D2A**	Distance from second dorsal-fin origin to point of greatest curvature of apex of fin
Second dorsal base length	**D2B**	Distance from second dorsal-fin origin to first dorsal-fin insertion
Second dorsal height	**D2H**	Greatest vertical height from fin base to apex of fin
Second dorsal inner margin	**D2I**	Distance from second dorsal-fin insertion to apex of free rear tip
Second dorsal posterior margin	**D2P**	Distance from points of greates curvature of the second dorsal-fin apex and apex of free rear tip
Pectoral anterior margin	**P1A**	Distance from pectoral-fin origin to apex of fin (measured from ventral surface)
Pectoral inner margin	**P1I**	Distance from pectoral-fin insertion to apex of free rear tip (measured from ventral surface)
Pectoral base length	**P1B**	Distance from pectoral-fin origin to pectoral-fin insertion (measured from ventral surface)
Pectoral posterior margin	**P1P**	Distance between points of greatest curvature of pectoral-fin apex and free rear tip (measured from ventral surface)
Pelvic length	**P2L**	Distance from pelvic-fin origin (use finger to find origin) to point of greatest curvature of apex (measured from ventral surface)
Pelvic heigth	**P2H**	Greatest width of pelvic fin (measured from ventral surface)
Pelvic inner margin	**P2I**	Distance from pelvic-fin insertion to apex of free rear tip (measured on ventral surface)
Dorsal caudal margin	**CDM**	Distance from origin of upper caudal lobe to point of greatest curvature of apex of dorsal caudal lobe
Preventral caudal margin	**TLV**	Distance from origin of lower caudal lobe to point of greatest curvature of apex of ventral caudal lobe
Upper postventral caudal margin	**TLU**	Distance from greatest angle of caudal fork to point of greatest curvature of apex of dorsal caudal lobe
Lower postventral caudal margin	**TLL**	Distance from greatest angle of caudal fork to point of greatest curvature of apex of ventral caudal lobe
Caudal fork width	**CFW**	Perpendicular distance from greatest angle of caudal fork to dorsal caudal margin
Caudal fork length	**CFL**	Distance from greatest angle of caudal fork to origin of lower caudal lobe
Head width at nostrils	**HANW**	Width of head at anterior margin of nostrils
Head width at mouth	**HAMW**	Width of head at level of anterior margin of mouth
Head width	**HDW**	Width of head at fifth gill slit
Trunk width	**TRW**	Width of body at pectoral-fin insertions
Abdomen width	**ABW**	Width of body at first dorsal-fin insertion
Tail width	**TAW**	Width of body at pelvic-fin insertions
Caudal peduncle width	**TLW**	Width of caudal peduncle in front of caudal groove
Head heigth	**HDH**	Vertical height of head at fifth gill slit
Trunk heigth	**TRH**	Vertical height of body at pectoral-fin insertions
Abdomen heigth	**ABH**	Vertical height of body at first dorsal-fin insertion
Tail heigth	**TAH**	Vertical height of body at pelvic-fin insertions
Caudal peduncle heigth	**TLH**	Vertical height of caudal peduncle in front of caudal groove
Clasper outer length	**CLO**	Distance between lateral junction of pelvic-fin inner margin to apex of clasper
Clasper inner length	**CLI**	Distance between connecton of the clasper base dorsally with the tail to apex of clasper
Clasper base width	**CLB**	Width of clasper at pelvic-fin insertion

Maps of distribution were built on the software QGIS 2.18.14 (QGIS Development Team (2018). QGIS Geographic Information System. Open Source Geospatial Foundation Project. http://qgis.osgeo.org) using the georeferenced sites of collection of known specimens on a base map with topology and bathymetry from NASA.

Measurements follow Compagno [13,36] and Last *et al*. [37], and were taken with a precision of 0.01 mm except for measurements marked with an asterisk (*), which were taken with a precision of 1 mm. Measurements of “width” were used with caution due to deformation of specimens as a result of preservation.

## Results

All morphological characteristics and measurements of the holotype of *I*. *labialis* fall within the range encountered for *I*. *brasiliensis*, corroborating that it is a junior synonym of the latter species (Table 2).

**Table 2:** Morphometric characterization of *Isistius brasiliensis*.

	MNHN A-7787		MNHN 0000–1178		NMW 76230		SCSFRI S07257		n	Range				Mean	SD
	mm	%TL	mm	%TL	mm	%TL	mm	%TL		mm		%TL		%TL	%TL
**TL**	172.00	-	471	-	162	-	442	-	226	112.14	520.00	-	-	335.54	-
**PCL**	133.35	77.53	404	85.77	131.95	81.45	381	86.20	225	97.83	445	77.06	138.76	85.14	4.48
**PD2**	109.76	63.81	343	72.82	109.91	67.85	317	71.72	226	31	378	7.19	101.56	70.83	5.04
**PD1**	90.05	52.35	285	60.51	91.68	56.59	262	59.28	226	69.04	317	52.35	66.15	59.41	1.77
**SVL**	97.89	56.91	304	64.54	102.28	63.14	294	66.52	220	74.23	340	54.07	70.21	63.92	2.48
**PP2**	95.41	55.47	288	61.15	95.15	58.73	280	63.35	226	69.29	326	52.91	80.56	60.74	2.79
**PP1**	34.46	20.03	94.62	20.09	36.9	22.78	89.31	20.21	226	24.16	100.36	16.96	25.81	20.61	1.31
**HDL**	35.11	20.41	96.38	20.46	35.85	22.13	88.98	20.13	224	24.89	102.11	17.40	25.11	20.84	1.25
**PG1**	28.71	16.69	77.52	16.46	30.13	18.60	72.6	16.43	223	19.93	83.51	13.91	20.82	16.95	1.08
**PSP**	15.1	8.78	37.23	7.90	16.33	10.08	38.16	8.63	222	10.5	41.94	7.16	11.16	8.95	0.74
**POB**	4.87	2.83	13.63	2.89	6.39	3.94	13.13	2.97	222	3.08	17.01	1.82	4.15	3.24	0.37
**PRN**	2.29	1.33	6.75	1.43	2.72	1.68	5.57	1.26	215	0.83	7.9	0.49	3.44	1.31	0.27
**POR**	11.08	6.44	24.07	5.11	12.02	7.42	26.64	6.03	207	6.5	35.08	5.11	11.75	6.77	0.77
**PINL**	2.46	1.43	8.31	1.76	3.71	2.29	7.23	1.64	199	0.99	10.2	0.39	2.45	1.56	0.32
**INFL**	19.19	11.16	27.07	5.75	11.55	7.13	25.69	5.81	212	6.43	31.61	2.50	11.16	6.44	0.93
**MOW**	17.08	9.93	41.8	8.87	10.79	6.66	32.33	7.31	206	8.58	41.8	5.86	11.57	7.41	0.89
**ULA**	8.04	4.67	29.13	6.18	8.98	5.54	23.82	5.39	213	5.69	29.13	2.29	9.29	5.22	0.80
**INW**	2.79	1.62	6.57	1.39	3.39	2.09	5.11	1.16	214	1.44	7.6	0.75	2.09	1.39	0.18
**INO**	8.36	4.86	21.11	4.48	9.61	5.93	22.49	5.09	223	6.21	28.63	3.91	6.56	5.31	0.49
**EYL**	7.69	4.47	16.89	3.59	7.71	4.76	13.79	3.12	213	4.57	17.76	2.92	5.46	3.86	0.53
**EYH**	4.44	2.58	10.48	2.23	4.51	2.78	8.88	2.01	213	2.87	13.46	1.70	3.86	2.64	0.39
**SPL**	4.01	2.33	10.66	2.26	3.69	2.28	8.61	1.95	223	1.81	10.68	1.50	2.96	2.14	0.27
**GS1**	1.51	0.88	3.3	0.70	1.2	0.74	4.2	0.95	217	1.13	6.53	0.52	1.46	1.02	0.16
**GS5**	1.25	0.73	3.26	0.69	1.13	0.70	2.87	0.65	218	0.6	5.11	0.35	1.81	0.80	0.16
**IDS**	15.19	8.83	44.88	9.53	13.85	8.55	40.19	9.09	225	8.93	51.03	6.95	11.01	9.10	0.81
**DCS**	21.48	12.49	45.03	9.56	17.99	11.10	48.24	10.91	225	9.19	54.25	7.61	13.23	10.48	0.98
**PPS**	53.24	30.95	178	37.79	57.48	35.48	168.94	38.22	225	40.04	214	28.90	44.62	36.80	2.34
**PCA**	28.73	16.70	72.63	15.42	27.29	16.85	78.37	17.73	225	20.08	93.61	14.46	20.83	17.74	1.22
**D1L**	9.98	5.80	30.23	6.42	12.4	7.65	30.9	6.99	221	4.86	34.36	4.20	8.16	6.39	0.61
**D1A**	7.16	4.16	18.51	3.93	9.86	6.09	21.7	4.91	221	3.16	27.15	2.82	6.09	4.76	0.51
**D1B**	5.33	3.10	13.56	2.88	5.64	3.48	14.28	3.23	225	3.51	17.35	2.16	4.54	3.04	0.40
**D1H**	2.55	1.48	8.97	1.90	2.43	1.50	8.31	1.88	220	1.99	14.9	1.19	3.51	1.77	0.29
**D1I**	3.84	2.23	17.63	3.74	7.12	4.40	17.43	3.94	222	0.57	19.7	0.15	4.40	3.36	0.47
**D1P**	4.15	2.41	15.69	3.33	3.45	2.13	13.94	3.15	218	2.06	19.41	1.26	4.24	2.82	0.42
**D2L**	9.07	5.27	32.13	6.82	13.33	8.23	31.82	7.20	224	6.11	37.91	3.73	8.99	6.96	0.66
**D2A**	8.24	4.79	15.26	3.24	9	5.56	20.16	4.56	226	3.5	24.48	3.02	5.92	4.53	0.51
**D2B**	5.99	3.48	15.07	3.20	6.78	4.19	15.47	3.50	226	4.14	19.23	2.69	4.98	3.50	0.42
**D2H**	2.49	1.45	8.92	1.89	3.5	2.16	9.29	2.10	225	1.47	15.4	1.27	3.80	2.01	0.30
**D2I**	2.7	1.57	17.23	3.66	7.46	4.60	16.44	3.72	224	1.61	20.64	1.17	4.60	3.54	0.51
**D2P**	3.99	2.32	21.01	4.46	5.63	3.48	16.55	3.74	222	2.19	21.36	1.92	4.94	3.48	0.48
**P1A**	10.04	5.84	27.04	5.74	14.67	9.06	30.49	6.90	226	6.19	41.72	5.41	10.53	7.99	0.97
**P1I**	7.28	4.23	21.14	4.49	9.95	6.14	15.52	3.51	226	1.68	101	0.74	6.53	4.46	0.75
**P1B**	7.66	4.45	18.9	4.01	5.72	3.53	16.13	3.65	226	3.2	21.44	2.65	4.70	3.58	0.37
**P1P**	4.14	2.41	18.69	3.97	10.64	6.57	19.8	4.48	226	4.03	30.12	2.41	6.63	4.79	0.76
**P2L**	9.27	5.39	37.74	8.01	15.51	9.57	37.06	8.38	226	3.37	47.98	2.89	11.84	8.74	1.40
**P2H**	3.63	2.11	15.27	3.24	5.37	3.31	16.04	3.63	218	2.35	23.65	1.32	5.02	2.71	0.73
**P2I**	2.17	1.26	12.68	2.69	5.91	3.65	13.75	3.11	223	1.91	26.51	0.50	10.11	2.83	1.40
**CDM**	26.42	15.36	66.81	14.18	27.02	16.68	59.69	13.50	224	7.15	81.19	6.01	17.86	15.01	1.28
**CPV**	20.22	11.76	48.49	10.30	19	11.73	46.77	10.58	221	7.62	59.8	6.66	13.69	10.74	0.99
**CPU**	13.6	7.91	42.63	9.05	15.66	9.67	34.98	7.91	216	4.9	53.19	2.81	11.41	9.56	1.11
**CPL**	6.55	3.81	27.48	5.83	8.46	5.22	21.95	4.97	216	2.58	33.26	2.27	9.51	5.55	0.70
**CFW**	11.44	6.65	28.11	5.97	9.86	6.09	25.04	5.67	219	5.24	34.05	4.44	7.43	6.07	0.49
**CFL**	15.69	9.12	38.54	8.18	16.7	10.31	35.21	7.97	215	7.89	41.84	6.69	15.35	8.57	0.88
**HANW**	3.65	2.12	12.15	2.58	4.41	2.72	11.37	2.57	213	2.16	18.6	1.90	4.59	2.64	0.36
**HAMW**	17.21	10.01	44.98	9.55	14.6	9.01	39.9	9.03	213	10.76	44.98	6.95	15.78	9.05	0.90
**HDW**	13.54	7.87	46.68	9.91	11.92	7.36	48.28	10.92	217	7.75	57.45	6.81	13.74	9.46	0.88
**TRW**	11.44	6.65	43.27	9.19	11.89	7.34	45.05	10.19	203	6.87	57.17	4.13	11.34	8.51	1.12
**ABW**	7.9	4.59	21.92	4.65	7.02	4.33	27.15	6.14	209	3.59	31.77	2.48	6.89	4.69	0.70
**TAW**	7.06	4.10	21.02	4.46	5.68	3.51	21.65	4.90	220	3.41	30.63	2.08	6.64	4.33	0.65
**CPW**	3.02	1.76	7.81	1.66	3.22	1.99	7.7	1.74	220	1.13	13.41	0.91	2.82	1.88	0.32
**HDH**	10.79	6.27	38.82	8.24	14.26	8.80	36.31	8.21	210	8.2	47.86	4.71	11.83	8.10	1.08
**TRH**	11.52	6.70	39.97	8.49	13.48	8.32	35.89	8.12	200	8.59	53.32	4.38	12.06	8.27	1.32
**ABH**	7.19	4.18	30.66	6.51	7.44	4.59	30.53	6.91	209	5.23	41.47	3.74	9.00	5.97	0.95
**TAH**	6.33	3.68	20.46	4.34	7.83	4.83	25.81	5.84	220	4.59	31.81	2.93	6.90	4.56	0.63
**CPH**	3.15	1.83	9.74	2.07	3.14	1.94	8.8	1.99	220	2.32	11.61	1.35	2.50	1.91	0.20
**CLO**	-	-	-	-	-	-	-	-	107	1.36	12.91	0.74	3.23	1.98	0.73
**CLI**	-	-	-	-	-	-	-	-	107	2.3	32.58	0.84	8.20	5.16	1.78
**CLB**	-	-	-	-	-	-	-	-	107	0.47	7.24	0.26	1.88	0.95	0.40

It includes holotypes of the junior synonyms *Scymnus brasiliensis* (MNHN A-7787), *Scymnus brasiliensis unicolor* (MNHN 0000–1178), *Leius ferox* (NMW 76230), and *I*. *labialis* (SCSFRI S07257). All values are presented as percentages of total length (TL), except TL, given in mm.

Taxonomy and redescriptions of cookiecutter sharks within the family Dalatiidae Gill (1893) [48] and order Squaliformes Compagno (1973) [49]:

### *Isistius* Gill (1864) [6]

*Isistius* Gill (1864): 264 (gender masculine; type species: *Scymnus brasiliensis* Müller & Henle (1841) by monotypy; equals *Scymnus brasiliensis* Quoy & Gaimard, 1824) [6].

*Leius* Kner (1864): 9 (gender masculine; type species: *Leius ferox* Kner (1864) by monotypy) [31]; Günther (1909): 490 (brief description) [50].

#### Diagnosis

**Fig 1:**
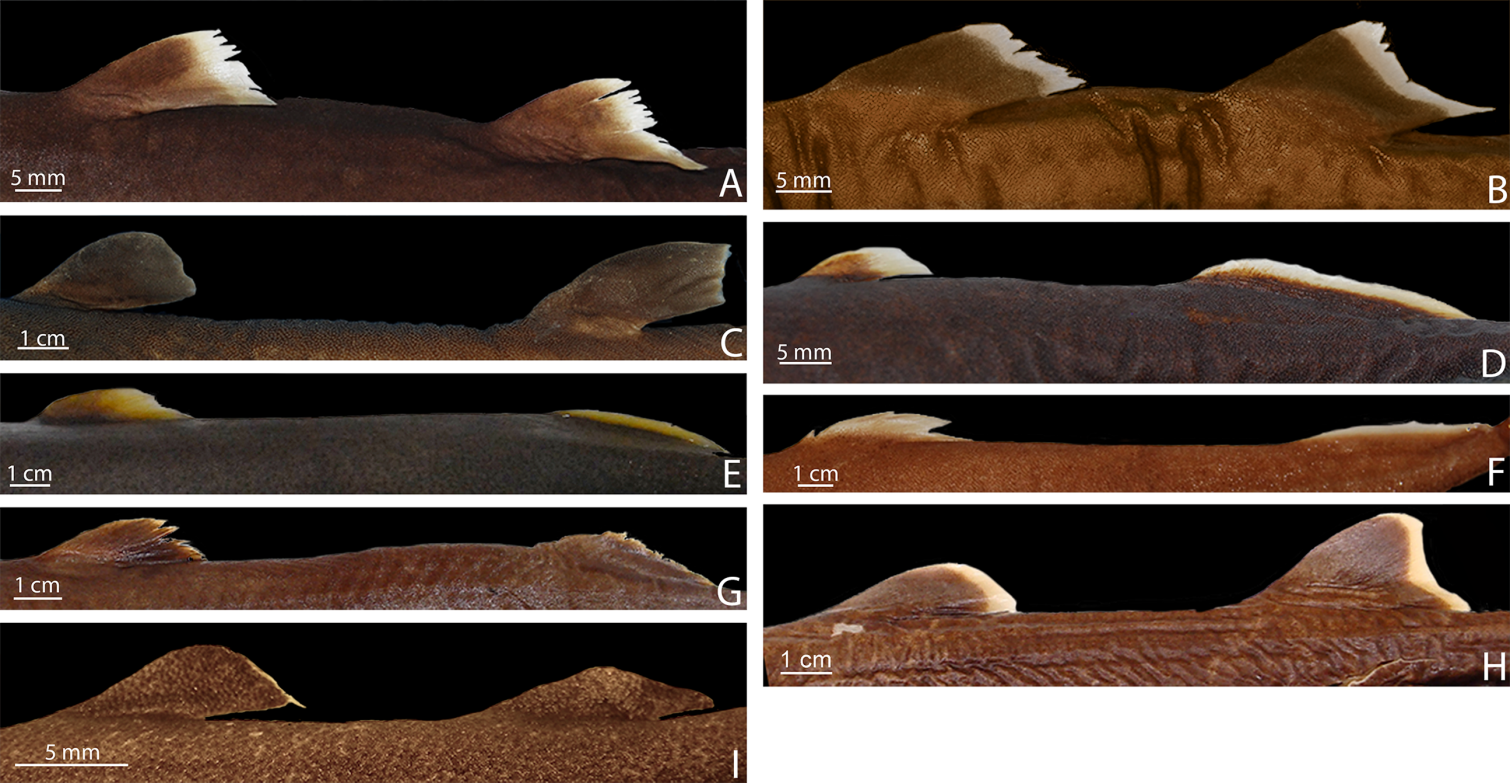
Dorsal fins of species of Dalatiidae. (A) *Isistius brasiliensis* (CSIRO H 4714–01); (B) *I*. *plutodus* (AMS 43044–001); (C) *Dalatias licha* (AMS 43469–001); (D) *Squaliolus laticaudus* (CSIRO H1215-01); (E) *S*. *aliae* (NSMT-P 35505); (F) *Euprotomicrus bispinatus* (NSMT-P 71062); (G) *Euprotomicroides zantedeschia* (ZMH 114732); (H) *Heteroscymnoides marleyi* (ZMH 123459); (I) *Mollisquama parini* (TU 203676).

Externally, *Isistius* is distinguished from the remaining dalatiid genera mainly by the presence of a dark collar on the ventral side of the head, posterior to the mouth, and extending dorsolaterally to surround the gill openings, and by the cylindrical body, with a mean length of 34.6 cm and mean trunk height at pectoral fin insertion of 2.78 mm. Both dorsal fins in *I*. *brasiliensis* and *I*. *plutodus* are located relatively posteriorly, with the origin of the first dorsal fin at the vertical line just anterior to pelvic-fin origin (Fig 1). The distance between dorsal-fin origins varies from 6 to 9% of TL, and origin of the second dorsal is, approximately, at the vertical line of the free rear tip of pelvic fins. Both dorsal fins have similar size and shape (second dorsal is only slightly taller, less than 1% of TL).

Lower teeth are proportionately greater than the upper teeth (3.2 to 6.2 times); lower teeth interlocked, in a single functional row, rectangular at base and triangular at apex, not serrated, and with a very upright main cusp. Upper teeth pointed, slightly inclined toward the posterior region of mouth, not serrated, and arranged in three functional rows. Spiracle length is greater than 10% of head length, proportionately the greatest among all dalatiids (*Heteroscymnoides marlyei* has the smallest spiracle at 4.36% HDL; mean size in the family is 8.28% HDL).

Regarding anatomical features, *Isistius* is easily differentiated from all other dalatiids by a large fenestra in the interorbital wall, which is covered by a membrane (other genera have a continuous cartilaginous wall); lack of the optic pedicel; a single pair of hypobranchials and a single or divided basibranchial plate in the branchial region; presence of gill-pickax formed by the fusion of pharyngobranchials 4 and 5 and epibranchial 5.

#### Comparison with other dalatiids

The dorsal fins in *Dalatias* are also of similar size and shape, but the origin of the first dorsal is at the vertical line of the pectoral fin free rear tip, and origin of second dorsal is at the vertical line of pelvic fin insertion, with an interdorsal space of 20% TL. In *Euprotomicroides*, first dorsal fin origin is posterior to the vertical at the free rear tip of the pectoral fins, second dorsal fin originates at vertical through origin of pelvic fins, and the second dorsal fin has a longer base but is not as tall as the first dorsal. In *Euprotomicrus*, the second dorsal fin is twice the size of the first, and originates at midlength of body between insertion of pectoral fin and origin of pelvic fin, whereas the second dorsal fin originates at the vertical line through pelvic fin insertion. Both dorsal fins also have similar sizes in *Heteroscymnoides*, and the first dorsal originates at the vertical line through pectoral fin insertion, whereas the second dorsal fin originates at insertion of pelvic fins. In *Mollisquama*, both dorsal fins are close together, have similar sizes and relative positions as in *Isistius*, but the second dorsal fin is slightly lower than the first (contrasting with *Isistius*). In *Squaliolus*, the first dorsal fin has a small spine (its length 0.5 to 2% TL) and is, approximately, 0.7 times smaller than the second dorsal fin; dorsal fins have a relatively wide space between them (2.5 times greater than in *Isistius*), and the first dorsal originates immediately posterior to the vertical through pectoral fin insertion, whereas the second dorsal originates at the vertical through pelvic-fin insertion.

**Fig 2:**
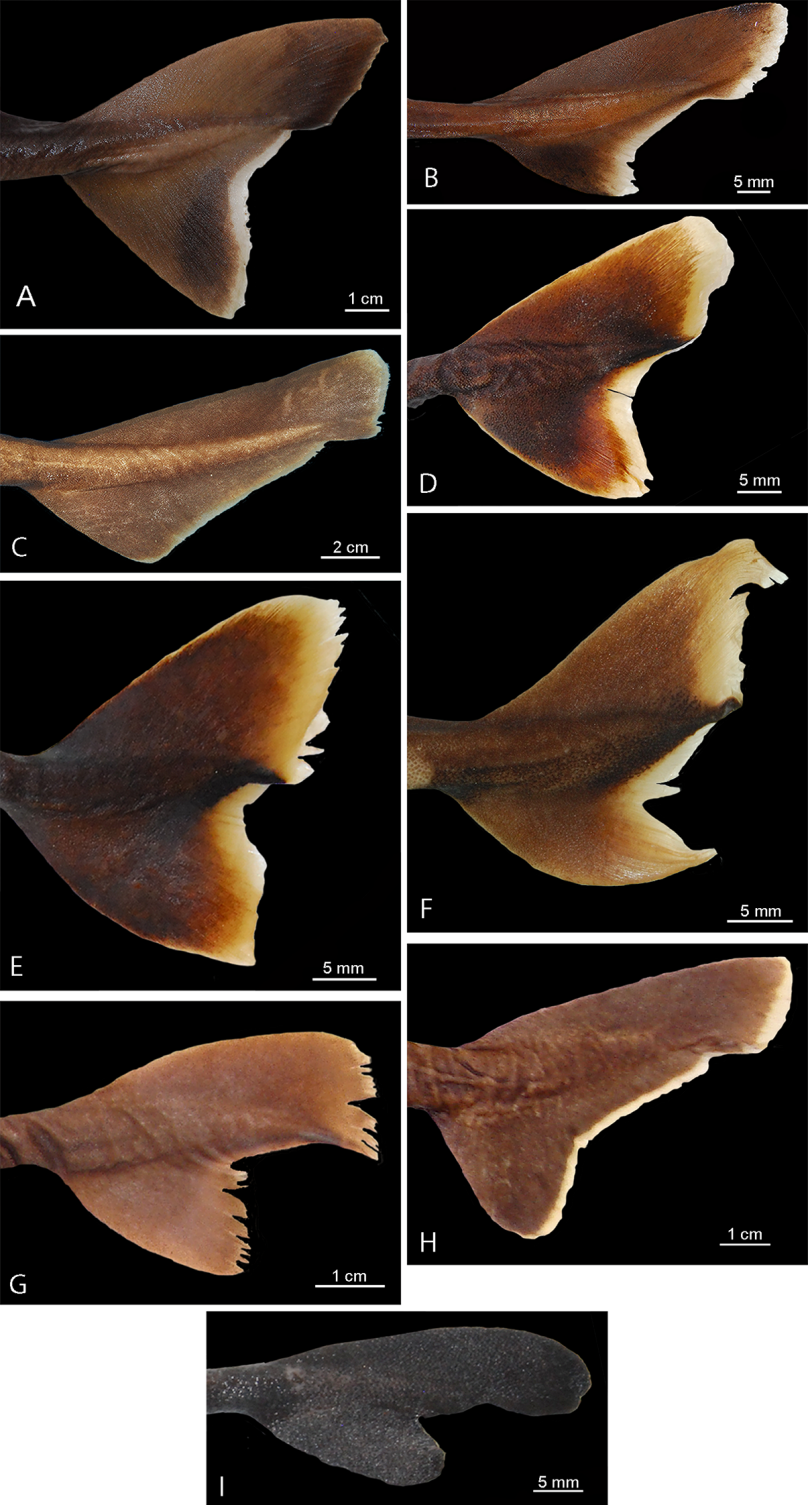
Caudal fins of species of Dalatiidae. (A) *Isistius brasiliensis* (HUMZ 211104); (B) *I*. *plutodus* (HUMZ 210817); (C) *Dalatias licha* (AMS 12876); (D) *Squaliolus laticaudus* (MNRJ 30199); (E) *S*. *aliae* (NSMT-P 35505); (F) *Euprotomicrus bispinatus* (NSMT-P 71062); (G) *Euprotomicroides zantedeschia* (ZMH 114732); (H) *Heteroscymnoides marleyi* (ZMH 108438); (I) *Mollisquama parini* (TU 203676).

Caudal fin morphology in dalatiids is very uniform as all species have similar caudal shapes, except *Dalatias*, whose postventral margin is not subdivided into upper and lower portions and is much longer than the preventral margin (Fig 2). There are some differences in proportions, such as the dorsal caudal margin that is 1.4 times larger than the lower caudal margin in *I*. *brasiliensis* (*vs*. 2.4 times in *I*. *plutodus*, *vs*. 1.8 in *Dalatias*, *vs*. 1.7 in *Mollisquama*, and 1.2 in *Euprotomicrus*, *Heteroscymnoides*, and *Squaliolus*).

Lower and upper dentition of *Dalatias* is very similar to *Isistius*; however, the proportions are different (lower teeth only 2.4 times greater), and its lower teeth are serrated and slightly inclined toward the back of mouth. In *Euprotomicroides*, *Euprotomicrus*, *Heteroscymnoides*, and *Squaliolus* the lower teeth, although similarly shaped to *Isistius*, are relatively narrower (2.8 times greater than upper teeth in *Euprotomicrus*, only 1.5 times in *Squaliolus*), besides having one side of the tooth straight and the other inclined, not symmetrical. In *Mollisquama*, the lower teeth, although presenting triangular apices, do not have serrations, are narrower, more pointed, and more directed toward mouth corners than in *Isistius*, while the upper teeth are also similar to those in *Isistius* in shape but thinner and proportionately longer.

#### Etymology

The generic name *Isistius* comes from the Greek words *isos* (equal) and *istios* (sail), which is a reference to the almost symmetrical caudal fin [51].

#### Taxonomic history

Linnaeus [52] described the genus *Squalus* for fishes with five gill slits situated on the side of the head, lacking anal fin, with a long and tapering body, and terminal mouth, in which he included all known shark species. Broussonet [53] described a species, *La Liche*, not yet using the binomial nomenclature proposed by Linnaeus. It had spiny skin, lacked dorsal spines (different from *Squalus*), had ventral fins very close to the tail, and the second dorsal fin was greater than the first. This species of Broussonet cannot be considered a synonym of any valid species of *Isistius*; however, it was the foundation for subsequent descriptions of certain sharks, since *La Liche* can be considered the equivalent of *Dalatias licha*.

Bonnaterre [54] subdivided fishes into groups and described *Squalus licha* based on *La Liche* of Broussonet [53]; he placed it in his First Class (Cartilaginous Fishes) and Third Genus (*Squalus*, specimens with 4–7 lateral openings and 7 or 8 fins, as the anal may be absent). Cuvier [55] described the subgenus *Scymnus* as part of the genus *Squalus*, which is equivalent to *La Liche* Broussonet (1780) [53], for *Squalus americanus* Gmelin (1788) [56] and *Squalus licha* Bonnaterre (1788) [54]. Some of the characters used in Cuvier's description are second dorsal fin above the pelvic fins, sharp lower teeth arranged in one or two rows, and upper teeth narrow, pointed, and in many rows.

Quoy & Gaimard [16] described two new species for the genus *Scymnus* Cuvier (1817) [55]: one based on a male specimen from the Mauritius, *Scymnus bispinatus*, and another based on a very small female specimen from Brazil, *Scymnus brasiliensis*, which had the body shape of *S*. *bispinatus* but its lower jaw was disproportionately large, had its mouth closer to the snout, dorsal fins larger, both caudal lobes deeply divided with a light brown color, and a wide darker band on the ventral side of the head.

Bennett [18] described two bioluminescent specimens in detail with characteristics of what is currently known as *Isistius*. He named them *Squalus fulgens*, following the generic concept of Linnaeus [52], without mentioning the descriptions of Cuvier [55] and Quoy & Gaimard [16], who had already noticed a new group of specimens slightly distinct from *Squalus* and described new genera and species for them. Bennett indicated *fulgens* was a new species of *Squalus* that belonged to the subgenus *Scymnus* and he suggested it be called *Squalus fulgens*. Besides, he did not mention the wherabouts of these specimens, making it impossible to evaluate their identification as species of *Isistius*.

Müller & Henle [30], in their extensive revision of elasmobranchs, created the family Scymni, which encompassed the genera *Scymnus* Cuvier, *Echinorhinus* Blainville, and *Pristiophorus* Müller & Henle. The former was divided into two subgenera: *Scymnus* and *Laemargus*. Two species were considered as belonging to the subgenus *Scymnus*: *Scymnus (Scymnus) Lichia* Bonaparte and *Scymnus (Scymnus) brasiliensis* Cuvier. Müller & Henle [30] described the species *Scymnus (Scymnus) brasiliensis* with the following characters: nasal flap present at the end of the snout and lower teeth smooth, not serrated; rounded and small pectoral fins, which were wider at the tip than at the base; squared pelvic fins, with rounded angles at the front and with a pointy posterior part; first dorsal right before the origin of the pelvic fins; second dorsal in between the first dorsal fin and the base of the caudal fin; both dorsal fins rounded anteriorly and prolonged posteriorly in an acute angle, with a straight upper margin; both dorsal fins very small and the same height; second dorsal base length greater than the first; lower lobe of caudal fin relatively large; scales small, without a distinct point, and with a depression at their middle.

Muller & Henle [30] divided the species into two varieties, which had been suggested by Valenciennes: *Scymnus (Scymnus) brasiliensis torquatus* and *Scymnus (Scymnus) brasiliensis*. The former had a brown color, being lighter on the ventral side, and with a wide darker band bellow the head; the pectoral fin margins and the lower part of the caudal fin were white. The second specimen had the same coloration as the first one, but the pelvic fins were darker and it lacked the darker band beneath the head. Both type specimens are located in the Muséum national d’Histoire naturelle in Paris (the type specimen of *Scymnus brasiliensis* Quoy & Gaimard is the same specimen that is the type of *S*. *(S*.*) b*. *unicolor*).

Kner [31] described a new genus, *Leius*, and species, *Leius ferox*, whose holotype is at the Natural History Museum in Vienna. In this specimen, the upper lobe of the caudal fin is truncated and the coloration is a dark brown. The author suggested that this specimen belonged to a group close to *Scymnus* and *Laemargus*, but very different from *Scymnus bispinatus* Quoy & Gaimard (a synonym of *Euprotomicrus bispinatus* (Quoy & Gaimard, 1824) [16]) and from *Somniosus brevipinna* Les? [sic] (a synonym of *Somniosus microcephalus* (Bloch & Schneider, 1801)).

However, Gill [6], in a synopsis of sharks from eastern North America, described the new genus *Isistius* in the family Scymnoidea based on the species *Scymnus brasiliensis* Müller & Henle, which was distinctive by its similar dorsal fins that were posteriorly located on the body. Later, Gill [57] explained the reason why he proposed the name *Isistius* to replace *Scymnus* because the latter was preoccupied by Coleoptera Kugelmann (1794) (*apud* [57]).

Rochebrune [32] described another species in the genus *Isistius* Gill, *I*. *marmoratus*. This species was described as having a narrow and rounded body, obtuse snout, small and inferior mouth, short teeth, and first dorsal fin at the posterior third of the body. Although Rochebrune indicated the specimen was located in the Museo Bouvieri and was collected in "Landana and the entire Gambia", both the museum and the site of collection could not be found. Eugène-Louis Bouvier (1856–1944) was a professor of entomology at the Paris Museum (MNHN) from 1895 to 1931, and no 'Bouvier collection' is known at the MNHN (Paris). Hence, the types supposedly deposited in this collection may be considered lost [58].

Garrick & Springer [15] described another *Isistius* species, *I*. *plutodus*, with the dental formula 14+1+14/9+1+9; presence of a short caudal peduncle and small caudal fin, having the lower lobe half the length of the upper lobe; second dorsal fin remarkably taller than the first; no well-defined dark collar, and without caudal fin markings.

Meng, Zhu & Li [17] described the most recent species of the genus, *Isistius labialis*. They proposed it could be differentiated from *I*. *plutodus* by having the dark collar and 10 more teeth in the lower jaw; and from *I*. *brasiliensis* by having folds at the lower lip and by the length of the pelvic fins, equal to the first and second dorsal fins, whereas in *I*. *brasiliensis* they are supposedly greater.

With regards to the nomenclature of the family Dalatiidae, Gray [59] is supposed to have coined the family name, as he wrote a list of specimens and divided them in successively inclusive groups. However, he mentioned a subfamily called Dalatiana that encompassed those sharks that lack dorsal spines. Within this subfamily, he included two genera: *Dalatias* and *Echinorhinus*. In *Dalatias* he included the species *Dalatias lichia* (= *Dalatias licha*) and *Dalatias brasiliensis*. The latter can be regarded as a synonym of *Isistius brasiliensis*.

Gray [59] was the first to ever use the name “*Dalatias*” to determine and identify a group of sharks since this name became available in 1810 when Rafinesque described the new genus and both species *Dalatias Sparophagus* and *Dalatias Nocturnus*. However, the first author to use “Dalatiidae” was Gill [48] when he included within the group the subfamily Dalatiana Gray (1851) [59], modifying it to Dalatiina, and the subfamily Somniosina.

Another issue regarding the nomenclature for both *Isistius* and *Euprotomicrus* is a mistake concerning the year of publication of Gill’s paper describing them. It is currently held that both were described in 1865 [13]; however, this work by Gill was published in 1864 [6]. In an article entitled “Synopsis of the Eastern American Sharks” he includes a synonymy and, on page 264, in the synonymy of *Somniosus microcephalus*, he mentions the family Scymnoidae and puts an asterisk besides it to indicate in a footnote its included genera. Among these genera he includes *Isistius* and *Euprotomicrus*, two names that had never been used before. He writes a short description of *Isistius* (p. 264): “it is distinguished by its similar posterior dorsals and caudal fins”, and he places *Scymnus brasiliensis* Müller & Henle (1839) [30] in it. Gill does the same with *Euprotomicrus* (p. 264): “teeth like *Somniosus*, but in moderate number and very small first dorsal”, and places *Scymnus labordii* Müller & Henle (1839) [30] in it. Therefore, when referring to the genera *Isistius* and *Euprotomicrus*, the correct author and date for these names is Gill (1864) [6].

### *Isistius brasiliensis* (Quoy & Gaimard, 1824) [16]

(Fig 3)

**Fig 3:**
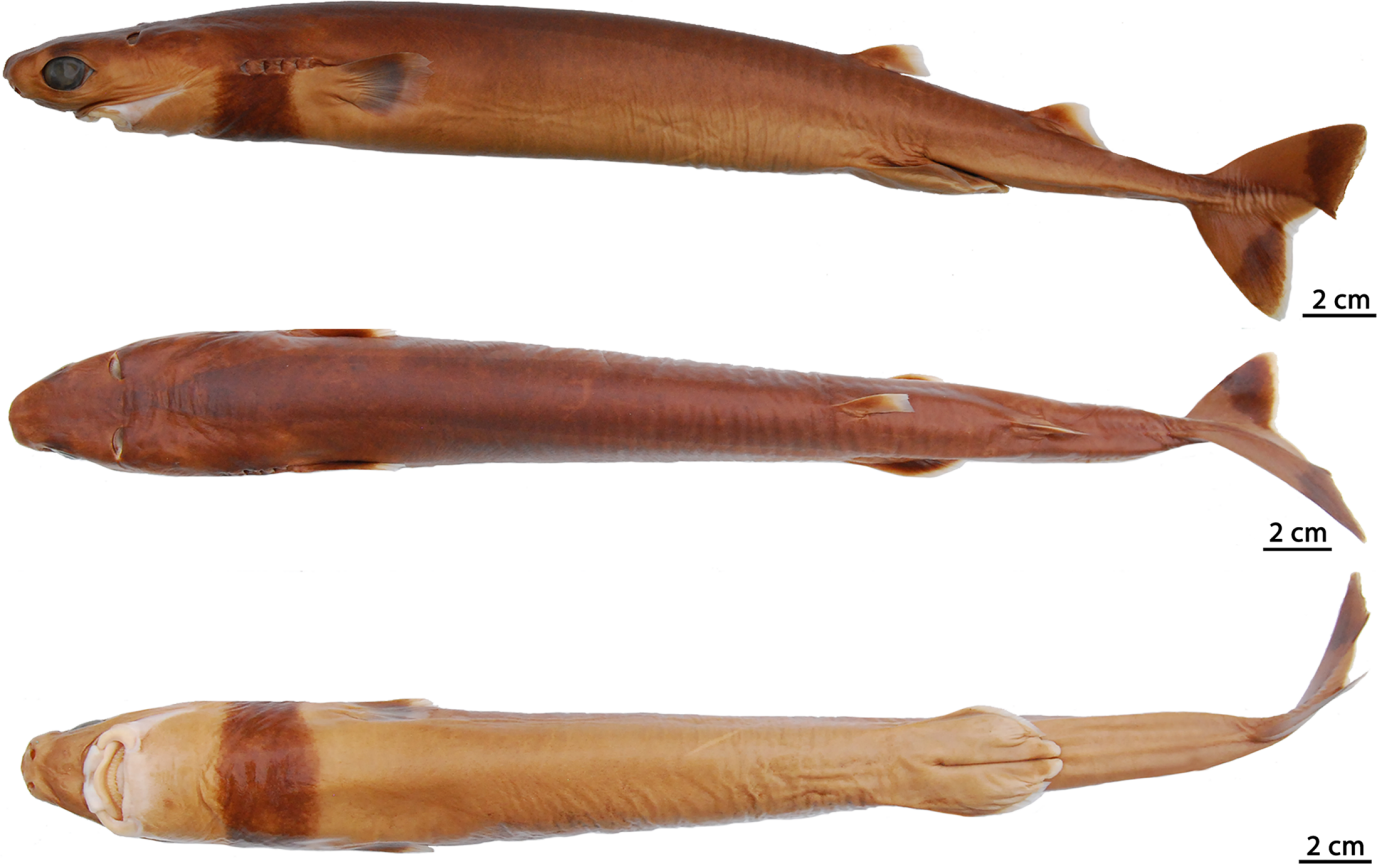
Adult male specimen of *Isistius brasiliensis* (HUMZ 124775). Lateral (top), dorsal (middle), and ventral (bottom) views; from the Northwestern Pacific Ocean, off Micronesia.

*Scymnus brasiliensis* Quoy & Gaimard (1824): 198 (original description, not figured; type locality: Brazil) [16]; Rochebrune (1883): 48 (Cape Verde) [60]; Garman (1899): 40 (historical account) [61]; Jüngersen (1899): 4 (mention of clasper) [46]; Miranda Ribeiro (1907): 205 (references) [62].
*Scymnus (Scymnus) brasiliensis torquatus* Vallenciennes [A.] in Müller & Henle (1839): 93 (original descriptions, Mauritius, Cape Verde at St. Jago, Rio de Janeiro) [30]; Duméril (1865): 453 (references, description, Mauritius) [63].
*Scymnus (Scymnus) brasiliensis unicolor* Vallenciennes [A.] in Müller & Henle (1839): 93 (original descriptions, Mauritius, Cape Verde at St. Jago, Rio de Janeiro) [30]; Duméril (1865): 453 (references, description, Mauritius) [63]
*Squalus (Scymnus) fulgens* Bennett (1840): 255 (description, luminescence, tropical Pacific near Christmas Island) [18]; Bennett (1860): 66 (luminescence, a second tropical Pacific specimen) [64]; Waite (1897): 196 (reference) [65].
*Dalatias brasiliensis* Gray (1851): 76 (description, Mauritius, St. Jago, Rio de Janeiro) [59].
*Scymnus torquatus*: Duméril (1861): 261 (name only, St. Jago) [66]; Waite (1897): 196 (reference) [65].
*Leius ferox* Kner (1864): 10, pl. 4, fig 2 (description, illustration, Australia) in [31]; Schmeltz (1866): 13 ("South Seas") [67]; Waite (1897): 196 (reference) [65]; Johann (1899): 152 (luminescence) [68]; Whitley (1940): 149 (description, illustration, luminosity, Australia) [69].
*Isistius brasiliensis*: Günther (1870): 429 (references, description, South Pacific and Gulf of Guinea) [70]; Bleeker (1874): 68 (Madagascar) [71]; Peters (1876): 853 (Indian Ocean) [72]; Rochebrune (1883): 48 (Cape Verde) [60]; Dean (1891): 513 (luminescence) [73]; Sauvage (1891): 5, 511 (specimen in Paris museum) [74]; Waite (1897): 194, 195 (Lord Howe Island, description) [65]; Garman (1899): 34, pl.1, fig 1, pi. 2–3, pl. 69, fig 2 (description, size, teeth, anatomy, Galapagos Islands) in [61]; Johann (1899): 152 (luminescence) [68]; Burckhardt (1900a): 559, 566, 568, fig 5 (luminescence, luminous organs) in [75]; Burckhardt (1900b): 488 (body shape) [76]; Waite (1900): 195, fig 1, 2 (teeth, largest recorded spec, Lord Howe Island) in [77]; Fatio & Spiess (1902): 534 (Burkardt’s presentation on the brain) [78]; Hebb (1903): 289 (Burkhardt’s description of brain) [79]; Waite (1904): 188 (references, Lord Howe Island) [80]; Leriche (1905): 95 (comparison with fossil teeth of *I*. *trituratus*) [81]; Mangold (1907): 583 (luminescence) [82]; Miranda Ribeiro (1907): 169, 205 (description, references, Brazil) [62]; Brauer (1908): 133, pl. 2 (luminescence) [83]; Regan (1908): 55 (classification, size) [84]; Burckhardt (1907): 26, figs 16–23 (description of brain, illustrated) in [85]; Giglioli (1912): 72, 109 (luminescence) [86]; Lydekker *et al*. (1912): 421 (light emission) [87]; Garman (1913): 238, 239 (references, description) [88]; Jordan *et al*. (1913): 23 (South Pacific, off Fiji, Brazil, Guinea, Japan) [89]; Lampe (1914): 214 (teeth, size, west of Sierra Leone) [90]; Metzelaar (1919): 191 (references) [91]; Miranda Ribeiro (1923): 26 (same as Miranda Ribeiro, 1907) [92]; Fowler (1926): 5 (in footnote) [93]; Whitley (1927): 3 (Fiji) [94]; Fowler (1928): 23 (description, references, Hawaii) [95]; Duncker & Mohr (1929): 84 (size, depth, Equatorial Pacific near New Guinea) [96]; Fowler (1930): 497 (distribution) [97]; Parr (1937): 1 (north of Bahamas) [98]; Beebe & Tee-Van (1941): 121 (references) [99]; Fowler (1941): 270 (references, description, distribution, luminescence) [100]; Tinker (1944): 28 (description, habitat, size) [101]; Bigelow and Schroeder (1945): 146, fig 55 (description, illustration, habits, range) in [102]; Bigelow & Schroeder (1948): 509–513 (description, references, range, illustrated) [21]; Grey (1956): 94 (depth) [103]; King & Ikehara (1956): 18, fig 2 (morphometrics, illustrated) in [104]; Bigelow & Schroeder (1957): 11, fig 14c, 109, 124 (morphology, luminescence, illustrated) in [105]; Backus (1960): 245 (record from Puerto Rico) [106]; McCormick *et al*. (1963): 357 (luminescence) [107]; Strasburg (1963): 33–38 (morphology, teeth, nursery, feeding, illustrated, records from around Marquesas Islands, Central Pacific) [26]; Garrick & Springer (1964): 681 (morphometrics) [15]; Hulley & Penrith (1966): 228 (differences from *Euprotomicroides*) [9]; Hubbs *et al*. (1967): 9, 14, 15, 20 (comparison with *Euprotomicrus bispinatus*, luminescence) [23]; Lewis (1969): 721 (stomach oils) [108]; Isouchi (1970) (Eastern Pacific) [109]; Jones (1971): 791 (predation on whales) [110]; Beardsley Jr. *et al*. (1972): 107 (predation on istiophorids) [111]; Bass *et al*. (1976): 44–46 (description, biology, South Africa) [112]; Compagno (1977): 308, 309 (jaw kinetics, morphology) [113]; Figueiredo (1977): 8, 9, fig 8 (in key, Brazil, illustrated) in [114]; Thomson & Simanek (1977): 248 (caudal region, illustrated) [115]; Bass (1978): 576 (Mozambique channel) [116]; Hodgson & Mathewson (1978): 576 (Mozambique channel) [117]; Seigel (1978): 602, 603, 605–610, 612 (teeth, anatomy, feeding, reproduction, comparison with *Squaliolus laticaudus* and *Euprotomicrus*, Atlantic, Indian, North and South Pacific oceans) [118]; Norris & Dohl (1980): 846 (predation) [119]; Cadenat & Blache (1981): 105, 106, 109, figs 80, 81 (in species key, description, morphology, morphometrics, dermal denticles illustrated) in [120]; Moss (1981): 28 (biting mechanism) [121]; Perrin *et al*. (1981): 594 (predation) [122]; Taylor *et al*. (1983): 110 (predation on *Megachasma pelagios*) [123]; Compagno (1984): 93, 94, 96, 108, 109, 228 (diagnosis, range, biology, predation on squids and *Megachasma pelagios*) [13]; Reif (1985): 112, 113, 115, 116 (photophores, denticles, illustrated) [124]; Le Beouf & McCosker (1987): 389 (predation on northern elephant seals) [125]; Wullimann (1987): table 1 (encephalon) [126]; Sadowsky *et al*. 1987: 660, 661 (predation, Brazil) [127]; Reddy & Griffith (1988): 11, 12, fig 2 (predation, llustrated) in [128]; Sadowsky *et al*. (1988): 919 (teeth) [129]; Stehmann & Krefft (1988): 24, 25 (luminescence, clasper) [130]; Herman *et al*. (1989): 102, 103, 122 (description of teeth), text plate 16 (teeth), pl. 18 (fossil teeth) in [131]; Fulton (1990): 124 (predation on seal) [132]; Nakano & Tabuchi (1990): 60–62 (distribution, biology, reprooduction) [133]; Wetherbee *et al*. (1990): 30 (predation on marine mammals) [134]; Würsig & Jefferson (1990): 46 (predation) [135]; Choy & Hiruki (1992): 3 (predation) [136]; Raschi & Tabit (1992): 129, 135 (photophores, morphology) [137]; Shirai (1992) (morphology) [3]; Shirai (1992): 516 (in material examined) [138]; Alcorn & Westlake (1993): 3 (predation) [139]; Anderson & Ahmed (1993): 54 (predation) [140]; Bres (1993): 135 (reference) [141]; Hiruki *et al*. (1993a): 464, figs 4, 8 (predation) in [142]; Hiruki *et al*. (1993): 470 (predation) [143]; Gadig (1994): 33, 43–45 (predation, in key, Brazil) [144]; Ito *et al*. (1994): 483, fig 2 (predation, illustrated, *Gasterochisma melampus*) in [145]; Jefferson & Leatherwood (1994): 2 (predation) [146]; Mullin *et al*. (1994): 467 (predation) [147]; Stacey *et al*. (1994): 1 (predation) [148]; Ellis & Shackley (1995): 162 (teeth) [149]; Zanelatto *et al*. (1995): 142 (predation) [150]; Gasparini & Sazima (1996): (predation) [151]; Mikhalev (1997): 18 (predation) [152]; Amorim *et al*. (1998): 623, 629 (Santos) [153]; Delgado-Estrella *et al*. (1998): 133 (predation) [154]; Gadig (1998): 50 (southeastern Brazil) [155]; MacLeod (1998): 72 (predation) [156]; McKinnell & Seki (1998): 131 (catches in North Pacific) [157]; Widder (1998): 267–272 (predation, morphology, bioluminescence) [27]; Mazzoleni & Schwingel (1999): 114, 115 (southeastern Brazil) [158]; Stehmann *et al*. (1999): 613 (distribution) [159]; Visser (1999): 518 (predation) [160]; Walker & Hanson (1999): 1324 (predation) [161]; Bozzano & Collin (2000): 192, 194, 197, 199, 204 (distribution, vision, northeastern Atlantic Ocean) [162]; Fish & Shannahan (2000): 1069 (biology) [163]; Gonzalez & Magenta-da-Cunha (2000) (differences in biting with *Squaliolus laticaudus*) [164]; Kiraly *et al*. (2000): 2, 8, 9 (biology, U.S.A.) [165]; Lucas & Hooker (2000): 49 (predationon beaked whales) hooker [166]; Addink & Smeenk (2001): 47 (predation) [167]; Baird *et al*. (2001): 989 (predation) [168]; Gadig (2001): 30, 84–86, 224, 232, 233, 239, 243, 244, 265 (in key, image, description, range, biology, Brazil) [169]; Heithaus (2001): 58, 59, 64 (predation on cetaceans) [170]; Mincarone *et al*. (2001): 124 (predation) [171]; Motta & Wilga (2001): 136 (feeding mechanism) [172]; Soto (2001): 66, 93 (in checklist, Brazil) [173]; Soto & Mincarone (2001): 23 (reference Brazil) [174]; Belcher & Lee Jr. (2002): 3 (predation) [175]; Cocke (2002): 117 (fossil teeth) [176]; Gadig & Gomes (2002): 1323, fig 1 (morphometrics, embryos) in [22]; Motta *et al*. (2002): 24 (predation) [177]; Pérez-Zayas *et al*. (2002): 309, 310 (predation, mouth and teeth illustrated) [178]; Shimada (2002): 70 (jaws) [179]; Azevedo *et al*. (2003): 412 (predation) [180]; Cavanagh *et al*. (2003): 35, 36, 168 (distribution, conservation) [181]; Hutchins *et al*. (2003): 139, 152, 154, 155 (predation on *Megachasma pelagios*, vertical migration, description, distribution, habitat and behavior, illustrated) [182]; Matott (2003): 6 (feeding mechanism) [183]; Moore *et al*. (2003): 388 (distribution, biogeography, predation on swordfishes) [184]; Silva-Jr & Sazima (2003): 1 (predation) [185]; Zidowitz (2003): 1433 (biology, comparison with *I*. *plutodus*) [186]; Benz & Bullard (2004): 395 (illustration) [187]; Haney *et al*. (2004): 410 (predation) [188]; Johnsen *et al* (2004): 1, 2 (photophores) [189]; Makino *et al*. (2004): 169, 170 (observed predation) [190]; Peach & Rouse (2004): 236 (pit organs) [191]; Sielfeld & Kawaguchi (2004): 80, 83 (listed) [192]; Smith *et al*. (2004): 134, fig 24. (illustrated) in [193]; Soto & Mincarone (2004): 7, 70 (listed) [194]; Abercrombie *et al*. (2005): 779 (molecular primers) [195]; Bacilieri (2005): 11 (captured by midwater trawl) [196]; Coello (2005): 34 (Ecuador) [197]; Compagno & Kyne (2005): 23 (distribution) [198]; Compagno *et al*. (2005): 127, pl. 14 (distribution, teeth, description, illustrated) [33]; Compagno *et al*. (2005): 55 (Philippines) [199]; Dean *et al*. (2005): 357 (predation) [200]; Frontier Madagascar (2005): appendix 5 (reference) [201]; Gonzalez (2005): appendix II (southeastern Brazil) [202]; Lamilla & Bustamante (2005): 9, 24, 60 (Chile) [203]; Musick & Ellis (2005): 59 (reproduction) [204]; Van Den Hoff *et al*. (2005): fig 2 (predation, illustrated) in [205]; Bertilsson-Friedman (2006): 362 (predation) [206]; Cunha & Gonzalez (2006): 466 (reproduction) [207]; George & Zidowitz (2006): 76 (reference) [208]; Kyne *et al*. (2006): 15 (conservation) [209]; Nelson (2006): 59 (predation on *Megachasma pelagios*) [210]; Priede *et al*. (2006): 1437 (vertical range) [211]; Sigler *et al*. (2006): 402 (predation) [212]; White *et al*. (2006): 62 (identification, distribution, habitat, biology, fisheries) [213]; Yearsley *et al*. (2006): 16 (common name: Smalltooth Cookiecutter Shark) [214]; Bossart *et al*. (2007): 235, 236 (predation on whales) [215]; Brownell Jr. *et al*. 2007: 3 (predation) [216]; Burdin *et al*. (2007): 8 (predation) [217]; Kyne & Simpfendorfer (2007): 30, 79 (distribution, embryos) [218]; Lisney & Collin (2007): 268 (biology, ecology) [219]; Mejía-Falla *et al*. (2007): 127, 135 (literature Pacific Ocean) [220]; Nunan & Senna (2007): 163, 164 (1997, continental slope, Brazil) [221]; Ohishi *et al*. (2007): 628 (predation) [222]; Ramsay & Wilga (2007): 679, 680 (predation) [223]; Silva-Jr. *et al*. (2007): 507, 508, 509 (predation) [224]; Souto *et al*. (2007): 22 (predation) [225]; Anderson & LaBarbera (2008): 3625 (teeth) [226]; Babcock & Nakano (2008): 473 (reference) [227]; Bermúdez Villapol *et al*. (2008): 158, fig 6 (predation on whale) in [228]; Bunkley-Williams *et al*. (2008): 264 (predation on turtles) [229]; Compagno (2008): 19 (pelagic diversity) [230]; Fergusson *et al*. (2008): 221 (predation on *Odontaspis ferox*) [231]; Greenberg (2008): 7 (predation) [232]; Hazin *et al*. (2008): 214, 215 (Brazil) [233]; Jucá-Queiroz *et al*. (2008): 78 (northeastern Brazil) [234]; Mandelman *et al*. (2008): 435 (predation) [235]; Mannering & Hiller (2008): 1351 (teeth compared to *Centroselachus crepidater*) [236]; Meneses (2008): 38, fig 5 (feeding behavior, illustrated) in [237]; Petersen *et al*. (2008): 63, 64 (in bycatch) [238]; Renner & Bell (2008): 102 (predation) [239]; Van Waerebeek *et al*. (2008): 6 (predation) [240]; Weir *et al*. (2008): 1227 (reference) [241]; Bornatowski *et al*. (2009): 2 (southern Brazil) [242]; Camhi *et al*. (2009): 9, 43, 75 (pelagic) [243]; Claes & Mallefet (2009): 3684 (luminescence) [244]; Goto *et al*. (2009): 1 (distribution, depth) [245]; Lowry *et al*. (2009): 2484 (predation) [246]; Santos & Gadig (2009): 4 (predation on cetaceans and *Carcharodon carcharias*) [247]; Souto *et al*. (2009): 1 (interactions with seals) [248]; Velozo *et al*. (2009): 3 (predation) [249]; Aguiar & Valentin (2010): 568 (predation) [250]; Claes *et al*. (2010): 28 (luminescence) [251]; Deakos *et al*. (2010): 122 (predation) [252]; Gomes *et al*. (2010): 41, 59 (description, wounds on *Megachasma pelagios*) [253]; Largacha *et al*. (2010): 1–4 (description, morphometrics, weight) [254]; Mendonça *et al*. (2010): 32 (reference) [255]; Menezes (2010): table 1 (listed, Brazil) [256]; Papastamatiou *et al*. (2010): 362 (occurrence) [257]; Sáez & Pequeño (2010): 479, 480 (in key, teeth) [258]; Sáez *et al*. (2010): 622, 626, 633 (in key, coloration, Chille) [259]; Straube *et al*. (2010): 909, 914, fig 3 (molecular phylogeny) in [260]; White & Dharmadi (2010): 1365 (predation) [261]; Castro (2011): 95, 140, 141, 145–150 (identification, in key, luminescent organs compared to *Centroscyllium fabricii*, range, biology, relation to humans, dermal denticles, illustrated) [262]; Claes *et al*. (2011): 4 (luminescence) [263]; Dwyer & Visser (2011): 111–113, 130 (predation, New Zealand) [264]; Claes *et al*. (2012): 1691 (predation) [265]; Gardiner *et al*. (2012): 358 (feeding) [266]; Heithaus & Vaudo (2012): 519 (feeding) [267]; Hoyos-Padilla *et al*. (2012): 2, 4 (feeding, migration, morphology, Eastern Pacific) [268]; Kyne *et al*. (2012): 40, 142 (conservation, North and Central America, and Caribbean) [269]; Motta & Huber (2012): 155, 160, 169, 191 fig 6.14 (bioluminescence, feeding, teeth, illustrated) in [270]; Naylor *et al*. (2012): 65, 128, 147 (molecular data, classification) [271]; Naylor *et al*. (2012): 42 (molecular phylogenetic relationships) [272]; Wenzel & Suárez (2012) (Cape Verde) [273]; Wetherbee *et al*. (2012): 241 (unusual tooth and jaw morphology) [274]; Ebert *et al*. (2013): 166, 167, 170 (destribuion, description, behavior, illustrated) [1]; Ebert *et al*. (2013): 298 (Taiwan) [275]; Ebert *et al*. (2013): 10 (Taiwan) [276]; Ebert & Stehmann (2013): 132, 133, 135 (comparison to *I*. *plutodus*, behavior, Eastern and Central-Estern Atlantic) [277]; Iglésias (2013): 26, 81 (in key) [278]; White & Last (2013): 236, 237, 243 (as senior synonym of *I*. *labialis*) [279]; Bustamante *et al*. (2014): 1622 (Chile) [280]; Carrillo-Briceño *et al*. (2014): 11 (fossils) [281]; Claes *et al*. (2014): 1, fig 4 (photophores, predation) in [4]; Helfman & Burgess (2014): 27 (teeth) [282]; Munroe *et al*. (2014): 319 (feeding) [283]; Rosa & Gadig (2014): 90, 93, 97 (Brazil) [284]; da Silva & de Carvalho (2015): 3, 6, 11, 36 (pectoral fin anatomy) [43]; da Silva *et al*. (2015): 876, 880 (pectoral fin anatomy) [285]; Dyldin (2015): 54 (listed, Russia) [286]; Ebert *et al*. (2015) (range extension, to eastern North Pacific) [287]; Stehmann & Kukuev (2015): 73 (references to *I*. *plutodus*), 75, 77 (caudal fin, distribution) [34]; Moyer & Bemis (2016): 535 (teeth replacement) [288]; Nakaya *et al*. (2016): 6 (feeding) [289]; Nelson *et al*. (2016): 67 (predation on megamouth sharks) [290]; Weigmann (2016): 892 (listed) [291]; da Silva *et al*. (2017): 3, fig 13 (scapular morphology) in [292], Perez & Marks (2017): 140, 144, 145, 146 (fossil teeth, Florida, feeding) [293].
*Iristius ferox*: Schmeltz (1866): 10 ("South Seas") [67].
*Isistius braziliensis*: Macleay (1881): 368 (reference, Australia) [294]; Morris *et al*. (1983): 296 (composition of liver) [295]; Williams (2001): 214 (teeth) [296]; Sielfeld & Kawaguchi (2004): 85 (distribution, biology) [192].
*Isistius marmoratus* Rochebrune (1885): 98 (description, Senegambia) [32].
*Leius brasiliensis*: Günther (1909): 490–1 (references, description) [50].
*Scymnus fulgens*: Giglioli (1912): 72, 109 (luminescence) [86].
*Isistius* sp. [probably *I*. *brasiliensis*]: Daniel (1934): 266 (morphology of eyes compared to *Squatina*) [297]; White (1937): 70 (teeth) [298]; Moss (1977): 359 (teeth) [299]; Maisey (1983): 50 (teeth) [300]; Johnson & Wolman (1984): 35 (predation) [301]; Bonde & O’Shea (1989): 448 (predation) [302]; Clark & Kristof (1990): 278 (feeding habits, similar to *Dalatias*) [303]; Compagno (1990): 45, 63 (predation) [304]; Foster & Hare (1990): 50 (predation) [305]; Shirai & Nakaya (1990): 351 (morphology) [306]; Miyake *et al*. (1992): 286 (morphology) [307]; de Carvalho (1996): 41 (morphology) [308]; Laurito (1996): 87, 88, 91 (tooth fossils, Upper Paleocone, Eocene, Costa Rica) [309]; Shirai (1996): 16, 24, 33 (morphology, phylogeny) [310]; Jefferson & Barros (1997): 3 (High Guayacán) [311]; Zerbini & Santos (1997): 105 (predation) [312]; Peach & Marshall (2000): 1133 (morphology) [313]; Wardle *et al*. (2000): 640 (predation) [314]; Adnet & Cappetta (2001): 242, 247, 248 (fossil record) [2]; Best (2001): 281 (predation), 288 (predation among oceans) [315]; Pitman *et al*. (2001): 498 (predation) [316]; Soto (2001): 32 (feeding similar to *Centroscymnus coelolepis*) [317]; Dalebout *et al*. (2002): 599, 604 (predation, distribution) [318]; Jefferson & Curry (2003): 2 (predation) [319]; Kriwet (2003): 588 (teeth) [320]; Dalebout *et al*. (2004): 350 (predation) [321]; Kriwet & Benton (2004): 188 (fossil record) [322]; Wilga & Lauder (2004): 142 (morphology) [323]; Borsa & Robineau (2005): 467 (predation) [324]; Meneses *et al*. (2005): 80, 82 (predation, northeastern Brazil) [325]; Souza *et al*. (2005): 132, 134 (predation) [326]; Claridge (2006): 6, 26 (predation) [327]; Fitzpatrick *et al*. (2006): 397 (predation on *Rhincodon typus*) [328]; Kocsis (2007): 30 (fossils, Upper Paleocene, France, South and North America) [329]; McSweeney *et al*. (2007): 688 (predation) [330]; Baird *et al*. (2008): 11 (predation, diel vertical migrations) [331]; Steiner *et al*. (2008): 3 (predation) [332]; Neto *et al*. (2008): 4, fig 3 (predation, illustrated) in [333]; Araújo *et al*. (2010): 4 (predation) [334]; Klug & Kriwet (2010): 335, 337, 338 (phylogeny, stratigraphy) [335]; Lane (2010): 25 (morphology) [336]; Vialle *et al*. (2011): 243 (record, France) [337]; Iserbyt & De Schutter (2012): 150 (distribution) [338]; Dolce & Wilga (2013): 86, 97, 98, append. 1–4 (morphology, gill slits, morphometrics) [339]; Maisey *et al*. (2014): 590 (tooth replacement) [340].
*Isistius labialis* Meng, Zhu & Li (1985): 442 (original description, illustration) [17]; Compagno *et al*. (2005): 128, pl. 14 (distribution, description, illustrated, compilation) [33]; Kyne & Simpfendorfer (2007): 30, 79 (South China Sea, compilation) [218]; Compagno (2008): 19 (pelagic diversity) [230]; Camhi *et al*. (2009): 19, 44, 75 (conservation) [243]; Klug & Kriwet (2010): fig 1 (illustration) in [335]; Dwyer & Visser (2011): 111, 112 (South China Sea) [264]; Claes *et al*. (2012): 1691 (compilation) [265]; Ebert & Stehmann (2013): 133 (reference) [277]; Ebert *et al*. (2013): 166, 167, 171 (distribuion, description, behavior, illustrated, compilation) [1]; White & Last (2013): 236, 237, figs 10, 11 (in synonymy of *I*. *brasiliensis*) in [279]; Dyldin (2015): 54 (in synonym of *I*. *brasiliensis*) [286]; Weigmann (2016): 892 (listed as valid) [291].
*Holotype*: MNHN A-7787, female 172 mm TL, Western Atlantic, Brazil, Expédition D. de Freycinet 1817–1820.

#### Diagnosis

A species of *Isistius* differentiated from its only congener, *I*. *plutodus*, by the following characters: snout more rounded and proportionally longer (*vs*. snout shorter and less rounded in *I*. *plutodus*); preorbital (15.50% HDL *vs*. 12.01% HDL in *I*. *plutodus*), prenasal (6.27% HDL *vs*. 4.79% HDL), preoral (32.46% HDL *vs*. 23.15%), and interorbital (25.48% HDL *vs*. 17.27% HDL) lengths (relative to head length) proportionately greater than in *I*. *plutodus*; head length 2.25 times interdorsal space (*vs*. 3.3); second dorsal fin almost same height as first (*vs*. second dorsal fin higher than first in *I*. *plutodus*); lower symphyseal tooth smaller (its height 1.41% TL *vs*. 2.08% TL), and tooth base more slender (0.62% TL *vs*. 1.08% TL); lower symphyseal tooth 5% shorter than adjacent teeth in *I*. *brasiliensis*, whereas they are the same height in *I*. *plutodus*; relatively more tooth rows, with tooth formula for upper/lower teeth 15+15/12+1+13 (*vs*.12+12/9+1+9); lower teeth proportionally smaller, only 3 times greater than upper teeth (*vs*. 6 times in *I*. *plutodus*). Oral integument with concave anterior margin and relatively narrow in *I*. *brasiliensis* (with straight anterior margin and more broad in *I*. *plutodus*); upper postventral margin of caudal fin smaller than lower postventral margin (*vs*. upper margin greater than lower margin in *I*. *plutodus*); general body color in *I*. *brasiliensis* lighter than in *I*. *plutodus*; ventral dark collar proportionately smaller in *I*. *brasiliensis* (posterior end at level of pectoral fin origin) than in *I*. *plutodus* (posterior end at level of pectoral fin insertion). Morphology and proportions of neurocranium also differs between both species (Table 3): neurocranium in *I*. *brasiliensis* has greater nasobasal length (56.08% HDL *vs*. 54.17% HDL), longer otic capsule (18.05% HDL *vs*. 15.87% HDL), and greater width across postorbital processes (27.74% HDL *vs*. 22.49% HDL) even though the postorbital process itself is smaller in *I*. *brasiliensis* than in *I*. *plutodus* (4.81% HDL *vs*. 6.13% HDL).

**Table 3:** Neurocranial measurements.

Measurements	*I*. *brasiliensis* (MNHN 1996–0565)				*I*. *plutodus* (ZUEC 8333)			
	mm	%TL	%HDL	%NB	mm	%TL	%HDL	%NB
Nasobasal length	46.5	11.37	56.08	46.5	36.69	11.02	54.17	36.69
Width across nasal capsules	18.09	4.42	21.82	38.90	13.82	4.15	20.40	37.67
Width of nasal capsule	8.5	2.08	10.25	18.28	7.95	2.39	11.74	21.67
Length of nasal capsule	10.32	2.52	12.45	22.19	8.48	2.55	12.52	23.11
Width of nasal aperture	6.36	1.56	7.67	13.68	5.36	1.61	7.91	14.61
Distance between nasal apertures	4.82	1.18	5.81	10.37	4.69	1.41	6.92	12.78
Distance from dorsal edge of anterior fontanelle to base of medial rostral cartilage	6.55	1.60	7.90	14.09	5.66	1.70	8.36	15.43
Width of anterior fontanelle	2.12	0.52	2.56	4.56	1.67	0.50	2.47	4.55
Width of basal plate at orbital notches	2.52	0.62	3.04	5.42	1.59	0.48	2.35	4.33
Length of orbit	22.1	5.40	26.65	47.53	17.31	5.20	25.56	47.18
Length of postorbital process	3.99	0.98	4.81	8.58	4.15	1.25	6.13	11.31
Length of otic capsule	14.97	3.66	18.05	32.19	10.75	3.23	15.87	29.30
Width across suborbital shelves	18.98	4.64	22.89	40.82	14.82	4.45	21.88	40.39
Width across otic capsules	13.23	3.23	15.96	28.45	9.12	2.74	13.47	24.86
Width across postorbital processes	23	5.62	27.74	49.46	15.23	4.57	22.49	41.51

Following Compagno [41]. Specimens of *Isistius brasiliensis* (MNHN 1996–0465, 409 mm TL, 82.92 mm HDL) and *Isistius plutodus* (ZUEC 8333, 333 mm TL, 67.73 mm HDL). (NB: nasobasal length).

#### Description

Morphometric data presented in Table 2, tooth counts in Table 4, and vertebral counts in Table 5. Measurements of specimens from Atlantic, Pacific, and Indian Oceans were compared with each other, but no differences between specimens were found.

**Table 4:** Tooth count.

		Min	Max	Mean	SD	n	Formula
***I*. *brasiliensis***	upper	17	39	31.27	4.23	56	16+15
	lower	15	31	26.39	3.67	83	12+1+13
***I*. *plutodus***	upper	19	28	24.4	3.58	5	13+12
	lower	17	19	18.5	1	4	9+1+9

Upper and lower tooth count, and hypothetical tooth formula, of specimens of *Isistius brasiliensis* and *I*. *plutodus*.

**Table 5:** Vertebral counts of dalatiids.

	*I*. *brasiliensis*	*I*. *plutodus*	*D*. *licha*	*S*. *laticaudus*	*S*. *aliae*	*E*. *bispinatus*	*H*. *marleyi*
**Monospondylous**	37, 39, 40, 41, 42, 43, 44	43, 44, 45	36, 37, 41	28, 29, 30, 31, 32	29	31, 32, 33	33
**Precaudal Diplospondylous**	18, 19, 20, 21, 22, 23, 24	23, 24, 26	10, 11, 12	15, 16, 18	17	16, 17, 18, 19, 21	18
**Postcaudal Diplospondylous**	17, 18, 19, 20, 21, 22, 23	22, 26, 27	27, 28	12, 13, 14, 15	13	14	15

Vertebral counts of female and male specimens of *Isistius brasiliensis*, *I*. *plutodus*, *Dalatias licha*, *Squaliolus laticaudus*, *S*. *aliae*, *Euprotomicrus bispinatus* and *Heteroscymnoides marleyi*. Counts separated by monospondylous, precaudal, and postcaudal diplospondylous vertebrae.

Morphometric data of males and females were treated separately, but no differences associated with sex were found, except pelvic length (means of 9.56% TL *vs*. 7.97% TL in males and females, respectively). Differences associated with growth were also evaluated between immature and mature females (adults at 39 cm) and males (adults at 36 cm). In females, the measurements that have the most allometric differences between immature and mature specimens are PP1, HDL, PG1, PSP, POB, PRN, EYL, DCS, PPS, PCA, and CFL. In males, these measurements are PP2, PG1, POR, EYL, EYH, CLO, and CLI (see Table 1 for explanation of measurements).

Monospondylous vertebral counts 37–44, precaudal diplospondylous 18–24, and postcaudal diplospondylous 17–23. The location of the transition from mono- to diplospondylous vertebrae is observed in radiographs between the dorsal fins.

**Fig 4:**
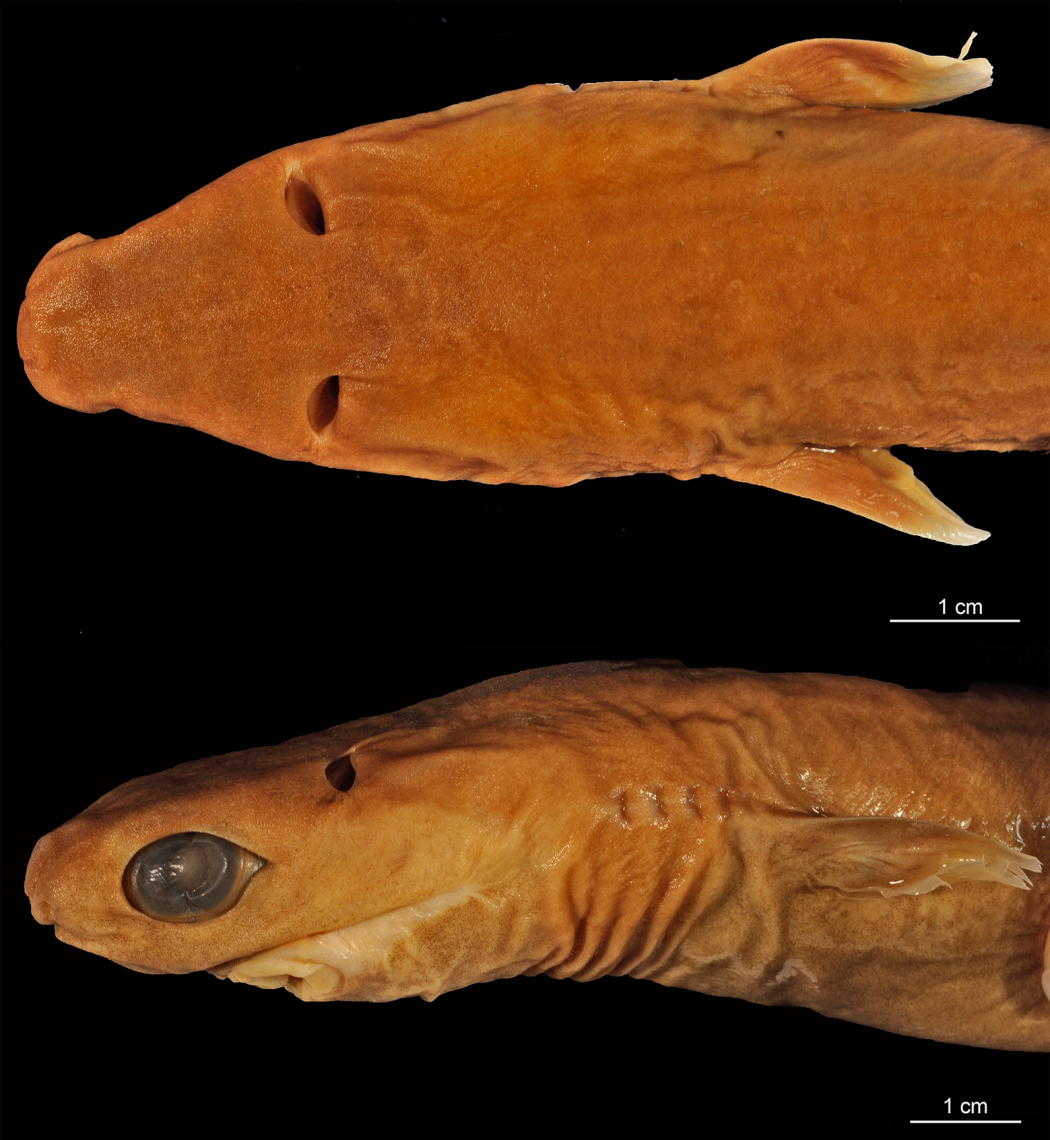
Head of a specimen of *Isistius brasiliensis* (MCZ 41352). Dorsal (top) and left lateral (bottom) views; from Central Atlantic Ocean.

*External morphology*. Trunk very slender and cylindrical, cigar shaped, and relatively straight but tapering anterior to first dorsal fin and toward pelvic and caudal fin origins. Trunk greatest height at about one-third its length. Lateral outline of head subrectangular, but slightly triangular anterior to mouth. Dorsal and ventral head profiles somewhat parallel, but dorsal profile slightly sloping anteriorly toward bulbous, rounded snout tip. Preorbital length relatively short, 6.4 times in head length; interorbital space 1.5 times eye length. Eyes large (18% HDL), somewhat tear-drop shaped, anteriorly rounded but slightly more slender posteriorly. Prespiracular length 4.15 times spiracle length. Spiracles very large (2% TL), conspicuously dorsally positioned posterior to eyes, and markedly oval, their greatest width transverse to body axis. Gill slits very small (1–0.8% TL); first gill slit greater than fifth, well behind eye; fifth gill slit just anterior to pectoral fin origin (Fig 4).

**Fig 5:**
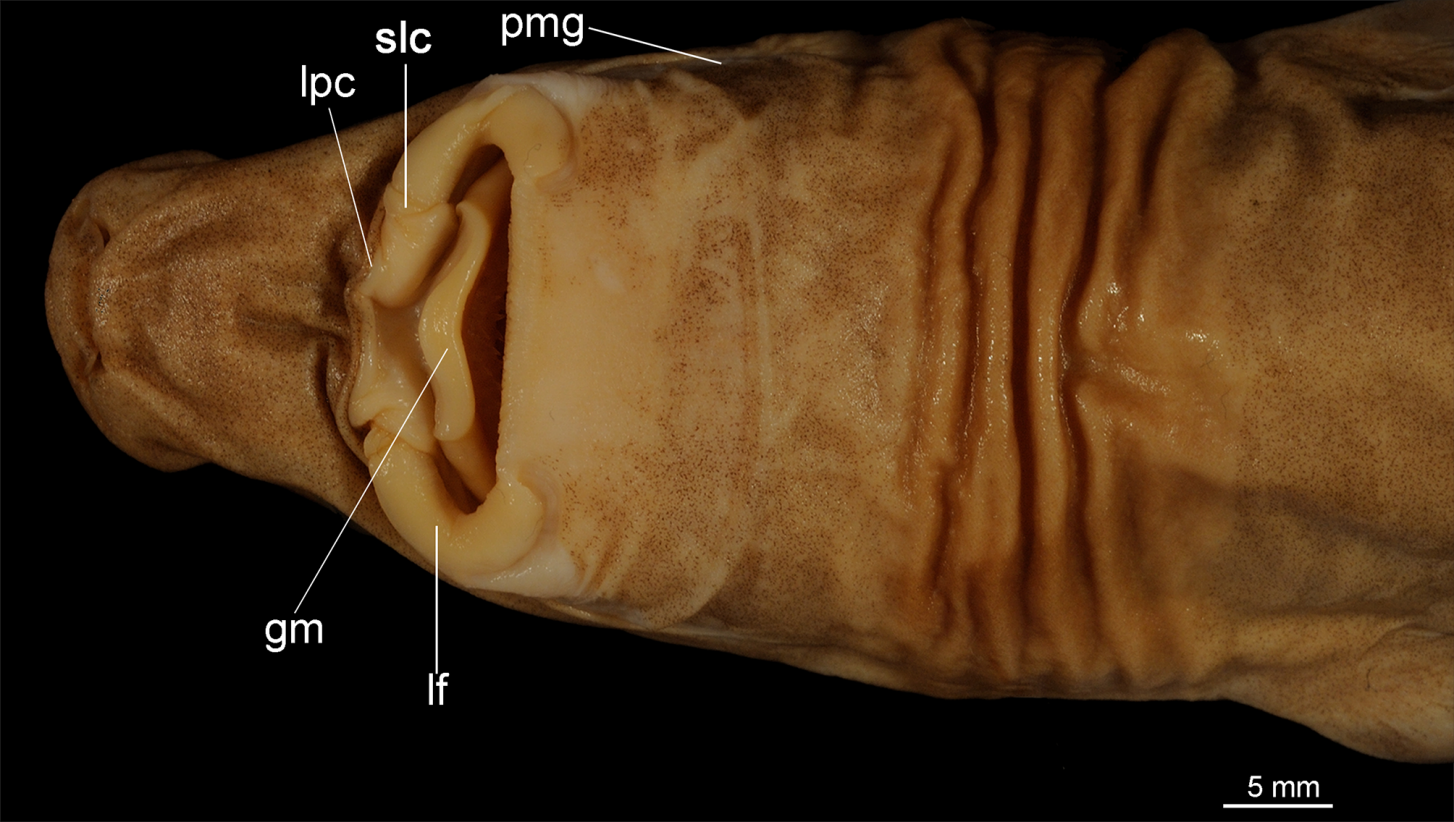
Ventral view of mouth region of *Isistius brasiliensis* (MCZ 41352). Abbreviations: **gm**, gum; **lf**, lip fold; **lpc**, upper lip cover; **pmg**, posterior mouth groove; **slc**, fleshy sack for the upper labial cartilages.

Nostrils anteroventrally positioned on snout; anterior portion of nostrils rounded, with a nasal flap separating anterior and posterior apertures; posterior portion of nostril elongated and slightly directed toward midline, lacking barbels. Internarial distance almost equal to prenarial length. Mouth very wide (7.2% TL), transverse, with lateral skin folds and deep grooves (25% HDL). Lower lip thick and slightly vertically striated. Upper lip cover (**lpc**) contiguous with lower ventral surface of head anterior to mouth, and laterointernally attached to thick and naked lip fold (**lf**) (Fig 5). Lip fold is a short tissue at corner of mouth, posteriorly directed and slightly turned toward medial region of lower jaw; lip fold rounded and visible when mouth closed; concealed by upper lip cover. Thick and wavy gum (**gm**) immediately dorsal to both upper lip cover and fleshy sack for upper labial cartilages (**slc**). Deep groove dorsal to lip fold extending from anterior to labial cartilages to almost posterior margin of lower jaw. Posterior mouth groove (**pmg**) extends from corner of mouth posteriorly to almost ventral collar; deep oral pocket present at mouth corner, immediately dorsal to lip fold. Preoral pouch anterior to mouth corner and dorsal to joint of upper anterior and lower labial cartilages [14].

Pectoral fins originate immediately posterior to fifth gill slit. Pectoral fins with straight posterior margins; pectoral fin length fits 7.5 times in predorsal body length, 2.6 times in head length. Anterior pectoral margins relatively straight and almost parallel to body axis, and posterior margins almost perpendicular to body, with corners slightly rounded. Inner pectoral margins slightly oblique and directed ventrally at their most posterior portion. Pectoral base 3.4 times height of first gill slit. Dorsal fins without spines. First dorsal very small and obliquely inclined rearward; its origin slightly anterior to pelvic fin origin but very posterior to posterior tip of pectorals. Origin of first dorsal about two-thirds of precaudal body length. First dorsal base length 1.7 times its height, and about 15% of head length. Posterior edge straight and free lower tip forms an acute angle. Second dorsal fin slightly taller and longer than first. Both its base length and height 1.15 times those of first dorsal fin. Origin somewhat above pelvic fin free rear tip. Base of second dorsal 1.7 times its height and about 17% of head length. Posterior margin of second dorsal similar to first dorsal, although its free rear tip has a prolongued filament.

Interspace between second dorsal and caudal fins about 1.13 times greater than interdorsal distance. Caudal fin length about 5.5 times predorsal length. Caudal peduncle without lateral ridges and rounded in cross-section, with height and width about equal (approximately 1.9% TL). Caudal fin asymmetrical with end of vertebral column somewhat upturned. Dorsal margin 1.4 times preventral margin, the latter originating slightly anterior to the former. Terminal margin of dorsal caudal lobe almost straight and perpendicular to body axis, but with slight inclination; end of dorsal margin posterior to end of subterminal margin. Subterminal notch present at posterior end of vertebral column; subterminal margin parallel to body axis. Upper postventral margin almost one-half size of lower postventral margin. Pelvic fin originates posterior to insertion of first dorsal fin, but anterior to its free rear tip. Pelvic-fin base almost three times first dorsal base length. Female pelvic fins with a smooth lateral angle and almost straight rear tip; in males, lateral angle smaller, and pelvic fin shorter, with claspers in adults slightly longer than end of fin. In ventral view, most distal portion of clasper white, somewhat triangular in outline (straight internal portion and oblique outer side directed toward midline). In dorsal view, clasper groove originates anteriorly almost at dorsal insertion of pelvic fin; groove somewhat straight, entirely dorsal, and terminating at gland. Claspers of juveniles not reaching rear tip of pelvic fin.

#### Teeth

**Fig 6:**
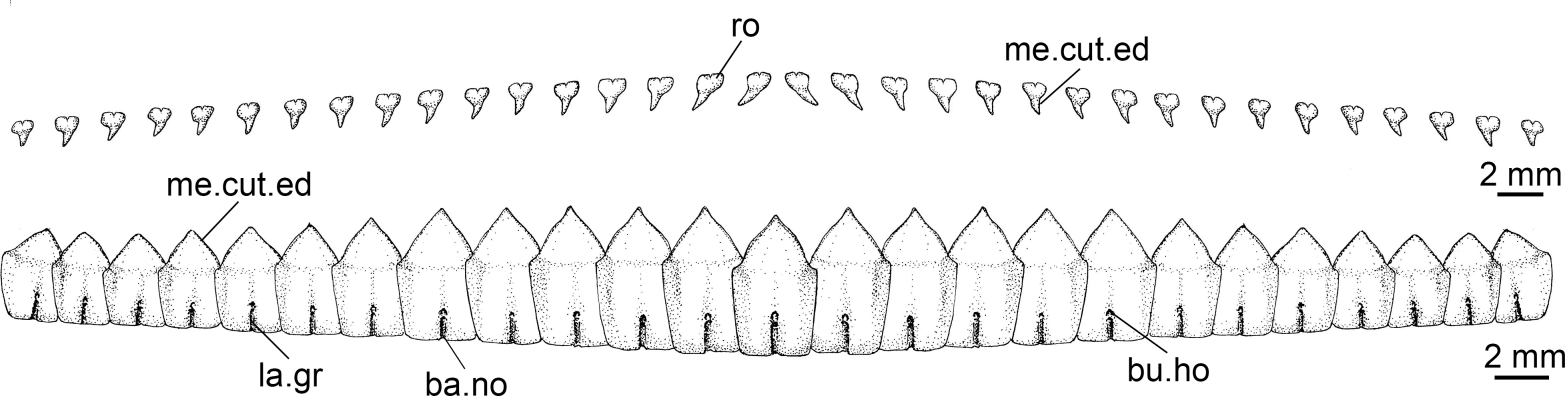
Upper and lower teeth of *Isistius brasiliensis* (MNHN 1996–0465) in labial view. Top. Upper teeth; bottom. Lower teeth. Abbreviations: **ba.no**, basal notch; **bu.ho**, button hole; **la.gr**, labial groove; **me.cut.ed**, mesial cutting edge; **ro**, root.

**Fig 7:**
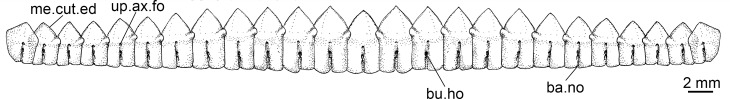
Lower teeth of *Isistius brasiliensis* (MNHN 1996–0465) in lingual view. Abbreviations: **ba.no**, basal notch; **bu.ho**, button hole; **me.cut.ed**, mesial cutting edge; **up.ax.fo**, upper axial foramen.

Dignathic heterodonty present, with palatoquadrate and mandibular teeth unicuspid, and smooth mesial cutting edges (**me.cut.ed**). On average, 31 (16+15) upper teeth with no teeth at palatoquadrate symphysis, but two parasymphyseal directed toward opposite sides. Upper teeth narrow, sharp, slightly distally inclined, not overlapping, usually in three rows, and multiserial in function. Upper teeth roots (**ro**) trapezoidal, with uppermost portion larger than root-crown junction; root pseudolobated with a sulcus dividing it into two regions (Fig 6); presence of a subtle axial foramen (**ax.fo**) below the sulcus on lingual side of crown. Upper teeth have a gradient of inclination, becoming more oblique toward posterior region of mouth. Lower teeth 26 (12+1+13) on average, with a tooth at the mandibular symphysis present between both upper parasymphyseal teeth. Lower symphyseal tooth slightly shorter than adjacent parasymphyseal teeth. Lower teeth with vertical triangular cusp and flat root in only one functional row and more than three non-funcional rows, with teeth facing the opposite side, at the internal part of mandibular cartilage. Starting at the symphysis, roots of lower teeth overlap toward opposite sides, with mesial portion behind adjacent tooth and distal portion over the following tooth. These interlockings leave depressions on the margins of lower teeth. Teeth form a single functional series, with each root completely exposed on outer face, and inner side closely attached to lower jaw. Labial groove (**la.gr**) present on labial side from the button hole (**bu.ho**) to basal notch (**ba.no**), and this lower indentation at the lowest part of root gives it a somewhat rounded bilobed aspect; apron (**a**) covering crown and upper part of root until half labial groove height; a small lower axial foramen (**lo.ax.fo**) above labial groove; (Fig 6). Lingual side of lower teeth with an upper axial foramen (**up.ax.fo**) at the uppermost part of the root; apron (**a**) covering the lingual portion of crown and upper region of root; button hole (**bu.ho**) also visible in lingual view (Fig 7). Lower teeth greater than upper teeth (symphyseal lower tooth 5.42 cm, and parasymphyseal upper tooth 1.70 cm in an adult specimen). Teeth in lateroposterior position in lower row shorter than those at symphysis (commissural lower tooth 3.72 cm, and upper tooth 0.88 cm in an adult specimen), and crown of commissural teeth directed toward anterior teeth. Lower teeth proportionally three times larger than upper teeth.

#### Coloration

**Fig 8:**
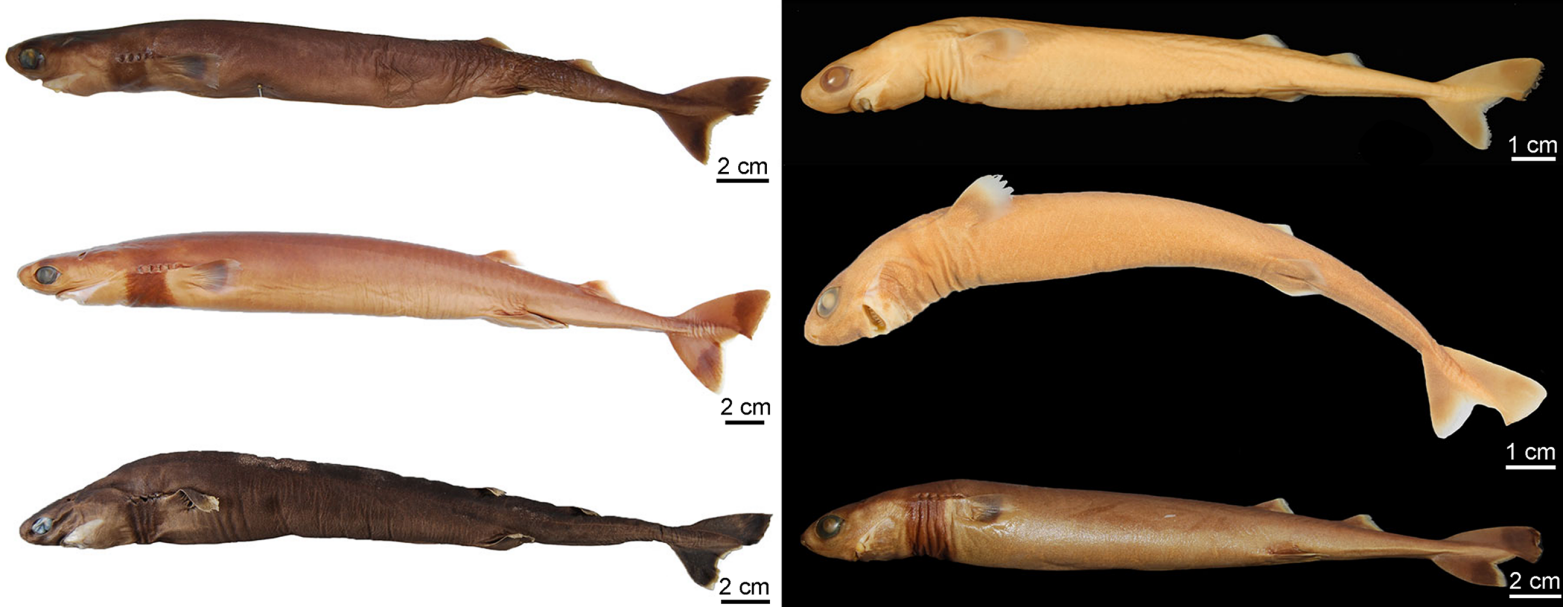
Preserved specimens of *Isistius brasiliensis* showing diversity of colors, shapes, and sizes. (A) CSIRO 3722 (Southwestern Pacific), (B) MCZ 58095 (Northwestern Atlantic), (C) HUMZ 124775 (Northwestern Pacific), (D) UF 165691 (Northeastern Atlantic), (E) NMW 60844 (Southwestern Indian), and (F) ZMH 10836 (Southeastern Atlantic).

Body light to dark brown with dorsal side slightly darker than ventral side. Darker brown ventral collar around branchial region posterior to mouth and extending to origin of pectoral fins. Color of collar the same as that of dorsal side, and easily distinguished from body ventrally. All fins brown with distal slender white margins. Both dorsal and ventral posterior margins of caudal fin also with a darker brown area, absent on subterminal margin. (Fig 8). Specimens varied according to intensity of coloration and presence of dark collar, which may possibly be due to fixation methods and length of time of preservation.

#### Dermal denticles

**Fig 9:**
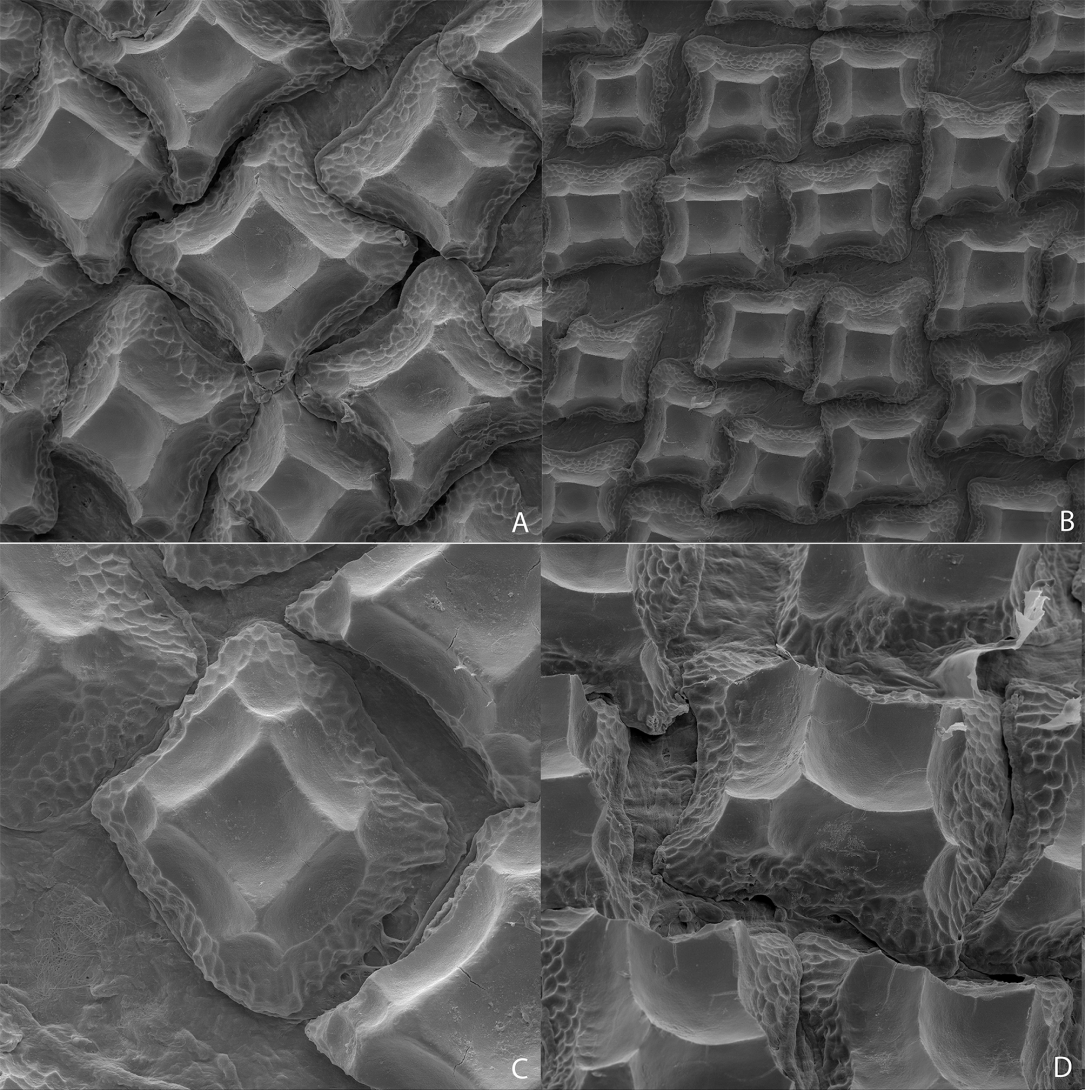
Dermal denticles of *Isistius brasiliensis* from the Pacific Ocean. (A) BPBM 24471, ventral region pre-collar, magnification 200x; (B) BPBM 24471, ventral region post-collar, magnification 100x; (C) HUMZ 211104, ventral region post-collar, magnification 290x; (D) BPBM 24471, ventral region post-collar, magnification 290x.

Dermal denticles without regular alignment, closely packed together, with small areas of skin in between where photophores can be found. Denticles small, very low, with no distinction between basal plate and pedicel (Fig 9). No acute medial cusp and medial ridges on crown. Denticles square-shaped but occasionally polygonal at base and trapezoidal apically, almost symmetrical. Base and crown with concave surfaces, and some concave indentations at the base. No variation of size and shape throughout body.

#### Luminescent markings

**Fig 10:**
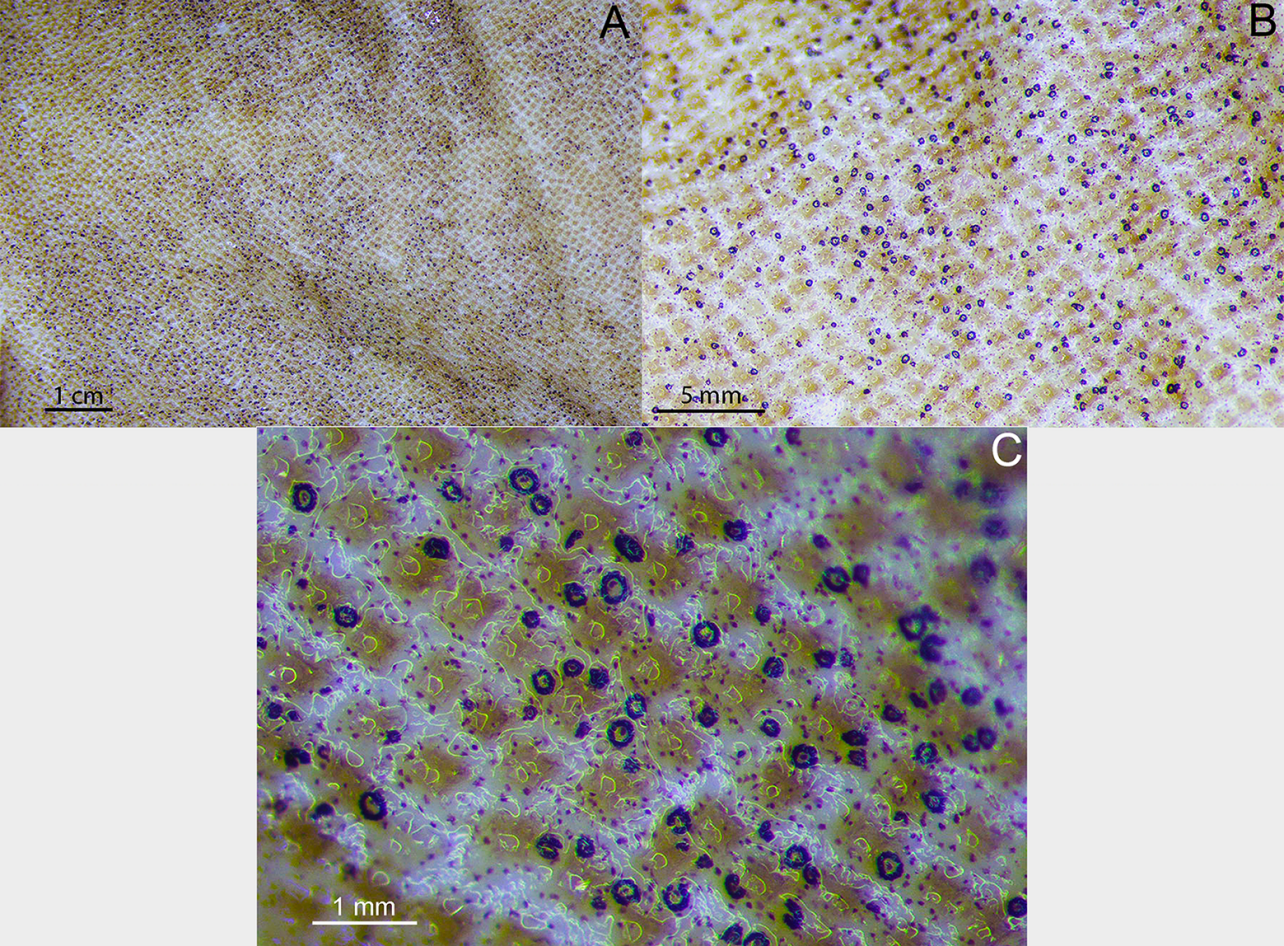
Ventral side of *Isistius brasiliensis* (BPBM 24471), post-collar. Progressively greater magnification from (A) to (C). Photophores (highlighted in purple) are irregularly distributed on top of and lateral to dermal denticles.

In fixed specimens, photophores black, small, with annular elements approximately 200 μm in size (Fig 10). Photophores usually present on ventral side of body, from tip of snout to posterior end of vertebral column, including lips, ventrolaterally to eyes, ventral fins, and claspers in males (except their white tip). No photophores present on ventral dark collar. Photophores present on dorsolateral side of head, lateral aspect of pectoral fins, dorsal and caudal fins. Photophore presence and position varies among specimens; some specimens lack photophores, others with photophores only on ventral and dorsal sides and fins. Conspicuously absent on ventral collar in all specimens.

#### Geographic distribution

**Fig 11:**
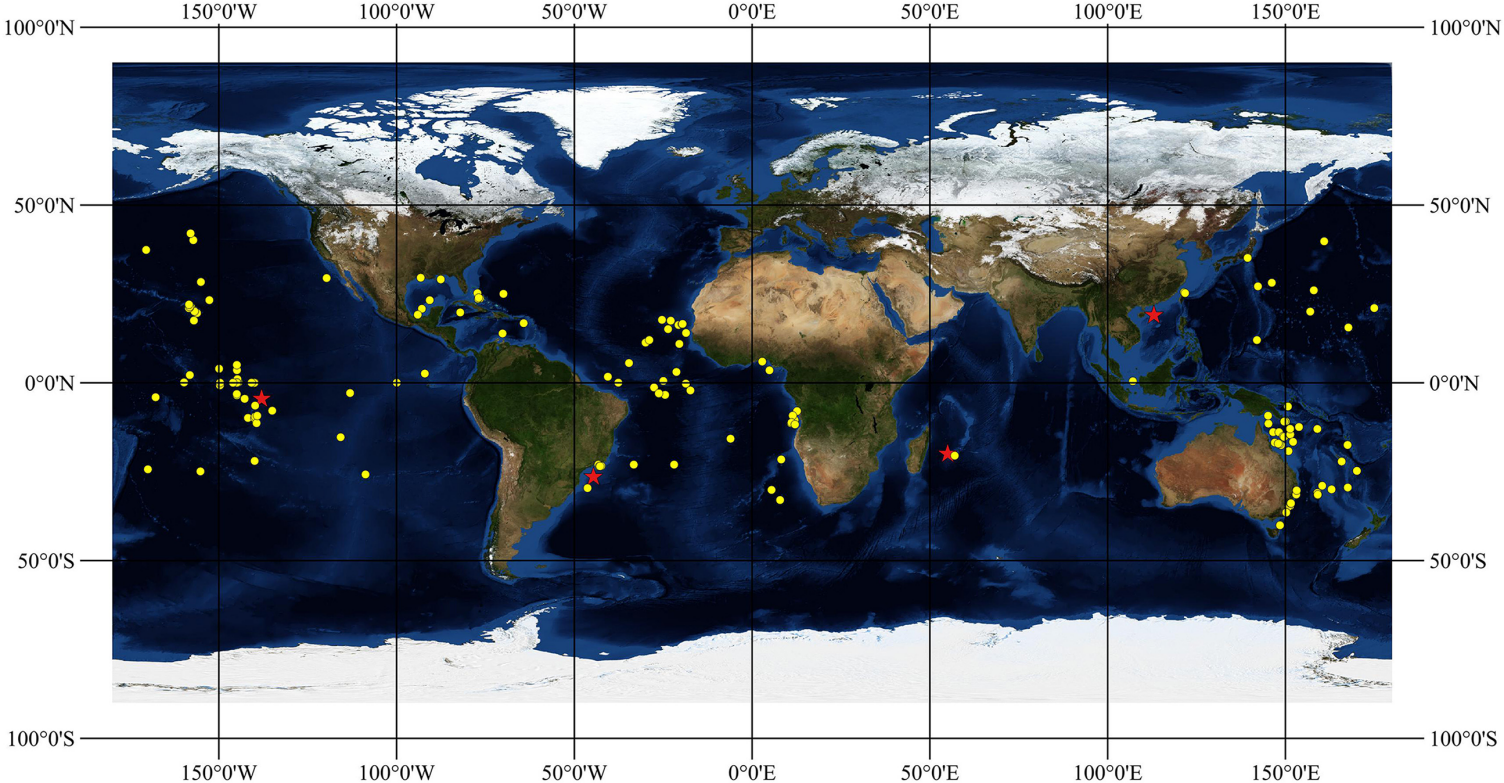
Distribution of *Isistius brasiliensis*. Yellow circles: analyzed specimens; red stars: holotypes of *Isistius brasiliensis* and of nominal species in its synonymy. World base map credit: Reto Stöckli, NASA Earth Observatory.

Distributed worldwide in tropical and subtemperate waters (Fig 11). Known from the western (southern Brazil to Gulf of Mexico) to eastern Atlantic (South Africa to Mauritania and Cape Verde), western (Tasmania to Japan, including Indonesia and Taiwan) to eastern Pacific (Easter Is., Galapagos Is. to Baja California) including French Polynesia and Hawaii, and Indian (Indonesia and Mauritius) oceans. Usually an oceanic species in the epi- to mesopelagic realms, from the surface to 3,700 m in depth [19]. Its distribution is delimited mainly by surface (10° to 30° C) and depth (1.5° to 2.5° C at 3,500 m) temperatures. Other physical-chemical parameters associated with their occurrence include 4 to 7 ml/l of dissolved oxygen at the surface, 0 to 1 μmol/l of surface phosphate and low volume of surface silicates (up to 10 μmol/l) [341].

#### Etymology

The specific epithet *brasiliensis* refers to the locality of the holotype from the Brazilian coast, off Rio de Janeiro.

#### Common names

English: cookiecutter shark; French: squalelet féroce; German: Zigarrenhai; Portuguese: tubarão charuto; Spanish: tollo cigarro.

#### Remarks

**Fig 12:**
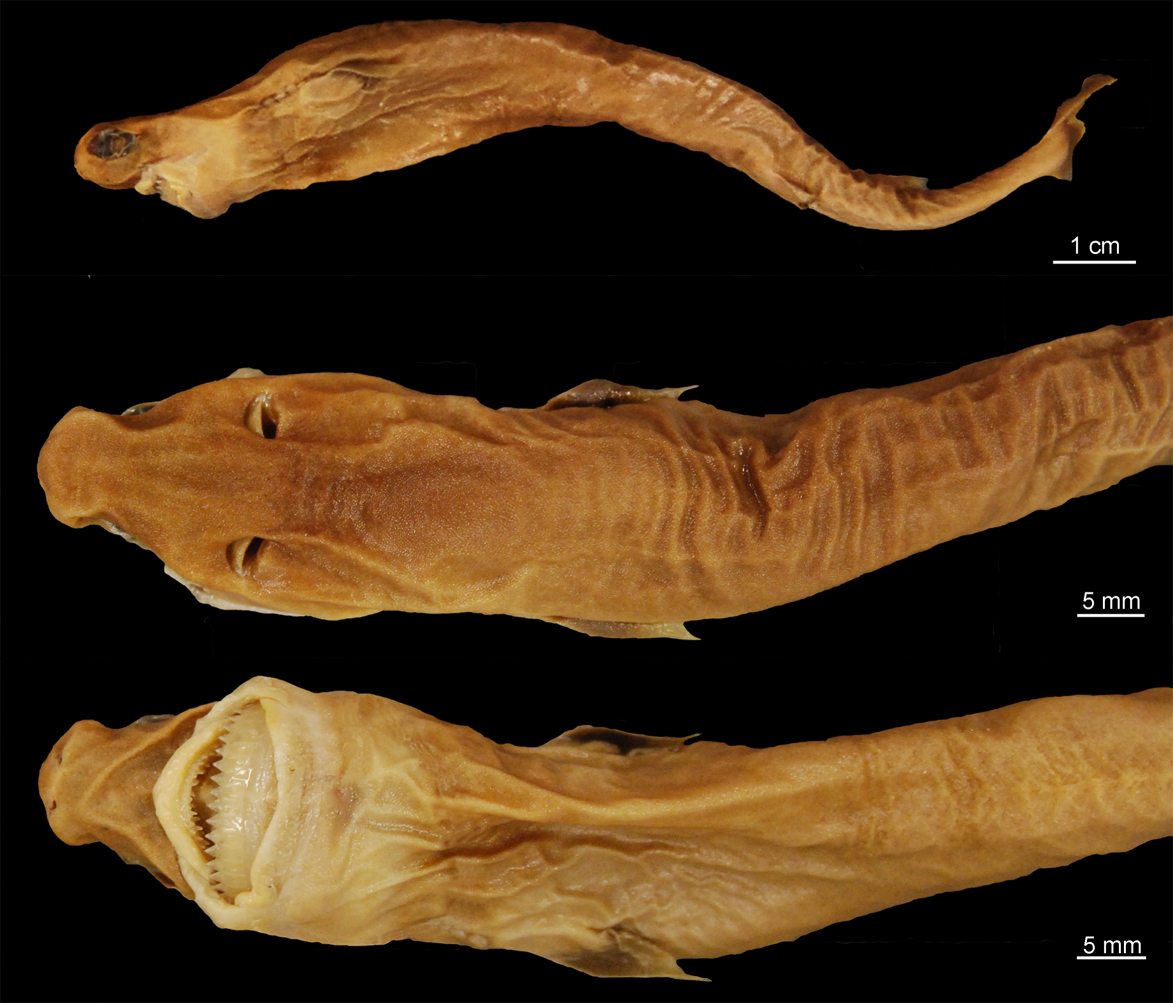
Specimen of *Isistius brasiliensis (*MNHN A-7787). Holotype of *Isistius brasiliensis* (Quoy & Gaimard, 1824) [16] and of the junior synonyms *Scymnus brasiliensis* Quoy & Gaimard (1824) [16] and *Scymnus brasiliensis torquatus* var Valenciennes [A.] in Müller & Henle (1839) [30]. From the Southeastern coast of Brazil, Atlantic Ocean. TL = 172 mm. Left lateral (top), dorsal (middle), and ventral (bottom) views.

**Fig 13:**
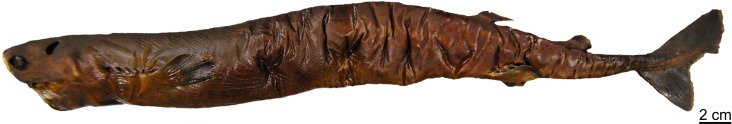
Specimen of *Isistius brasiliensis (*MNHN 0000–1178). Holotype of the junior synonym *Scymnus brasilensis unicolor* var Valenciennes [A.] in Müller & Henle (1839) [30]. From Mauritius Island, Indian Ocean. TL = 471 mm. Left lateral view.

**Fig 14:**
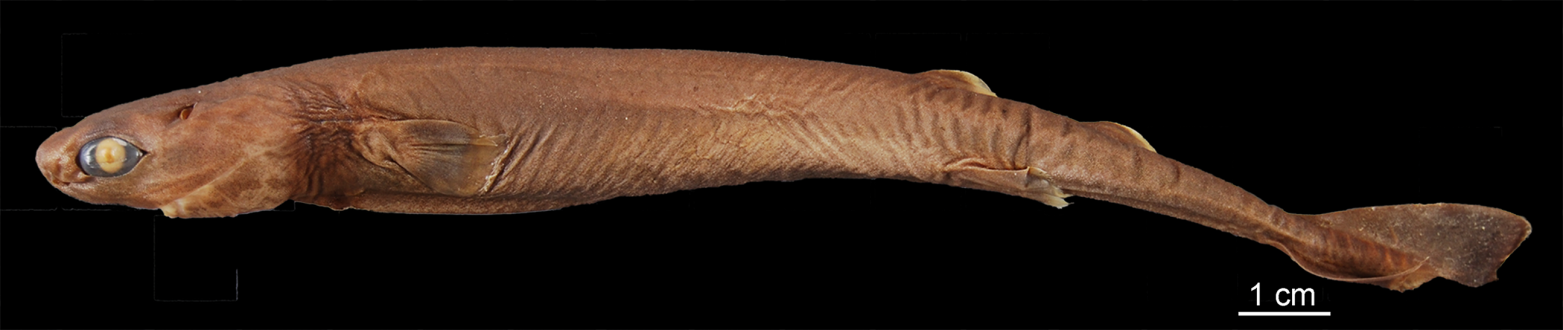
Specimen of *Isistius brasiliensis* (NMW 76230). Holotype of the junior synonym *Leius ferox* Kner (1865) [31]. From Australia, Pacific Ocean. TL = 162 mm. Left lateral view.

**Fig 15:**
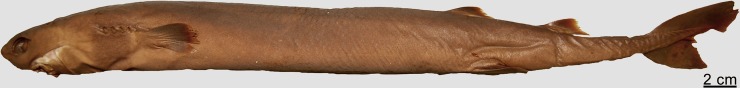
Specimen of *Isistius brasiliensis* (SCSFRI S07257). Holotype of the junior synonym *Isistius labialis* Meng, Zhu & Li (1985) [17]. From South China Sea, Pacific Ocean. TL = 442 mm. Left lateral view.

All extant holotypes of nominal species considered junior synonyms of *Isistius brasiliensis* in this study were examined: *Scymnus brasiliensis* Quoy & Gaimard (1824) [16] (Fig 12), *Scymnus brasiliensis torquatus* Valenciennes [A.] in Müller & Henle (1839) [30] (Fig 12), *Scymnus brasiliensis unicolor* Valenciennes [A.] in Müller & Henle (1839) [30] (Fig 13), *Leius ferox* Kner (1864) [31] (Fig 14), and *Isistius labialis* Meng, Zhu & Li (1985) [17] (Fig 15). The holotypes of *Squalus fulgens* (Bennett, 1840) [18] and *Isistius marmoratus* Rochebrune (1885) [32] were not found in collections, and the original descriptions do not indicate their possible whereabouts.

Holotypes of the nominal species *Scymnus brasiliensis* and *Scymnus brasiliensis torquatus* are based on the same specimen (and are therefore objective synonyms), the former described as a species by Quoy & Gaimard (1824) [16] and the latter described as a subspecies of *I*. *brasiliensis* by Müller & Henle (1839) [30]. This specimen was collected before 1824 and is in poor condition, besides being a juvenile. Therefore, its measurements are not very accurate due to damage of fins and post-mortem deformation.

Another holotype, also described by Müller & Henle [30], *Scymnus brasiliensis unicolor*, is also not in good condition, although this specimen is a large adult (470 mm). The name given, *unicolor*, is in reference to the absence of the ventral dark collar in the gill region. However, this specimen is very dark brown and the difficulty in observing the collar might be a result of its dark color, as observed in many other analyzed specimens.

In the description of *Isistius labialis*, Meng, Zhu & Li [17] mentioned, among other features, the presence of a rounded projection on the lower lip of their specimen (the holotype). But since this trait was not found in any other specimen and is very inconspicuous, it may be considered a variable feature of *I*. *brasiliensis*. Although the holotypes show some morphological differences, which are also present in many other examined specimens of *I*. *brasiliensis*, the recognition of specific morphotypes and populations by region is not justified, and *I*. *brasiliensis* is recognized as a widely distributed species represented by specimens with slightly variable colors and sizes.

### *Isistius plutodus* Garrick & Springer (1964) [15]

(Fig 16)

**Fig 16:**
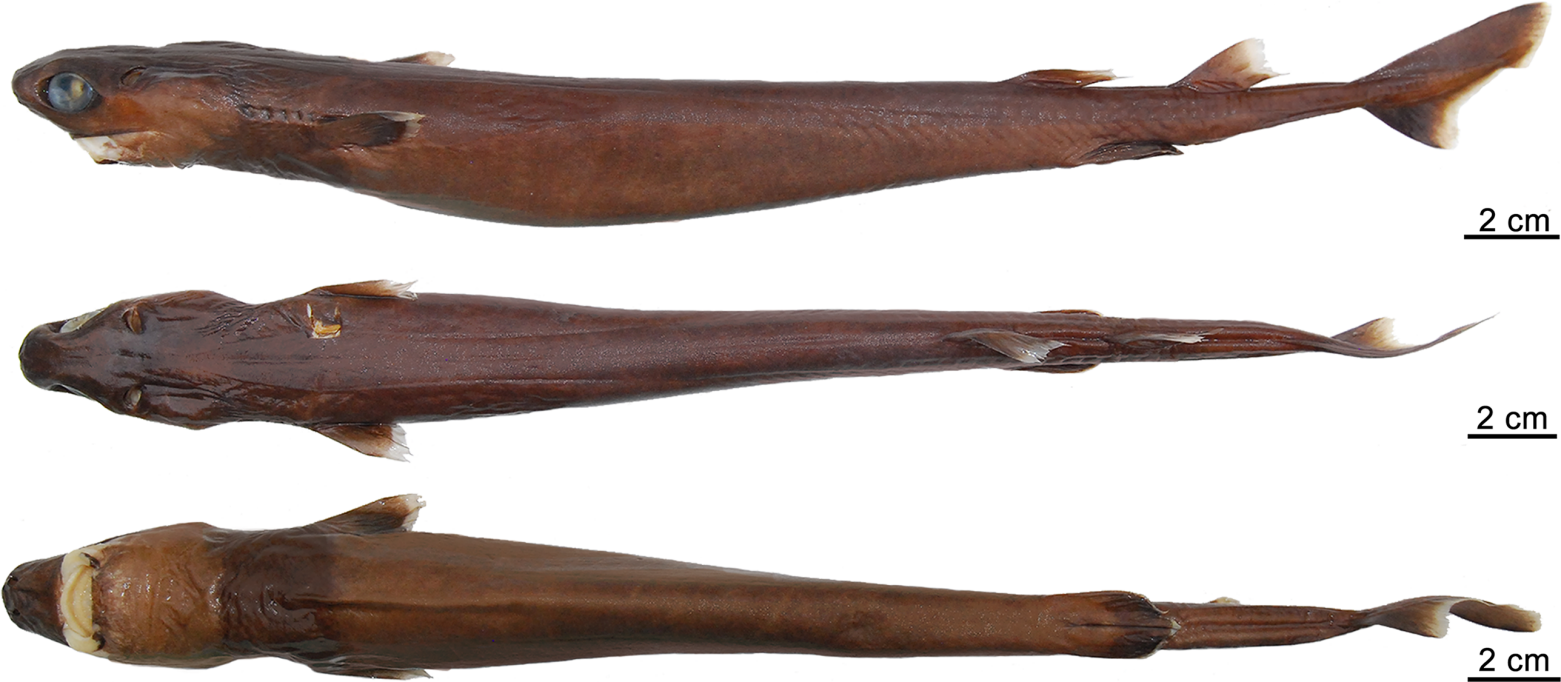
Specimen of *Isistius plutodus* (HUMZ 210817). Left lateral (top), dorsal (middle), and ventral (bottom) views; from Northwestern Pacific Ocean.

**Fig 17:**
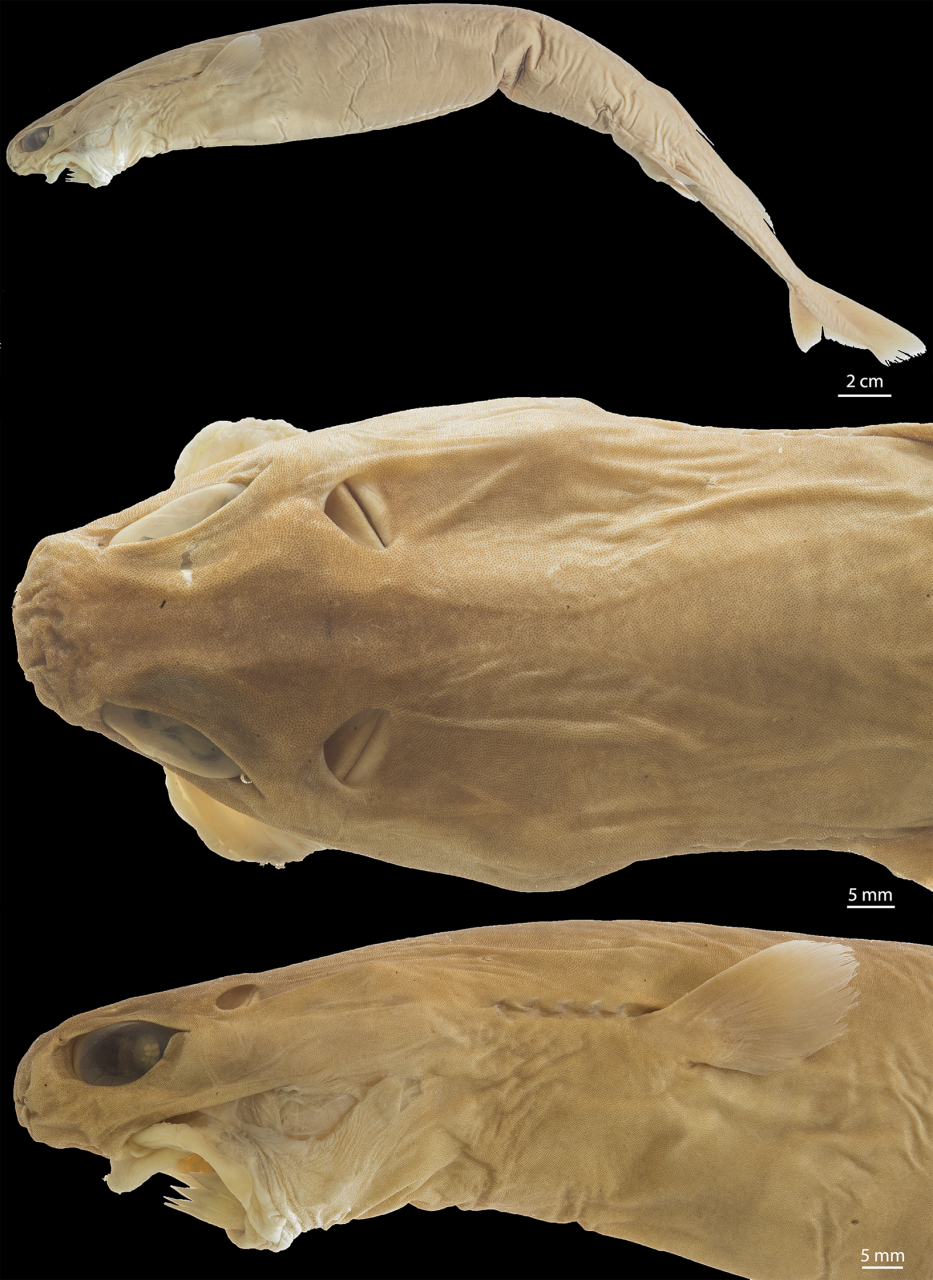
Holotype of *Isistius plutodus* Garrick & Springer (1964) [15] (USNM 188386). From Gulf of Mexico. TL = 416 mm.

*Isistius plutodus* Garrick & Springer (1964): 679, figs 1A, 2A, 2C (original description; type locality: off the coast of Alabama, Gulf of Mexico) in [15]; Jones (1971): 791 (feeding) [110]; Figueiredo (1977): 9 (compilation) [114]; Cadenat & Blache (1981): 105, 109 (in key, description) [120]; Compagno (1981): 9, 10 (teeth) [342]; Compagno (1984): 96 (diagnosis, range, biology) [13]; Reif (1985): 113 (photophores compared to *Dalatias licha* and *Isistius brasiliensis*) [124]; Jahn & Haedrich (1987): 298, 299, fig 47 (distribution, morphometrics, predation) in [20]; Sadowsky *et al*. (1988): 919 (first catch in the southern hemisphere, Brazil) [129]; Herman *et al*. (1989): 103 (tooth counts), 122 [131]; Howe & Springer (1993): 16 (listed) [343]; Gadig (1994): 33, 43, 45, 47 (predation, in key, Brazil) [144]; Mullin *et al*. (1994): 467 (predation) [147]; Amorim *et al*. (1998): 623, 629 (southeastern Brazil) [153]; Lessa *et al*. (1999): 28, 55, 57 (south/southeastern Brazil) [344]; Kiraly *et al*. (2000): 2, 9 (habitat, biology, U.S.A.) [165] 1999; Gadig (2001): 30, 84, 86, 87, 224, 232, 233, 239 (in key, description, range, biology, Brazil) [169]; Soto (2001a): 66, 93, 94 (listed, Brazil) [173]; Soto & Mincarone (2001): 23 (feeding) [174]; Pérez-Zayas *et al*. (2002): 308, 309 (predation on marine mammals) [178]; Moore *et al*. (2003): 388 (distribution inferred from bites on swordfishes) [184]; Zidowitz (2003): 1–4 (biology compared to *I*. *brasiliensis*) [186]; Makino *et al*. (2004): 169 (predation) [190]; Soto & Mincarone (2004): 7, 70 (listed) [194]; Zidowitz *et al*. (2004): 1430–1433 (notheast Atlantic) [345]; Compagno *et al*. (2005a): 128, 129, pl. 14 (distribution, description, teeth, illustrated) in [33]; Kyne *et al*. (2005): 321 (Queensland, Australia) [346]; George & Zidowitz (2006): 76 (North Atlantic Ocean, deepsea) [208]; Kyne *et al*. (2006): 15 (conservation) [209]; Yearsley *et al*. (2006): 16 (common name: Largetooth Cookiecutter Shark) [214]; Kyne & Simpfendorfer (2007): 9 (vertical migration) [218]; Silva-Jr. *et al*. (2007): 509 (predation) [224]; Souto *et al*. (2007): 22 (predation) [225]; Compagno (2008): 19 (compilation) [230]; Renner & Bell (2008): 102 (predation) [239]; Bornatowski *et al*. (2009): 2 (southern Brazil) [242]; Camhi *et al*. (2009): 9, 44, 75 (habitat) [243]; Souto *et al*. (2009): 2 (predation) [248]; Largacha *et al*. (2010): 1 (teeth) [254]; Castro (2011): 140, 141, 151, 152 (identification, distribution, in key, teeth, dermal denticles, illustrated) [262]; Dwyer & Visser (2011): 111–113 (distribution, predation) [264]; Claes *et al*. (2012): 1691 (compilation) [265]; Kyne *et al*. (2012): 40, 142 (conservation) [269]; Wenzel & Suárez (2012) (Cape Verde) [273]; Ebert & Stehmann (2013): 126, 131 (description, illustrated) [277]; Ebert *et al*. (2013a): 166, 167, 171 (destribuion, description, illustrated) [1]; White & Last (2013): 236 (identification) [279]; Carrillo-Briceño *et al*. (2014): 11 (fossil record) [281]; Dulvy *et al*. (2014): 8 (conservation) [347]; Rosa & Gadig (2014): 93 (Brazil) [284]; Ebert *et al*. (2015) (world distribution except Eastern Pacific) [12]; Stehmann & Kukuev (2015): 73–77 (southwestern Atlantic, description, illustration) [34]; Weigmann (2016): 892 (checklist) [291]; Perez & Marks (2017): 145, 146, 148 (habitat, feeding) [293].
*Isistius plotudus*: Falcón-Matos *et al*. (2003): 164 (bites) [348].
*Isistius plutodon*: Wetherbee *et al*. (2012): 241 (unusual tooth and jaw morphology) [274].
*Holotype*. USNM 188386 (holotype), female 416 mm TL, Western Atlantic, Gulf of Mexico, off Mississippi delta, 28°58’ N, 88°18’ W, 18 m midwater trawl in depth of bottom 814–997 m, Oct 27, 1960 (Fig 17).

#### Diagnosis

A species of *Isistius* differentiated from its only congener, *I*. *brasiliensis*, by the following characters: snout shorter and less rounded (*vs*. snout longer and more rounded in *I*. *brasiliensis*); preorbital (12.01% HDL *vs*. 15.50% HDL in *I*. *brasiliensis*), prenasal (4.79% HDL *vs*. 6.27% HDL), preoral (23.15% HDL *vs*. 32.46% HDL), and interorbital (17.27% HDL *vs*. 25.48% HDL) lengths (relative to head length) proportionately smaller than in *I*. *brasiliensis*; head length 3.3 times larger than interdorsal space (*vs*. 2.25); second dorsal fin higher than first (*vs*. second dorsal fin almost same height as first in *I*. *brasiliensis*); lower symphyseal tooth larger (its height 2.08% TL *vs*. 1.41% TL), and tooth base wider (1.08% TL *vs*. 0.62% TL); lower symphyseal tooth the same height as adjacent teeth in *I*. *plutodus*, whereas tooth 5% shorter than adjacent teeth in *I*. *brasiliensis*; relatively less tooth rows, with tooth formula for upper/lower teeth 12+12/9+1+9 (*vs*. 15+15/12+1+13); lower teeth proportionally larger, 6 times greater than upper teeth (*vs*. 3 times in *I*. *brasiliensis*); oral integument with straight anterior margin and broad (*vs*. concave anterior margin and relatively narrow in *I*. *brasiliensis*); upper postventral margin of caudal fin greater than lower margin (*vs*. upper margin smaller than lower postventral margin in *I*. *brasiliensis*); general body color darker than in *I*. *brasiliensis*; ventral dark collar proportionately larger (posterior end at level of pectoral fin insertion) than in *I*. *brasiliensis* (posterior end at level of pectoral fin origin). Morphology and proportions of neurocranium also differs between both species (Table 3): neurocranium in *I*. *plutodus* has smaller nasobasal length (54.17% HDL *vs*. 56.08% HDL), shorter otic capsule (15.87% HDL *vs*. 18.05% HDL), and smaller width across postorbital processes (22.49% HDL *vs*. 27.74% HDL) even though the postorbital process itself is longer in *I*. *plutodus* than in *I*. *brasiliensis* (6.13% HDL *vs*. 4.81% HDL).

#### Description

Morphometric data for this species is given in Table 6, and meristic data and tooth rows, and vertebral counts, in Tables 4 and 5, respectively.

**Table 6:** Morphometric characterization of *Isistius plutodus*.

	USNM 188386 (holotype)		n	Range				Mean	SD
	mm	%TL		mm		%TL		%TL	%TL
**TL**	416	416	6	329	426	-	-	-	-
**PCL**	356	85.6%	6	284	378	85.6%	88.7%	87.2%	1.3%
**PD2**	308	74.0%	6	245	323	73.4%	75.8%	74.5%	0.9%
**PD1**	269	64.7%	6	210	284	63.2%	66.7%	64.8%	1.3%
**SVL**	292	70.2%	6	215	295	65.3%	70.6%	68.9%	1.9%
**PP2**	281	67.5%	6	213	286	64.7%	67.9%	66.8%	1.2%
**PP1**	76.18	18.3%	6	65.5	86.04	18.3%	20.2%	19.7%	0.7%
**HDL**	78.69	18.9%	6	65.68	83.76	18.9%	20.3%	19.8%	0.5%
**PG1**	62.77	15.1%	6	52.21	65.2	15.1%	16.8%	15.8%	0.6%
**PSP**	28.74	6.9%	6	24.06	31.19	6.9%	8.8%	7.7%	0.7%
**POB**	6.78	1.6%	6	6.78	10.37	1.6%	3.1%	2.4%	0.5%
**PRN**	3.13	0.8%	6	2.14	4.76	0.7%	1.4%	1.0%	0.3%
**POR**	16.42	3.9%	6	13.33	20.68	3.7%	6.2%	4.7%	0.9%
**PINL**	3.55	0.9%	6	3.32	5.91	0.9%	1.8%	1.4%	0.4%
**INFL**	19.82	4.8%	5	13.32	22.87	3.1%	6.2%	5.1%	1.2%
**MOW**	34.49	8.3%	5	22.18	34.49	5.9%	8.3%	7.2%	0.9%
**ULA**	16.43	3.9%	6	11.5	24.5	3.5%	5.8%	4.3%	0.9%
**INW**	5.59	1.3%	6	3.67	6.59	1.1%	1.5%	1.3%	0.2%
**INO**	13.7	3.3%	6	9.57	13.7	2.9%	3.9%	3.3%	0.3%
**EYL**	15.63	3.8%	6	13.5	16.22	3.6%	4.7%	4.1%	0.4%
**EYH**	10.8	2.6%	6	8.34	11.22	2.5%	3.1%	2.7%	0.2%
**SPL**	9.01	2.2%	6	5.82	9.01	1.8%	2.2%	2.0%	0.1%
**GS1**	3.14	0.8%	6	2.46	3.73	0.7%	1.1%	0.8%	0.1%
**GS5**	2.97	0.7%	6	2.05	2.97	0.6%	0.7%	0.7%	0.0%
**IDS**	27.82	6.7%	6	11.75	27.82	3.6%	7.3%	5.9%	1.3%
**DCS**	33.71	8.1%	6	22.84	33.71	6.2%	8.4%	7.4%	0.8%
**PPS**	185.12	44.5%	6	146.62	191	44.1%	45.0%	44.6%	0.3%
**PCA**	68.01	16.3%	6	52.57	68.01	14.4%	18.4%	16.3%	1.3%
**D1L**	33.36	8.0%	6	26.49	34.04	8.0%	8.5%	8.2%	0.2%
**D1A**	22.37	5.4%	6	17.73	25.45	5.2%	6.8%	5.7%	0.6%
**D1B**	16.24	3.9%	6	13.47	16.66	3.9%	4.9%	4.3%	0.4%
**D1H**	10.34	2.5%	6	6.38	10.34	1.9%	2.5%	2.2%	0.2%
**D1I**	15.38	3.7%	6	10.94	17.02	3.3%	4.1%	3.7%	0.3%
**D1P**	15.33	3.7%	6	9.79	15.88	2.9%	3.7%	3.4%	0.3%
**D2L**	32.33	7.8%	6	25.23	34.4	7.7%	8.3%	8.0%	0.2%
**D2A**	25.2	6.1%	6	18.27	25.4	5.5%	6.8%	6.1%	0.4%
**D2B**	19.17	4.6%	6	14.77	20.48	4.4%	5.2%	4.8%	0.3%
**D2H**	14.17	3.4%	6	8.68	14.93	2.6%	3.5%	3.2%	0.4%
**D2I**	12.68	3.0%	6	9.13	13.93	3.0%	3.5%	3.3%	0.2%
**D2P**	16.64	4.0%	6	11.34	18.42	3.4%	4.3%	3.9%	0.3%
**P1A**	32.04	7.7%	6	20.69	32.04	5.7%	8.2%	7.0%	0.9%
**P1I**	19.2	4.6%	6	8.99	19.2	2.7%	4.6%	3.7%	0.6%
**P1B**	9.59	2.3%	6	7.78	10.33	2.3%	3.1%	2.6%	0.3%
**P1P**	14.33	3.4%	6	10.73	17.33	3.3%	4.2%	3.7%	0.4%
**P2L**	27.99	6.7%	6	24.91	29.18	6.7%	7.9%	7.4%	0.5%
**P2H**	9.45	2.3%	6	3.82	9.45	1.1%	2.3%	1.7%	0.5%
**P2I**	14.67	3.5%	6	2.15	16.03	0.7%	4.6%	2.8%	1.7%
**CDM**	57.58	13.8%	6	46.13	60.27	13.8%	14.8%	14.2%	0.3%
**CPV**	24.77	6.0%	6	21.67	25.89	6.0%	7.5%	6.7%	0.6%
**CPU**	40.84	9.8%	6	23.64	40.84	7.2%	10.2%	9.0%	1.1%
**CPL**	10.05	2.4%	6	5.83	10.31	1.8%	3.1%	2.2%	0.5%
**CFW**	19.8	4.8%	6	14.52	19.8	4.4%	5.9%	4.9%	0.6%
**CFL**	26.21	6.3%	6	21.69	26.21	5.5%	7.2%	6.5%	0.6%
**HANW**	8.61	2.1%	6	6.63	9.29	2.0%	2.8%	2.3%	0.3%
**HAMW**	38.19	9.2%	6	26.05	38.19	7.0%	9.2%	8.1%	0.8%
**HDW**	35.02	8.4%	6	25.12	35.02	7.6%	8.4%	8.0%	0.4%
**TRW**	26.96	6.5%	6	19.68	38.98	5.9%	9.3%	7.6%	1.4%
**ABW**	13.16	3.2%	6	8.34	14.98	2.5%	3.5%	3.1%	0.4%
**TAW**	11.58	2.8%	6	7.92	13.6	2.4%	3.5%	2.8%	0.4%
**CPW**	4.42	1.1%	6	3.17	4.75	1.0%	1.4%	1.1%	0.2%
**HDH**	33.58	8.1%	6	22.99	41.75	6.9%	9.8%	7.8%	1.0%
**TRH**	30.86	7.4%	6	22.79	44.04	6.8%	10.3%	7.8%	1.3%
**ABH**	17.51	4.2%	6	12.44	22.9	3.7%	5.4%	4.6%	0.6%
**TAH**	13.43	3.2%	6	9.54	16.88	2.9%	4.0%	3.4%	0.4%
**CPH**	5.6	1.3%	3	4.2	6.07	1.3%	1.5%	1.4%	0.1%
**CLO**	-	-	3	6.18	6.83	1.9%	2.1%	1.9%	0.1%
**CLI**	-	-	3	15.93	17.27	4.8%	5.2%	5.0%	0.2%
**CLB**	-	-		2.46	2.58	0.7%	0.8%	0.8%	0.0%

All values are presented as percentages of total length (TL) except TL, given in mm.

Monospondylous vertebral counts 43–45, precaudal diplospondylous 23–26, and postcaudal diplospondylous 22–27. Monospondylous to diplospondylous transition occurs at level between the dorsal fins.

#### External morphology

Trunk cigar shaped, cylindrical and slender, tapering toward pelvic and caudal fin origins. Predorsal length about 4.5 times caudal fin length. Greatest height of trunk just posterior to level of pectoral fins tips when adpressed. Lateral outline of head rectangular, more triangular anterior to mouth. Dorsal head profile parallel to ventral, slopping slightly toward bulbous, rounded snout tip. Eyes large (20.6% HDL), rounded, slender posteriorly. Preorbital length short (11.4% HDL); interorbital space 0.82 times eye length. Spiracle large (10.3% HDL), dorsal, posterior eye, transverse but somewhat oblique (inner portion directed slightly posteriorly); prespiracular length 3.74 times spiracle length. Spiracles oval, their largest width transverse to body axis. Gill slits very small (0.86–0.66% of TL), first greater than fifth, well behind eye and just anterior to pectoral fin origin (Fig 17).

**Fig 18:**
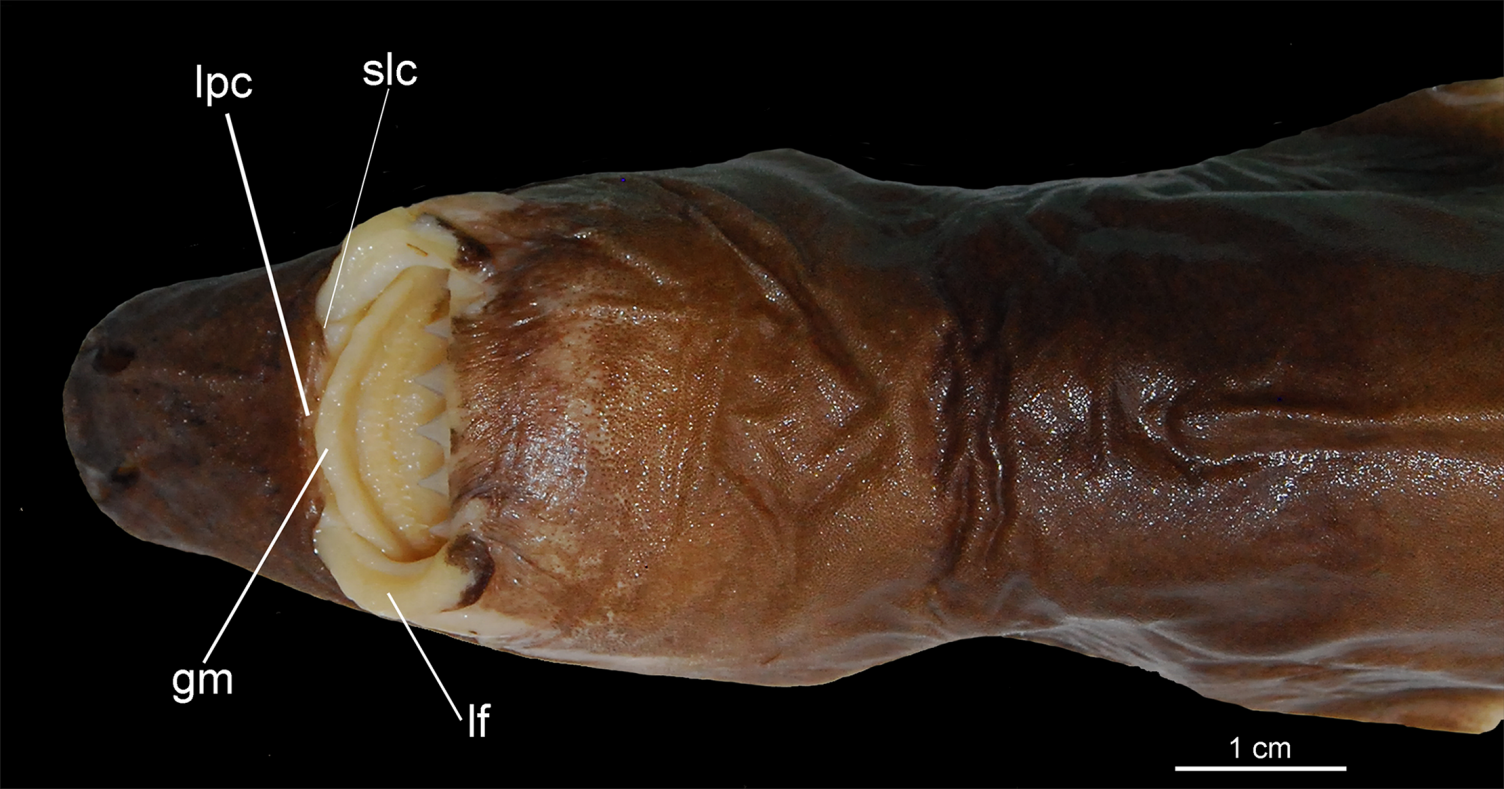
Ventral view of mouth region of *Isistius plutodus* (HUMZ 210817). Abbreviations: **gm**, gum; **lf**, lip fold; **lpc**, upper lip cover; **slc**, fleshy sack for the upper labial cartilages.

Nostrils anteroventral on snout, anteriorly rounded, with a nasal flap dividing anterior and posterior apertures; posterior region of nostril elongated and slightly directed toward midline, lacking barbels. Internarial length 1.42 times prenarial length. Mouth wide, transverse (7.4% of TL), with lateral skin folds and deep grooves (21.6% of head length); lower lip thick and wrinkled. Upper lip cover (**lpc**) contiguous with lower ventral surface of head anterior to mouth; upper lip cover with thick and naked lip fold (**lf**) on lateral and internal portion (Fig 18). Naked lip fold is a short tissue at mouth corner posteriorly directed and slightly turned toward medial region of lower jaw; fold rounded, visible when mouth closed, and upper lip covers almost its whole length. Thick and straight gum (**gm**) and a fleshy sack for upper labial cartilages (**slc**) immediately dorsal to upper lip cover. Deep groove dorsal to lip fold extending from anterior to labial cartilages to almost posterior end of lower jaw. Posterior mouth groove from corner of mouth to posterior region, almost reaching ventral collar. Deep oral pocket at corner of mouth and immediately dorsal to lip fold. Preoral pouch anterior to corner of mouth, and dorsal to labial cartilages joint and upper labial cartilage [14].

Pectoral fins originate immediately posterior to fifth gill slit. Pectoral fins terminate abruptly; their lengths 10.4% of predorsal body length. Anterior pectoral margins straight and almost parallel to body axis, and posterior margins almost perpendicular to body axis, with corners slightly curved. Inner pectoral margins somewhat oblique, directed toward ventral side at their most posterior portion. Pectoral base 2.9 times first gill slit. Dorsal fins without spines. First dorsal very small and obliquely inclined rearward; middle of its base at origin of pelvic fins, but very posterior to tip of pectorals. First dorsal origin at about 3/4 of precaudal body length. First dorsal base length 1.93 times fin height and about 21.6% of head length. Posterior first dorsal margin straight, forming a triangle with anterior margin, and free lower tip forms an acute angle. Second dorsal fin slightly higher and longer than first dorsal. Second dorsal base length 1.48 times its height. Second dorsal fin base 1.11 times first dorsal base, and second dorsal height 1.45 times first dorsal height. Second dorsal fin origin above end of pelvic fin free rear tip; its posterior margin similar to first dorsal, although its free rear tip with a prolonged filament.

Interspace between first and second dorsals slightly larger (about 1.23 times) than space between second dorsal and caudal fins. Caudal fin asymmetrical, posterior end of vertebral column slightly directed upwards. Caudal peduncle without lateral ridges and width 0.8 times its height. Dorsal caudal margin 2.14 times preventral margin, the latter originating slightly in advance of the former. Dorsal caudal lobe with oblique terminal margin. Posterior end of dorsal caudal margin posterior to end of subterminal margin. Upper caudal postventral margin more than twice length of lower postventral margin. Pelvic fins originate posterior to insertion of first dorsal fin, but anterior to its free rear tip. Pelvic fin length 0.92 times first dorsal-fin length. Pelvic fins in females with reduced angle and almost straight rear tip; in males, pelvic fins shorter with smaller angle; claspers of adult slightly longer than end of fin. In ventral view, most distal portion of clasper white, triangular in outline, with straight internal margin and oblique outer margin directed toward midline. In dorsal view, clasper groove originates from its most anterior portion, almost at dorsal insertion of pelvic fin. Clasper groove somewhat straight and dorsal along clasper axis, ending at gland. Juvenile claspers not reaching rear tip of pelvic fin.

#### Teeth

**Fig 19:**
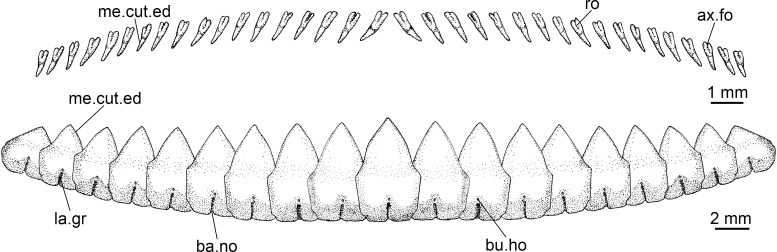
Upper and lower teeth of *Isistius plutodus* (ZUEC 8333) in labial view. Top. Upper teeth; bottom. Lower teeth. Abbreviations: **ba.no**, basal notch; **bu.ho**, button hole; **la.gr**, labial groove; **me.cut.ed**, mesial cutting edge; **ro**, root.

**Fig 20:**
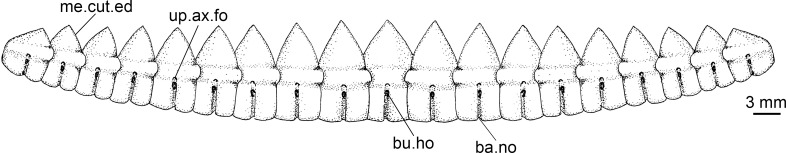
Lower teeth of *Isistius plutodus* (ZUEC 8333) in lingual view. Abbreviations: **ba.no**, basal notch; **bu.ho**, button hole; **me.cut.ed**, mesial cutting edge; **up.ax.fo**, upper axial foramen.

Dignathic heterodonty present, with palatoquadrate and mandibular teeth unicuspid, and smooth mesial cutting edges (**me.cut.ed**). On average, 25 (13+12) upper teeth with no teeth at palatoquadrate symphysis, but two parasymphyseal directed toward opposite sides. Upper teeth narrow, sharp, slightly distally inclined, not overlapping, usually in three rows, and multiserial in function. Upper teeth root (**ro**) elongated and following the vertical line of crown; root pseudolobated with a sulcus dividing it into two regions (Fig 19); presence of a subtle axial foramen (**ax.fo**) below the sulcus on lingual side of crown. Upper teeth have a gradient of inclination, becoming more oblique toward the posterior region of mouth. Lower teeth averaging 19 (9+1+9) in a row, with a tooth at mandibular symphysis wedged between upper parasymphyseal. Lower teeth with vertical triangular cusp and flat root in only one functional row and more than three non-funcional rows, with teeth facing the opposite side, at the internal part of mandibular cartilage. Starting at the symphysis, roots of lower teeth overlap toward opposite sides, with mesial portion behind adjacent tooth and distal over the following. These interlockings leave depressions on the margins of lower teeth. Teeth form a single functional series, with each root completely exposed on outer face, and inner side closely attached to lower jaw. Labial groove (**la.gr**) present on labial side from the button hole (**bu.ho**) to basal notch (**ba.no**), and this lower indentation at the lowest part of root gives it a somewhat rounded bilobed aspect; apron (**a**) covering crown and upper part of root until half labial groove height; a small lower axial foramen (**lo.ax.fo**) above labial groove (Fig 19). Lingual side of lower teeth with an upper axial foramen (**up.ax.fo**) at the uppermost part of the root; apron (**a**) covering the lingual portion of crown and upper region of root; button hole (**bu.ho**) also visible in lingual view (Fig 20). Lower teeth greater than upper teeth (in an adult specimen, symphyseal lower tooth 6.8 mm, and parasymphyseal upper 1.2 mm). Crown of commissural teeth directed toward anterior teeth. Lower teeth proportionally six times larger than upper teeth.

#### Coloration

Body light or dark brown with dorsal side slightly darker than lower. Darker brown ventral collar around branchial region same color as dorsal side, and easily distinguished from body. Anterior portion of darker collar at level of first gill slit, its posterior portion posterior to level of pectoral fin insertion. All fins brown, with white tips. Dark brown area on both dorsal and ventral endings of caudal fin, despite white margins. Some specimens have variation in coloration, such as the absence of the dark collar, white fin tips, and darker brown caudal fin area, which are probably due to preservation.

#### Dermal denticles

**Fig 21:**
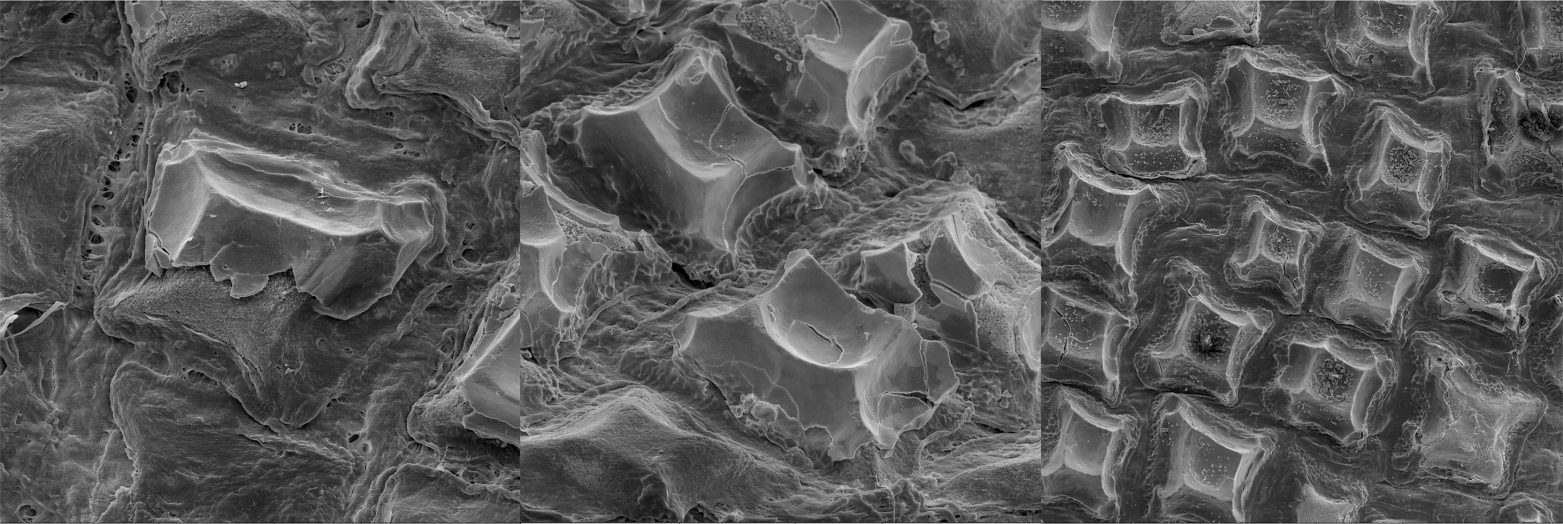
Dermal denticles of *Isistius plutodus* from Atlantic Ocean (ZUEC 8333). a. ventral collar region, magnification 200x; b. ventral post-collar, magnification 200x; c. first dorsal fin, magnification 100x.

No significant morphological distinctions were found from the denticles of *I*. *brasiliensis* (Fig 21).

#### Luminescent markings

**Fig 22:**
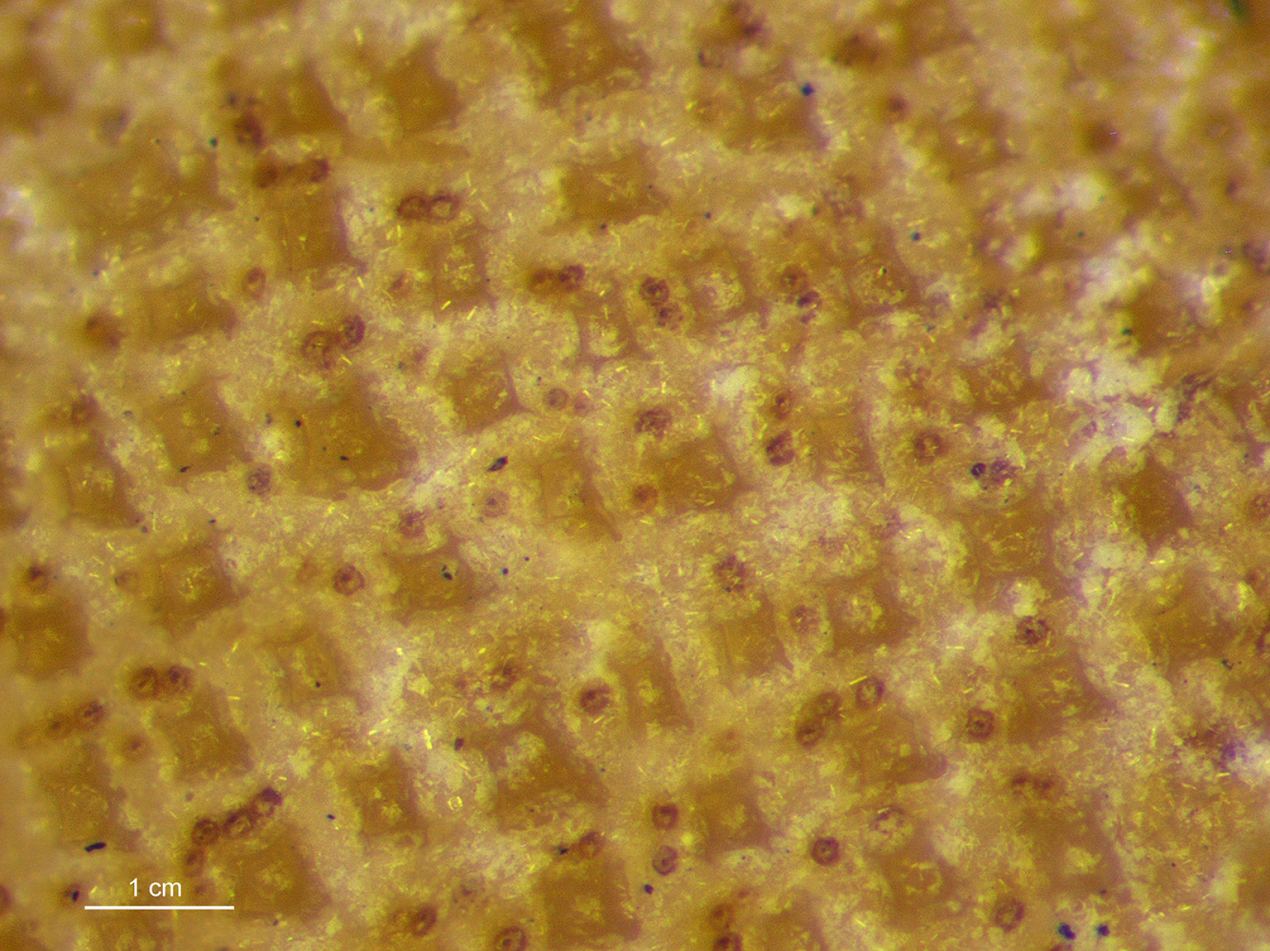
Image of ventral side at post-collar region of *Isistius plutodus* (ZUEC 8332). Photophores irregularly distributed on top of, and lateral to, dermal denticles.

Ventral patterns of photophores arranged as in *I*. *brasiliensis* (Fig 22).

#### Geographic distribution

**Fig 23:**
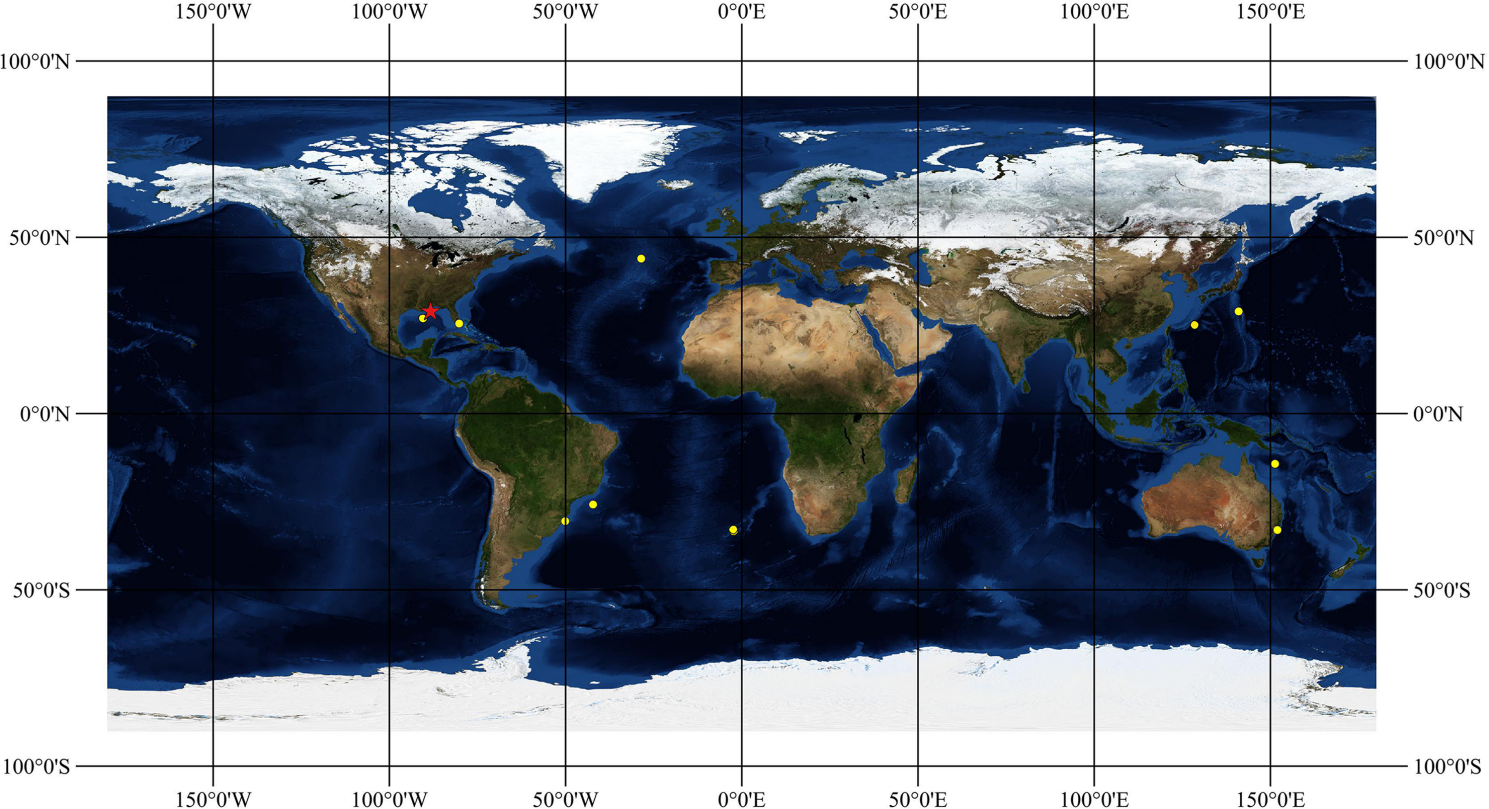
Distribution of *Isistius plutodus*. Yellow circles: analyzed and known specimens; red star: holotype of *I*. *plutodus* Garrick & Springer (1964) [15]. World base map credit: Reto Stöckli, NASA Earth Observatory.

Known from the Southwestern (Southeastern coast of Brazil), Southeastern (South Africa) [34], Northwestern (Gulf of Mexico and Florida) (M. Grace, pers. comm.), and Northeastern Atlantic, and Southwestern (Australia) and Northwestern Pacific (Japan) [349]. Probably circumglobal. Epi- to mesopelagic, from 60 to 1,300 m of depth [33] (Fig 23). Its distribution is delimited mainly by surface (15° to 25° C) and depth (1.5° to 4° C at 3,500 m) temperatures. Other physical-chemical parameters of its occurrence include 4.5 to 6 ml/l of dissolved oxygen at surface, less than 0.5 μmol/l of surface phosphate, and low surface silicates (up to 10 μmol/l) [341].

#### Etymology

The specific epithet *plutodus* comes from the Greek *ploutos* (wealth, abundance) and *odous* (tooth), in reference to its very large lower teeth in relation to body size [15].

#### Common names

English: largetooth cookiecutter shark; French: squalelet dentu; German: Großzahn-Zigarrenhai; Portuguese: tubarão charuto dentuço; Spanish: tollo cigarro dentón.

#### Remarks

Nine specimens were measured and photographed in the present study. There are 13 known specimens in total [34]. Additionally, there are five other specimens that are mentioned in the literature but that are not in collections [129,153,349,350]. Amorim *et al*. [153] mentioned four specimens, but only two are known from ZUEC.

**Fig 24:**
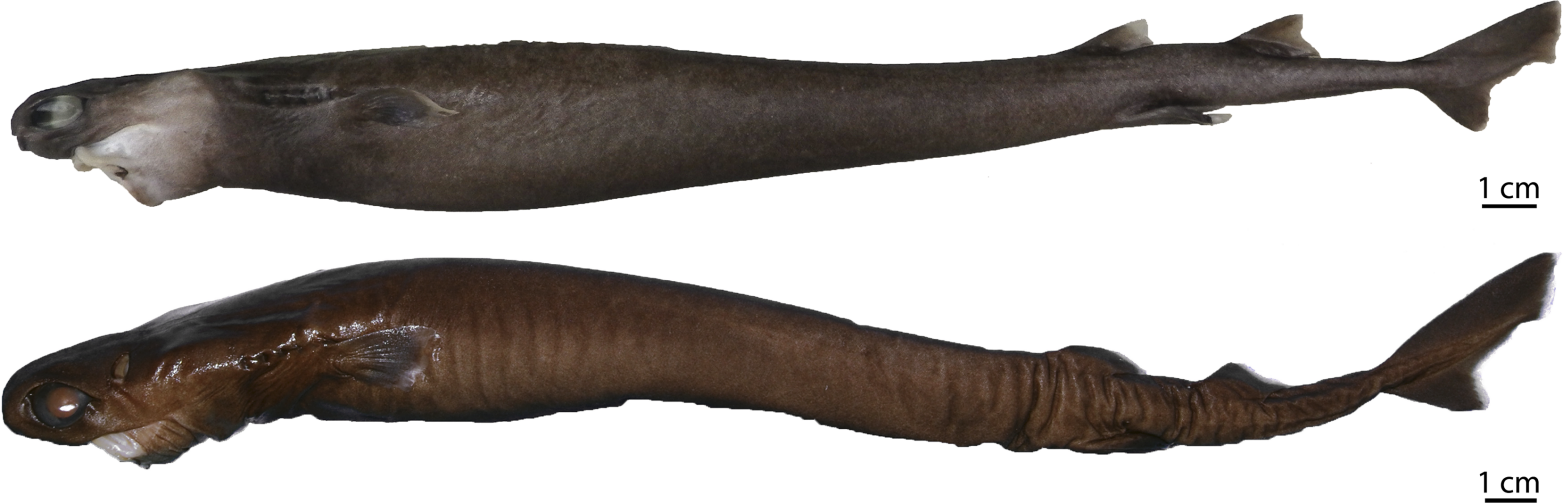
Specimens of *Isistius plutodus*. Top: Gulf of Mexico (TU 204003) and bottom: Southwestern Pacific Ocean, off Australia (AMS 43044–001).

**Fig 25:**
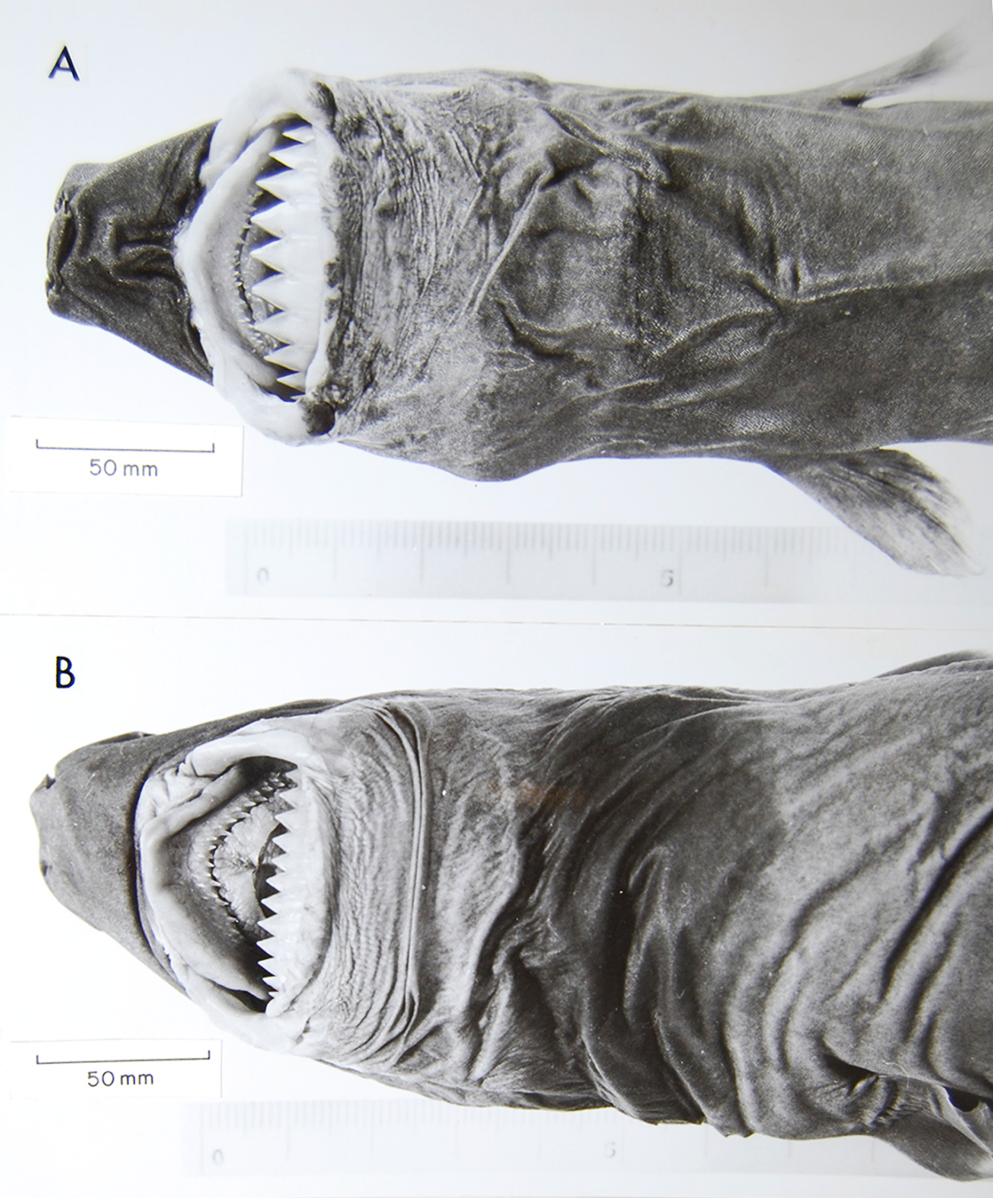
Photographs published in the description of *Isistius plutodus* by Garrick & Springer (1964) [15]. Ventral views of head of (A) *I*. *plutodus* (USNM 188386); (B) *I*. *brasiliensis* showing the dark colar in both species. Reprinted from Copeia under a CC BY license, with permission from The American Society of Ichthyologists and Herpetologists, original copyright 1964 (S3 File).

This species is easily recognizable because it has very large lower teeth and small interorbital distance when compared to *I*. *brasiliensis*, besides having a slightly darker body color. The holotype used by Garrick & Springer [15] to describe *I*. *plutodus* has lost most of its coloration, as it is light brown or beige and lacks the darker collar. Analyzed specimens from the Gulf of Mexico and Southwestern Pacific Ocean (Fig 24) are dark brown and represent the most observed color in specimens of *I*. *plutodus*. Even though there is a supposed lack of dark collar attributed to this species [13,15], this feature is clearly visible in the original photograph used by Garrick & Springer for the description of the holotype of *I*. *plutodus* (Fig 25).

### Comparative internal morphology of species of *Isistius*

#### Skeleton

**Fig 26:**
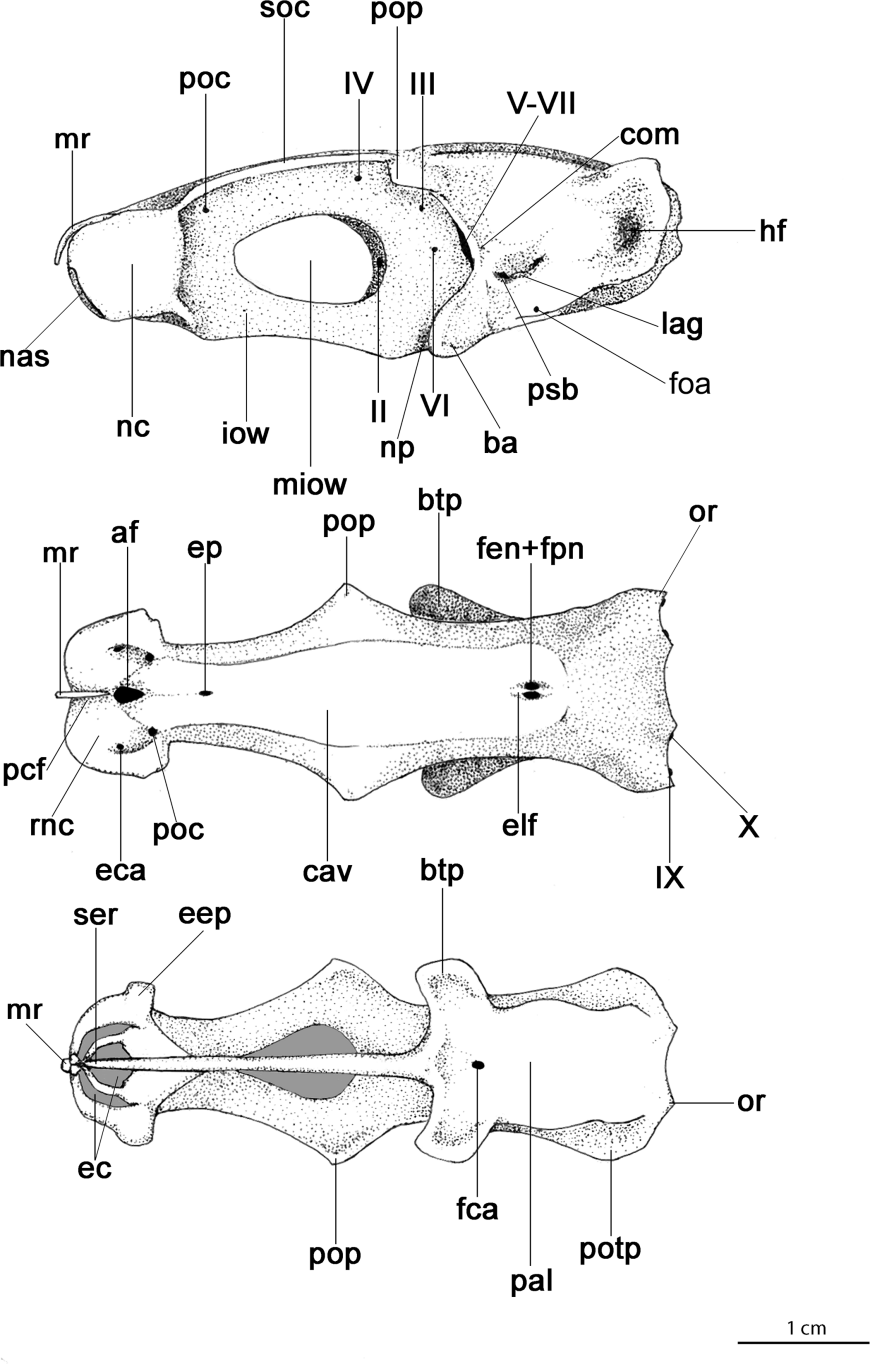
Neurocranium of *Isistius brasiliensis* (MZUSP 121506). (A) lateral (top), (B) dorsal (middle), and (C) ventral (bottom) views. Abbreviations: **af**, anterior fontanelle; **ba**, basal angle; **btp**, basitrabecular process; **cav**, cranial cavity; **com**, lateral commissure; **ec**, ectethmoidal chamber; **eca**, ethmoid canal; **eep**, ectethmoidal process; **elf**, endolymphatic fossa floor; **ep**, epiphyseal organ; **fca**, foramen for the carotid artery; **fen+fpn**, endolymphatic foramina+ perilymphatic fenestra; **foa**, foramen for orbital artery; **hf**, hyomandibula fossa; **II**, optic nerve foramen; **III**, oculomotor nerve foramen; **iow**, interorbital wall; **IV**, trochlear nerve foramen; **IX**, glossopharyngeal nerve foramen; **lag**, lateral auditory groove; **miow**, membranous interorbital wall; **mro**, medial rostral; **nas**, nasal cartilage; **nc**, nasal capsule; **np**, orbital notch; **or**, opistotic ridge; **pal**, palatine; **pcf**, precerebral fossa; **poc**, preorbital canal; **pop**, postorbital process; **potp**, postotic process; **psb**, efferent artery of the pseudobranchial; **rnc**, roof of nasal capsule; **ser**, subethmoidal crest; **soc**, supraorbital crest; **V-VII**, prootic nerve foramen; **VI**, abducens nerve foramen; **X**, vagus nerve foramen.

**Fig 27:**
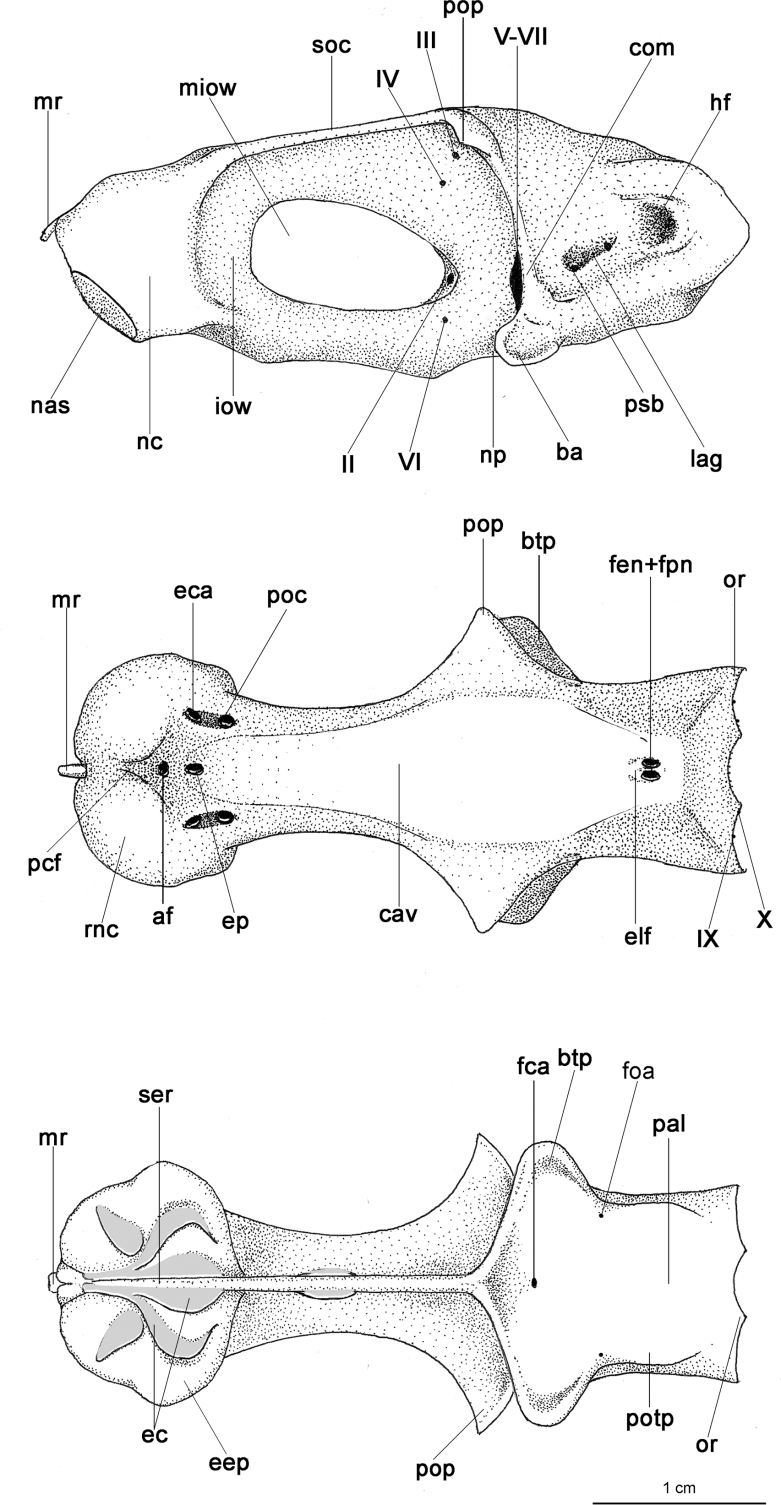
Neurocranium of *Isistius plutodus* (ZUEC 8333). (A) lateral (top), (B) dorsal (middle), and (C) ventral (bottom) views. Abbreviations: **af**, anterior fontanelle; **ba**, basal angle; **btp**, basitrabecular process; **cav**, cranial cavity; **com**, lateral commissure; **ec**, ectethmoidal chamber; **eca**, ethmoid canal; **eep**, ectethmoidal process; **elf**, endolymphatic fossa floor; **ep**, epiphyseal organ; **fca**, foramen for the carotid artery; **fen+fpn**, endolymphatic foramina + perilymphatic fenestra; **foa**, foramen for orbital artery; **hf**, hyomandibula fossa; **II**, optic nerve foramen; **III**, oculomotor nerve foramen; **iow**, interorbital wall; **IV**, trochlear nerve foramen; **IX**, glossopharyngeal nerve foramen; **lag**, lateral auditory groove; **miow**, membranous interorbital wall; **mro**, medial rostral; **nas**, nasal cartilage; **nc**, nasal capsule; **np**, orbital notch; **or**, opistotic ridge; **pal**, palatine; **pcf**, precerebral fossa; **poc**, preorbital canal; **pop**, postorbital process; **potp**, postotic process; **psb**, efferent artery of the pseudobranchial; **rnc**, roof of nasal capsule; **ser**, subethmoidal crest; **soc**, supraorbital crest; **V-VII**, prootic nerve foramen; **VI**, abducens nerve foramen; **X**, vagus nerve foramen.

*Neurocranium* (Figs 26 and 27). Neurocranium divided into seven anatomical regions, as proposed by Compagno [41]: rostrum (RO), nasal capsules (NC), cranial roof (CR), basal plate (BP), orbits (OR), otic capsules (OC), and occiput (OCC).

The rostrum (RO), encompassing the rostral and adjacent cartilages, is very reduced in *Isistius* when compared to other squaliforms [3], carcharhiniforms [41], and lamniforms [351,352]. The medial rostral cartilage (**mro**) is a slender longitudinal elevation, weakly prolonged anterior to the neuroocranium; its posterior portion is immediately anterior to the anterior fontanelle (**af**), and is positioned slightly dorsal to the nasal capsule roof (**rnc**). In *I*. *brasiliensis* the medial rostral cartilage (**mro**) is only connected to the neurocranium at its posteriormost part, in between the nasal capsules (**nc**), but is supported by connective tissue throughout its length. In *I*. *plutodus* the medial rostral cartilage (**mro**) is even more reduced and does not project dorsally above the nasal capsules.

The nasal capsule (NC) encloses the olfactory organs in the nasal cavity; the nasal aperture is positioned ventrally and anteriorly in the capsule, allowing for the inflow and outflow of water within the capsules to stimulate each olfactory organ. The anterior wall of the olfactory cavity is dorsally rounded, forms the roof of the nasal capsule and joins the preorbital wall to form its posterior aspect. The nasal capsule is conjoined with the preorbital process and supraorbital crest (**soc**) forming the anterior internal orbital wall. The posterior nasal capsule wall has a ventrolateral process that forms an acute curved tip, the ectethmoid process (**eep**). Anterior and ventral openings of the nasal capsules slightly differ in size between both species of *Isistius*, at 7.67% and 7.91% of HDL in *I*. *brasiliensis* and *I*. *plutodus*, respectively.

Each nasal capsule has an irregular anteroventral opening accomodating the nasal cartilage (**nas**), an annular element that supports the nostril and nasal lobes, whose most posterior portions taper and form pointed ends that do not meet but internally encircle the nasal aperture. The nasal capsules comprise the ventral surface of the ethmoid region, and are separated by the internasal septum (**ins**), which forms the ectethmoid chambers (**ec**). The subethmoidal crest (**ser**) is a centrally positioned, ventrally elevated ridge that continues posteriorly to form the anterior segment of the interorbital wall. From the posteroventral portion of the nasal capsules, two cartilaginous pieces that are directed anteriorly arise; at their midlength they deflect toward the midline. Whereas in *I*. *plutodus* these cartilages do not meet, in *I*. *brasiliensis* the antimeres almost meet at midlength of the subethmoidal crest, dividing the ectethmoid chamber (**ec**) into anterior and posterior regions.

The anterior fontanelle (**af**) in *I*. *brasiliensis* is small and subrhomboidal, with its posterior margin more acute, and is covered by a tough and fibrous membrane. It is located at the anterior region of the cranial roof (**CR**), at the posterior extremity of the ethmoid region. In *I*. *plutodus* the anterior fontanelle is more circular and much smaller. The foramen of the epiphyseal organ (**ep**) is a perforation in the cranial roof posterior to the anterior fontanelle (**af**), and also has an oval shape, but is slender and shorter than the anterior fontanelle, especially in *I*. *brasiliensis*; in *I*. *plutodus* the epiphysial perforation is closer to the anterior fontanelle and there is a dorsal elevation between them, not present in *I*. *brasiliensis*. The precerebral fossa (**pcf**) opens immediately anterior to the anterior fontanelle and is occupied by a gelatinous mass. The nasal capsules laterally restrict this wedge-shaped shallow concavity. Anterior to the precerebral fossa is the medial rostral cartilage. In *I*. *brasiliensis* the foramen for the epiphyseal organ (**ep**) is posterior to the level of preorbital canals (**poc**), but in *I*. *plutodus* it is slightly anterior to them. In *I*. *brasiliensis*, the anterior fontanelle (**af**) is at the level of the ethmoid canal (**eca**) and is not only larger than the epiphyseal organ (**ep**) in this species, but is also larger than both foramina in *I*. *plutodus*.

The basal plate (**BP**) is a wide and flat surface on the ventral side of the otic region that posteriorly forms the palatine plate (**pal**). The carotid artery foramen (**fca**) is at the anterior portion of the palatine plate.

The orbital region (**OR**) occupies more than one-third of the neurocranium and medially supports the eye and serves as anchorage for ligaments associated with jaw suspension. The supraorbital crest (**soc**) is a lateral projection of the neurocranium at its dorsalmost aspect. Longitudinal grooves are present at crest midlength, and the preorbital canal (**poc**), for ramifications of the ophthalmicus superficialis and ophthalmicus profundus nerves, is present anteriorly. The ethmoid canal (**eca**), through which pass the ramus of ophthalmicus superficialis (of the ethmoidal nerve), is located more anteriorly, on the nasal capsule, and lateral to the preorbital canal (**poc**). Both canals are in the longitudinal grooves of the supraorbital crest (**soc**). The orbital anterolateral wall has a ventrolaterally directed ectethmoid process (**eep**). This process is more flattened dorsoventrally and is at a greater angle in *I*. *brasiliensis* than in *I*. *plutodus*, but in the latter it is more ventrolaterally pointed, and the orbital anterior wall is almost perpendicular to body axis.

The interorbital wall (**iow**) supports many foramina for cranial nerves and blood vessels. The optic nerve foramen (**II**) is located at the medial portion of this wall. The prootic foramen (**V-VII**, for the trigeminus and facialis nerves, except the ramus hyomandibularis of the facialis which is posterior to the lateral commissure) is located at the posterior border of the interorbital wall (**iow**); it is slightly more ventrally positioned in *I*. *plutodus*. The oculomotor (**III**) and trochlear (**IV**) nerves have their own foramina that are posterior and dorsal to the optic foramen (**II**), respectively; their positions differ among both *Isistius* species (Figs 23 and 24). The foramen of the abducens (**VI**) nerve is just anterior to the prootic (**V-VII**) foramen.

Both species have a membranous interorbital wall (**miow**) covering the large central opening, which, according to Shirai [3] is a synapomorphic feature of *Isistius*. This opening is proportionately greater in *I*. *plutodus*, as it almost reaches the neurocranial roof, the anterior orbital wall, and the ventral portion of the interorbital wall. As the opening is very wide in this species, the ventral interorbital wall is thinner than in *I*. *brasiliensis*. The optic pedicel is absent in *Isistius*.

The efferent pseudobranchial (**psb**) artery foramen, together with the ramus hyomandibularis of the facialis nerve, are positioned just posterior to the lateral commissure within a shallow depression. The lateral commissure (**com**) is at the posterior border of the orbital region and ventral to the postorbital process. An articular facet, the orbital notch (**np**), is located below and anterior to the basal angle (**ba**), which is a lateral process of the basicranium that supports the jaw medially. The basitrabecular process (**btp**) is a lateral expansion of the suborbital area that supports the ascending process of the palatoquadrate; it is laterally expanded to form a wide palatine surface.

The lateral auditory groove (**lag**) is a longitudinal groove at the lateral otic wall. The hyomandibular fossa (**hf**), to which the proximal end of the hyomandibula attaches, is located at the posterior end of the lateral otic wall. The posterior crest to the hyomandibular fossa is laterally expanded, and ventrally forms the postotic process (**potp**). The foramen for the orbital artery (**foa**) is on the lateral side of the otic capsule, below the postorbital groove in *I*. *brasiliensis*, but is more ventrally positioned in *I*. *plutodus*.

The otic capsules (**OC**) and occipital region (**OCC**) are the posterior portions of the neurocranium and lack clear divisions. The parietal fossa, a deep ovoid concavity, is at the posterior end of the supraotic region, between the otic capsules. There are two paired perforations within this concavity, the endolymphatic foramina (**fen**) and the perilymphatic fenestra (**fpn**), but as the endolymphatic fossa floor (**elf**) is wide open both the foramina and the fenestra go through it (**fen**+**fpn**). The parietal fossa is very similar in both *Isistius* species; however, in *I*. *brasiliensis* it is deeper. The opistotic ridge (**or**) is well developed and dorsolaterally expanded.

Immediately posterior to the parietal fossa, the neurocranial posterior wall is widely expanded and posteroventrally inclined. The foramen magnum is located in the medial portion of this wall. The basioccipital fovea is an articular facet ventral to the foramen magnum, and within it lies the occipital centrum articulated to the first vertebral centrum (axial articulation). Besides this articular facet, the occipital condyle is articulated to the basiventral processes of the first vertebra (co-lateral articulation). Foramina of the glossopharyngeal (**IX**) and vagus (**X**) nerves open in the posterior wall of the neurocranium; a foramen for a blood vessel is present dorsal to the vagus foramen, about one-third its size.

In relation to neurocranial morphometry, although *I*. *brasiliensis* has a wider distance across postorbital processes (**pop**) in terms of nasobasal length (49.46% *vs*. 41.50%), the length of the postorbital process is greater in *I*. *plutodus* (4.15 mm *vs*. 3.99 mm), even in a smaller specimen. The basitrabecular process (**btp**) is much greater and more pronounced in *I*. *brasiliensis* than in *I*. *plutodus*, as it is larger (width from left to right edges in terms of head length: 22.89% *vs*. 21.88%, respectively), and the angle from which it arises from the palatine plate (**pal**) is much wider. The basal angle (**ba**) is also different among these two species, as in *I*. *plutodus* its anterior portion is concave and it makes an abrupt turn towards the posteromedial portion of the neurocranium at the level of the foramen for the carotid artery (**fca**). In *I*. *brasiliensis*, the basal angle, instead of curved, is rectangular. The length of otic capsule is greater in *I*. *brasiliensis* in terms of nasobasal length (32.19% *vs*. 29.29%, respectively), and the hyomandibular facet (**hf**) in *I*. *plutodus* is closer to the foramina for the pseudobranchial artery (**psb**) and the hyomandibular branch of the facialis, as the otic capsule is reduced.

**Fig 28:**
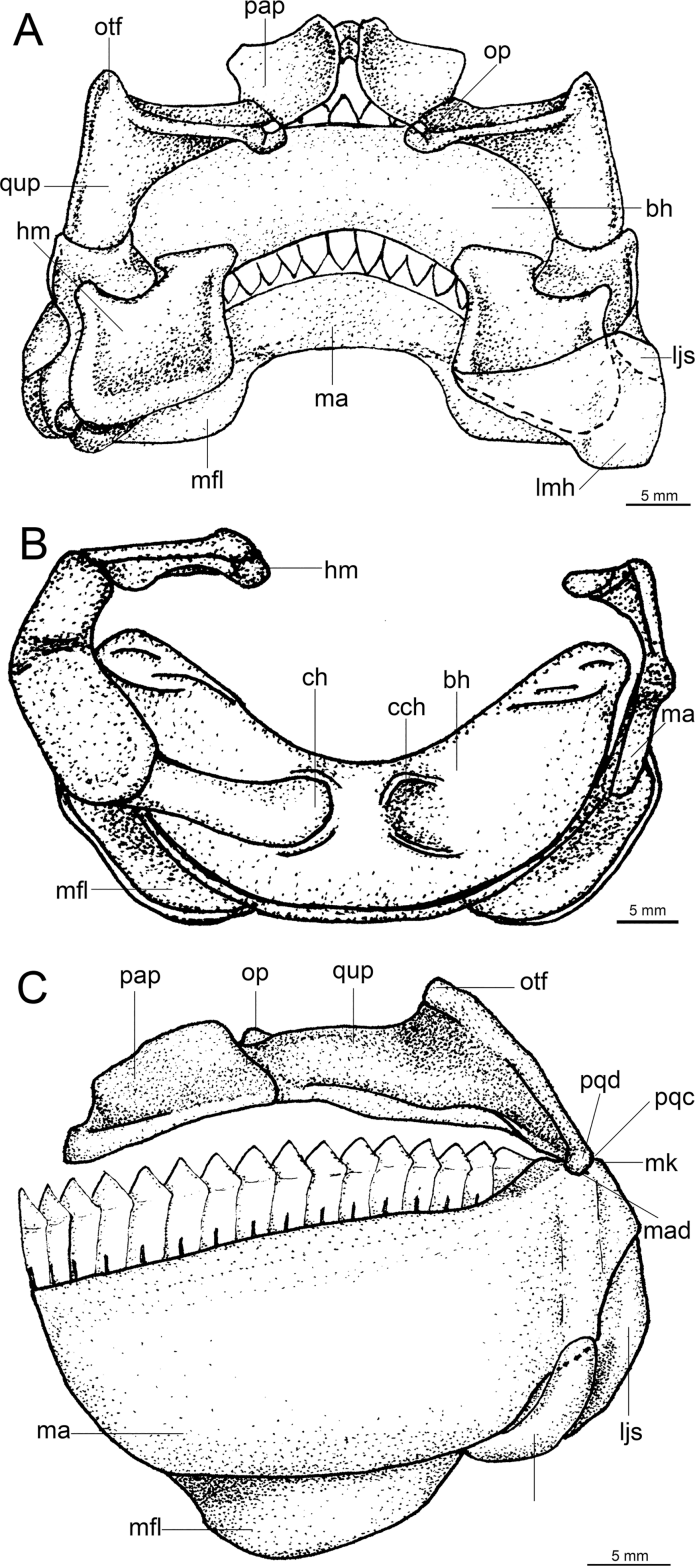
Mandibular and hyoid arches of *Isistius brasiliensis* (MZUSP 121506). (A) dorsal (anterior to top), (B) posterior (dorsal to top), and (C) left lateral views. Note **lmh** missing from left side in (A), and ceratohyal and hyomandibula missing from right side in (B). Abbreviations: **bh**, basihyal; **cch**, large concavities on ceratohyal; **ch**, ceratohyal; **hm**, hyomandibula; **lmh**, ligamentum mandibulo-hyoideum; **ma**, mandibula; **mad**, articular fossa; **mfl**, mandibular accessory cartilage; **mk**, mandibular knob; **op**, orbital process; **otf**, otic flange; **pap**, palatine plate; **pqc**, quadrate plate concavity; **pqd**, quadrate plate condyle; **qup**, quadrate plate.

**Fig 29:**
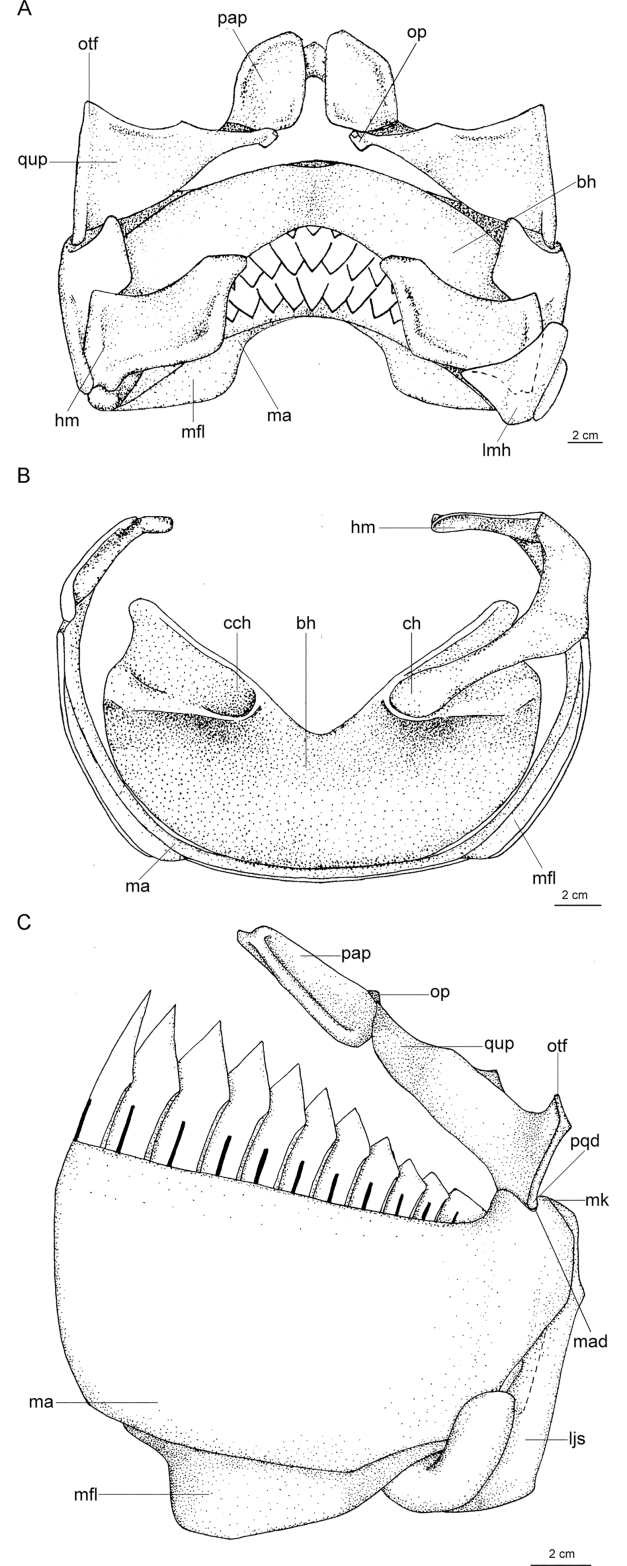
Mandibular and hyoid arches of *Isistius plutodus* (ZUEC 8333). (A) dorsal (anterior to top), (B) anterior (dorsal to top), and (C) left lateral views. Note **lmh** missing from left side in (A), and ceratohyal and hyomandibula missing from left side in (B). Abbreviations: **bh**, basihyal; **cch**, large concavities on ceratohyal; **ch**, ceratohyal; **hm**, hyomandibula; **lmh**, ligamentum mandibulo-hyoideum; **ma**, mandibula; **mad**, articular fossa; **mfl**, mandibular accessory cartilage; **mk**, mandibular knob; **op**, orbital process; **otf**, otic flange; **pap**, palatine plate; **pqc**, quadrate plate concavity; **pqd**, quadrate plate condyle; **qup**, quadrate plate.

*Mandibular arch* (Figs 28 and 29**)**. The palatoquadrate is divided into two components, the palatine plate (**pap**) and the quadrate plate (**qup**). The quadrate plate, near its junction to palatine, supports the orbital process (**op**) for the orbital articulation and the otic flange (**otf**) at its dorsalmost portion. The external (ventrolateral) surface of the quadrate plate is depressed and forms a concavity for the m. adductor mandibulae. The articular facet for the jaw-joint at the posterior end of the ventral edge has a large condyle (**pqd**) and a small quadrate concavity (**pqc**). The palatine plate of the palatoquadrate supports most of the upper tooth rows. In *I*. *brasiliensis*, the palatine plate in lateral view is somewhat trapezoidal, but its anterior portion is lower than its posterior; however, in *I*. *plutodus* this plate is more symmetrical. Dorsally, the quadrate plate has a straight dorsal edge in *I*. *brasiliensis*, and a slight protuberance at its midlength in *I*. *plutodus*.

Mandibular cartilage (**ma**) is much larger than both the palatine and quadrate plates together. It is divided into right and left antimeres and each has a flap-like accessory cartilage (**mfl**) made of weakly calcified tissue that is flexible and probably related to the peculiar feeding habit of *Isistius* [14]. This accessory cartilage is larger and longer in *I*. *plutodus* than in *I*. *brasiliensis*. Lower tooth rows occupy almost the whole lower jaw transverse length, and the only toothless portions are the most lateral where the mandibular cartilage articulates with the quadrate plate. At the posterodorsal corner, a large articular fossa (**mad**) receives the quadrate condyle (**pqd**). Immediately dorsal to it, a pronounced mandibular knob (**mk**) is evident, fitting into the small quadrate concavity (**pqc**). A well-developed ligament supports the mandibular knob and the hyoid arch. The mandible bears a process at its posteroventral edge, where there is an insertion for a thick ligament to the ceratohyal, the ligamentum mandibulo-hyoideum (**lmh**), which allows the wide movement of the basihyal. In *I*. *plutodus*, this ligament is thicker, in relation to jaw size, than in *I*. *brasiliensis*.

The labial cartilages support the upper lip and the corner between upper and lower lips. They are paired structures composed of two upper cartilages, the posterior (**plc**) and the anterior (**alc),** and a lower one (**llc**), which are connected to each other at the mouth corner. The lowest labial cartilage is the largest and extends from anterior to the mouth corner to almost the posterior end of mandibular cartilage, laying directly ventral to it. The upper anterior cartilage is transverse to body length; its distal portion is close to anteriormost part of the lower cartilage; the medial portion of both left and right **alc** overlap, which allows for a greater gape. The smallest labial cartilage, the upper posterior labial cartilage, is parallel and posterior to the **alc,** also supporting the upper lip.

Positioned dorsal to the quadrate cartilage and lateral to the neurocranium, immediately posterior to the postorbital process, is the minute spiracular cartilage (**spc**) that supports the spiracular filaments. The spiracular cartilage is flat and rectangular and vertically positioned, with a concave dorsal portion.

**Fig 30:**
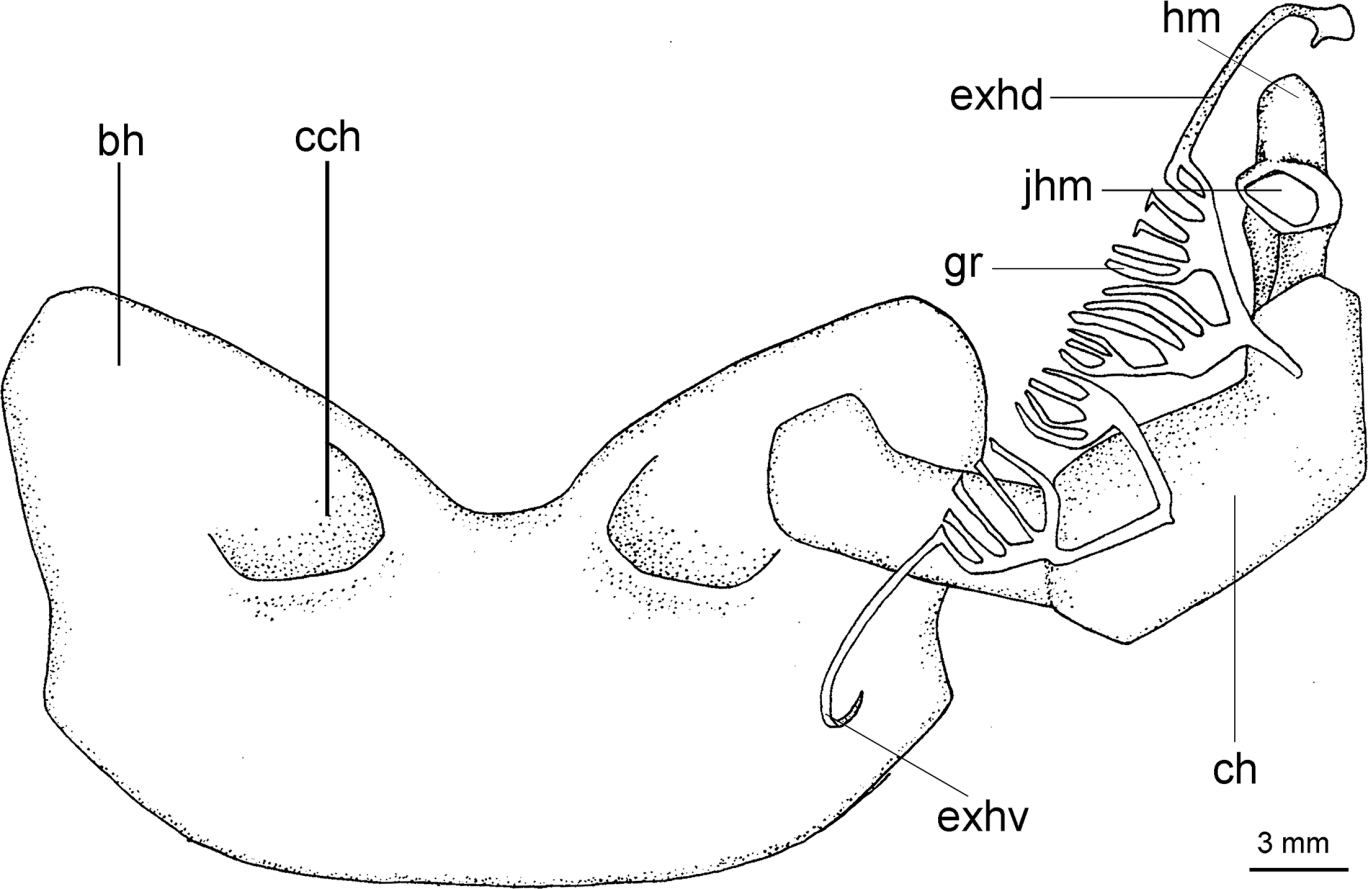
Hyoid arch of *Isistius brasiliensis* (MZUSP 121506). Posterior view (dorsal to top). Ceratohyal cartilage displaced from its fossa at the basihyal for illustration purposes. Abbreviations: **bh**, basihyal; **cch**, large concavities on ceratohyal; **ch**, ceratohyal; **exb**, extrabranchial cartilage of branquial arches; **exhd**, dorsal extrabranchial cartilage of hyoid arch; **exhv**, ventral extrabranchial cartilage of hyoid arch; **gr**, gill rays; **hm**, hyomandibula; **jhm**, joint cartilage of hyomandibula.

*Hyoid arch* (Fig 30**)**. The hyoid arch supports the lower jaws at their posterointernal corners and is composed of the paired hyomandibula and ceratohyal cartilages and the single median basihyal cartilage. The hyomandibula (**hm**) is the dorsal element of the arch and joins with the neurocranium at the hyomandibular fossa (**hf**) on the auditory capsule. It is rectangular with depressions and protuberances; its longest length is transverse to body length. Even though this cartilage is very similar between *I*. *brasiliensis* and *I*. *plutodus* there are some differences (Figs 25A and 26A). The upper margin of the hyomandibula (**hm**) has a posteromedial depression or concavity, but its medial and posterior corners are elevated. Both elevations are more pronounced in *I*. *brasiliensis*. At the posterior corner a very small, flat and oval cartilage, the joint cartilage of hyomandibula (**jhm**), is present. This posterodorsal projection of the hyomandibula (**hm**) articulates with the neurocranium at the hyomandibular fossa (**hf**) together with the joint cartilage of hyomandibula (**jhm**) at the posterior corner. The anterior region of the hyomandibula (**hm**) articulates with the mandibular knob (**mk**).

The ceratohyal (**ch**) is an elongate, subcylindrical and angled element. It is more robust in *I*. *brasiliensis* than in *I*. *plutodus*, in which it is longer and more slender. Its posterodorsal portion is slightly rounded, articulating to the lateroanterior corner of the hyomandibula (**hm**) through the ligamentum hyomandibulo-hyoideum (**lhc**). The posterior segment of the ceratohyal is subrectangular to cylindrical; its anterior portion is more deflected toward the midline and is more cylindrical and slender. Its anterior end is somewhat triangular where it articulates with the basihyal (**bh**); its posteroventral margin is expanded for the interhyoideus muscle. Immediately posterior to the ligamentum hyomandibulo-hyoideum (**lhc**) there is another ligament that unites the lateral corner of the lower jaw with the ceratohyal: the ligamentum mandibulo-hyoideum (**lmh**). These two tissues form a unique ligament joining the posterolateral corner of the hyomandibula (**hm**), the posterodorsal region of the ceratohyal (**ch**) and the lateral corner of the lower jaw.

The basihyal (**bh**) is a massive, unpaired element supporting the anterior floor of the oral cavity. It is very wide, wider than long and positioned transversally to body axis. The anterior margin of the basihyal is convex and its posterior margin concave or angular. In *I*. *plutodus*, the ventral concavity has a smaller angle and is anteroposteriorly longer than in *I*. *brasiliensis*. It has two large concavities (**cch**), one at each side of posterior surface for the joints with the ceratohyals (**ch**), which are positioned more posteriorly in *I*. *plutodus* than in *I*. *brasiliensis*.

The gill rays (**gr**) are elongated and supported by the ceratohyal (**ch**). There are two bundles of gill rays (**gr**) with separate origins on the posterior side of each ceratohyal (**ch**), and both distally subdivide into more than ten individual rays. The extrabranchial cartilages (**exb**) on the hyoid arch are slender, ray-like elements that extend dorsally and ventrally from the gill rays; extrabranchials proximally slightly more broad and tapering distally, and support the hyoidean hemibranch. The dorsal hyoid extrabranchial cartilage (**exhd**) is fused to the dorsalmost ramification of the gill rays, while the ventral hyoid extrabranchial cartilage (**exhv**) is fused to the ventralmost ramification of the gill rays.

**Fig 31:**
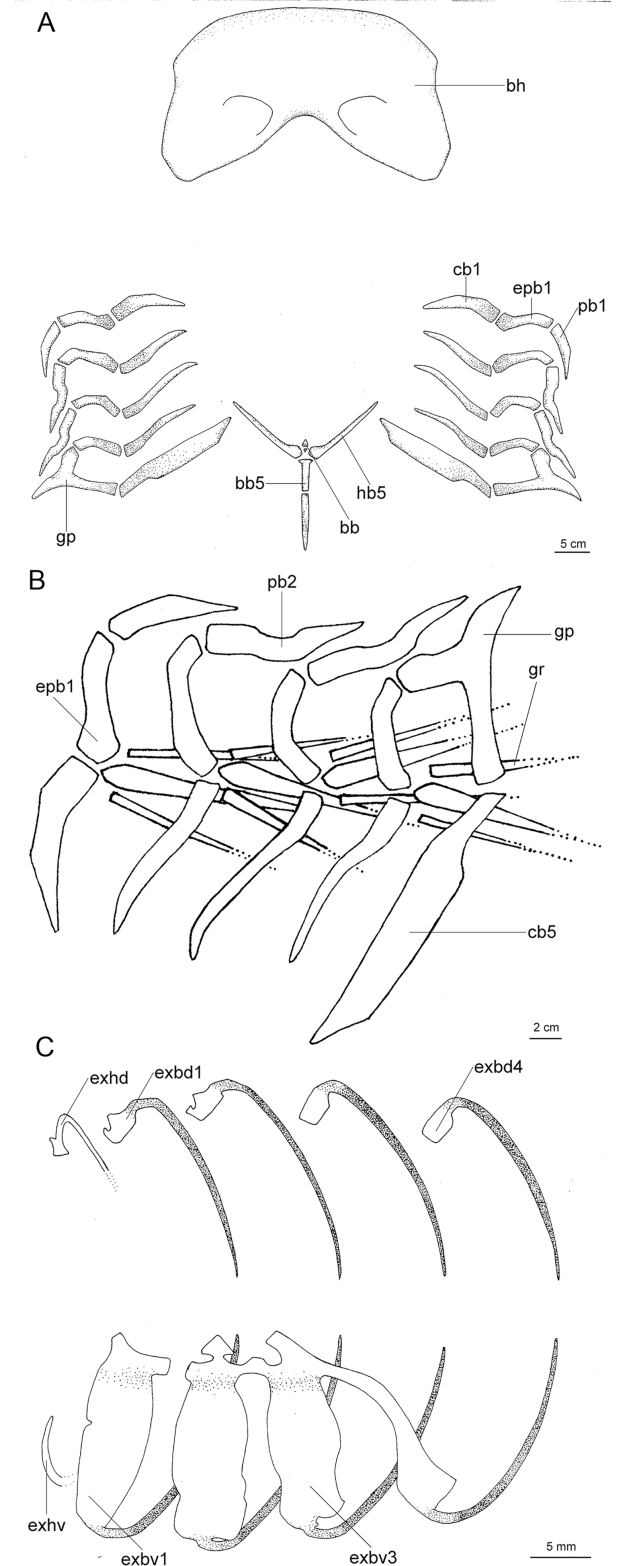
Branchial arches of *Isisitus brasiliensis* (MNHN 1996–0465). (A) ventral view; (B) internal view of right branchial arches pulled open; (C) internal view of right extrabranchial cartilages. Anterior to top in (A) and anterior to left in (B) and (C). Distance between basihyal cartilage and branchial arches in scale. Abbreviations: **bb**, basibranchial; **bb5**, fifth basibranchial; **bh**, basihyal; **cb1**, first ceratobranchial; **cb5**, fifth ceratobranchial; **epb1**, first epibranchial; **exbd1**, first dorsal extrabranchial cartilage of branquial arches; **exbd4**, fourth dorsal extrabranchial cartilage of branquial arches; **exbv1**, first ventral extrabranchial cartilage of branquial arches; **exbv3**, third ventral extrabranchial cartilage of branquial arches; **exhd**, dorsal extrabranchial cartilage of hyoid arch; **exhv**, ventral extrabranchial cartilage of hyoid arch; **gp**, gill pickax; **gr**, gill rays; **hb5**, fifth hypobranchial; **pb1**, first pharyngobranchial; **pb2**, second pharyngobranchial.

**Fig 32:**
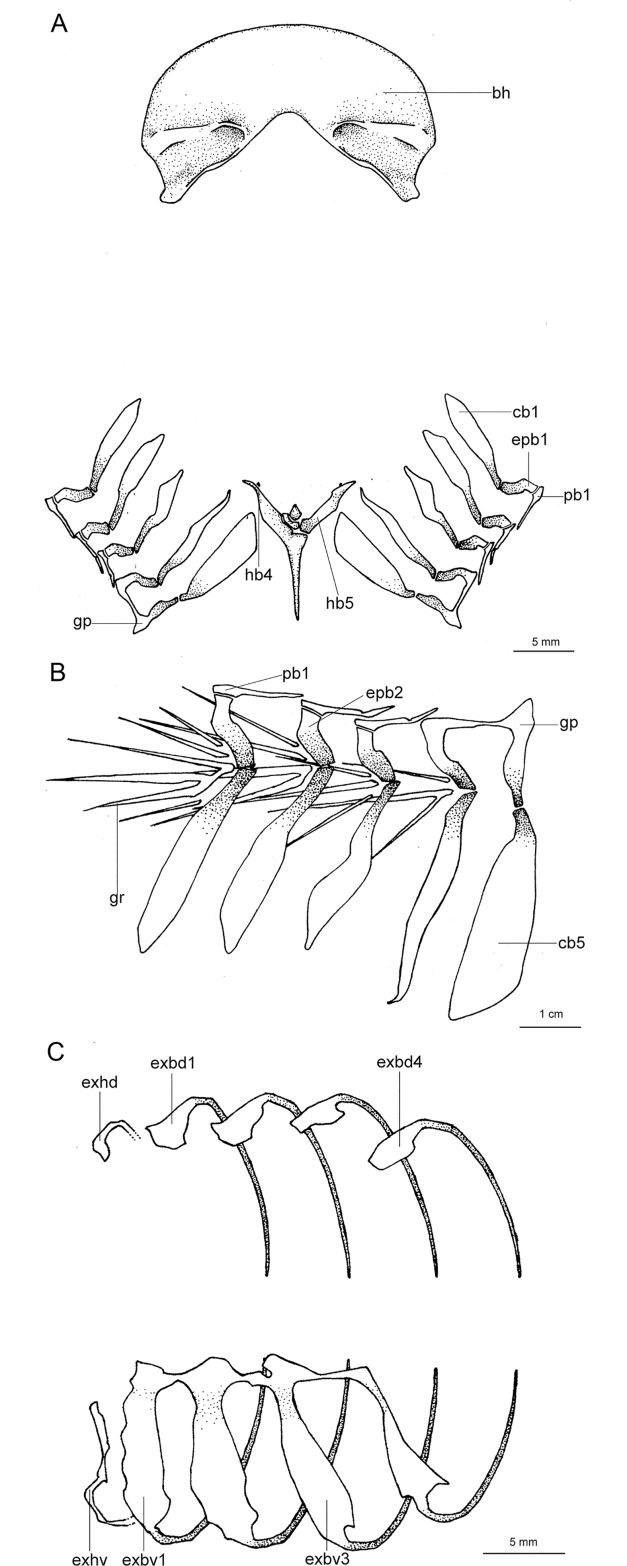
Branchial arches of *Isisitus plutodus* (ZUEC 8333). (A) ventral view; (B) internal view of right branchial arches pulled open; (C) internal view of right extrabranchial cartilages. Anterior to top in (A) and anterior to left in (B) and (C). Distance between basihyal cartilage and branchial arches in scale. Abbreviations: **bb**, basibranchial; **bb5**, fifth basibranchial; **bh**, basihyal; **cb1**, first ceratobranchial; **cb5**, fifth ceratobranchial; **epb1**, first epibranchial; **exbd1**, first dorsal extrabranchial cartilage of branquial arches; **exbd4**, fourth dorsal extrabranchial cartilage of branquial arches; **exbv1**, first ventral extrabranchial cartilage of branquial arches; **exbv3**, third ventral extrabranchial cartilage of branquial arches; **exhd**, dorsal extrabranchial cartilage of hyoid arch; **exhv**, ventral extrabranchial cartilage of hyoid arch; **gp**, gill pickax; **gr**, gill rays; **hb5**, fifth hypobranchial; **pb1**, first pharyngobranchial; **pb2**, second pharyngobranchial.

*Branchial Arches* (Figs 31 and 32**)**. From dorsal to ventral regions, each branchial arch is composed of pharyngobranchial (**pb**), epibranchial (**epb**), ceratobranchial (**cb**), hypobranchial (**hb**), and basibranchial (**bb**) cartilages, the latter being the only branchial cartilages which are not paired and positioned in the midventral portion of the branchial basket. In the examined specimen of *I*. *plutodus*, the branchial arches were less symmetrical, as left and right structures may present different patterns and differed in the number of cartilages, such as observed for the hypo- and basibranchials. The pharyngobranchials (**pb**) extend posteromedially form their articulation with epibranchials, and taper distally. Phrayngobranchials in more central and posterior arches are slightly concave on dorsal margins in *I*. *brasiliensis*. Pharyngobranchials in *I*. *brasiliensis* are more robust than those in *I*. *plutodus*. In the left side of analyzed specimens of *I*. *brasiliensis* (USNM 215948, MNHN 1996–0465, MZUSP 121506) pharyngobranchial 5 (**pb5**) is united with epibranchial 5 (**epb5**) and pharyngobranchial 4 (**pb4**) forming the gill pickax (**gp**) [3]; however, the gill pickax (**gp**) on the right side is formed by the fusion of four elements: **pb5**, **epb5**, **pb4**, and **epb4**. In the only dissected specimen of *I*. *plutodus*, both right and left **gp** structures are identical to the right side of *I*. *brasiliensis*. Therefore, Shirai's [3] hypothesis that the gill pickax is formed by four elements in *Isistius* is only partially true.

The epibranchial cartilages (**epb**) are distally articulated with proximal ends of pharyngobranchials, and proximally articulated with the distal portion of respective ceratobranchials. Epibranchials are slender, vertically positioned elements with slightly concave posterior margins, and are slightly shorter in *I*. *plutodus*. The ceratobranchials (**cb**) are long, thick, and flat cartilages, somewhat stouter in *I*. *plutodus* than in *I*. *brasiliensis*. They form the ventrolateral wall of the pharynx and carry gill filaments, as do epibranchials. The proximal portion of ceratobranchials, where they articulate with the epibranchials, is relatively straight; however, they terminate distally in more acute angles. The ceratobranchials of *I*. *plutodus* are wider distally than those of *I*. *brasiliensis*. The posterior ceratobranchial is greater than the anterior elements in both species. Also in both species, ceratobranchial 4 is the most slender ceratobranchial element.

In *I*. *brasiliensis* there is only one pair of hypobranchials (**hb**), which are positioned in a V-like arrangement, with the opening directed anteriorly. They do not articulate directly to each other. The hypobranchials are almost cylindrical, elongated, and their proximal ends are wider, tapering distally. Because the distal region of ceratobranchials 5 (**cb5**) reaches the distal region of these hypobranchials, they are here identified as hypobranchials 5 (**hb5**). In *I*. *plutodus*, the hypobranchials are also in a V-like position; however, this structure is not symmetrical because there are more elements on the left side. Similarly to *I*. *brasiliensis*, in *I*. *plutodus* ceratobranchials 5 (**cb5**) reaches hypobranchials 5 (**hb5**), as do ceratobranchials 4 (**cb4**). At the medial side of the distal regions of these hypobranchials are two other very small cartilages. Since they are in symmetrical positions at the midventral portion of the branchial basket (but not identical in shape), and they are paired cartilages, they are identified as hypobranchials 4 (**hb4**). Therefore, *I*. *plutodus* has two pairs of hypobranchials to which are connected the fourth (**cb4**) and fifth (**cb5**) ceratobranchials. Hypobranchials 5 (**hb5**) are similar in shape to those observed in *I*. *brasiliensis*; hypobranchials 4 (**hb4**) are oval. At the left side of the V-like structure there is another cartilage that is very closely connected to hypobranchial 5 (**hb5**). It does not have any symmetrical element, nor is in a medial position. Therefore, it could be considered a malformation of the left element of hypobranchial 5 (**hb5**).

The basibranchials (**bb**) are unpaired elements positioned dorsal to the heart region. There are two distinguishable single cartilages posterior to hypobranchial 5 (**hb5**) in *I*. *brasiliensis*, and only one in *I*. *plutodus*, which seems to be somewhat fused to the left hypobranchial 5 (**hb5**). The most anterior of these cartilages is shorter and truncated posteriorly; its anterior portion is wider and each corner is connected to hypobranchial 5 (**hb5**). The second cartilage in *I*. *brasiliensis* seems to be continuous to the anterior one, as its anterior part has the same width as the posterior ending of the anterior element. The posterior element is longer than the anterior and tapers posteriorly to end in a point. Anterior to these two basibranchial cartilages, and between both rounded proximal portions of hypobranchials 5 (**hb5**), are two very small and poorly calcified cartilages in both species. Both are triangular, but the anterior one is greater and is pointed anteriorly. The base of the posterior triangular element is parallel to the base of the anterior element and tapers posteriorly. These two elements, although weakly calcified and very small, could also be basibranchials, as they are single cartilages positioned in the mid-ventral region of the branchial basket.

Gill rays (**gr**) are slender cartilages supported by the anterior four pairs of epibranchials and ceratobranchials, and there are from three to six rays in each branchial arch, being usually more numerous in *I*. *plutodus*. Gill rakers are absent in *Isistius*, but extrabranchial cartilages (**exb**) are present on all arches. These paired elements externally support the branchial basket and are divided into dorsal and ventral elements; they are present in the hyoid arch and in branchial arches 1 to 4. The dorsal extrabranchial cartilages 1 (**exbd1**), 2 (**exbd2**), 3 (**exbd3**), and 4 (**exbd4**) are very similar to each other in both *Isistius* species, with broad proximal portions and tapering distally forming elongate cylindrical cartilages. The ventral extrabranchial cartilages 1 (**exbv1**), 2 (**exbv2**), 3 (**exbv3**), and 4 (**exbv4**) are proximally very broad and subrectangular; their dorsalmost portions are fused in *I*. *plutodus*, but **exbv1** is not fused in *I*. *brasiliensis*. These cartilages are proximally very flattened, but cylindrical and slender along most of their lengths, poorly calcified, and almost meet the dorsal extrabranchials.

**Fig 33:**
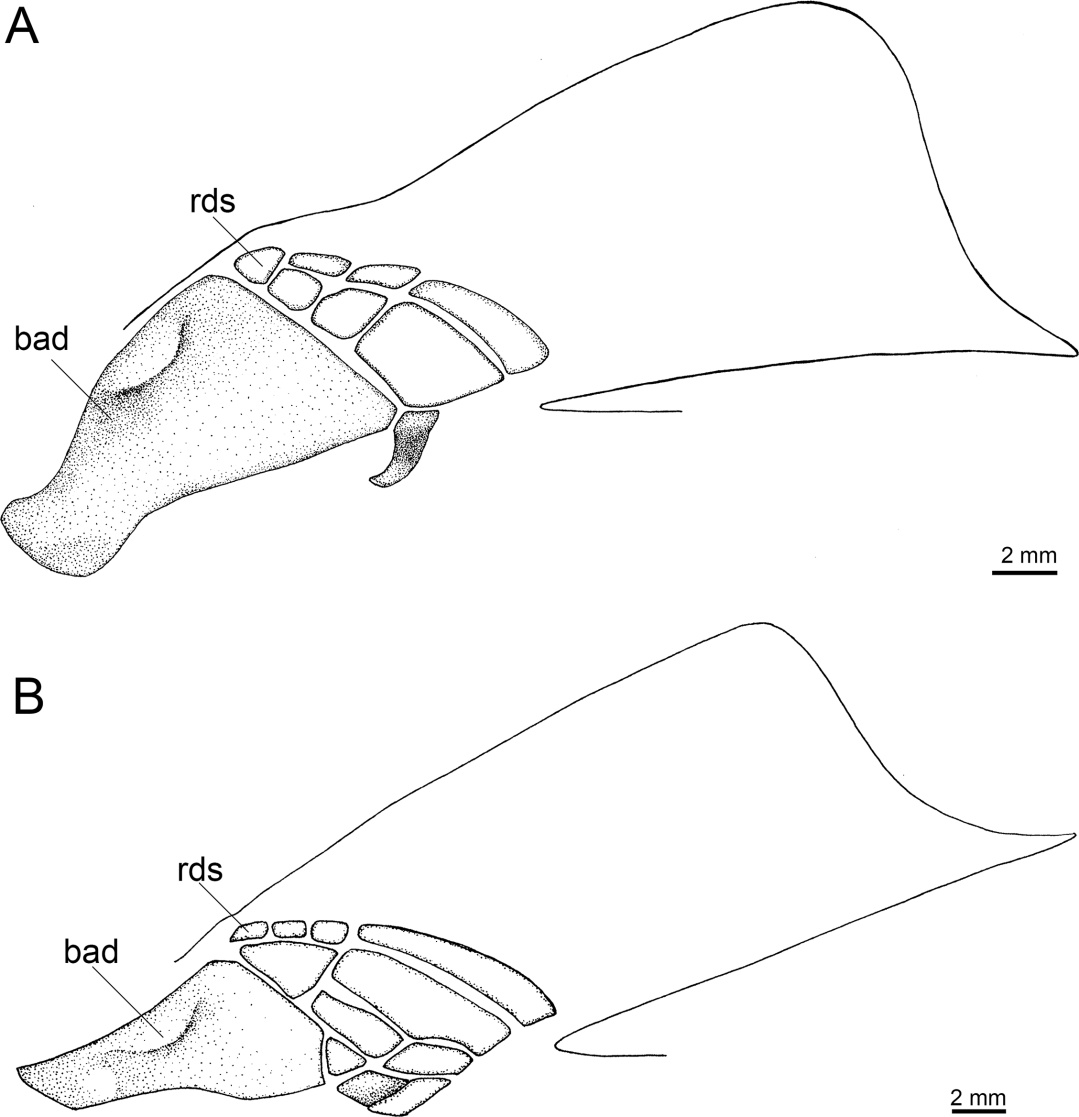
Dorsal fins of *Isistius brasiliensis* (MNHN 1996–0465). (A) first dorsal; (B) second dorsal fin. Anterior to left. Abbreviations: **bad**, basal cartilage of dorsal fin; **rds**, radials.

**Fig 34:**
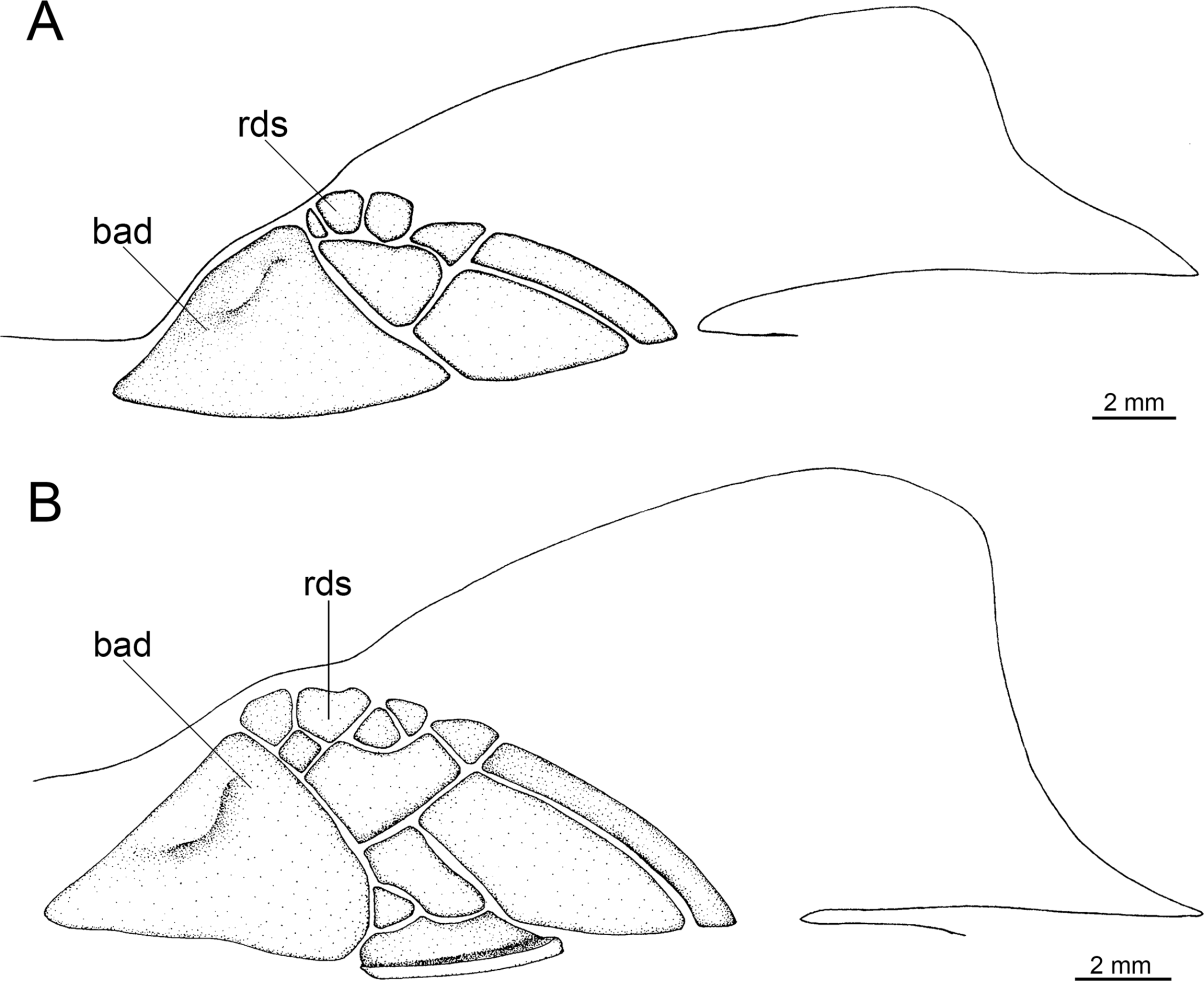
Dorsal fins of *Isistius plutodus* (ZUEC 8333). (A) first dorsal; (B) second dorsal fin. Anterior to left. Abbreviations: **bad**, basal cartilage of dorsal fin; **rds**, radials.

*Dorsal fins* (Figs 33 and 34). Basal cartilage (**bad**) and multiple radial elements (**rds**) aplesodic, not reaching fin margin. Fin spine absent. Basal cartilage detached from vertebral column, more slender anteriorly and broad posteriorly, and deeply inserted in body. In *I*. *brasiliensis*, the anteriormost portion of basal cartilage is slightly dorsally directed; the basal element of the first dorsal fin is trapezoidal, hourglass-shaped, with the posterior portion much broader and longer than the anterior one; the basal of the second dorsal fin is narrower. In *I*. *plutodus*, the basal cartilage is triangular in both dorsal fins, with the anterior region anteriorly pointed. In *I*. *brasiliensis*, the first dorsal has five to nine radials, and second dorsal has nine to 12 radials; in *I*. *plutodus* there are seven radials in the first dorsal fin, and 12 in the second, all with different sizes and shapes, as mentioned by Benzer [353] for species of *Squalus*. In both dorsal fins the radials are distinct from each other; however, there is always a slender, curved, half-moon shaped radial at the posterodorsal aspect of the dorsal fin skeleton in both species. A small and perpendicular cartilage to the fin’s axis is sometimes present in *I*. *brasiliensis*, posterior to the basal element. In the second dorsal, the base of this perpendicular cartilage is the same size of one radial in *I*. *brasiliensis*, whereas it is the size of two radials in *I*. *plutodus*.

**Fig 35:**
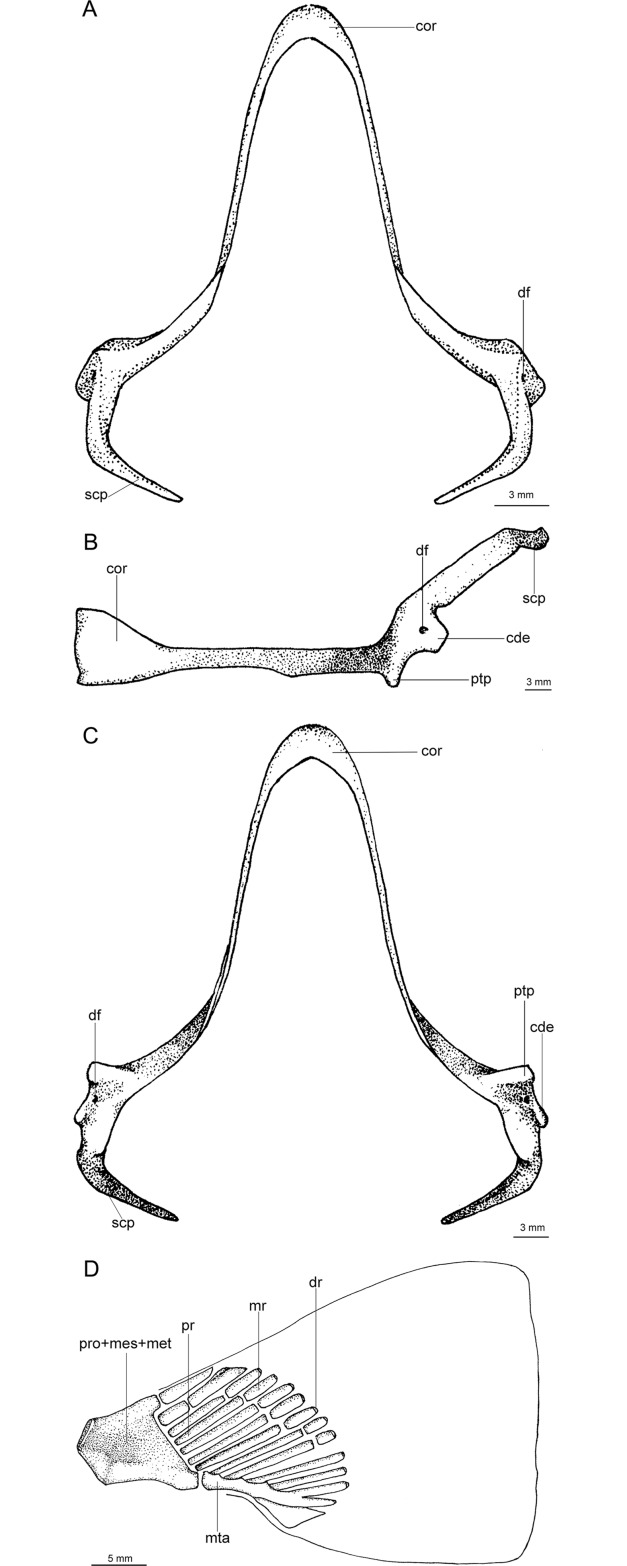
**Pectoral girdle (A, B, C) and left pectoral fin (D) of *Isistius brasiliensis* (MNHN 1996–0465).** (A) dorsal view (anterior to top); (B) lateral view (dorsal region to top); (C) ventral view (anterior to top); (D) left pectoral fin in lateral view (anterior to left). Abbreviations: **cde**, pectoral condyle; **cor**, coracoid bar; **df**, diazonal foramen; **dr**, distal radial; **mr**, medial radial; **mta**, metapterygial axis; **ptp**, posterior triangular process of coracoid bar; **pr**, proximal radial; **pro+mes+met**, fused propterygium, mesopterygium, and metapterygium; **scp**, scapular process.

**Fig 36:**
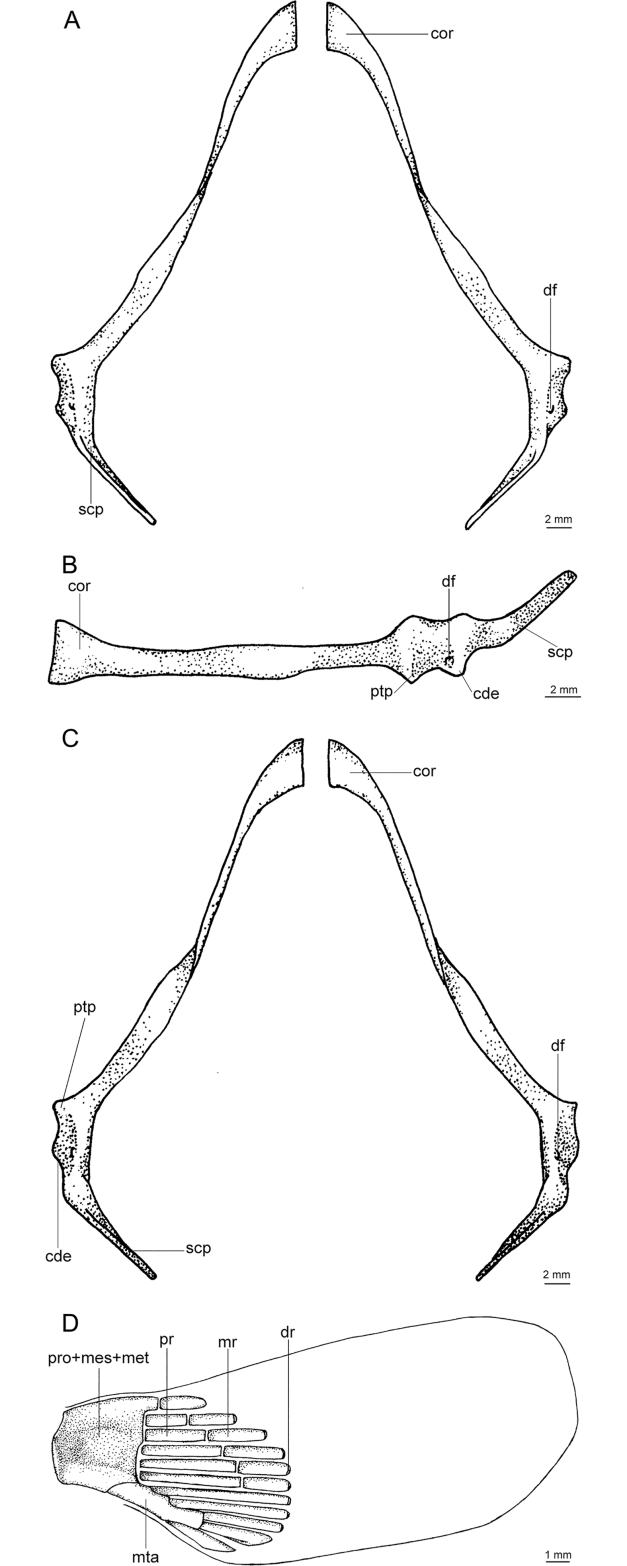
**Pectoral girdle (A, B, C) and left pectoral fin (D) of *Isistius plutodus* (ZUEC 8333).** (A) dorsal view (anterior to top); (B) lateral view (dorsal region to top); (C) ventral view (anterior to top); (D) left pectoral fin in lateral view (anterior to right). Abbreviations: **cde**, pectoral condyle; **cor**, coracoid bar; **df**, diazonal foramen; **dr**, distal radial; **mr**, medial radial; **mta**, metapterygial axis; **ptp**, posterior triangular process of coracoid bar; **pr**, proximal radial; **pro+mes+met**, fused propterygium, mesopterygium, and metapterygium; **scp**, scapular process.

*Pectoral girdle and fins* (Figs 35 and 36). The pectoral girdle (scapulocoracoid), which is deeply inserted in the hypaxial musculature, is a single elongate cartilage in *I*. *brasiliensis* but in *I*. *plutodus* the scapulocoracoid is formed by paired cartilages that meet medially. In both species, the coracoid bar (**cor**) has an inverted U- or V-shape in dorsoventral view, widens posteriorly near the scapulae (more so in *I*. *plutodus*), and deflects medially at the level of the suprascapulae. In lateral view, the coracoid bar is much taller anteromedially and is very narrow posteriorly toward the pectoral condyles (**cde**); the coracoid is laterally flattened. The coracoid bar is more robust and taller anteromedially in *I*. *brasiliensis* compared to *I*. *plutodus*. The coracoid is a very narrow structure from its anteriormost region to the pectoral condyles (**cde**). The scapular region is relatively small but taller than the lateral aspect of the coracoid bar; in *I*. *plutodus* it is more level with the lateral coracoid bar, but in *I*. *brasiliensis* it projects posterodorsally. The posterior triangular process of coracoid bar (**ptp**) is a ventral triangular projection anterior to the pectoral condyles (**cde**), where the pectoral fin basal and associated musculature attach to scapula. A single diazonal foramen (**df**) is present in between the triangular process and the pectoral condyles. In *I*. *plutodus* the dorsal margin of the scapula bears two broadly triangular protuberances. The scapular process (**scp**), at the most posterior part of the scapula, tapers posteriorly and is directed posterodorsally and toward the midline. In *I*. *plutodus*, this process is longer and slightly more slender than in *I*. *brasiliensis*.

The pectoral fin skeleton is aplesodic and composed of basal and radial cartilages. In *Isistius*, there is only one basal cartilage, which may be composed of the fusion of the propterygium, mesopterygium, and metapterygium (**pro+mes+met**). The basal element is trapezoidal in *I*. *brasiliensis* but more square in *I*. *plutodus*. There are, approximately, seven proximal radials (**pr)**, each with a medial radial (**mr**) except the first dorsal radial. Each medial radial supports a distal radial (**dr**) except the first dorsal medial radial. The metapterygial axis (**mta**) is a posteroventral cartilaginous piece that is more elongate than the proximal radials; it is longer in *I*. *plutodus* compared to *I*. *brasiliensis*. This cartilage supports a variable number of proximal, medial, and distal radials; the number of pectoral radials varies among specimens.

**Fig 37:**
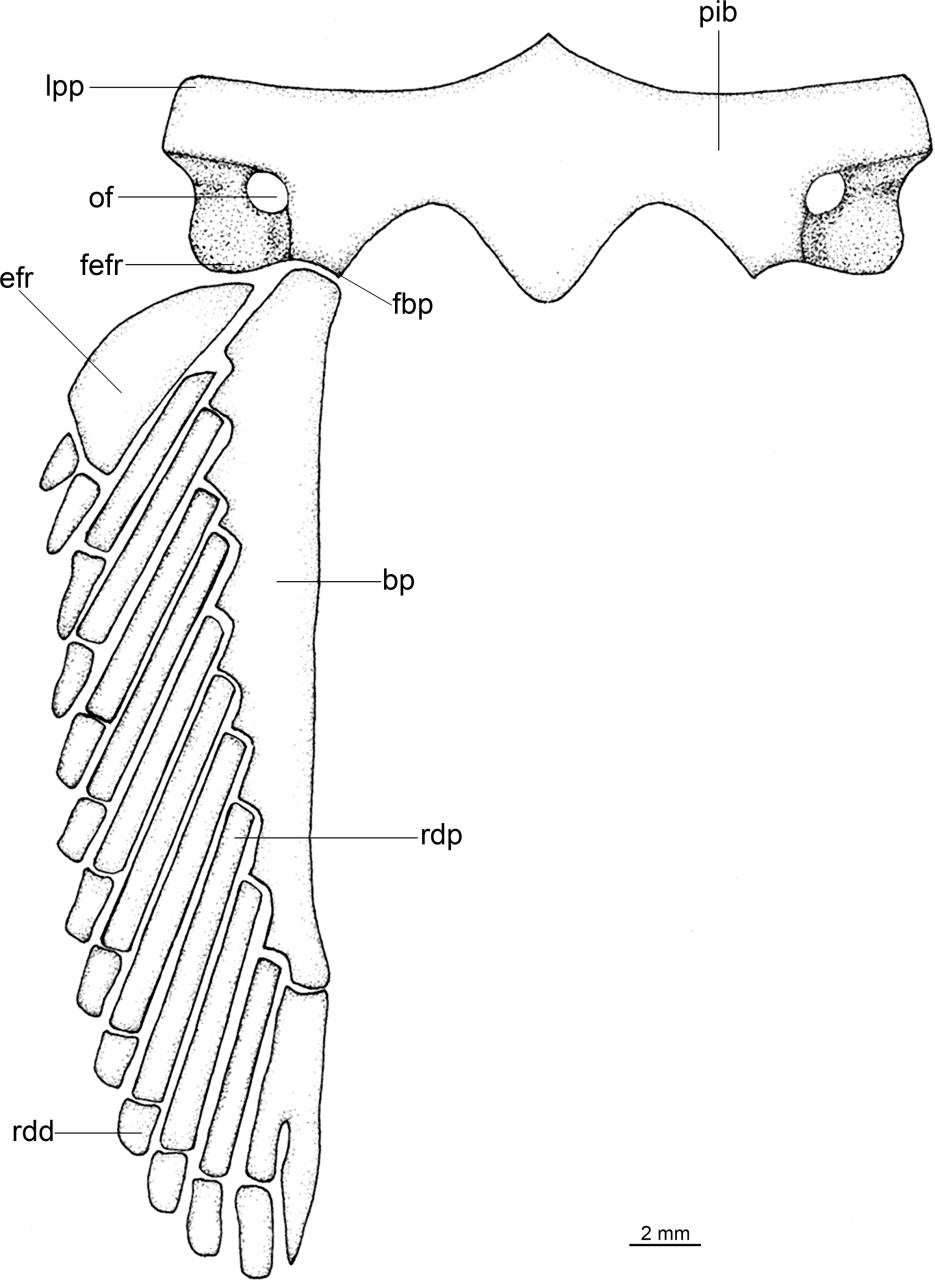
Pelvic girdle and right pelvic fin of a female specimen *Isistius brasiliensis* (MNHN 1996–0465). Ventral view. Abbreviations: **bp**, pelvic basipterygium; **efr**, enlarged first radial; **fbp**, basipterygium facet; **fefr**, first enlarged radial; **lpp**, lateral prepelvic process; **of**, obturator foramina; **pib**, puboischiadic bar; **rdd**, distal radial; **rdp**, proximal radial.

**Fig 38:**
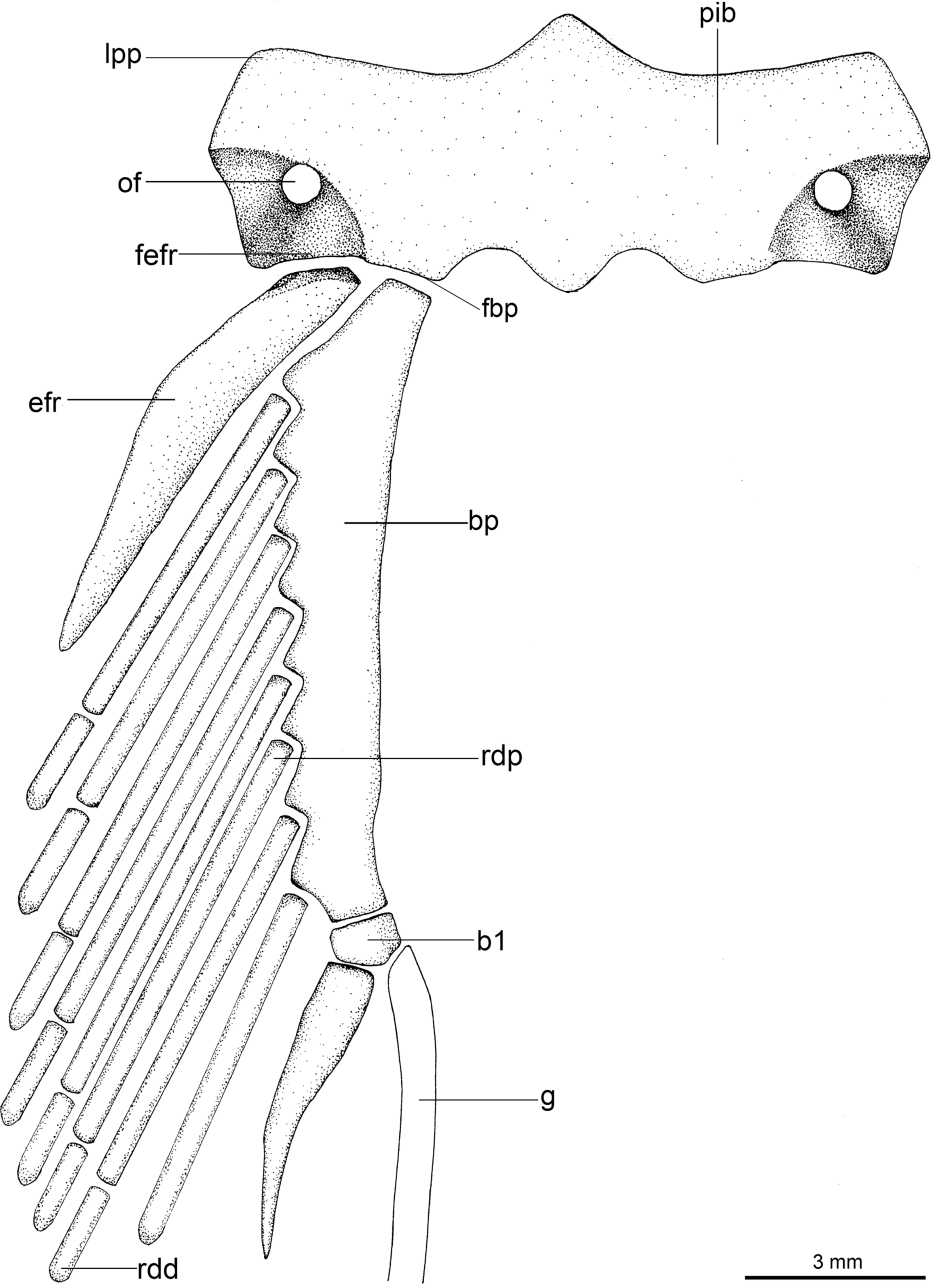
Pelvic girdle and right pelvic fin of a male specimen *Isistius plutodus* (ZUEC 8333). Ventral view. Abbreviations: **b1**, intermediate segment; **bp**, pelvic basipterygium; **efr**, enlarged first radial; **fbp**, basipterygium facet; **fefr**, first enlarged radial; **g**, end-style; **lpp**, lateral prepelvic process; **of**, obturator foramina; **pib**, puboischiadic bar; **rdd**, distal radial; **rdp**, proximal radial.

*Pelvic girdle and fins* (Figs 37 and 38). The puboischiadic bar (**pib**), the only cartilage that forms the pelvic girdle, is a short, relatively straight element transverse to body length. A central, triangular projection, more broad and pronounced in *I*. *plutodus* than in *I*. *brasiliensis*, is present on its anterior margin; lateral to the medial projection the anterior margin of the girdle is slightly concave. The posterior puboischiadic margin also bears a similar medial projection, bordered by an acute depression on either side that are much more pronounced and deeper in *I*. *brasiliensis* than in *I*. *plutodus*. The puboischiadic bar in *I*. *brasiliensis* is anteroposteriorly more slender in comparison to *I*. *plutodus*. The lateral prepelvic process (**lpp**) is a lateral projection of the puboischiadic bar just anterior to the pelvic fin radials. The obturator foramen (**of**) is a proportionally large and circular opening. The posterolateral corners of the puboischiadic bar accomodate two articular surfaces for the pelvic fin skeleton. The lateral surface articulates with the first enlarged radial (**fefr**), and the medial articular surface is for the basipterygium (**fbp**). The former surface is much more rounded in *I*. *brasiliensis* than in *I*. *plutodus*.

The pelvic fin skeleton comprises the pelvic basipterygium (**bp**), an anteroposteriorly elongated cartilage that supports the distal radials. Specimens usually have from 11 to 13 radials (**rds**). The anteriormost element is the triangular enlarged first radial (**efr**), which is short and broad and articulates to two short and parallel triangular distal radials in *I brasiliensis*, but is more elongate and does not articulate to distal radials in *I*. *plutodus*. The posteriormost radial in females is enlarged, has a wide proximal portion and is distally divided into two radial pieces that might also have a distal radial each. Each proximal pelvic radial is long and slender and supports a smaller segment, a rectangular distal radial. The proximal and distal pelvic radials are proportionately longer in *I*. *plutodus* than in *I*. *brasiliensis*. In males of both *Isistius* species, the posteriormost radial is not bifurcated as in females, and is supported by the intermediate clasper cartilage (**b1**) instead of the basipterygium (**bp**); this radial is somewhat flattened, proximally wide and curved toward the clasper, almost reaching the axial cartilage. No female specimen of *I*. *plutodus* was examined in order to verify if the posterior bifurcated pelvic radial is present; however, as this difference was observed in *I*. *brasiliensis*, it might be present in *I*. *plutodus*. In males, the basipterygium (**bp**) distally supports the clasper skeleton.

**Fig 39:**
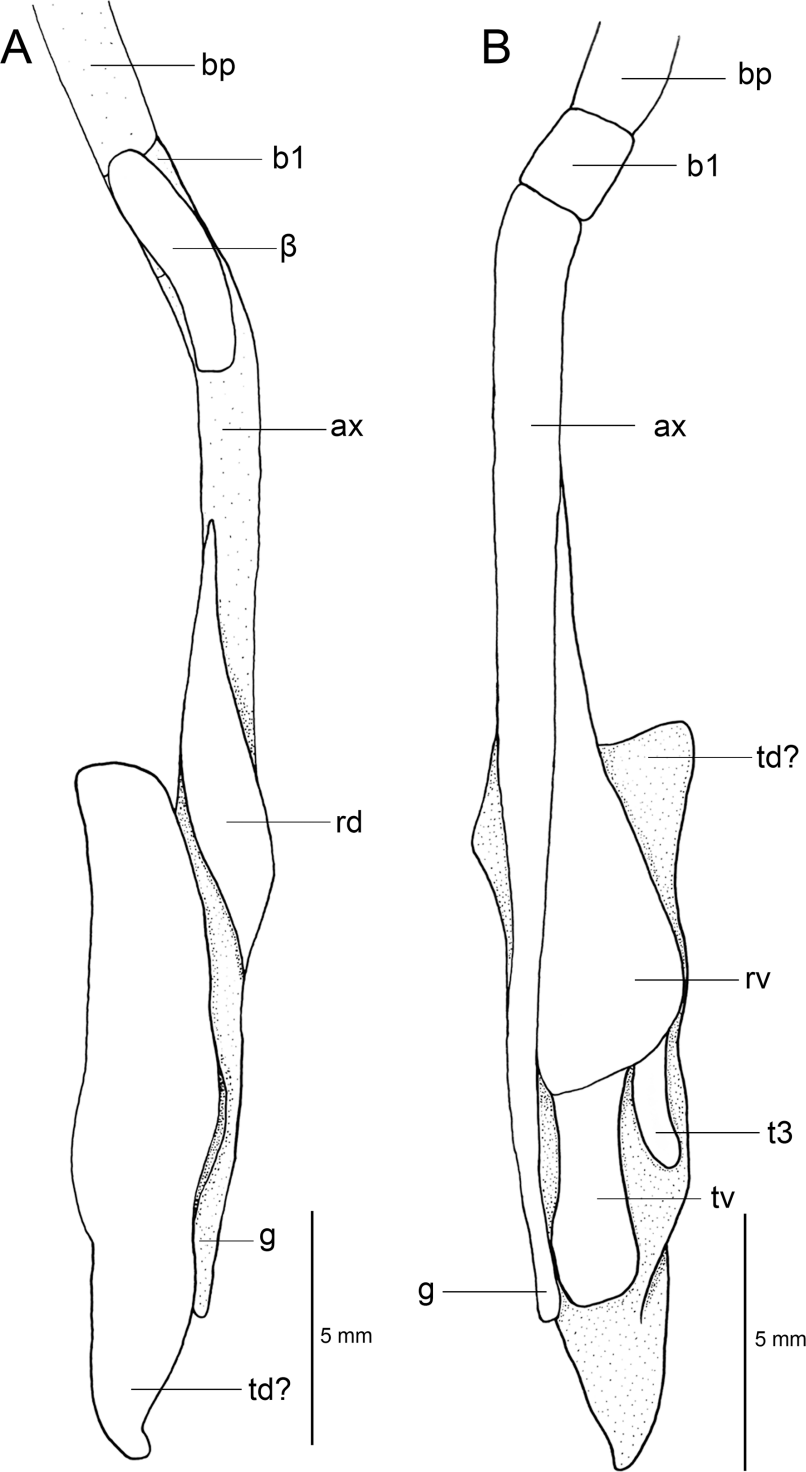
Left clasper skeleton of an adult specimen of *Isistius brasiliensis* (MNHN 1996–0465). **Dorsal (A) and ventral (B) views.** Abbreviations: **ax**, axial; **b1**, intermediate segment; **bp**, pelvic basipterygium; **g**, end-style; **rd**, dorsal marginal; **rv**, ventral marginal; **t3**, accessory terminal; **td?**, dorsal terminal?; **tv**, ventral terminal; β, beta cartilage.

*Claspers* (Fig 39). Nine cartilages form the clasper skeleton. In ventral view, the anteriormost cartilage is the single intermediate segment (**b1**) followed by the axial (**ax**) cartilage, which extends almost to the end of the clasper, where it becomes the less calcified, thinner and pointed end-style (**g**). The axial cartilage tapers toward the end-style at approximately its midlength; it is oblique at its lateral aspect but its medial margin is straight. The ventral marginal cartilage (**rv**) is triangular, very slender and pointed anteriorly and much wider posteriorly; it is a flattened cartilage attached laterally to the axial. The rectangular and flat ventral terminal cartilage (**tv**) is posterior to the ventral marginal cartilage, and its medial margin contacts the end-style. The dorsal terminal 3 cartilage (**t3**) is at the posterolateral region of the ventral marginal cartilage, positioned laterally to the ventral terminal cartilage; it is stout and short, with a wide and slightly ventrally curved distal portion.

In dorsal view, the beta cartilage (**β**) is rectangular and slightly curved, positioned dorsally to the intermediate segment; it is connected to the intermediate segment and the axial cartilage. The dorsal marginal cartilage (**rd**) is anteroposteriorly slender, wider at its midlength, and partially wraps the axial cartilage; its posterior margin reaches the end-style. A cartilage tentatively identified as the dorsal terminal (**td?**) is very large and occupies most of the clasper glans and about one-half of clasper length. Its anterior segment is lateral to the dorsal marginal cartilage. This cartilage is anteriorly rectangular and both sides are more or less parallel along their length, but taper at the level of the end-style. Its medial margin is dorsal to the axial cartilage and end-style, and borders the sperm duct together with the dorsal marginal cartilage.

**Fig 40:**
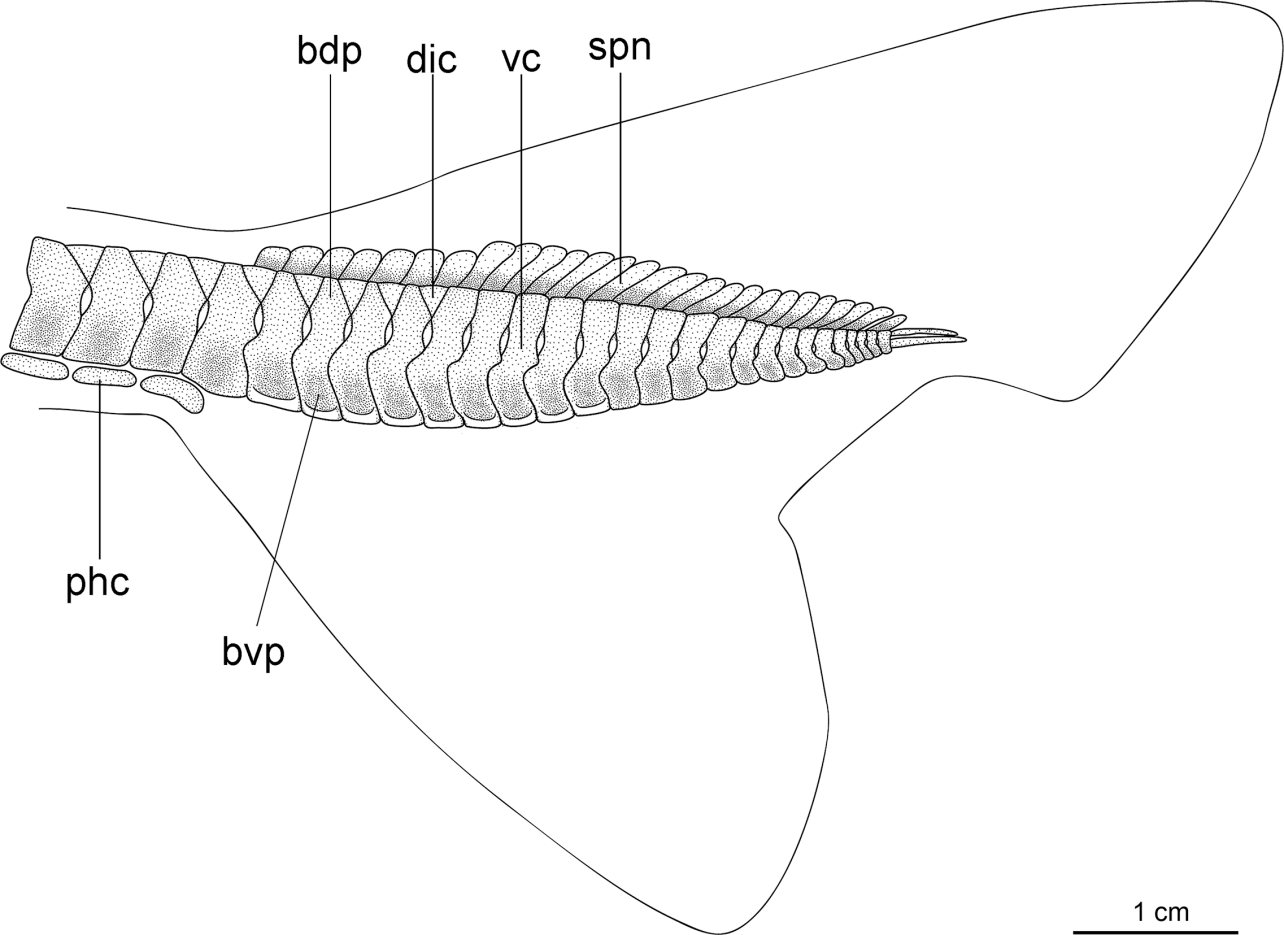
Left view of caudal fin skeleton of a specimen of *Isistius brasiliensis* (MNHN 1996–0465). Abbreviations: **bdp**, basidorsal element; **bvp**, basiventral elements; **dic**, interdorsal elements; **phc**, prehypochordal; **spn**, supraneural elements; **vc**, vertebral centra.

**Fig 41:**
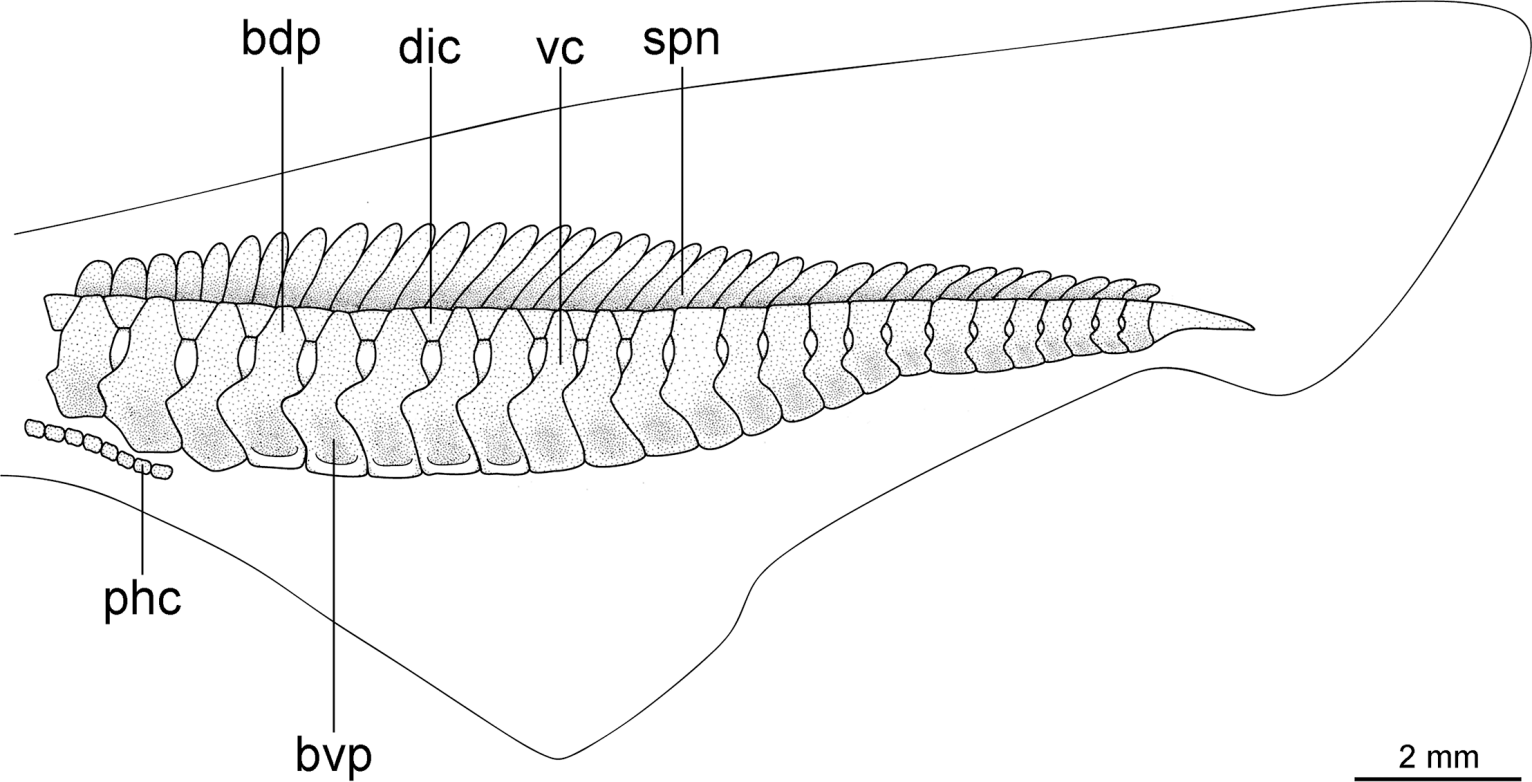
Left view of caudal fin skeleton of a specimen of *Isistius plutodus* (ZUEC 8333). Abbreviations: **bdp**, basidorsal element; **bvp**, basiventral elements; **dic**, interdorsal elements; **phc**, prehypochordal; **spn**, supraneural elements; **vc**, vertebral centra.

*Caudal fin* (Figs 40 and 41). Vertebral centra (**vc**) are fused with a basidorsal element (**bdp**) above each centrum and ventrally with a basiventral element (**bvp**). In between basidorsals there is an interdorsal element (**dic**) present until the middle of the caudal fin. Vertebral centra (**vc**) are cylindrical, but the basidorsals have two distict shapes, either triangular where interdorsals are present, or rhomboidal where there are no interdorsals. The interdorsals are triangular with their bases dorsally positioned. Supraneural spines (**spn**) are positioned above the basidorsals and interdorsals; these are posteriorly directed, elongate cartilages. There are, approximately, 30 supraneural spines, which are not correlated one-to-one to basidorsals and interdorsals. The seven anteriormost supraneural spines in *I*. *brasiliensis* are not as elongate as the more posterior spines; in *I*. *plutodus* the anterior supraneural spines are dorsally more rounded. The ventral basiventrals (**bvp**) result from the fusion of haemal arches and haemal spines; approximately 25 basiventrals are present. The size of the vertebral centra, basidorsals, basiventrals and haemal spines progressively decreases towards the posterior end of the caudal fin. At the posteriormost end of the caudal fin there is an elongated cartilage, which could be the last dorsal and ventral cartilages fused with the vertebral centrum or their complete absence; this cartilage is usually subdivided in *I*. *brasiliensis*. A small, ventrally positioned lateral projection is present from the most anterior basiventral to the middle of the caudal fin. Prehypochordal cartilages (**phc**) are present anterior to the basiventrals, which are more numerous and much smaller in *I*. *plutodus* than in *I*. *brasiliensis* (10 *vs*. 4, respectively); these cartilages are irregular in shape, either cylindrical, square, rectangular, or triangular. In *I*. *plutodus* the vertebral centra are more narrow than in *I*. *brasiliensis*, and there are more interdorsals and supraneural spines (approx. 36 *vs*. 27 in I. brasiliensis). There are also fewer haemal spines with a lateral protuberance at their ventral portion (approximately 5 *vs*. 10 in *I*. *brasiliensis*).

#### Musculature

*Eye muscles*. The external oculomotor muscles comprise four rectus and two oblique muscles. The rectus muscles originate from the posterior portion of the orbit and insert on the eyeball. These muscles are the *rectus superior*, *rectus internus*, *rectus inferior*, and *rectus externus*. The *rectus superior* inserts on the upper portion of the eyeball. The *rectus internus* and *rectus externus* originate at opposite sides of the neurocranium, but both at the posterior region of preorbital wall; they insert on the posterior portion of the eyeball: the *internus* on the upper portion and the *externus* on the lower one. The *rectus inferior* is opposite to the *superior* at its origin and insertion, as it inserts on the most ventral portion of the eyeball. The oblique muscles are *obliquus superior* and *obliquus inferior*. These muscles originate on the postorbital wall, anteriorly and opposite to the rectus muscles. Their origin is close to each other, but the *obliquus superior* inserts on the upper side of the eyeball, while the *obliquus inferior* inserts on the lower side.

**Fig 42:**
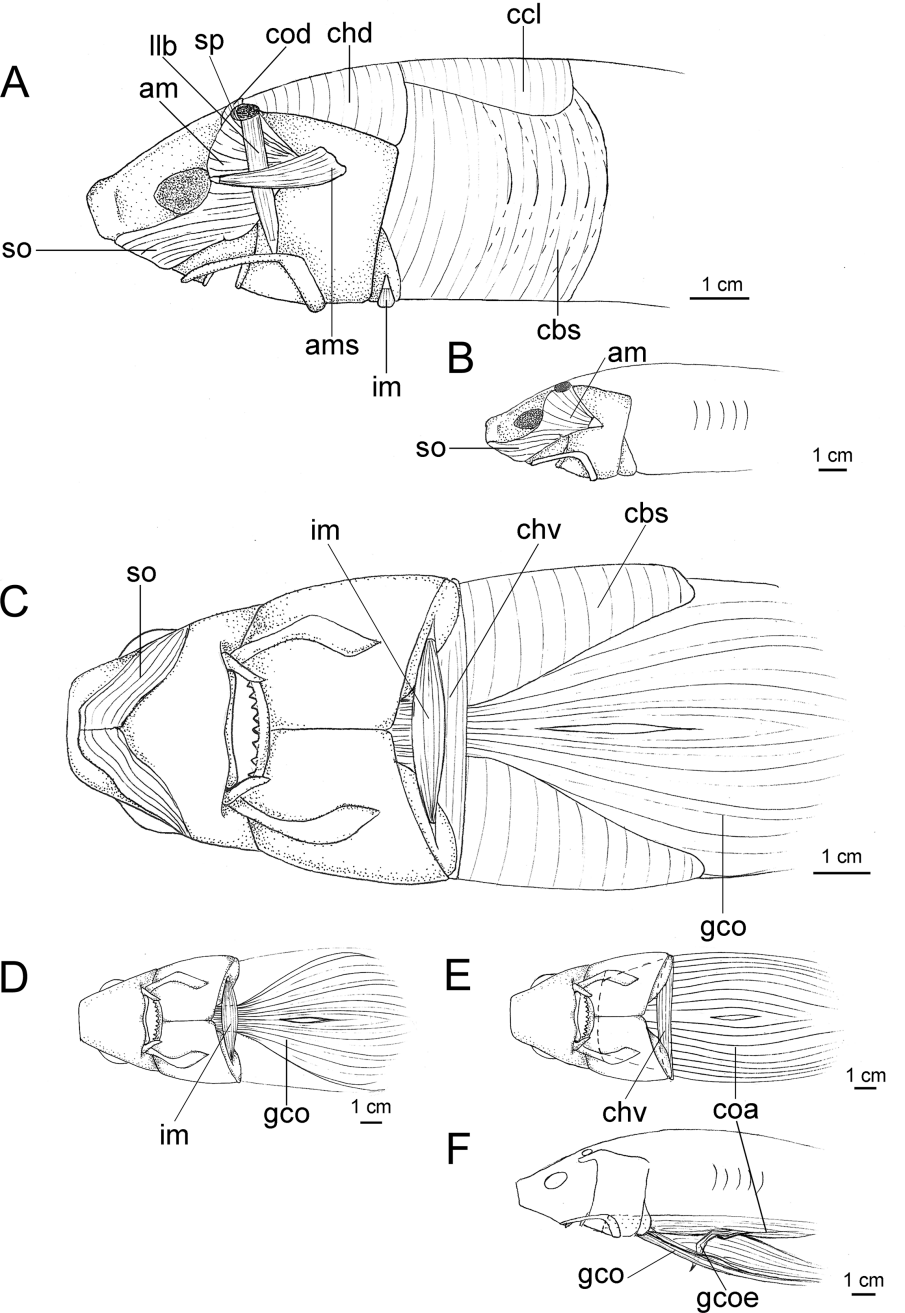
Mandibular, hyoid and partial hypobranchial musculature of *Isistius brasiliensis* (HUMZ 211104). (A) lateral view; (B) lateral view of *adductor mandibulae* and *suborbitalis* muscles in detail; (C) ventral view; (D, E) ventral views of *intermandibularis*, *genio-coracoideus*, *coraco-hyoideus ventralis*, and *coraco-arculalis* muscles in detail; (F) lateral view showing the muscle bundle *genio-coracoideus externus*. Abbreviations: **am**, adductor mandibulae; **ams**, adductor mandibulae superficialis; **cbs**, constrictors branchiales superficialis; **ccl**, cucullaris; **chd**, constrictor hyoideus dorsalis; **chv**, constrictor hyoideus ventralis; **coa**, coraco-arcualis; **cod**, constrictor dorsalis; **gco**, genio-coracoideus; **gcoe**, genio-coracoideus externus; **im**, intermandibularis; **llb**, levator labialis; **so**, suborbitalis; **sp**, siphon of clasper.

**Fig 43:**
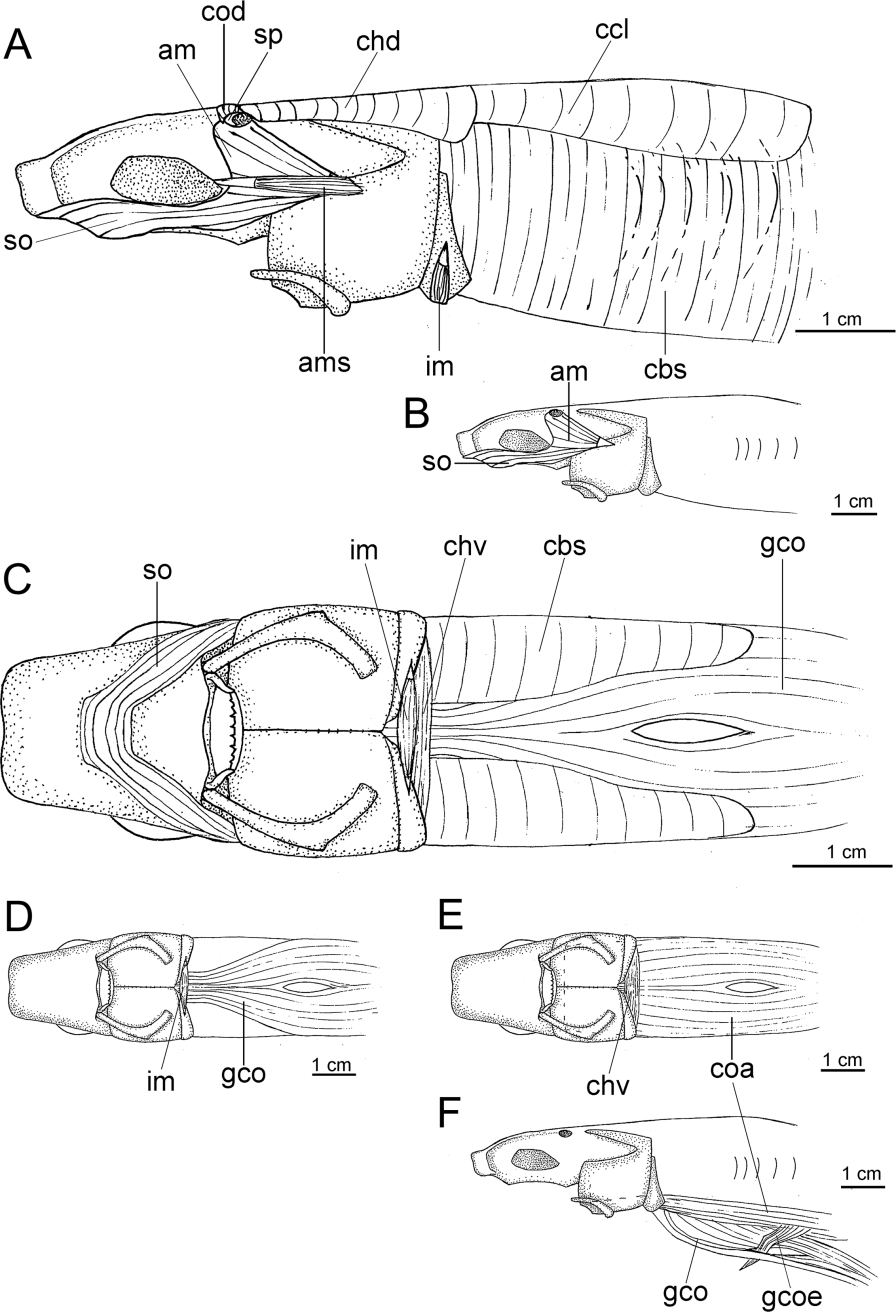
Mandibular, hyoid and partial hypobranchial musculature of *Isistius plutodus* (ZUEC 8333). (A) lateral view; (B) lateral view of *adductor mandibulae* and *suborbitalis* muscles in detail; (C) ventral view; (D, E) ventral views of *intermandibularis*, *genio-coracoideus*, *coraco-hyoideus ventralis*, and *coraco-arculalis* muscles in detail; (F) lateral view showing the muscle bundle *genio-coracoideus externus*. Abbreviations: **am**, adductor mandibulae; **ams**, adductor mandibulae superficialis; **cbs**, constrictors branchiales superficialis; **ccl**, cucullaris; **chd**, constrictor hyoideus dorsalis; **chv**, constrictor hyoideus ventralis; **coa**, coraco-arcualis; **cod**, constrictor dorsalis; **gco**, genio-coracoideus; **gcoe**, genio-coracoideus externus; **im**, intermandibularis; **llb**, levator labialis; **so**, suborbitalis; **sp**, siphon of clasper.

*Mandibular arch muscles* (Figs 42 and 43). The *adductor mandibulae superficialis* (**ams**) is the most superficial muscle; it is closely associated to the posterior portion of the *adductor mandibulae* (**am**). But, in *Isistius*, these two muscles are completely separated from each other and the **ams** is positioned on top of the **am**. The **ams** originates on mandibular cartilage and inserts through a tendon on the postorbital process of the neurocranium. The **ams** is a slender muscle and is positioned anteroposteriorly, parallel to the head axis. The **am** is located beneath the **ams** at the upper portion of the jaw; its origin is on the posterior portion of the postorbital process. The **am** has a dorsal and a ventral mass separated by a septum; however, these two portions are not totally separated from each other and are not considered two distinct muscles. The **am** passes in between the neurocranium and quadrate cartilages, right behind and slightly underneath the eye, and inserts with a tendon on mandibular cartilage. The **am** is a thick muscle that connects with the *suborbitalis* (**so**). It is difficult to separate these two muscles, as the latter originates on the subethmoidal crest (**ser**), runs along it and under the eyeball, and inserts on soft tissue on the upper lip. However, it forms a muscular continuum with the ***am*** that goes from the most anterior portion of the subethmoidal crest to the upper lateral wall of the neurocranium. The *levator labialis* (**llb**) is a slender, vertical muscle, positioned ventral to the **ams** and dorsal to the **am**; it is lacking in *I*. *plutodus*. Its origin is on the upper portion of the quadrate cartilage and its insertion is on the connective tissue on the outer side of the lower labial cartilage. Its origin is wide, almost the size of the outer side of the palatine cartilage, and tapers toward its insertion. The suborbitalis (**so**) is well developed, wrapping around the anteroventral snout region to meet its antimere medially; it is inserted via a tendon onto the adductor mandibulae.

*Hyoid arch muscles* (Figs 42 and 43). The dorsal and ventral sheets of the *constrictor hyoideus* (**chd** and **chv**) are associated to the hyomandibula and ceratohyal directly, and continue posteriorly to the *constrictor branchiales superficialis* (**cbs**). The *constrictor hyoideus dorsalis* (**chd**) is a short muscle with fibers perpendicular to the body axis. It is very wide and extends from the posterior spiracular region to just beyond the end of the upper jaw. Its origin is on the dorsolateral wall of the epaxial musculature, and has three points of insertion: on the posterior wall of the spiracle (forming its wall); in between the articulation of the dorsal margins of the mandibular, quadrate and hyomandibular cartilages, forming a triangle; and on the dorsolateral, posterior corner of the hyomandibula. The *constrictor hyoideus ventralis* (**chv**) is a ventral muscle that is separated from the *intermandibularis* (**im**), which is ventral to it, by a thin fascia. Almost its whole length is ventrally covered by ampullae of Lorenzini and connective tissue. This muscle originates from a seam of connective tissue at midventral line and inserts on the ventroposterior edge of the ceratohyal. The *constrictor dorsalis* (**cod**) is anterior to the constrictor hyoideus dorsalis (**chd**), and posterior to the *adductor mandibulae* (**am**) between the orbit and the spiracle; its origin is on the upper lateral wall of the neurocranium.

*Branchial constrictors* (Figs 42 and 43). The muscles *constrictor branchiales superficialis* (**cbs**) are five units, each covering externally and anteriorly a different branchial arch and the hyoid arch. As there is too much ampullae of Lorenzini and connective tissues, it is challenging to tear each bundle apart from the others. The anterior portion of these muscles originates on the dorsolateral wall of the epaxial musculature and the posterior portion on the *cucullaris* (**ccl**). They are located posteriorly to the *constrictor hyoideus dorsalis* (**chd**), in almost a continuum, and they are inserted on connective tissues on the ventrolateral side of the body that do not reach the midline; it is from this connective tissue mass that the *constrictor hyoideus ventralis* (**chv**) originates.

*Hypobranchial spinal musculature* (Figs 42 and 43). These muscles are located beneath the basibranchial cartilages. The *genio-coracoideus* (**gco**) is a thick and narrow muscle that strongly inserts on the mandibular symphysis, but becomes a thin and wide muscular sheet towards its posterior portion; its left and right sheets are eventually separated posteriorly by connective tissue; together with a fascia, the *genio-coracoideus* originates on the posterior portion of the *rectus cervicis*. Its origin is at the line of the pectoral fins and its most lateral portion is connected to the muscles at fin base. The *genio-coracoideus externus* (**gcoe**) is a paired muscle slip that arises from the anterolateral surface of the coracoid. Both right and left slips meet at the anteriormost portion of the coracoid and extend ventrally, converging on the *genio-coracoideus* (**gco**).

The *rectus cervicis* is muscle with two consecutive sheets dorsal to the *genio-coracoideus* (**gco**). Both portions are divided by a transverse septum that separates the muscle into an anterior part, the *coraco-hyoideus* (**coh**), and a posterior part, the *coraco-arcualis* (**coa**). The *coraco-hyoideus* (**coh**) is a wide and thick muscle that inserts on the posteroventral surface of the basihyal cartilage, and is covered ventrally by a thick sheet of connective tissue. Just anterior to the level of the coracoid is the septum that divides the muscle longitudinally, where the *coraco-arcualis* (**coa**) originates. Below the coracoid there is a fascia that passes along both sides of the *coraco-arcualis* (**coa**) and joins the dorsal portion of the *genio-coracoideus* (**gco**), which is ventral to **coa**.

The *coraco-branchiales* are slips of muscles immediately ventral to the floor of the pharynx. The most anterior one is the *coraco-branchialis 1* that originates from a dorsal posterior portion of the *coraco-hyoideus* (**coh**) and inserts on the posterolateral part of the *basihyal* (**bh**) cartilage, dorsal to the *coraco-hyoideus* (**coh**) insertion on the same cartilage. These two muscle bundles surround the articulation of the *ceratohyal* (**ch**) and the *basihyal* (**bh**) ventrally and dorsally.

*Other visceral muscles* (Figs 42 and 43). The *cucullaris* (**ccl**) is a trapezoidal muscle sheet located above the branchial region. It originates on the dorsolateral fascia of the epaxial musculature immediately posterior to *constrictor hyoideus dorsalis* (**chd**), and inserts on the dorsal end of extrabranchial cartilages, anteriorly to the scapula. The *constrictor oesophagi* is a circular and thick muscle that surrounds the esophagus. At its most anterior portion it attaches to *ceratobranchial 5* (**cb5**) and both posterior *basibranchials* (**bb**); it is associated to the dorsal region of the *gill pickax* (**gp**).

**Fig 44:**
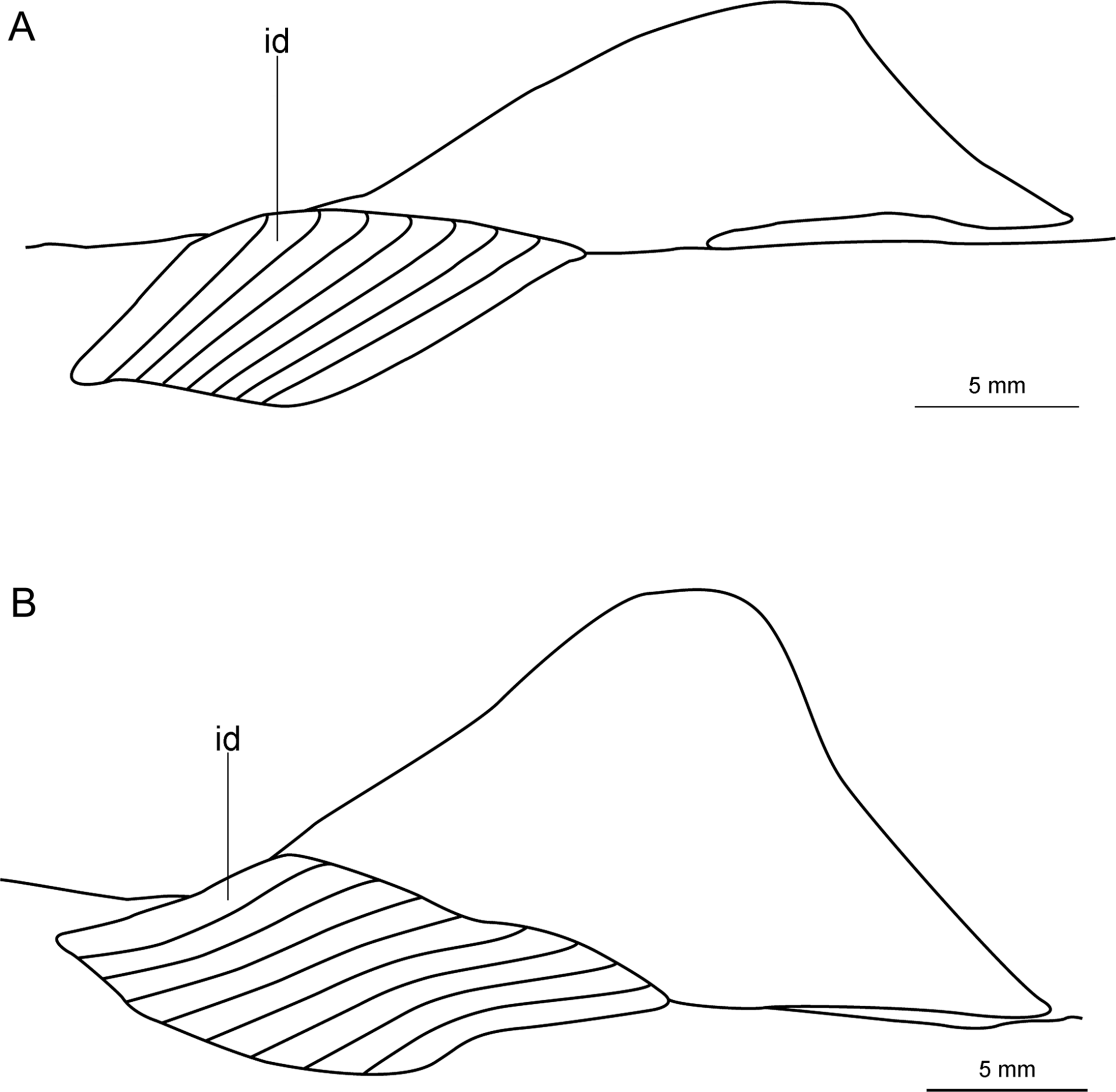
Dorsal fin musculature of *Isistius brasiliensis* (HUMZ 157844) from Northwestern Pacific Ocean. Lateral views of (A) first, and (B) second dorsal fins; anterior to left. Abbreviation: **id**, inclinator dorsalis.

*Dorsal-fin muscles* (Fig 44). Each base of the dorsal fin skeleton is covered by the *inclinator dorsalis* (**id**), which is a wide and flat muscle. It originates on the ventral margin of basal cartilaginous elements of the fin and the body musculature, and inserts on radials and ceratotrichia; it does not reach the vertebral column. This muscle is present on each side of the dorsal fin and in both dorsal fins.

**Fig 45:**
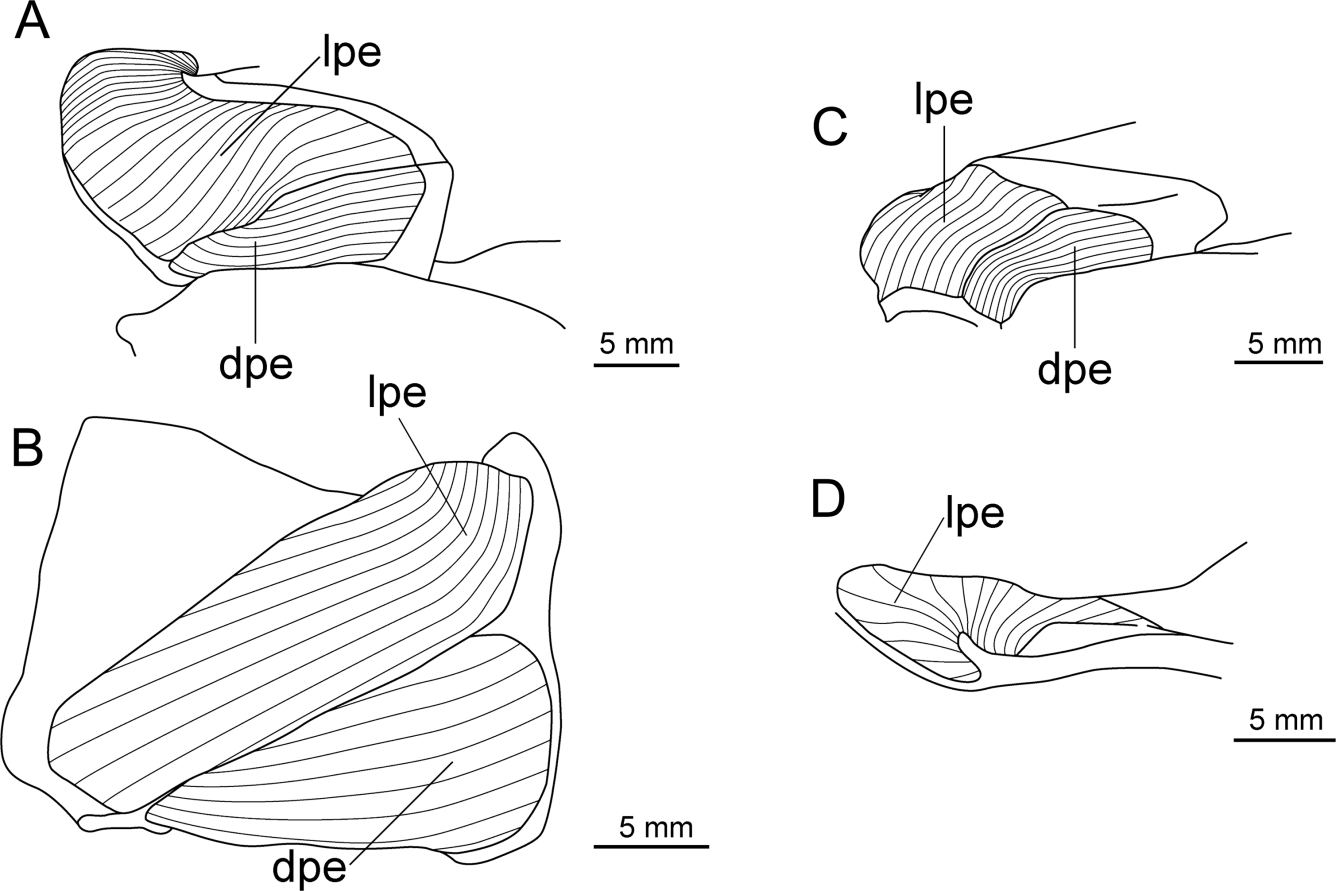
Pectoral fin musculature of a specimen of *Isistius brasiliensis* (HUMZ 211104) from Northwestern Pacific Ocean. (A) lateral view; (B) anteroposterior view; (C) ventral view; (D) dorsal view. Abbreviations: **dpe**, depressor pectoralis; **lpe**, levator pectoralis.

*Muscles of pectoral girdle and fins* (Fig 45). There are two muscles associated with the pectoral fin: the *levator pectoralis* (**lpe**) and the *depressor pectoralis* (**dpe**). The *levator pectoralis* covers half of the anterior, the dorsal, and half of the posterior sides of the pectoral fin. It is larger than the *depressor pectoralis* and originates on the posterior portion of scapula, covering the dorsal half of the anterior and posterior pectoral radials, and inserting on their tips. The *depressor pectoralis* covers the ventral portion of the anterior and posterior sides of the radials. Both muscles are connected to the *rectus cervicis*.

The hypaxial musculature completely covers the coracoid bar, and is continuous with the *rectus cervicis*, originating on the posterior portion of the girdle very close to the fin. There are muscle bundles associated with the anterior and posterior sides of the coracoid bar, as well as ventrally, and the girdle it totally nested within muscles.

**Fig 46:**
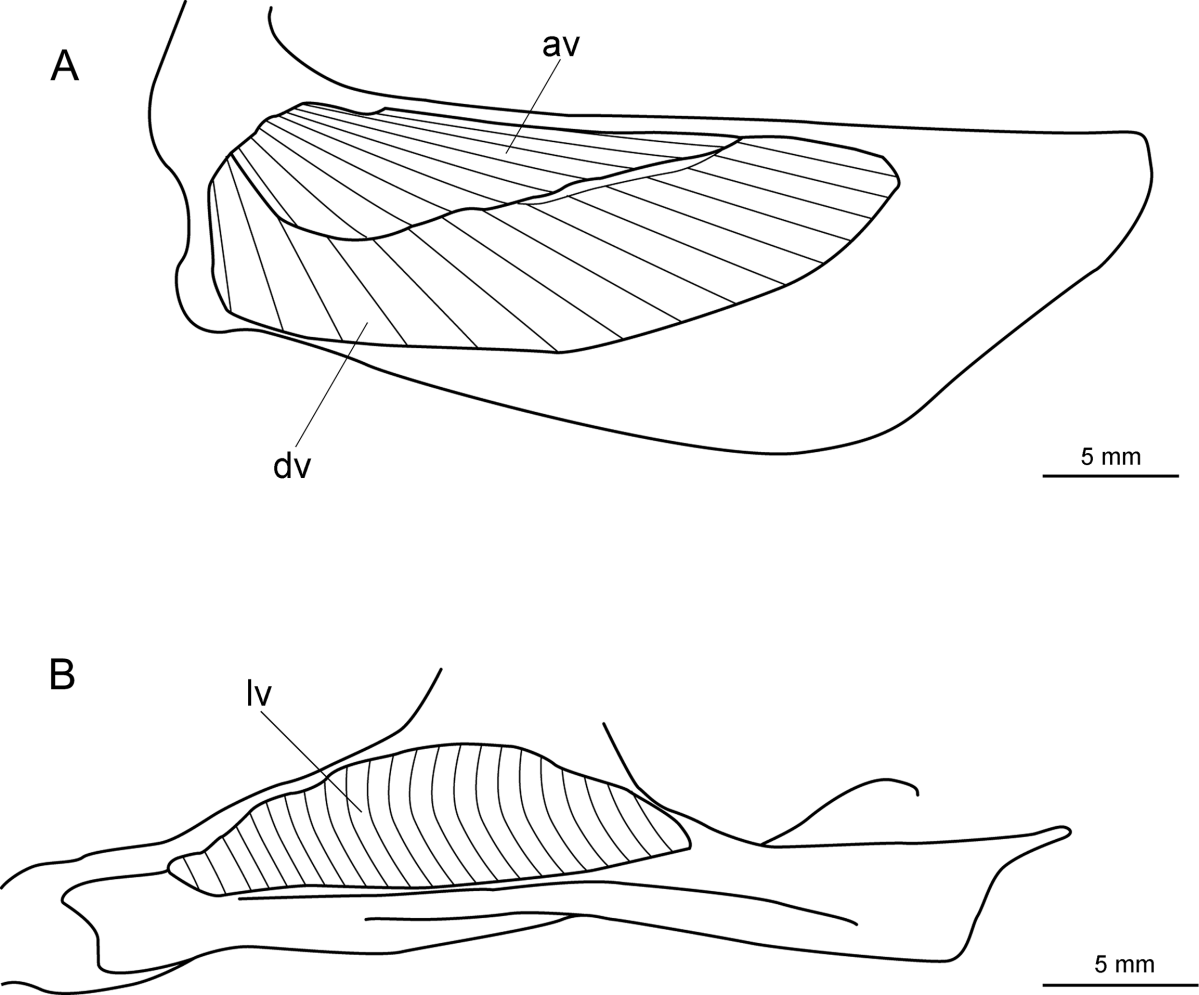
Pelvic fin musculature of *Isistius brasiliensis* (BPBM 24471). Anterior to left: (A) ventral; (B) lateral. Abbreviation: **av**, adductor pelvicus; **dv**, depressor pelvicus; **lv**, levator pelvicus.

*Muscles of pelvic fin* (Fig 46). There are three muscles in the pelvic fins: the levator, adductor and depressor muscles. The *levator pelvicus* (**lv**) originates on the ventrolateral surface of the hypaxial body musculature and inserts on the dorsal side of the pelvic fin, reaching the radials. It starts at the beginning of the pelvic girdle, and supports the fin until the ceratotrichia. It does not cover the whole dorsal surface of the fin and ends on a diagonal line from the end of the fin base until almost its external lateral corner. The ventral muscle, the *adductor pelvicus* (**av**), supports the rest of the dorsal side of the fin. This muscle originates from the most central part of the puboischiatic bar and from some connective tissue found in between both fins; it inserts on the ventral posterior portion of the basipterygium and ends at its distal portion. As it covers the internal region of the ventral side of the pelvic fin, the *adductor pelvicus* (**av**) makes a turn, surrounding the basipterygium reaching the dorsal side of the fin at its most internal portion. The other pelvic muscle is the *depressor pelvicus* (**dv**), which is also ventral and originates from the puboischiadic bar. However, it originates from the ventral side of this cartilage and covers almost all of its length and width. The only portion of this cartilage that is left exposed is the medial portion, which is rhomboidal. The *depressor pelvicus* (**dv**) covers the part of the ventral side of the pelvic fin that is not covered by the *adductor pelvicus* (**av**). It also extends farther than the previous muscle and reaches and inserts on the ventral side of the radials. There is, approximately, one muscular bundle per radial.

In males, there is a muscular bladder, the *siphon* (**sp**) [47]. It is positioned on the ventral side of the pelvic fin, ventral to the *adductor* (**av**) and *depressor pelvicus* (**dv**) muscles. It is a sac opening in the clasper duct at the lateral portion of the *dilator* (**dl**) muscle. The anteriormost portion of the *siphon* reaches the puboischiatic bar; its lateral and posterior regions cover most of the ceratotrichia, leaving only a portion of it free of muscle. The *siphon* is strongly attached to the medialmost radial present only in male specimens.

The pelvic musculature in *Isistius plutodus* is very similar to *I*. *brasiliensis*; however, the *siphon* is shorter than in *I*. *brasiliensis*, as its anterior portion is posterior to the puboischiatic bar and does not reach it.

**Fig 47:**
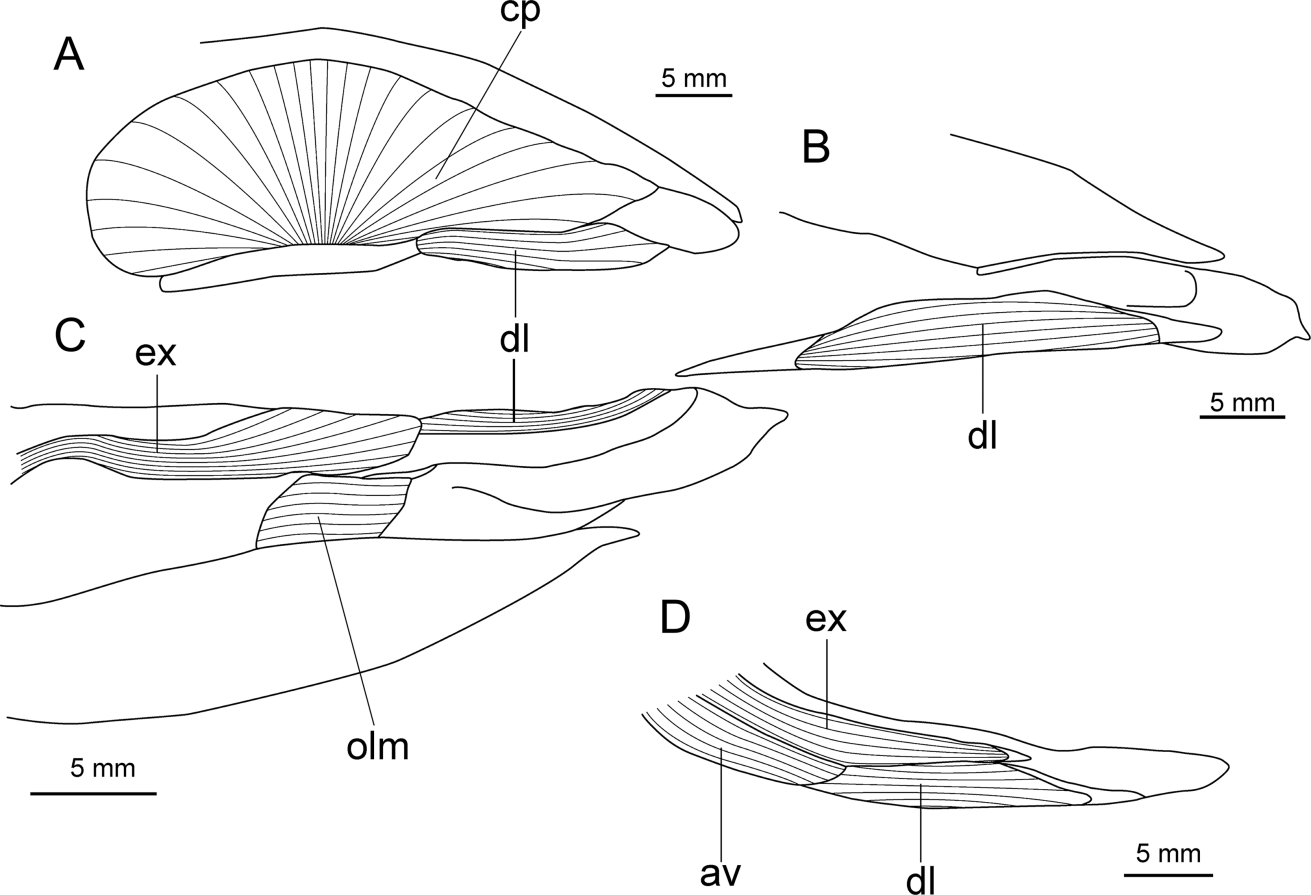
Clasper musculature (left side) of *Isistius brasiliensis* (UFPB 2669). (A) ventral view with siphon; (B) ventral view without siphon; (C) dorsal view; (D) mediolateral view. Abbreviations: **av**, adductor pelvicus; **cp**, compressor; **dl**, dilator of clasper; **ex**, extensor of clasper; **olm**, outer lip muscle.

*Clasper muscles* (Fig 47). Four muscles compose the clasper musculature. There are two bundles of the *extensor* (**ex**) muscles both running in the same direction, but one is more dorsal and internal than the other. These are dorsal muscles that originate from the posterior portion of the puboischiatic bar. They are dorsal to the *adductor pelvicus* (**ad**) and cover it almost completely. Posterior to them, at the dorsal distal part of the clasper, there is the *dilator muscle* (**dl**), which is the most internal muscle and inserts on the ventral terminal cartilage (**tv**) through a long and thick tendon. The *dilator* (**dl**) makes a turn on the inner portion of the clasper and is also found on its ventral side. Its tendon is apparent on the ventral side too, and inserts on the ventral portion of the gland. Therefore, there are no muscles directly inserting on the gland, just the tendon of the *dilator* (**dl**). The other clasper muscle is the *outer lip muscle* (**olm**), which is rather short and dorsally positioned on the clasper. It is a bundle that originates at the distal part of the *extensor*, extends on the side of the dorsal terminal cartilage lateral to the dorsal marginal, and ends right before the spur. The last clasper muscle is the *compressor* (**cp**), which is a wide and flat muscle laying ventral to the *adductor pelvicus* (**av**) and *depressor pelvicus* (**dv**).

**Fig 48:**
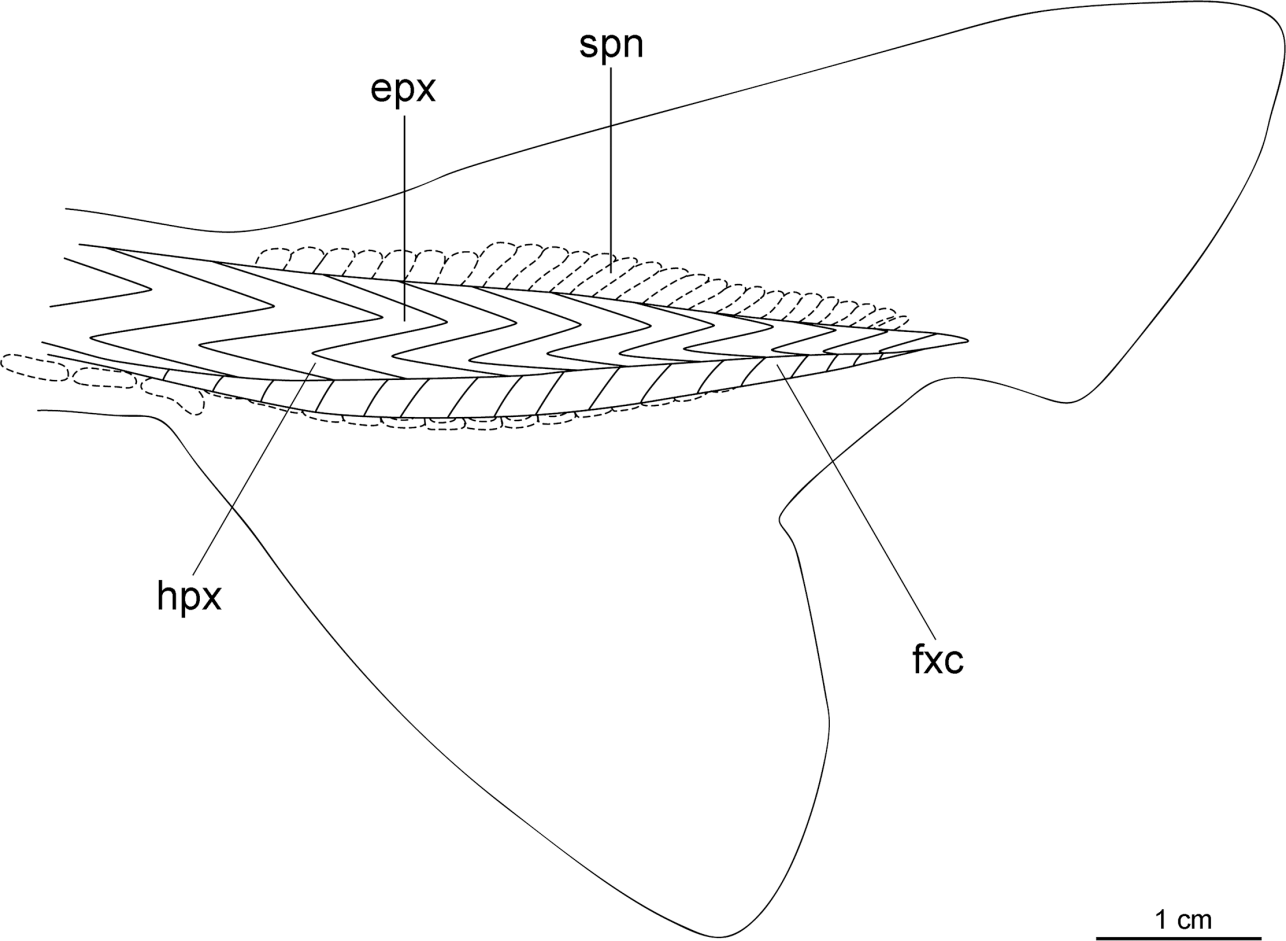
Caudal fin musculature (left side) of *Isistius brasiliensis* (MNHN 1996–0465). Abbreviations: **epx**, epaxial; **fxc**, flexor caudalis; **hpx**, hypaxial; **spn**, supraneural elements.

*Caudal fin muscles* (Fig 48). *Epaxial* (**epx**) and *hypaxial* (**hpx**) muscles cover and are attached to the caudal fin skeleton. The *flexor caudalis* (**fxc**), at the base of the lower caudal lobe, originates from the ventral surface of the hypaxial musculature and inserts on the distal ends of haemal spines and ceratotrichia of the lower caudal lobe. The supraneural spines (**spn**) are exposed and not covered by muscles.

#### Lateral-line canals

**Fig 49:**
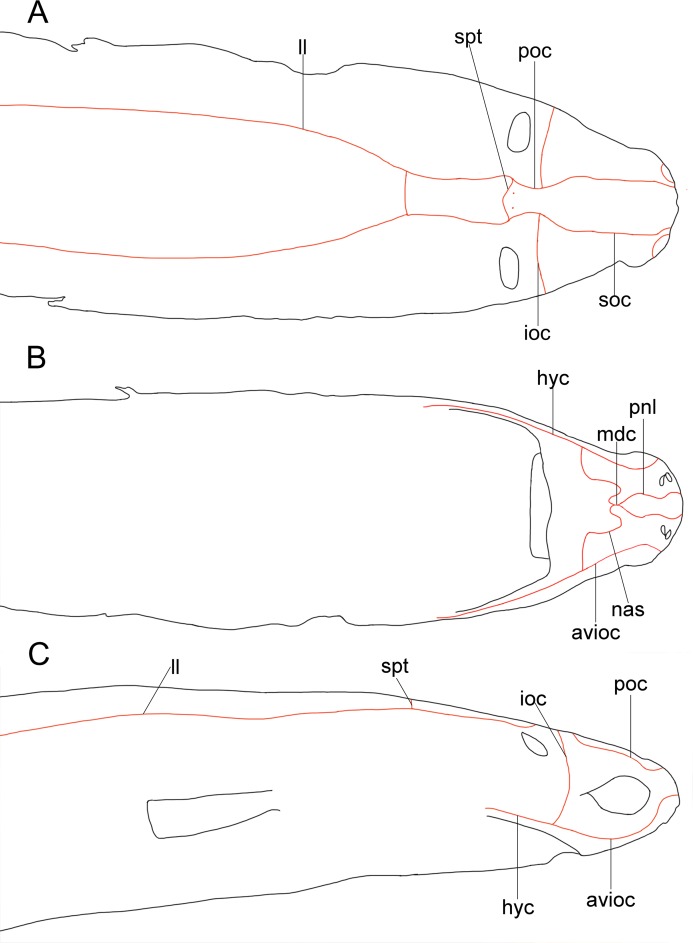
Lateral line canals of trunk and head of *Isistius brasiliensis* (MNHN 1996–0465). Dorsal (A), ventral (B) and lateral (C) views. Abbreviations: **avioc**, antero-ventral portion of the infraorbital canals; **hyc**, hyomandibular canals; **ioc**, infraorbital canals; **ll**, lateral line; **mdc**, medial canal; **nas**, nasal; **pnl**, prenasal canal; **poc**, preorbital canals; **soc**, supraorbital canals; **spt**, supratemporal canal.

**Fig 50:**
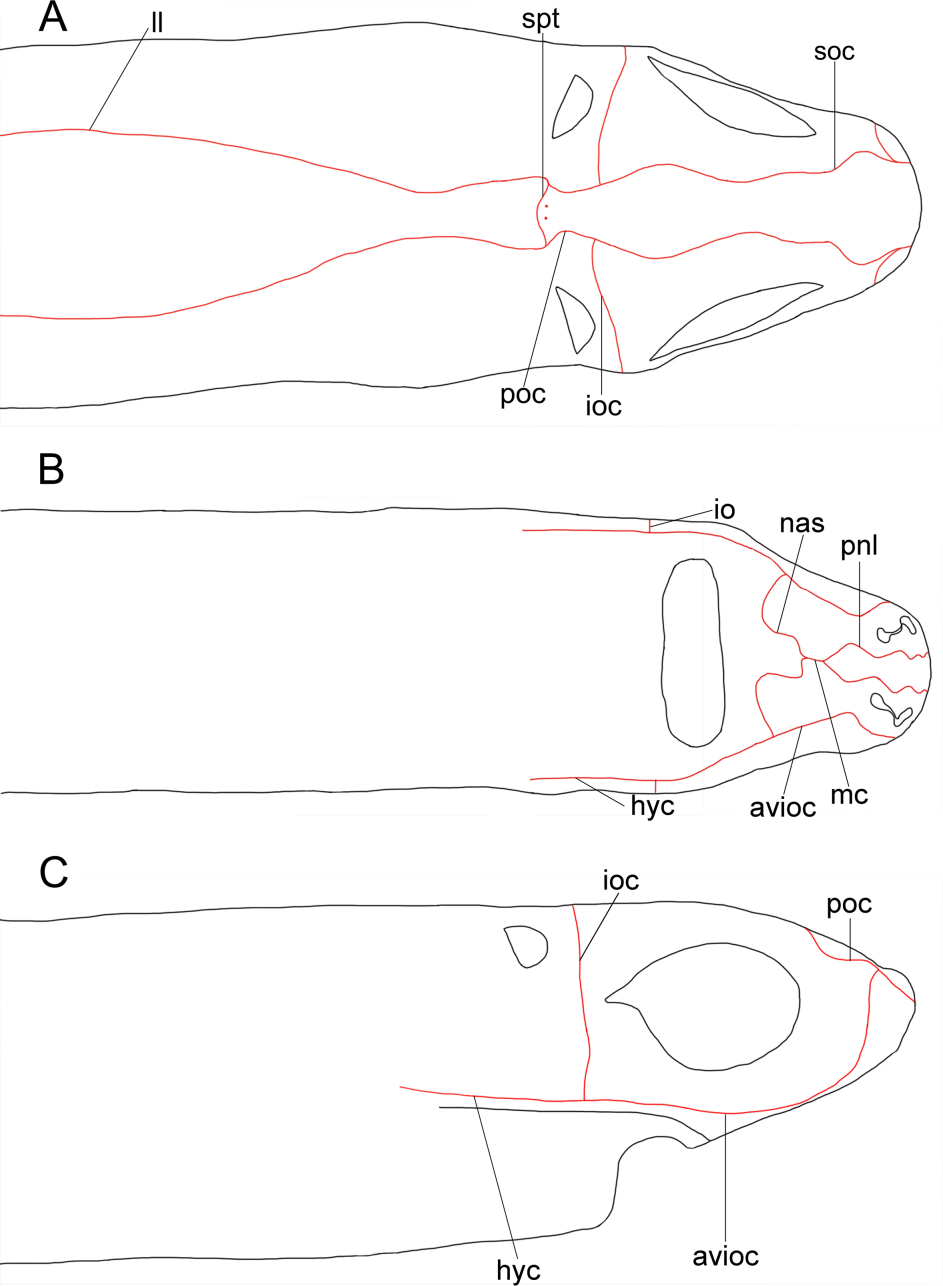
Lateral line canals of trunk and head of *Isistius plutodus* (ZUEC 8333). Dorsal (A), ventral (B), and lateral (C) views. Abbreviations: **avioc**, antero-ventral portion of the infraorbital canals; **hyc**, hyomandibular canals; **ioc**, infraorbital canals; **ll**, lateral line; **mdc**, medial canal; **nas**, nasal; **pnl**, prenasal canal; **poc**, preorbital canals; **soc**, supraorbital canals; **spt**, supratemporal canal.

The lateral-line canals are easily observed on the lateral trunk (Figs 49 and 50) and nearly reach the end of the caudal fin. In dorsal view, the lateral-line canals on either side are somewhat parallel to each other from above the gill openings to the caudal fin, but more so in *I*. *brasiliensis*. Both lateral-line (**ll**) canals progressively converge anteriorly and are very close to each other just posterior to spiracles, where they are connected to the short, perpendicular and posteriorly curved supratemporal canal (**spt**). Anterior to the supratemporal canal, the preorbital (**poc**) and supraorbital canals (**soc**) extend anteriorly toward the snout tip. In *I*. *brasiliensis* they are more or less straight but in *I*. *plutodus* they are sinuous. The infraorbital canals (**ioc**) extend laterally from the junction of the supraorbital and preorbital canals, perpendicular to the body axis, between the spiracle and eye. They extend ventrally on the lateral side of the head. The supraorbital canals continue anteriorly and pass to the ventral snout area as the prenasal canals (**pnl**). Posterior to the nostrils on the ventral snout region, the prenasal canals meet at midline to form the short medial canal (**mdc**). The nasal canals (**nas**) are sinuous, forming a small loop (more or less acute and differently shaped in both *Isistius* species), and extend on the ventral snout area between the medial canal and the lateral anteroventral portion of the infraorbital canals (**avioc**). This canal extends anteriorly ventral to the eyes from the more posterior hyomandibular canals (**hyc**), from which it is separated by the infraorbital canal. Anteriorly, the anteroventral portion of the infraorbital canals encircle the nostrils. The hyomandibular canals (**hyc**) continue along the ventrolateral portion of the head and end posterior to the mouth corner together with the end of the skin of the labial furrow. No mandibular canal was observed.

## Discussion

### Morphological variation in *Isistius*

Prior to the present revision, three species of *Isistius* were recognized as valid: *I*. *brasiliensis*, *I*. *plutodus* and *I*. *labialis*. After extensive research of the existing literature and examination of almost all specimens of *Isistius* in collections, including type specimens, we were able to unequivocally conclude that the nominal species *I*. *labialis* Meng, Zhu & Li (1985) [17] falls within the variation present among specimens of *Isistius brasiliensis*; we therefore place *I*. *labialis* in the synonymy of *I*. *brasiliensis*. *Isistius plutodus* is clearly a valid species, distinct in many characters from *I*. *brasiliensis*, including features of its skeleton and muscles, as described above.

There are differences in coloration among specimens of *I*. *brasiliensis*, such as the absence of the darker collar in some specimens, as first mentioned by Müller & Henle [30] in their account of *Scymnus brasiliensis unicolor*. Their holotype specimen came from the Western Indian Ocean, as well as another specimen deposited in the Natural History Museum of Vienna that they also examined. While the holotype of *Scymnus brasiliensis unicolor* does not present the darker collar, the other specimen does. A recently caught specimen from the Eastern Indian Ocean, which was examined by us only through photographs of the fresh specimen (made available by W. White, CSIRO), has the typical coloration of *I*. *brasiliensis*, with a dark brown color on the dorsal side and on the ventral collar, while the ventral side of the body is lighter brown. These are the only known specimens from the Indian Ocean, and although the type by Müller & Henle [30] lacks the darker collar, this is probably due its very dark dorsal and ventral color and its poor preservation.

There is also some variation in coloration in *I*. *plutodus* among preserved specimens. Garrick & Springer [15] affirmed there was no ventral dark collar in the holotype, and described the specimen as dark brown on dorsal and ventral sides with the exception of a paler ventral region between the mouth and gill openings. However, G. Burgess (FLMNH) has made available to us the original photographs used by Stewart Springer to describe the new species and a distinct darker ventral collar was indeed originally present (Fig 24) This difference is observed in other specimens of *I*. *plutodus*. The ventral light brown color posterior to the mouth mentioned by Garrick & Springer [15] usually precedes the darker collar in many analyzed specimens. Furthermore, the holotype, which is currently not dark brown as they mentioned but a light caramel color, has a subtle difference in color at the posterior end of the collar. A specimen of *I*. *plutodus* recently collected in the Gulf of Mexico (made available by M. Grace of NOAA) is dark brown on its dorsal side and light brown ventrally but with a distinct ventral collar, resembling other analyzed specimens of *I*. *plutodus*.

Variation in coloration other than in the ventral darker collar is present in both species, such as in the white tips of all fins, the darker region in the distal portion of the caudal fin (with the exception of the tips, which are usually white), and in the center of all fins. Furthermore, while some recently collected and better preserved specimens show a vivid brown color, older and poorly preserved specimens may vary from dark brown to a very pale beige. However, in specimens in which it is possible to observe the ventral darker collar, if the specimen is lighter than expected the collar is usually also lighter, even though it is darker then the rest of the ventral side.

Another feature that is variable among *Isistius* specimens is the presence of photophores. Generally, it is possible to observe their existence without a stereomicroscope as small black spots mainly on the ventral surface. The photophores also contribute to giving a darker coloration to specimens depending on their frequency and spacing between each other. As there are no photophores in the ventral collar, it may be harder to differentiate if the specimen has many photophores, as the individual will have a darker ventral side. There might also be some photophores on the dorsal side of the head, lateral region of pectoral fins, dorsal, caudal and pelvic fins, and claspers, with the exception of their white tip. However, many specimens entirely lack photophores, or may have some only on the ventral side and none on dorsal side, or lack photophores on fins. The shared feature among all specimens is the absence of photophores on the ventral collar region. Even though a specimen may not have a distinguishable collar, there is no photophore in this region. As a result, both *Isistius brasiliensis* and *I*. *plutodus* show intraspecific variability in terms of coloration, ventral collar intensity and photophore distribution, all of which may be compounded by poor preservation.

A trait previously not reported that can facilitate the identification of *Isistius* species is the gum, which is wavy and short in *I*. *brasiliensis* and straight and longer in *I*. *plutodus*. Likewise, the differences in the robustness of the skeletal mandibular and hyoid arches, as well as the absence of the upper posterior labial cartilage and the *levator labialis* muscle in *I*. *plutodus*, may together have consequences for the feeding mechanism of this species. These features are to some degree responsible for the peculiar feeding mechanism of *I*. *brasiliensis*, and are absent or more subtle in *I*. *plutodus*. In addition to these features, the relative size of the lower symphyseal tooth in relation to the parasymphyseal tooth is also a character that differentiates both species, as in *I*. *brasiliensis* the lower symphyseal tooth is approximately 5% shorter than those adjacent to it, while in *I*. *plutodus* they are the same height.

### Comparative morphology of *Isistius* and other squaliform sharks

The skeletal, muscular, and lateral line features observed in *Isistius* were compared to specimens of the families Dalatiidae, Etmopteridae, and Oxynotidae, data of which were collected from analyzed specimens and/or pertinent literature. Some structures of systematic relevance are commented on below.

#### Branchial arches

The branchial arches of *Isistius* are highly modified even in relation to other dalatiids, raising doubts about the correct identity of these cartilaginous elements. In *Isistius*, there is only one visible pair of hypobranchials and, as they are connected to the fifth ceratobranchials, are here considered to be the fifth hypobranchials. As no other paired element was observed in *Isistius*, it could be hypothesized that this species lost the four anterior hypobranchials or that they are fused to the remaining fifth hypobranchial element. Only an ontogenetic study will resolve this conflict.

A similar condition is found in *Trigonognathus*, as described by Shirai & Okamura [354], even though there are two pairs of cartilages anterior to what is considered the fifth hypobranchials. Another shared gill arch similarity between these two genera is the presence of only one basibranchial element. As this cartilaginous piece is directly connected to the hypothesized hypobranchials 5, it could be the basibranchial 5. Posterior to this cartilage, there is another one with a similar shape but tapering posteriorly. As it lies dorsal to the heart, it may be considered the basibranchial copula [355]. Shirai [3] defines a basibranchial copula as comprising basibranchials 4 and 5 and hypobranchials 5, which is not the case in *Isistius* as these cartilages are distinct from each other in *I*. *brasiliensis*. Therefore, the posteriormost basibranquial cartilage in *Isistius* could be the elongate accessory cartilage of Gegenbaur (*apud* [3]).

The unique arrangement of the hypo- and basibranchials observed in *Isistius*, with only one pair of hypobranchials and a single (in *I*. *plutodus*) or divided (*I*. *brasiliensis*) slender basibranchial, is not observed in other dalatiid genera. The analyzed specimen of *Dalatias licha* (HUMZ 74585), which was also studied by Shirai [3], has a pair of basibranchials connected anterolaterally to ceratohyal 3 and posteriorly to a wide and single cartilage which is anterior to the basibranchial copula. Anterior to the basibranchials 3 there are two single cartilages positioned in the midventral region of the branchial basket. The posterior element has a posterior extension that does not connect to any other cartilage, and lateral extensions that connect to ceratobranchials 2. Therefore, this cartilage is probably the hypobranchial 2, as suggested by Shirai [3], since it can possibly be a fusion of the hypobranchials and basibranchial 2. Anterior to it in *Dalatias* there is the other single and similar cartilage that Shirai [3] identified as the hypobranchial 1 as it connects laterally to ceratobranchials 1. These two single cartilages may be considered a fusion of a basibranchial to two hypobranchials as they are single cartilages positioned in the midventral line of the basibranchial region that connect laterally to ceratobranchials.

Shirai [3] also reported on a cleared and stained specimen of *Squaliolus laticaudus* (HUMZ 74974). Similar to what was observed in *Dalatias*, in *S*. *laticaudus* the anteriormost cartilage of the basibranchial region is a single cartilaginous piece that is connected to ceratobranchial 2. However, there is a single minute cartilage connected to it posteriorly. The anteriormost element may be the fused hypobranchials 2, while the posterior one could be the basibranchial 2. The hypobranchials 3 are connected to the ceratobranchials 3, which in turn are posteriorly connected to a single cartilage that is presumably the basibranchial 3. This basibranchial lies between two paired cartilages, which are connected to ceratobranchials 4; this pair may represent the hypobranchials 4. These three cartilages, hypobranchials 4 and basibranchial 3 are posteriorly connected to a wide cartilage, which is anterolaterally connected to ceratobranchials 5 and posteriorly to the basibranchial copula.

In *Isistius*, ceratobranchial 1 is far from the basihyal, which is a wide cartilage positioned dorsal to mandibular cartilage and somewhat inside its concavity. This condition is also observed in *Dalatias*, in which ceratobranchials 1 are connected to hypobranchials 1. However, in *Isistius*, ceratobranchials 1–4 do not connect to any other cartilages, while in *Dalatias* they connect to hypobranchials and basibranchials. This lack of connection between ceratobranchials 1 and the basihyal is also not observed in *Squaliolus laticaudus*. Other dalatiid genera should be studied in order to better understand this arrangement.

The absence of a connection between ceratobranchials 1 and the basihyal, as well as the possible absence (or fusion) of hypo- and basibranchials 1–4 leaves a wide space in the ventral region of the pharynx. Also, as the basihyal is not connected to ceratobranchials 1 its movements can be broad. Therefore, as suggested by Shirai & Nakaya [14], this particular arrangement in *Isistius* may be related to the retraction of the basihyal due to its unusual feeding behavior.

Ventral to the branchial basket, the poorly calcified ventral extrabranchial cartilages cover the ventromedial portion of the arches. These are dorsally connected to ceratobranchials (almost at their articulation with the epibranchials) and extend to the ventral end of the *constrictor branchialis superficialis* muscle, laterally surrounding the *coraco-arcualis* muscle. Although only specimens of *Isistius* were dissected in order to understand these structures, they were also observed in a specimen of *Dalatias licha* (MZUSP 123085). However, radiographs of other dalatiids show four darker slits in the branchial region that could be the ventral extrabranchial cartilages. These cartilages provide a better lateral support for the thick hypobranchial musculature present in dalatiid sharks, whose feeding habits rely on these muscles to pull the basihyal cartilage posteriorly.

#### Dorsal fins

No fin spine is present in any dalatiid species, except for the first dorsal fin of *Squaliolus*, whose spine might be hidden underneath the skin. The first dorsal fin has small, medium, and large radials posterodorsal to the basal cartilage. In *I*. *brasiliensis*, it is common to observe a separate posteroventral element, quite often perpendicular to body axis. The analyzed specimen of *I*. *plutodus* did not show this cartilage; however, it may be present as the dorsal fin skeleton may be somewhat variable.

The second dorsal fin has a posteroventral radial that is laterally curved and forms a right angle with the fin axis. This lateral radial was also observed in the first dorsal fin of a specimen of *Squaliolus laticaudus* (HUMZ 74974), and in the second dorsal fin of *Etmopterus lucifer* (HUMZ 35480), *Miroscyllium sheikoi* (HUMZ 74982), and *Oxynotus bruniensis* (HUMZ 91383). However, in both analyzed specimens of *Dalatias licha* (HUMZ 74585, HUMZ 74603 and MZUSP 123085), no lateral expansion of a radial element was observed in the dorsal fins.

Holmgren [356], in the description of the dorsal fins of *Dalatias licha*, mentioned the presence of a fin spine in the first dorsal (with dentin and enamel), but lacking in the second dorsal fin. As there are some anteroventral cartilages in both dorsal fins of *Dalatias* that are not supported by the basal cartilage, Holmgren [356] considered these to be basals as well. But Fürbringer [357] identified these cartilages as radials, associated to a single basal element. This same author previously described the dorsal fins of *Dalatias* as having fin spines: a rudimentary one in the first dorsal and a more pronounced spine in the second. However, no fin spine was observed in specimens of *Dalatias* we examined. Although there is an anterodorsal indentation on the basal cartilage ("rod" of Shirai [3]), which in other squaliform species supports fin spines, there is no evidence of a fin spine in *Dalatias*.

#### Pectoral fin

*Isistius* and all dalatiids have a single basal pectoral-fin cartilage which could be described as the fusion of the three elements: propterygium, mesopterygium, and metapterygium. This was observed not only in all analyzed specimens of the genus *Isistius*, but also in *Dalatias licha* (MZUSP 123085), *Euprotomicrus bispinatus* (BPBM 40404, LACM 55939–1), *Squaliolus aliae* (UF 159376), *Squaliolus laticaudus* (USNM 365693, LACM 36279–7) [3].

#### Clasper skeleton

The clasper skeleton is very similar between *I*. *brasiliensis* (MNHN 1996–0465 and UFPB 2669) and *I*. *plutodus* (ZUEC 8333), but differs significantly from other squalomorphs. Descriptions of dalatiid claspers are very rare; Jüngersen [46] described the claspers of *Somniosus microcephalus* and compared it to those of *Squalus acanthias*, *Etmopterus pusillus*, and *Dalatias licha*, among squalomorphs. He described the dorsal marginal cartilage for *S*. *microcephalus* as an “elevated, hard calcified ridge anteriorly beginning as quite low, posteriorly becoming higher and higher, as well as thicker, and bearing in the posterior half an edge, folded to the dorsal side, irregularly indented, and collarlike” (p. 9). Gilbert & Heath [47] indicated that in *S*. *acanthias* both ventral and dorsal marginal cartilages are fused to the stem (= axial cartilage), and four terminal cartilages are posteriorly present. Compagno [41] documented that in most sharks the clasper shaft, or sperm duct, is bordered by a pair of marginal cartilages fused to the axial cartilage; in carcharhinoids, the dorsal marginal forms a curved dorsomedial wall, and the ventral marginal a comparable dorsolateral wall.

However, in *I*. *brasiliensis*, the ventral marginal cartilage is completely ventral and does not form any part of the sperm duct. On the other side of the sperm duct is the flat, poorly calcified, and long cartilage that extends until the distalmost part of the clasper, tentatively identified as the dorsal terminal cartilage. This cartilage is also seen ventrally, posterior to the ventral terminal cartilage. But as it is a poorly calcified distal part of a dorsal cartilage, it could be the dorsal terminal that has fused to another terminal cartilage. However, by the definition of Jüngersen [46], a dorsal terminal cartilage is connected proximally to the dorsal marginal, which is not the case in *I*. *brasiliensis*. The proximal portion of this cartilage resembles the description of the anterior and lateral part of the dorsal marginal of *Somniosus* by Jüngersen [46]; however, the sperm duct in *I*. *brasiliensis* runs between the actual dorsal marginal cartilage and this tentatively identified piece. The identification of this cartilage will remain unnamed until the claspers of more genera and species of dalatiids are studied, especially *Dalatias*, *Euprotomicrus*, and *Squaliolus*.

#### Mandibular arch musculature

The overall structure of the mandibular muscles is very similar in *I*. *brasiliensis* and *I*. *plutodus*. Not only the muscles have approximately the same proportions in relation to head size, but they also occupy the same relative positions. However, the *levator labialis* (**llb**), which occurs in a layer below the *adductor mandibulae superficialis* (**ams**) and above the *adductor mandibulae* (**am**), and connects the dorsolateral portion of the quadrate plate with the lower labial cartilage, is not present in *I*. *plutodus*. Two specimens of *I*. *plutodus* were dissected (ZUEC 8332, 8333), confirming the absence of the *levator labialis* in this species. However, as both specimens are from the coast of Brazil, specimens from other regions should be studied in order to corroborate the absence of this muscle. Therefore, the *levator labialis* cannot be considered derived for the genus *Isistius* [3], being exclusive to *I*. *brasiliensis*. It is intriguing that *I*. *plutodus* lacks this muscle, as it plays an important role in the particular feeding mechanism of cookiecutter sharks [14].

#### Lateral-line canals

The lateral-line canal pattern in both species of *Isistius* are very similar, apart from slight differences in the *supraorbital canal* (**soc**), which is more sinuous in *I*. *plutodus*, and the position of the *supratemporal canal* (**spt**) that is exactly in between the spiracles in *I*. *brasiliensis* but just posterior to them in *I*. *plutodus*. Ventrally, the major differences are in the *nasal canal* (**nas**), as its curvature immediately posterior to the *medial canal* (**mdc**) is steeper in *I*. *brasiliensis* than in *I*. *plutodus*; the nasal canal meets the anteroventral *infraorbital canal* (**avioc**) at different angles in both species. Also, the prenasal canal (**pnl**) is more sinuous in *I*. *plutodus*.

The *anteroventral infraorbital* (**avioc**) canal was herein so identified as there is no anterior connection to the *supraorbital* canal (**soc**). It is an anterior and somewhat ventral portion of the *infraorbital* that extends anteriorly ventral to the eyes and makes a lateral curve around the nostrils, but does not connect to any other canal. As nomenclature of lateral-line canals followed Chu & Wen [44], and the only squaliform described by them was *Squalus*, this canal was not mentioned as there is no similar canal in this genus.

The *mandibular canal* (**md**) was not observed in any specimen of *Isistius*; however, this observation is not conclusive because the skin posterior to the mouth is very thin and has much connective tissue, which may be easily damaged during dissection.

**Fig 51:**
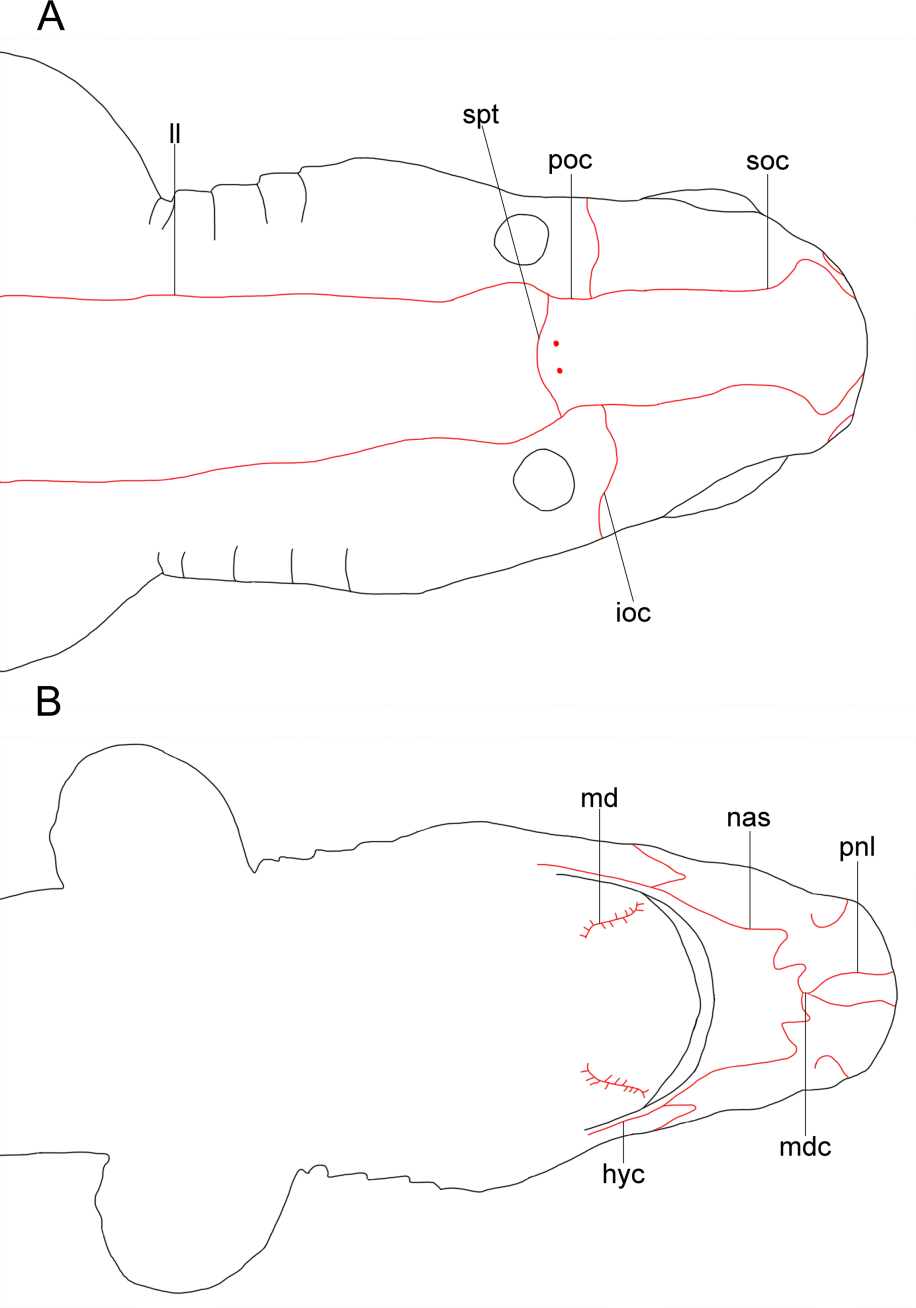
Lateral line canals of trunk and head of *Dalatias licha* (MZUSP 123085). Dorsal (A) and ventral (B) views. Abbreviations: **avioc**, anteroventral portion of the infraorbital canals; **hyc**, hyomandibular canals; **ioc**, infraorbital canals; **ll**, lateral line; **md**, mandibular canal; **mdc**, medial canal; **nas**, nasal; **pnl**, prenasal canal; **poc**, preorbital canals; **soc**, supraorbital canals; **spt**, supratemporal canal.

Dorsally, the lateral-line canals of *Dalatias* resemble those of *Isistius*, except for the position of the *supratemporal canal* (**spt**) that is slightly anterior to the spiracles (Fig 51). Ventrally, *Dalatias* has mandibular canals at the posterior corner of the mouth, one at each side, with many ramifications. Also, in *Dalatias* there is a peculiar insertion of the *infraorbital canal* (**ioc**) on the *nasal canal* (**nas**) to form the *hyomandibular canal* (**hyc**) posteriorly. Ventral to the eye and lateral to the mouth, the *nasal* canal has a small curvature directed dorsally before changing to a more ventral position; the *infraorbital* canal inserts on the anterior portion of this elevation. However, differently from *Isistius*, the *infraorbital* in *Dalatias* does not insert perpendicularly, as it extends ventrally and anteriorly, reaching the midlength of the eye. At this point, this canal abruptly curves posteriorly towards the *nasal canal*. This canal distribution in *Dalatias* is not frequently observed in other sharks [44].

### Distributional patterns

Both species of *Isistius* have a worldwide distribution. The majority of specimens are known from sites closer to the coast than in the open sea. This may be due to the fact that they might breed close to shallow waters [26]. However, as it is concluded here that *Isistius brasiliensis* and *I*. *plutodus* are unique species with worldwide distributions, and no clear-cut morphologically distinct populations exist within each species, it is questionable how the same species may occur in the Atlantic and Pacific oceans even if there is no known intermediate specimen, for example, in the south of South America. There are two possible explanations for this wide distribution, considering that they are good swimmers and able to travel long distances. The first is that its lack in between both oceans is a sampling artifact as it is not common to capture specimens that are mostly caught as bycatch.

Another possible explanation is that specimens of *Isistius* do not occur in the extreme south, as specimens would need to go below 55°S to pass between the Atlantic and Pacific oceans. Since no specimen of *Isistius* has ever been caught in latitudes higher than 44°N and 41°S, it is not probable that they could go from one ocean to another at the southern tip of South America. However, specimens occur between southern Africa and the Indian Ocean as the south of Africa is only at about 34°S. Between the Indian and Pacific oceans, specimens can occur below (38°S) or above (10°S) Australia. Hence, specimens of *I*. *brasiliensis* are found all over the world in between temperate zones. Even though no specimen of *I*. *plutodus* is known from the Indian Ocean, its occurrence there should not be disregarded as this is probably a misrepresentation due to poor sampling.

There are known teeth of the fossil species *Isistius trituratus* (Winkler, 1876) [358] from late Paleocene (Russia), early Eocene (France), and middle Eocene (Belgium), besides the species *I*. *triangulus* (Probst, 1879) [359] from eary Miocene (France), middle Miocene (Panama), late Miocene (Portugal), and early Pliocene (Belgium) [360]. These species based on fossil teeth corroborate that the genus established a worldwide distribution early in its evolution and used to be present in the region of the present day Mediterranean Sea. However, currently, there is no recorded specimen of extant cookiecutter sharks from this sea. The presence of many teeth of *Isistius* sp. From Late Miocene (Panama) [361] suggests that until recently the Atlantic and Pacific populations were still connected.

### Ecological characteristics

**Fig 52:**
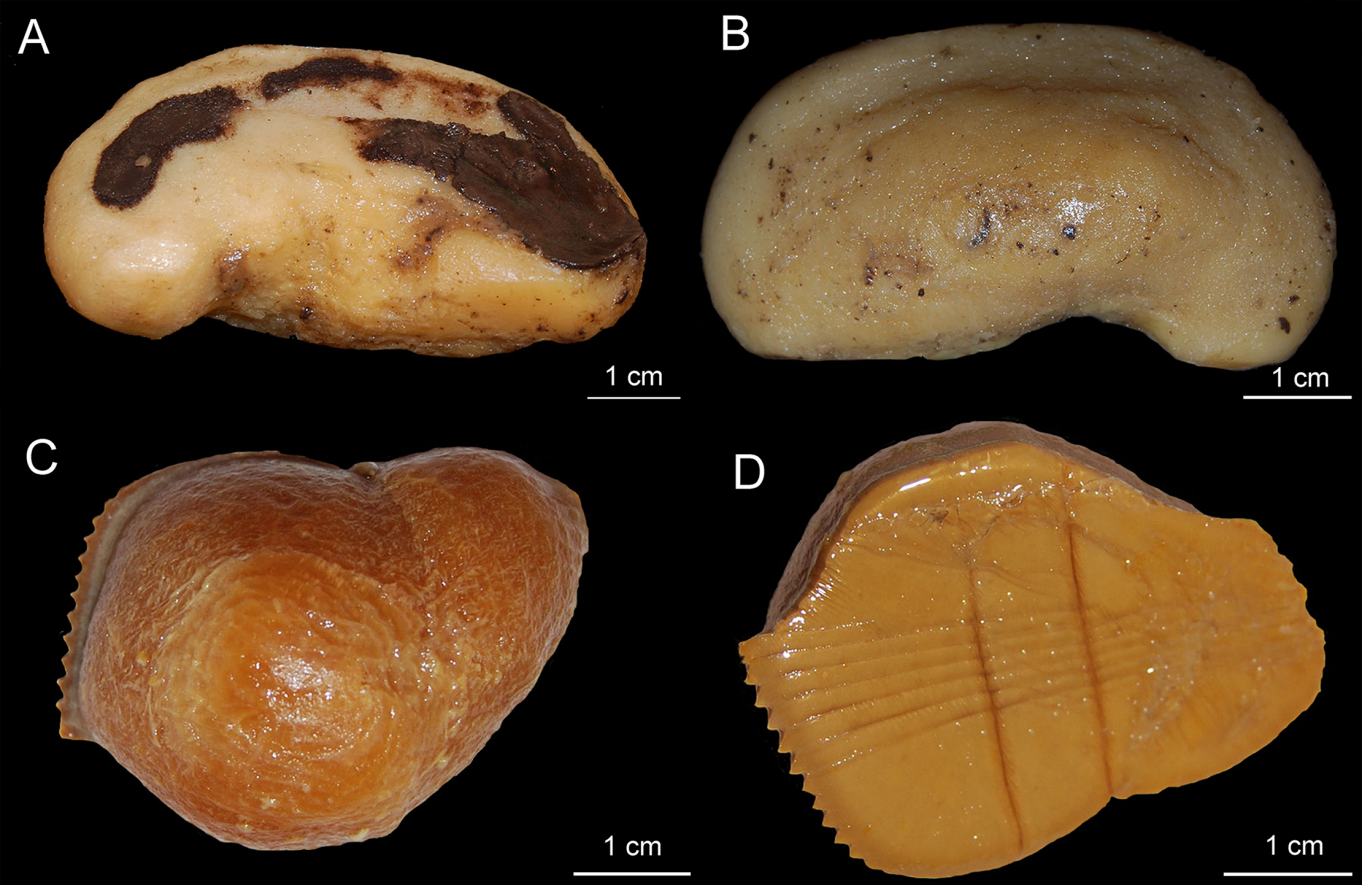
Stomach contents of two specimens of *Isistius brasiliensis*. (A) and (B) LACM 46046; (C) and (D) UW 21895. A and B are probably from a marine mammal as based on the remaining skin.

Individuals of *Isistius* may not be good swimmers because they have proportionately small pectoral and dorsal fins relative to TL. The oily liver in *Isistius* and their small dorsal and pectoral fins suggest neutral buoyancy. Besides, they are known to be ectoparasites of large fishes and cetaceans [14,110], which may be attracted by its bioluminescence (based on information on *I*. *brasiliensis* as there is little known about *I*. *plutodus*). Thick lips, strong labial cartilages, and a modified pharynx are used to attach to the prey, and the sharp lower teeth are employed to bite the skin and make a circular turn around the cookiecutter longitudinal axis, removing a piece of flesh (known as a “cookie”) and leaving the prey with a very characteristic rounded wound [14,110]. Many authors have reported wounds made by cookiecutter sharks on marine animals (S2 File), which include big fishes, such as marlins, mackerels, tunas, sharks and rays, as well as marine mammals such as seals, whales, and dolphins. Some specimens of *I*. *brasiliensis*, when cut open, have circular pieces of flesh inside their stomachs (Fig 52).

**Fig 53:**
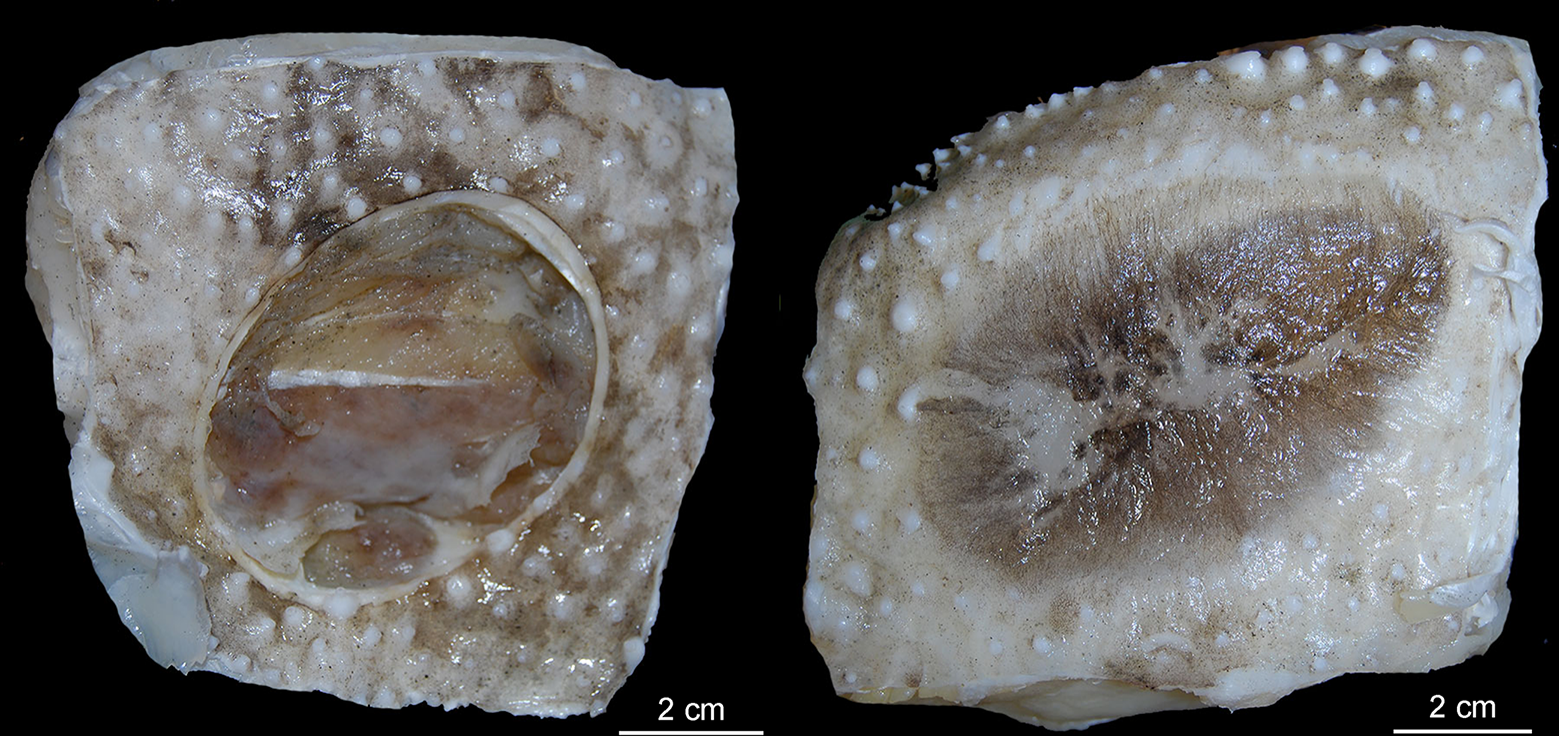
Bite wounds by *Isistius* on a specimen of oarfish. *Regalecus russelii* (SIO 13–259) that stranded on a beach in California, Northeastern Pacific Ocean.

There has not been any published report of wounds of cookiecutter sharks on oarfishes (genus *Regalecus*). However, the specimen of *Regalecus russelii* found dead in Oceanside, California, on October 21, 2013, and deposited in the collection at SCRIPPS, has circular wounds on its body. This is the first report of wounds inflicted by a cookiecutter shark on a giant oarfish (Fig 53).

As mentioned by Strasburg [26], many pieces of squids have been found inside cookiecutter shark stomachs, such as beaks and tentacles. This author also compared the volume of ingested squids with shark size: a shark of 480 ml and a squid of 500 ml, and states that such predation is possible, as *Isistius* usually prey on bigger specimens due to its large and unusual jaw structure.

**Fig 54:**
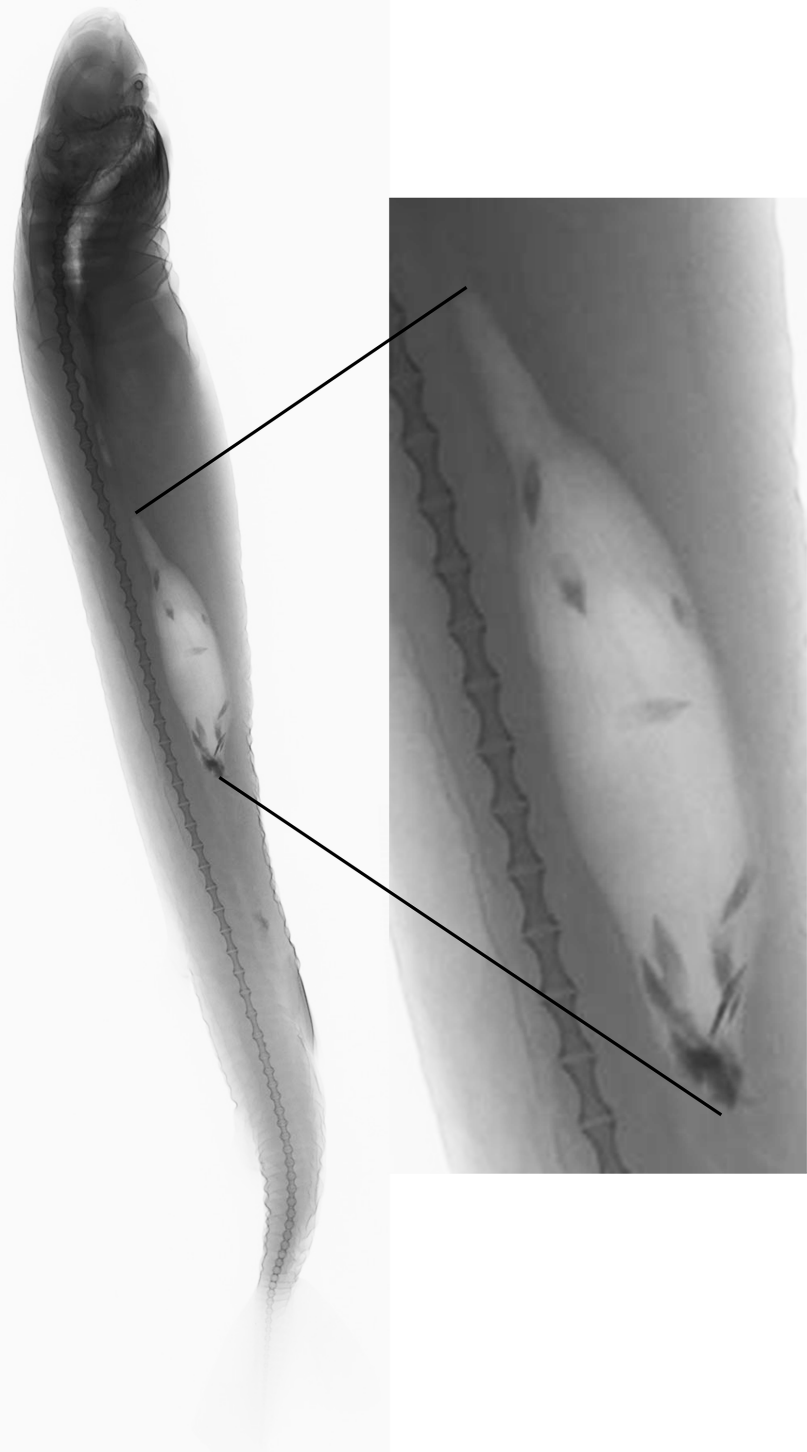
Radiograph of *Isistius brasiliensis* (MCZ 58096). Specimen from the Western Atlantic Ocean, showing its own lower teeth in stomach.

A common scenario when examining the stomach contents of *Isistius* specimens is to find their lower teeth (Fig 54). Strasburg [26] suggests these are replacement teeth and states that a specimen between 140 mm and 501 mm may have 15 tooth row replacements, which represents a loss of 435 to 465 teeth. These teeth might also be from cannibalism of other *Isistius* specimens, or may be teeth that are swallowed when they are dislocated during predation. However, Strasburg found a whole row in the stomach of a specimen, suggesting that the teeth were ingested while still articulated in their row as a mechanism to recycle calcium [33]. These teeth are usually found in stomachs before they undergo further digestion

Gadig & Gomes [22] analyzed some specimens of *I*. *brasiliensis* and inferred that the genus is lecithotrophic viviparous and that the number of neonates varies from six to 12 individuals. Although little is known regarding reproduction in cookiecutter sharks, it is believed that oceanic islands may provide a propitious environment for the growth of juveniles [26].

## Conclusions

- The genera *Isistius* and *Euprotomicrus*, which are currently regarded as being described by Gill (1865), actually date from Gill (1864) [6]. Besides, the family Dalatiidae, which has been attributed to Gray (1851) [59], was indeed first mentioned by Gill (1893) [48].
- There are two valid species in the genus *Isistius*: *I*. *brasiliensis* (Quoy & Gaimard, 1824) [16] and *I*. *plutodus* Garrick & Springer (1964) [15], both with a worldwide distribution. Nominal species for which type specimens fall within the observed variation encountered in *I*. *brasiliensis* are: *Scymnus brasiliensis* Quoy & Gaimard (1824) [16], *Scymnus brasiliensis torquatus* Valenciennes [A.] in Müller & Henle (1839) [30], *Scymnus brasiliensis unicolor* Valenciennes [A.] in Müller & Henle (1839) [30], *Leius ferox* Kner (1864) [31], *Isistius marmoratus* Rochebrune (1885) [32], *Squalus fulgens* Bennett (1840) [18], and the newest synonym *Isistius labialis* Meng, Zhu & Li (1985) [17].
- Both valid species are highly similar, but morphometrics differetiate them, as well as tooth count and size, pigmentation (proportions of the darker collar), caudal fin morphology, and morphology and proportions of the neurocranium, among other anatomical features.
- The muscle levator labialis, which plays an important role in the feeding mechanism of *Isistius brasiliensis*, was not observed in *I*. *plutodus*, as well as the upper posterior labial cartilage. These absences, in addition to less robust mandibular and hyoid arches in *I*. *plutodus*, may have some implications concerning its feeding mechanism.
- Ventral extrabranchial cartilages, which are flattened, poorly calcified, and occur ventromedially along the inner portion of the branchial arches, are observed in the branchial basket in both *Isistius* species, as well as in radiographs of other dalatiid genera, possibly enhancing their feeding strategy by supporting the thick hypobranchial musculature.
- A distinct, very large cartilage was observed in the dorsolateral portion of the *Isistius* clasper skeleton. It is here identified as the dorsal terminal cartilage but may be the dorsal marginal cartilage that separated into two pieces due to the change in path of the sperm duct. However, these interpretations are not conclusive since claspers of related species need to be studied in order to understand the morphology of this particular element and any purported distinction in the sperm duct.

## Supporting information

S1 File Material examined of family dalatiidae. (DOCX)

S2 File List of reported wounds of cookiecutter shark bites on cetaceans, fishes, a turtle, and a human corpse. (DOCX)

S3 File Permission by the American Society of Ichthyologists and Herpetologists to republish an illustration. (PDF)

S1 Table Morphometric measurements of *Isistius brasiliensis*. (XLSX)

S2 Table Morphometric measurements of *Isistius plutodus*. (XLSX)
