# Supplementary material for: Comparative morphology and systematics of the cookiecutter sharks, genus Isistius Gill (1864) (Chondrichthyes: Squaliformes: Dalatiidae)
Source: PLoS One. 2018 Aug 20;13(8):e0201913. doi: 10.1371/journal.pone.0201913 (PMC6101376; doi:10.1371/journal.pone.0201913)
Supplement: S1 File — (DOCX) [file pone.0201913.s001.docx]

**S1 FILE**

**Material examined of Family Dalatiidae**

***Isistius brasiliensis*** (243 specimens)

Southwestern Atlantic Ocean (15). C.DBAV.UERJ 1635, female 431 mm TL, Southwestern Atlantic, 23-24° S, 130 nautical miles off São Paulo, Brazil, 20 Mar 1995; C.DBAV.UERJ 1636, 8 specs., embryos 112-120 mm TL, Southwestern Atlantic, 23-24° S, 130 nautical miles off São Paulo, Brazil, 20 Mar 1995; C.DBAV.UERJ 1660.1, female 420 mm TL, Southwestern Atlantic, Brazil; C.DBAV.UERJ 1671, male, Southwestern Atlantic, Brazil; MNHN 0000-4891, female 209 mm TL, Southwestern Atlantic, South of Brazil, 23°00’00” S, 43°16’59” W, depth 250 m, 1863; MNHN A-7787 (Holotype of *Scymnus brasiliensis*, *Scymnus torquatus* and *Isistius brasiliensis*), female 172 mm TL, Southwestern Atlantic, Brazil, Expédition D. de Freycinet 1817-1820; MZUSP 16360, male 470 mm TL, Southwestern Atlantic, South of Brazil, 29°38'33,33' S, 46°20'00,01" W, 24 May 1976; ZMH 103780, male 321 mm TL, Southwestern Atlantic, 23°02'00” S, 33°19'00” W, 1966;

Southeastern Atlantic Ocean (13). CAS 222996, male 377 mm TL, Southeastern Atlantic, Angola, 7°58’60” S, 12°35’60” E, depth 630 m, 16 Apr 2005; MCZ 57370, male 282 mm TL, Southeastern Atlantic, Angola, 11°23’ S, 10°55’ E, depth 190-200 m, 4 Jun 1971; MNHN 1991-6827, male 382 mm TL, Southeastern Atlantic, Angola, 10°30’00” S, 11°55’01” E, depth 1756-1756 m, Campagne Walda 23cy15; NHM 1982.9.22.1, 2 specs., female 385 and 461 mm TL, Southeastern Atlantic, 21° 34,4' S, 8° 9,8' E and 32°108' S, 36°21,7' E, 182>? m, 1604 e 1000>0 m, 1764; SAIAB 64998, female 405 mm TL, Southeastern Atlantic, Angola, 5 Mar 2001; SAIAB 65007, female 395 mm TL, Southeastern Atlantic, Angola, 12 Mar 2001; SAIAB 65972, female 288 mm TL, Southeastern Atlantic, Angola; ZMH 108396, male 286 mm TL, Southeastern Atlantic, 30°09'00” S, 5°26'00” E, 1971; ZMH 108492, 2 specs., male 395 mm TL and female 466 mm TL, Southeastern Atlantic, 33°00'00” S, 7°50'00”E, 1971; ZMH 108493, male 371 mm TL, Southeastern Atlantic, 33°00'00” S, 7°50'00” E, 1971; ZMH 109991, male 262 mm TL, Southeastern Atlantic, 15°45'00” S, 6°06'00” W, 1971.

Northwestern Atlantic Ocean (16). MCZ 55447, male 392 mm TL, Northwestern Atlantic, Gulf of Mexico, 29°32’ N, 93°16’ W, depth 405-460 m, 20:11-23:32h, 19 Jun 1966; MCZ 55498, male 375 mm TL, Northwestern Atlantic, Caribbean Sea, 13°51’ N, 70°15’ W, 27 May 1966; MCZ 58094, female 153 mm TL, Northwestern Atlantic, Amazon, 1°41’ N, 40°37’ W, depth 0-80 m, Mar 1977; MCZ 58096, 2 specs., male 158 mm TL, female 224 mm TL, Northwestern Atlantic, Amazon, 0° 1’ N, 37°40’ W; MCZ 36039, male 382 mm TL, Northwestern Atlantic, Bahamas, 25°11’ N, 77°19’ W, depth 800-1000 m, 30 Jan 1938; NMW 78801, female 163 mm TL, Northwestern Atlantic, 25° N, 70° W; TCWC 3985.01, male 397 mm TL, Northwestern Atlantic, Gulf of Mexico, 23° 12’36” N, 90° 44’5.94” W, depth 3700 m; TCWC 5130.01, female 257 mm TL, Northwestern Atlantic, Caribbean Sea, 20°48’ N, 92°55’60” W, depth 2270 m; TCWC 5131.01, female 178 mm TL, Northwestern Atlantic, Gulf of Mexico, 19°7’48” N, 94°5’30” W, depth 935-950 m; TCWC 8770-09, female 446 mm TL, Northwestern Atlantic, Caribbean Sea, 19°46’0” N, 82°9’ W; UF 207807, female, Northwestern Atlantic, Bahamas, 23°42’ N, 76°38’ W; UF 232917, male 406 mm TL, Northwestern Atlantic, Bahamas, 23°38’ N, 77°6’ W; UF 35686, 2 specs., males 358 and 395 mm TL, Northwestern Atlantic, Gulf of Mexico, 29°4’ N, 87°37’ W; YPM ICH 3727, female 428 mm TL, Northwestern Atlantic, Bahamas, Ocean tongue, 7000 ftm SE of Booby Rocks;

Northeastern Atlantic Ocean (14). MCZ 55495, male 386 mm TL, Northeastern Atlantic, Cape Verde, 16°14’ N, 20°44’ W, 14 Nov 1970; MCZ 55496, male 375 mm TL, Northeastern Atlantic, Cape Verde, 16°28’ N, 19°45’ W, 14 Nov 1970; MCZ 55497, male ?, Northeastern Atlantic, Cape Verde, 16°32’ N, 19°35’ W, 13 Nov 1970; MNHN 0000-1179, female, 210 mm TL, Northeastern Atlantic, Cape Verde, 15°07’01” N, 23°34’59” W, Expédition Dumont d'Urville 1826-1829; NHM 1994.9.28.4, female 356 mm TL, Atlantic; NHM 1995.8.22.11, male 365 mm TL, Northeastern Atlantic, Cape Verde, 17° 43' N, 25° 23' W, depth 510-0 m; NHM 1996.7.10.29, female 427 mm TL, Northeastern Atlantic, Gambia, 10° 54,7' N, 20° 30’ W, depth 510-405 m; NHM 1996.9.11.10, male 393 mm TL, Northeastern Atlantic, Cape Verde, 17° 41' N, 25° 23' W; UF 165691, female 173 mm TL, Northeastern Atlantic, Liberia, 12° N, 28°57’ W; UF 224595, Northeastern Atlantic, Nigeria, 3°30’ N, 4°51’ E; UF 224657, female, Northeastern Atlantic, Nigeria, 5°57’ N, 2°49’ E; ZMH 103882, male 384 mm TL, Northeastern Atlantic, 19°11'00” N, 21°58'00” W, 1966; ZMH 109456, male 386 mm TL, Northeastern Atlantic, 17°24'00” N, 22°57'00” W, 1971; ZMH 109828, male 391 mm TL, Northeastern Atlantic, 17°27'00” N, 22°55'00” W, 1971;

Central Atlantic Ocean (9). MCZ 41350, male 271 mm TL, Central Atlantic, 3°02’ N, 21°19’ W, 27 Apr 1961; MCZ 41351, male 331 mm TL, Central Atlantic, 0°15’ S, 18°40’ W, 26 Apr 1961; MCZ 41352, male 274 mm TL, Central Atlantic, 11°16’60” N, 30° W, depth 0-230 m; MCZ 41353, male 349 mm TL, Central Atlantic, 02°10’ S, 17°23’ W, depth 250-350 m, 21 Apr 1961; MCZ 55499, male 274 mm TL, Central Atlantic, 11°17’ N, 30°00’ W, depth 0-230 m, May 1961; MCZ 57371, male 158 mm TL, Central Atlantic, 1°20’ S, 27°37’ W, 22h, 25 Feb 1963; MCZ 58095, female 170 mm TL, Central Atlantic, 5°32’00” N, 34°40’ W, depth 0-75 m, Mar 1977; NRM 9026, Central Atlantic, 03° 25' S, 24° 30' W, 17 Mar 1890; ZMH 106154, male 185 mm TL, Central Atlantic, 3°00'00” S, 26°16'00” W, 1968;

Pacific Ocean (3). HUMZ 126958, female 421 mm TL, Pacific, 14 Feb 1983; MNHN 1997-3575, male 385 mm TL, Pacific, Campagne Caride 6; NHM 1868.6.22.41, female 226 mm TL, Pacific;

Southwestern Pacific Ocean (55). AMS I.9280, female 424 mm TL, Southwestern Pacific, New South Wales, Lord Howe Is., Australia, 31° 31' S, 159° 05' E, 1908; AMS IA.1427, female 449 mm TL, Southwestern Pacific, New South Wales, Lord Howe Is., Australia, 31° 31' S, 159° 5' E, 1923; AMS I.5427, female 345 mm TL, Southwestern Pacific, New South Wales, Lord Howe Is., Australia, 31° 31' S, 159° 5' E, 1903; AMS IA.128, female 393 mm TL, Southwestern Pacific, New South Wales, Lord Howe Is., Australia, 31° 31' S, 159° 5' E, 1920; AMS I.14058, male 370 mm TL, Southwestern Pacific, New South Wales, Lord Howe Is., Australia, 31° 31' S, 159° 5' E, 1917; AMS I.29996-001, female 191 mm TL, Southwestern Pacific, Coral Sea, Osprey Reef, 1,5 miles west of entrance, Australia, 13° 54' S, 146° 33' 30" E, 1988; AMS I.16150-001, female 506 mm TL, Southwestern Pacific, New South Wales, Lord Howe Is., Australia, 31° 32' S, 159° 4' E, 1971; AMS I.32445-001, female 160 mm TL, Southwestern Pacific, Queensland, Coral Sea, east of Orford Ness, Australia, 11° 30' 3" S, 145° 14' 36" E, 1988; AMS I.4308, male 385 mm TL, Southwestern Pacific, New South Wales, Lord Howe Is., Australia, 31° 31' S, 159° 5' E, 1900; AMS IA.8061, ?, Southwestern Pacific, New South Wales, off Sydney, Australia, 33° 50' S, 151° 18' E, 1939; AMS I.7883, female 340 mm TL, Southwestern Pacific, New South Wales, Lord Howe Is., Australia, 1907; AMS IB.2523, female 491 mm TL, Southwestern Pacific, New South Wales, 120 miles SE Sydney, Australia, 34° 50' S, 153° E , 1950; AMS I.43092-001, male 395 mm TL, Southwestern Pacific, Coral Sea, Australia, 10° 51' 43" S, 149° 44' 6" E, 1995; AMS I.43094-001, male 362 mm TL, Southwestern Pacific, Coral Sea, Australia, 10° 51' 43" S, 149° 44' 6" E, 1995; AMS I.42095-006, male 169 mm TL, Southwestern Pacific, Coral Sea, Australia, 12° 59' 52" S, 151° 19' 37" E, 1997; AMS I.27639-002, male 393 mm TL, Southwestern Pacific, New South Wales, east of Kiama, Australia, 34° 39' S, 151° 18' E, 1988; AMS I.43073-001, male 205 mm TL, Southwestern Pacific, Coral Sea, Australia, 12° 33' 39" S, 153° 50' 38" E, 1995; AMS I.42066-002, female 244 mm TL, Southwestern Pacific, Coral Sea, Australia, 14° 36' 7" S, 151° 21' 18" E, 1997; AMS I.43100-001, 2 specs., female 330 and 364 mm TL, Southwestern Pacific, Coral Sea, Australia, 11° 1' 22" S, 149° 43' 8" E, 1995; AMS I.42172-002, female 189 mm TL, Southwestern Pacific, Coral Sea, Australia, 10° 53' 56" S, 150° 9' 21" E, 1997; AMS I.42003-004, male 238 mm TL, Pacific, Coral Sea, Australia, 13° 55' 58" S, 148° 12' 50" E, 1997; AMS I.42058-001, male 255 mm TL, Southwestern Pacific, Coral Sea, Australia, 14° 41' 20" S, 151° 19' 26" E, 1997; AMS I.42038-001, male 370 mm TL, Southwestern Pacific, Coral Sea, Australia, 15° 12' 14" S, 149° 34' 51" E, 1997; AMS I.42030-001, male 188 mm TL, Southwestern Pacific, Coral Sea, Australia, 15° 3' 57" S, 149° 20' 56" E, 1997; AMS IA.3792, female 360 mm TL, Southwestern Pacific, New South Wales, Lord Howe Is., Australia, 31° 31' S, 159° 5' E, 1929; AMS I.24161-002, female 358 mm TL, Southwestern Pacific, New South Wales, Lord Howe Is., Australia, 31° 28' S, 159° 9' E, 1983; AMS I.28069-001, Southwestern Pacific, New South Wales, off Shoalhaven Heads, Australia, 34° 53' S, 151° 14' E, 1988; AMS I.28748-004, Southwestern Pacific, New South Wales, Tasman Sea, off Sydney, Australia, 33° 49' 30" S, 152° 33' 48" E, 1989; CSIRO CA 190, male 455 mm TL, Southwestern Pacific, New South Wales, east of Bermagui, Australia, 36°27'15.75" S, 150°14'27.59" E, depth 494 m, 1978; CSIRO CA 191, male 375 mm TL, Southwestern Pacific, New South Wales, east of Bermagui, Australia, 36°30'0.56" S, 150°14'27.91" E, depth 74 m, 1978; CSIRO CA 3137, male 402 mm TL, Southwestern Pacific, Australia, New South Wales, off Macquarie harbor, 31°26'28.22" S, 153° 4'4.76" E; CSIRO CA 3302, male 342 mm TL, Southwestern Pacific, Australia, New South Wales, Bougainville Reef, 16°50' S, 147°02’ E, 1979; CSIRO H 4714, female 443 mm TL, Southwestern Pacific, Australia, Tasman Sea, Lord Howe Ridge, 28°59' S, 160°22' E, 1997; CSIRO H 5150-01, male 485 mm TL, Southwestern Pacific, Australia, New South Wales, 300 miles east of Coffs Harb, 30°17'47.61" S, 153°10'33.91" E, 1999; CSIRO H 6008-01, male 376 mm TL, Southwestern Pacific, Australia, Tasman Sea, Norfolk Ridge, 29°29' S, 167°33' E, depth 200 m, 2003; CSIRO T 544, male 404 mm TL, Southwestern Pacific, Australia, Tasmania, 33 miles east of Flinders, 40°5'59.06" S, 148°28'47.89" E, 1981; CSIRO T 718, male 438 mm TL, Southwestern Pacific, Australia, Tasmania, 33 miles east of Flinders, 40°5'59.06" S, 148°28'47.89" E, 1981; CSIRO H 3722-01, male 305 mm TL, Southwestern Pacific, Australia, Queensland, Coral Sea, 16°38' S, 152°10' E, 1994; HUMZ 141845, female 445 mm TL, Southwestern Pacific, 30° S, 165° E, Hokuyo-maru vessel, 30 Aug 1995; HUMZ 141846, male 420 mm TL, Southwestern Pacific, 12°59’60 S, 159° E, Hokuyo-maru vessel, 09 Apr 1995; MCZ 60364, female 156 mm TL, Southwestern Pacific, New Britain trench, 6°40’60” S, 150°43’ E; MCZ 60365, female 373 mm TL, Southwestern Pacific, Queensland, Coral Sea, 17°33’ S, 148°47’ E, depth 400 m, 22 Jun 1981; MCZ 60366, male 178 mm TL, Southwestern Pacific, Queensland, Coral Sea, 17°13’ S, 148°20’ E, 19:07-00:10h, 23, 24 May 1981; MCZ 60367, 2 specs., female 478 and 462 mm TL, Southwestern Pacific, Queensland, Coral Sea, 17°7’ S, 148°E, depth 500 m, 00:30-05:50h, 24 Jun 1981; MNHN 1996-0464, male 413 mm TL, Southwestern Pacific, Coral Sea, New Caledonia, 17,5° S, 167,5° E, depth 1010 m, cc327; MNHN 1996-0465, male 409 mm TL, Southwestern Pacific, Coral Sea, New Caledonia, 17,5° S, 167,5° E, depth 1010 m, p27472; MNHN 1997-3574, female 480 mm TL, Southwestern Pacific, Coral Sea, New Caledonia, 24°48’00” S, 170°07’01” E, Campagne Halipro 2, bt21, 1996; MNHN 1997-3576, male 388 mm TL, Southwestern Pacific, Coral Sea, New Caledonia, 17,5° S, 167,5° E, Campagne Mola 3; MNHN 1997-3578, male 178 mm TL, Southwestern Pacific, Coral Sea, New Caledonia, 17,5° S, 167,5° E, Campagne Mola 3; MNHN 1997-3579, male 172 mm TL, Southwestern Pacific, Coral Sea, New Caledonia, 17,5° S, 167,5° E, Campagne Mola 2; MNHN 1997-3580, female 275 mm TL, Southwestern Pacific, Coral Sea, New Caledonia, 22,16° S, 165,83° E, depth 530-540 m, Campagne Diaphus 7, TL12; MNHN 1997-3581, male 382 mm TL, Southwestern Pacific, Coral Sea, New Caledonia, 17,5° S, 167,5° E, Campagne Mola 3, fab25; NHM 1997.5.21.56, male 419 mm TL, Southwestern Pacific, 24° 21,02' S, 170° 1,02' W, depth 1140-1188 m;

Eastern Pacific Ocean (2). MCZ 1005, female 475 mm TL, Eastern Pacific, Galapagos Is., 2°34’ N, 92°6’ W, 2992 m, 05 Apr 1891; SIO 52-413, male 396 mm TL, Eastern Pacific, Galapagos Is., surface, 0° N, 100° W;

Southeastern Pacific Ocean (13). MNHN 1987-1306, male 385 mm TL, Southeastern Pacific, Polynesia, 22° S, 140° W; 1971; NMW 76230 (Holotype of *Leius ferox* Kner, 1864), female, 162 mm TL, Southeastern Pacific, Australia, 1866; SIO 02-136, male 397 mm TL, Southeastern Pacific, 15°19.2’ S, 115°47.4’ W; SIO 69-345, female 467 mm TL, Southeastern Pacific, North of Easter Is., 25°48’ S, 108°47.5’ W, depth 0-2000 m; SIO 73-162, female 332 mm TL, Southeastern Pacific, French Polynesia, 24°56.6’ S, 155°14.7’ W, depth 1000 mwo; USNM 190035, male 365 mm TL, Southeastern Pacific, 2°54’ S, 113°8’ W; USNM 190036, male 163.71 mm TL, Southeastern Pacific, Polynesia, 9°32’30” S, 139°51’ W; USNM 190037, female 190.27 mm TL, Southeastern Pacific, Polynesia, 6°26’ S, 139°52’ W; USNM 190038, female 250 mm TL, Southeastern Pacific, Polynesia, 4°32’ S, 142°49’ W; USNM 190040, female 170 mm TL, Southeastern Pacific, Polynesia, 9°33’ S, 139°49’30” W; USNM 190041, male 226 mm TL, 9°34’ S, 139°52’ W, Southeastern Pacific, Marquesas Is., 9°34’ S, 139°52’ W; USNM 190042, male 178 mm TL, 9°34’ S, 139°52’ W Southeastern Pacific, Polynesia, 11°22’ S, 139°27’ W; USNM 190043, female 179.84 mm TL, 9°34’ S, 139°52’ W, Southeastern Pacific, Polynesia, 9°17’ S, 139°16’ W;

Northwestern Pacific Ocean (16). ASIZP 0059802, male 411 mm TL, Northwestern Pacific, Taiwan, 13 Oct 1998; FRIP 00573, female 299 mm TL, Northwestern Pacific, Taiwan; FRIP00573 (F33), male 421 mm TL, Northwestern Pacific, Taiwan; HUMZ 69173, female 372 mm TL, Northwestern Pacific, Japan, Chichijima, Ogasawara, 12.5° N, 158° E, Mar 1975; HUMZ 87120, female 447 mm TL, Northwestern Pacific, 12.5° N, 158° E, Hokusei-maru vessel, 24 Jan 1980; HUMZ 89900, female 520 mm TL, Northwestern Pacific, 12.5° N, 158° E, Hokusei-maru vessel; HUMZ 124775, male 405 mm TL, Northwestern Pacific, 10.6° N, 175° E; HUMZ 203771, male 450 mm TL, Northwestern Pacific, Japan, Ogasawara Is., 27° 8'59.32" N, 142°15'56.73" E; HUMZ 208882, male 224 mm TL, Northwestern Pacific, 12° N, 142° E, 14 Jun 2010; MCZ 1368S, female 463 mm TL, Northwestern Pacific, Japan, Sagami Bay, 35°7’ N, 139°22’6” E, Dec 1906; NMW 50132, male 361, Northwestern Pacific, Singapore, 1877; SCSFRI S07257 (Holotype of *Isistius labialis*), female 442 mm TL, Northwestern Pacific, South China Sea, 18°41’-19°32’ N, 112°31’-113°57’ E, depth 520 m, Oct 1980; SIO 80-176, male 382 mm TL, Northwestern Pacific, Taiwan, 28°07.5’ N, 146°11’ E, depth 3000 mwo; UF 79884, male, Northwestern Pacific, Papua-New-Guinea; UW 021822, female 394 mm TL, Northwestern Pacific, 42°1’0.12” N, 158°1’ W, 9 Oct 1989; UW 047600, male 490 mm TL, Northwestern Pacific, Japan, 39°46’60” N, 160°55’ E, 1 Aug 1991;

Northeastern Pacific Ocean (16). ANSP 111459, female 322 mm TL, Northeastern Pacific, Hawaii, Honolulu, Jul 1919; BPBM 5559, ? 280 mm TL, Northeastern Pacific, Hawaii, Oahu, Waikiki, 19 Oct 1966; BPBM 24959, female 255 mm TL, Northeastern Pacific, Hawaii, off Kailua Kona, 0-308 m, 19 Ago 1964; LACM 46046-1, male 325 mm TL, Northeastern Pacific, Hawaii, 10 Sep 1785; SIO 73-157, male 412 mm TL, Northeastern Pacific, Hawaii, 28°20.3’ N, 155°07.2’ W, depth 3000 mwo; SIO 73-413, 2 specs., males 403 and 423 mm TL, Northeastern Pacific, Hawaii, 23°14’ N, 152°44’ W; SIO 78-183, male 393 mm TL, Northeastern Pacific, 29°26.5’ N, 119°44’ W; USNM 190033, 228 mm TL, Northeastern Pacific, Hawaii, 17°29.30’ N, 157°05’ W; USNM 215947, male 364 mm TL, Northeastern Pacific, Hawaii, 21°24’54” N, 158°24’54” W; USNM 215948, C&S, Northeastern Pacific, Hawaii, 21°15’ N 158°15’ W; USNM 418822, female 192 mm TL, Northeastern Pacific, Hawaii, 20°58’48” N, 158°28’54” W; USNM 418823, male 195 mm TL, Northeastern Pacific, Hawaii, 21° 22'24" N, 158° 14'36" W; USNM 418838, female 250 mm TL, Northeastern Pacific, Hawaii, 21°59’36” N, 158°27’18” W; UW 021809, male 403 mm TL, Northeastern Pacific, 40°7’0.12” N, 157°15’ W, 8 Oct 1989; UW 021895, female 466 mm TL, Northeastern Pacific, 37°22’0.12” N, 170°31’0.1” W, 28 Aug 1989;

Central Pacific Ocean (39). BPBM 3504, male 400 mm TL; BPBM 24471, female 363 mm TL, Central Pacific, 3°32’ N, 144°59’ W, depth 50 m, 22 May 1969; BPBM 24490, 2 specs., male 220 and 260 mm TL, Central Pacific, 1° N, 144°50’ W, depth 50 m, 25 May 1969; BPBM 24500, female 266 mm TL, Central Pacific, 0° N, 145° W, depth 50 m, 25 Jul 1969; BPBM 24501, female 332 mm TL, Central Pacific, 0° N, 145° W, depth 50 m, 26 Jul 1969; BPBM 24502, 2 specs., female 364 and 402 mm TL, Central Pacific, 0° N, 145° W, depth 20-100 m, 29 Jul 1969; BPBM 24506, male 350 mm TL, Central Pacific, 3°30’ S, 145° W, depth 75 m, 3 Aug 1969; BPBM 24538, 2 specs., male 165 and 230 mm TL, Central Pacific, 3°29’ N, 144°54’ W, depth 50 m, 30 Oct 1969; BPBM 24543, female 211 mm TL, Central Pacific, 03°30’ S, 145° W, depth 20 m, 4 Nov 1969; BPBM 24544, female 227 mm TL, Central Pacific, 3°11’ S, 145°1’ W, depth 80 m, 6 Nov 1969; BPBM 24545, 4 specs., male and female 211-360 mm TL, Central Pacific, 0°10’ N, 144°36’ W, depth 50 m, 9 Nov 1963; BPBM 24548, ? 240 mm TL, Central Pacific, 0°03’ N, 144°31’W, depth 50 m, 11 Nov 1969; BPBM 24549, 2 specs., male 272 and 217 mm TL, Central Pacific, 0° N, 144°44’ W, depth 50 m, 13 Nov 1969; BPBM 25284, female 204 mm TL, Central Pacific, 04°05’ S, 167°51’ W, depth 120-135 m, 14 Feb 1966; BPBM 26281, female 240 mm TL, Central Pacific, 7°52’ S, 135°03’ W, depth 0-200 m, 21 Aug 1956; BPBM 26909, ? 249 mm TL, Central Pacific, 0°44’ S, 149°46’ W, depth 320 feet, 2 Nov 1958; LACM 46044-1, male 215 mm TL, Central Pacific, 5° N, 145° W; LACM 46047-1, male 191 mm TL, Central Pacific, 0° N 145° W, 9 Nov 1969; LACM 46048-1, male 161 mm TL, Central Pacific, Polynesia, 4°25’ S, 142°44’ W, 1 Dec 1960; LACM 55936-1, female 220 mm TL, Central Pacific, 0°30’ N, 25° W, 23 Feb 1963; MNHN 1996-0725, male 425 mm TL, Central Pacific, 0°1'59" N, 139°55'59" W, depth 700 m, Campagne Caride 5, 177, 1824; MNHN 1996-0726, male 377 mm TL, Central Pacific, 0° N, 139°52’59” W, depth 750 m, Campagne Caride 5, 166, 1969; MNHN 1996-0727, male 411 mm TL, Central Pacific, 0°01’59” S, 140°04’01” W, depth 510 m, Campagne Caride 5, 263, 1969; MNHN 1996-0728, male 405 mm TL, Central Pacific, 0°01’59” S, 140°04’01” W, depth 510 m, Campagne Caride 5, 263, 1969; MNHN 1996-0892, male 206 mm TL, Central Pacific, 9,9° S, 141,88° W, Campagne Caride 5; MNHN 1997-3577, female 266 mm TL, Central Pacific, 0° N, 140°52' W, depth 1180 m, Campagne Caride 3, 1969; MNHN 1997-3739, male ?, Central Pacific, 10° S, 140° W, Campagne Caride 3; USNM 164174, female 187 mm TL, Central Pacific, Line Is., 2°09’ N, 158°14’ W; USNM 190034, female 374 mm TL, Central Pacific, 0°5’ N, 159°51’ W; USNM 190039, male 344 mm TL, Central Pacific, Line Is., 3°56’ N, 150° W; USNM 221045, female 448 mm TL, Central Pacific, Line Is., 0°01’ S, 149°44’ W; ZMH 10215, female 209 mm TL, Central Pacific, 0° N, 146°05‘00” W, 1908;

Indian Ocean (2). MNHN 0000-1178 (Holotype of *Isistius brasiliensis* Quoy & Gaimard, 1824 and *Scymnus unicolor* var. Valenciennes [A.] in Müller & Henle 1839), female 471 mm TL, Indian Ocean, Reunion Is., 20° S, 55° E; NMW 60844, female 318 mm TL, Indian, Mauritius, 1887;

No Data (13). HUMZ 126957, female ? mm TL; HUMZ 126959, female ? mm TL, Hokuyo-maru vessel; HUMZ 126960, male 385 mm TL, Hokuyo-maru vessel; HUMZ 126961, female ? mm TL, Hokuyo-maru vessel; HUMZ 126962, female 475 mm TL, Hokuyo-maru vessel; HUMZ 177913, male 410 mm TL; NHM 1996.2.14.33, female 427 mm TL; NHM 1998.8.9.12113, male 372 mm TL; NHM 2000.2.24.13, female 376 mm TL; USNM 164173, male 167 mm TL; USNM 221043, female 463 mm TL; USNM 221044, 2 specs., female 380 mm TL and male 389 mm TL.

***Isistius plutodus*** (9 specimens)

Southwestern Atlantic Ocean (2). ZUEC-PIS 8332, female, 329 mm TL, Rio Grande do Sul coast, Brazil, 30°30'S, 50°06'W, longline, 1300m depth, 17 Sep 1987; ZUEC-PIS 8333, adult male, 333 mm TL, Eastern coast of Brazil, from Bahia to Rio Grande do Sul, 17°-35°S, 27°-52°W, longline.

Northwestern Atlantic Ocean (3). TU 204003, adult male, 312 mm TL, Northwestern Atlantic, Gulf of Mexico, 27°12'N, 90°26'W, trawl, 675m depth, 01 Feb 2010; UF 232954, female ? mm TL, Northwestern Atlantic, Biscayne Bay, 1976; USNM 188386 (Holotype), female 416 mm TL, Northwestern Atlantic, Gulf of Mexico, Mississippi Delta, 28°58’ N, 88°18’ W, depth 545-445 fms, 27 Oct 1960;

Northeastern Atlantic Ocean (1). ZMH 9368, female 426 mm TL, Northeastern Atlantic, 44°00,82' N, 28°31,37' W, 03 Aug 2000;

Southwestern Pacific Ocean (2). AM I.43044-001, adult male 334 mm TL, Southwestern Pacific, Australia, Coral Sea, 14°17'31" S, 151°21'10" E, 1995; AM I.28924-001, female ? mm TL, Southwestern Pacific, Australia, New South Wales, off Newcastle, 33° S, 152° E, 1988;

Northwestern Pacific Ocean (1). HUMZ, adult male 330 mm TL, Northwestern Pacific, off Ogasawara, 29° N, 141°55’ E, depth 40-780 m, 19 Dec 2010.

***Dalatias licha*** (32 specimens)

Northwestern Atlantic Ocean (1). USNM 157844, female 370 mm TL, Northwestern Atlantic, Gulf of Mexico, 29°11’ N, 88°03’ W;

Northeastern Atlantic Ocean (5). AMNH 1217, ? embryo 143 mm TL, Northeastern Atlantic, Mediterranean Sea, Italy; MCZ 910, female 370 mm TL, Northeastern Atlantic, Mediterranean Sea, France, Jul 1885; MCZ 949, female 422 mm TL, Northeastern Atlantic, Mediterranean Sea, France, Jul 1886; MCZ 966, female embryo 234 mm TL, Northeastern Atlantic, Mediterranean Sea, Sicily, 37°59’ N, 12°25’ E, Jun 1890; UF 42106, male 358 mm TL, Northeastern Atlantic, Mediterranean Sea, Dec 1984;

Southwestern Pacific Ocean (17). AMS E.3702 (Holotype), male 1160 mm TL, Southwestern Pacific, Australia, South Australia, Great Australian Bight, 127°-129° E; AMS E.12876 (Paratype), male 496 mm TL, Great Australian Bight, Southwestern Pacific, Australia, 32° S, 127° E, Jun 1913; AMS I.15975-018, female 560 mm TL, Southwestern Pacific, Australia, New South Wales, 35 miles SE of Newcastle, 33°10'58.80" S, 152°22'58.80" E, 29 Apr 1971; AMS I.15990-009, female 560 mm TL, Southwestern Pacific, Australia, New South Wales, 30 miles NE of Batemans, 35°32'60.00" S, 150°42'57.60" E, 08 Jul 1971; AMS I.16596-007, male 455 mm TL, Southwestern Pacific, Australia, New South Wales, off Sydney, 33°27' S, 152° 6'57.60" E, 10 Oct 1972; AMS I.18125-001, 8 specs., male 391 mm TL, female 393 and 400 mm TL, Southwestern Pacific, Australia, New South Wales, east of Broken Bay, 33°34'58.80" S, 151°30' E, 11 Dec 1974; AMS I.18764-007, male 470 mm TL, Southwestern Pacific, Australia, New South Wales, east of Kembla harbour, 34°32'60.00" S, 151°18' E, 29 May 1975; AMS I.19082-005, female 408 mm TL, Southwestern Pacific, Australia, New South Wales, NE of Crowdy Head, 9 Nov 1975; AMS I.19198-004, female 396 mm TL, Southwestern Pacific, Australia, New South Wales, east of Broken Bay, 33°51' S, 151°40'58.80" E, 04 May 1976; AMS I.43469-001, male 414 mm TL, Southwestern Pacific, Australia, Ulladulla, 35°21' S, 150°28'58.80" E, 12 Aug 1993;

Northwestern Pacific Ocean (2). MZUSP 123085, female 468 mm TL, Northwestern Pacific, Taiwan, Chang-b.h., 19 Mar 2012; UF 159698, female 373 mm TL, Northwestern Pacific, Taiwan, Tashi fish market, 25 May 2005;

Southwestern Indian Ocean (5). CSIRO H5823-01, male 415 mm TL, Southwestern Indian, 33°13’ S, 44°34’ E, depth 970 m, 2000; HUMZ 74173, male 450 m TL, Southwestern Indian, 33°21.5’ S, 44°16.1’ E, depth 782 m, 20 Jun 1977; HUMZ 74586, male 445 mm TL, Southwestern Indian, 33°19’ S, 44°13’ E, depth 770 m, 04 Ago 1977; HUMZ 74605, female 472 cm TL, Southwestern Indian, 33°19’ S, 44°13’ E, depth 770 m, 04 Aug 1977; SAIAB 189435, male 575 mm TL, Southwestern Indian, Off Northern Madagascar, 12°28’ S, 48°10’ E, 27 Sep 2009;

No Data. AMNH 19446, female embryo 311 mm TL, 19 Out 1909; HUMZ M392, male 408 mm TL; NSMT P68123, male 484 mm TL.

***Squaliolus laticaudus*** (23 specimens)

Northwestern Atlantic Ocean (6). UF 176528, female 106,84 mm TL, Northwestern Atlantic, Gulf of Mexico, 27°53’23.4” N, 91°9’58.2” W, depth 274 m; USNM 187941, female 137,51 mm TL, Northwestern Atlantic, Gulf of Mexico, 28°59’ N, 88°37’ W; USNM 365693, 4 specs., Northwestern Atlantic, Gulf of Mexico, 29°11’ N, 87°55’ W, 21 Nov 1961;

Northeastern Atlantic Ocean (1). ANSP 106248, male 219 mm TL, Northeastern Atlantic, Madeira Is., Funchal, 1947;

Southweastern Atlantic Ocean (1). MNRJ 30199, male 216 mm TL, Southwestern Atlantic, Brazilian continental slope, REVIZEE D-463, 22 Jun 1999;

Northwestern Pacific Ocean (16). AMNH 239379, female 116,24 mm TL, Northwestern Pacific, Taiwan, Tungkan harbour, 22.27°58.3’ N, 120.26°38.6’ E; AMNH 242686, female 224,7 mm TL, Northwestern Pacific, Taiwan, Suao, Nan Fassao fish market, 23 Mar 2007; HUMZ 74973, female 254 mm TL, Northwestern Pacific, Kyushu-Palau ridge, 29 Jan 1978; HUMZ 74975, female 232 mm TL, Northwestern Pacific, Kyushu-Palau ridge, 11 Feb 1978; LACM 36021-1, female 125,56 mm TL, Northwestern Pacific, Philippines, Sulu Sea, 8°18.7’ N, 121°12’ E, 4 Jun 1975; LACM 36022-1, female 94,96 mm TL, Northwestern Pacific, Philippines, Sulu Sea, 3 Jun 1975; LACM 36279-2, female 106,31 mm TL, Northwestern Pacific, Japan, Suruga Bay, Mar Jun 1976; LACM 36279-7, female 127,24 mm TL, Northwestern Pacific, Japan, Suruga Bay, Mar Jun 1977; SIO 00-155, female 172,25 mm TL, Northwestern Pacific, Taiwan, 22°13’ N, 120°32’ E, depth 200-300 m; SIO 00-178, female 91,71 mm TL, Northwestern Pacific, Taiwan, 22°16’ N, 120°19’ E, depth 200 m; SIO 83-127, 3 specs., male 101,91 mm TL, male 120,67 mm TL, Northwestern Pacific, Japan, 35°15’ N, 139°10’ E; USNM 70259 (Holotype), male 142,98 mm TL, Northwestern Pacific, Philippines, Batangas Bay; USNM 76679 (Paratype), female 108,33 mm TL, Northwestern Pacific, Philippines; USNM 398487, Northwestern Pacific, Taiwan, Dong-gang fish market, 12 Nov 2009;

Southeastern Indian Ocean (3). CSIRO CA3355, female 144,54 mm TL, Southeastern Indian, Western Australia, SW of Rowley Shoals, 18°40’ S, 117°11’ E, depth 398 m, 1982; CSIRO H1215, male 158,57 mm TL, Southeastern Indian, Western Australia, 1988; CSIRO H1624-01, female 170,08 mm TL, Southeastern Indian, Western Australia, close to Rowley Shoals, 17°46’ S, 118°42’ E, depth 375 m, 1988;

No Data (1). HUMZ 95249, female 245 mm TL, ?, 05 Nov 1981.

***Squaliolus aliae*** (15 specimens)

Northwestern Pacific Ocean (11). BPBM 32767, female 100.93 mm TL, Northwestern Pacific, Japan, Honshu, Suruga Bay, 29 May 1979; BPBM 32768, male 110.01 mm TL, Northwestern Pacific, Japan, Honshu, Suruga Bay, 03 Jun 1979; CSIRO H6296-05, female 108.95 mm TL, Northwestern Pacific Taiwan, Tashi fish market; CSIRO H6297-04, female 124.81 mm TL, Northwestern Pacific Taiwan, Tashi fish market; CSIRO H7403-03, female 201.16 mm TL, Northwestern Pacific Taiwan, Kaohsiung; HUMZ 119285, female 179 mm TL, Northwestern Pacific, Japan; NMMBA 11102, female 84.59 mm TL, Northwestern Pacific, TongKang Fishmaket, 12 Jan 2011; NMMBA 15220, female 199.4 mm TL, Northwestern Pacific, Changbin, Taitung, 17 Nov 2011; USNM 399935, female 128.1 mm TL, Northwestern Pacific, Taiwan; USNM 402441, 2 specs., female 196 mm TL, and male 146 mm TL, Northwestern Pacific, Taiwan.

Southwestern Pacific Ocean (4). AMS I.20515-001, female 97 mm TL, Southwestern Pacific, Australia, East of Sydney; AMS I.30411-001, female 215 mm TL, Southwestern Pacific, Australia, East of Kiama; AMS I.42175-004, male 85 mm TL, Southwestern Pacific, Coral Sea; AMS I.43064-001, male 134 mm TL, Southwestern Pacific, Coral Sea;

Eastern Indian Ocean (1). CSIRO H6416-02, female 189.46 mm TL, Eastern Indian, Northwestern Australia, Ashmore Terrace

***Euprotomicrus bispinatus*** (31 specimens)

South Atlantic Ocean (1). LACM 55938-1, male 207 mm TL, South Atlantic, 35°18’ S, 18°39’ W, 16 Apr 1971;

Southeastern Atlantic Ocean (1). LACM 55939-1, female, Southeastern Atlantic, 15°59’ S, 2°2’ E, 31 May 1971;

Southwestern Pacific Ocean (2). MCZ 41717, male 97 mm TL, Southwestern Pacific, Fiji, 18°21’ S, 178°21’ E, 20 Nov 1928; SIO 76-237, male 223 mm TL, Southwestern Pacific, New Zealand, 37°48.2’ S, 175°46.7’ W, surface;

Southeastern Pacific Ocean (7). MCZ 45900, male 204.4 mm TL, Southeastern Pacific, 33°48’ S, 90°17’ W, surface, 19 Jan 1966; SIO 58-309, male 159.44 mm TL, Southeastern Pacific, 14°17’ S, 108°52’ W, surface; SIO 72-4, male 130.15 mm TL, Southeastern Pacific, 13°08’ S, 122°19’ W, surface; SIO 76-119, male 205.02 mm TL, Southeastern Pacific, French Polynesia, 27°59.6’ S, 155°02’ W, surface; SIO 76-235, male 208.82 mm TL, Southeastern Pacific, French Polynesia, 24°25.8’ S, 154°57.5’ W, surface; SIO 76-236, male 189.11 mm TL, Southeastern Pacific, French Polynesia, 25°15’ S, 154°59.8’ W, surface; USNM 190032, male 111 mm TL, Southeastern Pacific, 13°38’ S, 110°34’ W.;

Northeastern Pacific Ocean (17). BPBM 33481, male 154.97 mm TL, Northeastern Pacific, Hawaii, 28 Aug 1987; BPBM 40404, female 257 mm TL, Northeastern Pacific, off California, 85 miles SW of Long Beach; BPBM 40983, 2 specs., male 150.82 mm TL, female 256 mm TL, Northeastern Pacific, Hawaii, surface, 27 Apr 2009; BPBM 25281, Northeastern Pacific, Hawaii; CAS 20431, female 225 mm TL, Northeastern Pacific, off California, 19 Nov 1948; LACM 55941-1, male 223 mm TL, Northeastern Pacific, Mexico, 30°15’ N, 116°27’ W; LACM 6988-1, female 204 mm TL, Northeastern Pacific, 35°08’ N, 137°28’ W, 20 May 1961; LACM 6989-1, female 224 mm TL, Northeastern Pacific, 35°13’ N, 130°36’ W, 02 Jun 1961; LACM 6990-1, female 266 mm TL, Northeastern Pacific, 33°11’ N, 131°24’ W, 05 Jun 1965; SIO 66-111, male 206.1 mm TL, Northeastern Pacific, 29°51’ N, 175° W, surface; SIO 68-432, male 169.28 mm TL, Northeastern Pacific, Hawaii, 22° N, 155°50’ W, surface; SIO 98-173, 2 specs., male 196.2 mm TL, female 235 mm TL, Northeastern Pacific, 29°15’ N, 144°43’ W, surface; USNM 164176, female 161 mm TL, Northeastern Pacific, 18°24’ N, 175°12’ W; UNSM 190031, female 240 mm TL, Northeastern Pacific, Midway Island, 32°46’ N, 176°42’ W; UW 110386, male 133.56 mm TL, Northeastern Pacific, Hawaii, 23°46’60” N, 163°41’60” W, 11 Nov 2002;

Indian Ocean (2). MNHN 0000-1216 (Holotype), female 196 mm TL, Indian, Mauritius, 20° S, 55° E; SIO 66-110, male 205 mm TL, Indian, Maldives, 1° S, 74° E, surface;

No Data (1). LACM 46049-1, female 205 mm TL, 01 Jul 1979.

***Heteroscymnoides marleyi*** (1 specimen)

Southwestern Indian Ocean (1). ANSP 53046 (holotype), female 122.56 mm TL, Indian Ocean, South Africa, Natal, 01 Apr 1923.

***Mollisquama parini*** (1 specimen)

Northwestern Atlantic Ocean (1). TU 203676, male 145 mm TL, Northwestern Atlantic, Gulf of Mexico, 04 Feb 2010.
