# Supplementary material for: Comparative morphology and systematics of the cookiecutter sharks, genus Isistius Gill (1864) (Chondrichthyes: Squaliformes: Dalatiidae)
Source: PLoS One. 2018 Aug 20;13(8):e0201913. doi: 10.1371/journal.pone.0201913 (PMC6101376; doi:10.1371/journal.pone.0201913)
Supplement: S2 File — (DOCX) [file pone.0201913.s002.docx]

**S2 FILE**

List of reported wounds of cookiecutter shark bites on cetaceans, fishes, a turtle, and a human corpse.

| **Reference** | **Species** | **Wounds on body** | **Geographical**  **location** |
| --- | --- | --- | --- |
| Addink & Smeenk, 2001 [47] | *Steno bredanensis* | - | Mauritania |
| Alcorn & Westlake, 1993 [133] | *Monachus schauinslandi* | - | Northeastern Hawaiian Islands |
| Azevedo *et al.*, 2003 [173] | *Lagenodelphis hosei* | - | Southwestern Atlantic |
| Baird *et al.*, 2001 [162] | *Stenella attenuata* | - | Hawaii |
| Baird *et al.*, 2008 [323] | *Mesoplodon densirostris*, *Ziphius cavirostris* | - | Hawaii |
| Belcher & Lee, 2002 [169] | *Arctocephalus townsendi* | - | Northeastern Pacific |
| Bermúdez Villapol *et al.*, 2008 [221] | *Kogia sima* | - | Venezuela |
| Bertilsson-Friedman, 2006 [199] | *Monachus schauinslandi* | - | Hawaii |
| Best, 2001 [307] | *Balaenoptera edeni* | Posterior half of body | West coast of southern Africa |
| Bonde & O’Shea, 1989 [294] | *Mesoplodon bidens* | Both flanks | Gulf of Mexico |
| Borsa & Robineau, 2005 [316] | *Mesoplodon densirostris* | On back | New Caledonia |
| Bossart *et al.*, 2007 [208] | *Peponocephala electra* | - | Florida |
| Brownell, Jr. *et al.,* 2007 [209] | *Balaenoptera musculus* | - | North of Chile |
| Bunkley-Williams *et al.,* 2008 [222] | *Eretmochelys imbricata* | - | Northeastern Australia |
| Burdin *et al*., 2007 [210] | *Orcinus orca* | - | Russian far east |
| Choy & Hiruki, 1992 [130] | *Monachus schauinslandi* | - | Pearl and Hermes Reef, Hawaii |
| Claridge, 2006 [319] | *Mesoplodon densirostris* | - | Intertropical, Worldwide |
| Dalebout *et al.*, 2002 [310] | *Mesoplodon perrini* | On flanks | Monterey, Northeastern Pacific |
| Dalebout *et al.*, 2004 [313] | *Mesoplodon grayi* | Below right flank | New Zealand |
| Deakos *et al.,* 2010 [245] | *Tursiops truncatus* | On right side of body | Hawaii |
| Delgado-Estrella *et al.*, 1998 [148] | *Kogia breviceps* | Left pectoral fin | Gulf of Mexico |
| Dwyer & Visser, 2011 [257] | 1. *Balaenoptera brydei, Balaenoptera musculus, Megaptera novaeangliae, Delphinus* sp.*, Globicephala melas, Pseudorca crassi-dens, Orcinus orca* | - | New Zealand |
| Falcón-Matos, *et al.,* 2003 [339] | *Ziphius cavirostris* | - | Gulf of Mexico |
| Fitzpatrick *et al.*, 2006 [320] | *Rhincodon typus* | Left flank | Ningaloo Reef in Western Australia |
| Foster & Hare, 1990 [297] | *Ziphius cavirostris* | Abdominal region | Northeastern Pacific |
| Gasparini & Sazima, 1996 [145] | *Peponocephala electra* | Whole body | Southeastern coast of Brazil |
| Goto *et al*., 2009 [238] | *Balaenoptera* sp. | - | Northwestern Pacific |
| Haney *et al.*, 2004 [181 | *Globicephala macrorhynchus* | - | Northeastern coast of Brazil |
| Heithaus, 2001 [164] | *Stenella longirostrus* | Whole body, except appendages | Hawaii |
| Hiruki *et al.*, 1993 [136] | *Monachus schauinslandi* | - | Hawaii |
| Honebrink *et al.*, 2011 [349] | *Homo sapiens, Ruvettus pretiosus* | Lower chest, left calf | Hawaii |
| Hoyos-Padilla *et al.*, 2012 [261] | *Carcharodon carcharias* | Lateral to corner of mouth | Guadalupe Island, Northwestern Pacific |
| Ito *et al.*, 1994 [139] | *Gasterochisma melampus* | Posteriorly, above opercle | Hawaii |
| Jefferson & Barros, 1997 [303] | *Peponocephala electra* | - | Tropical and subtropical zones throughout the world |
| Johnson & Wolman, 1984 [293] | *Megaptera novaeangliae* | - | Artic to Antarctic |
| Le Beouf & McCosker, 1987 [120] | *Mirounga angustirostris* | Posterior side of flippers, on ventral side or back, and on either side of midline, on chest and neck, and just behind ear | Northeastern Pacific |
| MacLeod, 1998 [150] | *Kogia breviceps* | - | Tropical and temperate waters of the Atlantic, Pacific and Indian Oceans |
| Makino *et al.*, 2004 [183] | *Homo sapiens*, *Lampris guttatus* | Temporal region of head, upper region of back, and right elbow | Okinawa, Japan |
| McSweeney *et al.*, 2007 [322] | *Mesoplodon densirostris, Ziphius cavirostris* | - | Hawaii |
| Mikhalev, 1997 [146] | *Balaenoptera borealis, B. musculus* and *B. musculus brevicauda* | - | Arabian Sea |
| Mincarone *et al.*, 2001 [165] | *Trachipterus jacksonensis* | - | Southeastern coast of Brazil |
| Moore *et al.*, 2003 [177] | *Balaenoptera physalus, Megaptera novaeangliae* | - | Cape Verde Islands |
| Mullin *et al.*, 1994 [141] | *Stenella clymene* | - | Gulf of Mexico |
| Neto *et al.*, 2008 [325] | *Megaptera novaeangliae* | Ventral region | Northeastern coast of Brazil |
| Norris & Dohl, 1980 [114] | *Stenella longirostris* | - | Hawaii |
| Ohishi *et al.*, 2007 [215] | *Kogia breviceps* | - | Pacific coast of Japan |
| Papastamatiou *et al.*, 2010 [250] | *Xiphias gladius, Thunnus obesus, Thunnus albacares, Katsuwonus pelamis, Taractichthys steindachneri, Lampris guttatus, Acanthocybium solandri, Makaira mazara, Tetrapturus audax, Tetrapturus angustirostris* | - | Hawaii |
| Pérez-Zayas *et al*., 2002 [171] | *Makaira*  *nigricans, Coryphaena hippurus, Thunnus albacares, Thunnus alalunga, Thunnus*  *Atlanticus, Arctocephalus townsendi, Mirounga angustirostris, Balaenoptera musculus, Balaenoptera physalus, Lagenodelphis hosei, Peponocephala*  *electra, Phocoena phocoena, Mesoplodon* spp*., Ziphius cavirostris* | - | Puerto Rico and Virgin Islands |
| Perrin *et al.*, 1981 [117] | *Stenella clymene* | Dorsal region | Caribbean |
| Pitman *et al.*, 2001 [308] | *Orcinus orca* | Behind dorsal fin | Northeastern Pacific, California |
| Reddy & Griffith, 1988 [123] | *Monachus schauinslandi* | Dorsal, chest, and ventral (above right front flipper) regions, and shoulder | Kure Atoll, Hawaii |
| Renner & Bell, 2008 [232] | *Orcinus orca* | Behind blow hole | Adak Island, Alaska |
| Sadowsky *et al.*, 1988 [124] | *Prionace glauca* | Lateral region, close to 5^th^ gill opening | South coast of Brazil |
| Silva-Jr & Sazima, 2003 [178] | *Remora australis* | Right flank | Fernando de Noronha, Northeastern Brazil |
| Silva-Jr *et al.*, 2007 [217] | *Stenella longirostris* | Posterior half of body | Fernando de Noronha, Northeastern Brazil |
| Souto *et al.*, 2007 [218] | *Peponocephala electra, Grampus griseus, Stenella attenuata, Stenella coeruleoalba, Ziphius cavirostris, Physeter macrocephalus, Sotalia guianensis, Mesoplodon layardii, Kogia breviceps, Stenella clymene, Megaptera novaeangliae, Kogia sima, Pseudorca crassidens, Megaptera novaeangliae* | - | Bahia, Northeastern Brazil |
| Souto *et al.*, 2009 [241] | *Arctocephalus tropicalis* | Distal part of dorsal region of left pectoral paddle, posterior portion of back | Bahia, Northeastern Brazil |
| Souza *et al.*, 2005 [318] | *Mesoplodon mirus* | Dorsal region | Brazil |
| Stacey *et al.*, 1994 [142] | *Pseudorca crassidens* | - | Tropical, subtropical, and warm temperate seas |
| Steiner *et al.*, 2008 [324] | *Balaenoptera edeni* | Around dorsal fin | Azores |
| Taylor *et al.*, 1983 [118] | *Megachasma pelagios* | On throat and behind right pectoral fin | Hawaii |
| Van Den Hoff *et al.*, 2005 [198] | *Mirounga leonina* | Above fore flipper | Heard and Macquarie Islands, and Prydz Bay, Southwestern Pacific |
| Van Waerebeek *et al.*, 2008 [233] | *Peponocephala electra* | - | Cape Verde Islands |
| Velozo *et al.,* 2009 [242] | *Arctocephalus tropicalis* | - | Northeastern coast of Brazil |
| Visser, 1999 [154] | *Orcinus orca* | - | New Zealand |
| Walker & Hanson, 1999 [155] | *Mesoplodon stejnegeri* | - | Adak Island, Alaska |
| Wardle *et al.*, 2000 [306] | *Peponocephala electra* | Dorsal surface near base of dorsal fin | Corpus Christi, Texas, Gulf of Mexico |
| Würsig & Jefferson, 1990 [129] | *Stenella atenuata*, *Stenella longirostris* | All over body | Worldwide |
| Zerbini & Santos, 1997 [304] | *Feresa attenuata* | Ventral surface | Brazil |
