# Supplementary material for: Comparative morphology and systematics of the cookiecutter sharks, genus Isistius Gill (1864) (Chondrichthyes: Squaliformes: Dalatiidae)
Source: PLoS One. 2018 Aug 20;13(8):e0201913. doi: 10.1371/journal.pone.0201913 (PMC6101376; doi:10.1371/journal.pone.0201913)
Supplement: S3 File — (PDF) [file pone.0201913.s003.pdf]

American Society of Ichthyologists
and Herpetologists
PUBLISHERS OF "COPEIA"
ΙΧΘΥΣ
ΒΑΤΡΑΧΟΣ
ΕΡΠΕΤΟΝ

Prosanta Chakrabarty
ASIH – Secretary
Museum of Science
Louisiana State University
119 Foster Hall
Baton Rouge, LA 70803-3216

4 May 2018

To Whom It May Concern:

Dear Dr. Petean,

The American Society of Ichthyologists and Herpetologists grants you permission to republish the illustration of:

Fig. 2 of "Garrick JAF, Springer S. *Isistius plutodus*, a new squaloid shark from the Gulf of Mexico. Copeia. 1964;1964(4):678–82"

To be published in PlosOne under a CC BY license.

Please acknowledge the American Society of Ichthyologists and Herpetologists wherever the image is used.

Sincerely,

Prosanta Chakrabarty
ASIH Secretary
225-578-3079
